# Supplementary material for: Repression of apelin Furin cleavage sites provides antimetastatic strategy in colorectal cancer
Source: EMBO Mol Med. 2025 Feb 17;17(3):504–34. doi: 10.1038/s44321-025-00196-5 (PMC11904221; doi:10.1038/s44321-025-00196-5)
Supplement: Supplementary file 1 — Appendix [file 44321_2025_196_MOESM1_ESM.pdf]

Appendix

Repression of apelin Furin cleavage sites provides antimetastatic strategy in colorectal cancer.

Béatrice Demours[1], Fabienne Soulet[1], Jean Descarpentrie[1], Isabel Galeano-Otero[1], José Sanchez Collado[1], Maria Casado[1,2], Tarik Smani,[3] Alvaro González[1], Isabel Alves[4], Fabrice Lalloué[5], Bernard Masri[6], Estelle Rascol[4], Jean-William Dupuy[7], Cyril Dourthe[7], Frédéric Saltel[1,7], Anne-Aurélie Raymond[1,7], Iker Badiola[2], Serge Evrard[1,8], Bruno Villoutreix[9], Simon Pernot[1,8], Géraldine Siegfried[1*], Abdel-Majid Khatib[1*]

[1] University of Bordeaux, Bordeaux Institute of Oncology (BRIC)-UMR1312 Bordeaux, France
[2] Department of Cell Biology and Histology, University of the Basque Country, Bº Sarriena sn 48940 Leioa Spain
[3] Institute of Biomedicine of Seville, University Hospital of Virgen del Rocío/University of Seville/CSIC, Avenida Manuel Siurot s/n, 41013 Seville, Spain.
[4]Univ. Bordeaux, CNRS, Bordeaux INP, CBMN, France
[5]EA3842- CAPTuR, GEIST, Faculté de Médecine , Université de Limoges , 2 rue du Dr Marcland , 87025 Limoges Cedex , France.
[6] Institut Cochin, INSERM U1016, CNRS UMR 8104, Université Paris Cité, 75014 Paris, France.
[7] Oncoprot Platform, TBM-Core US 005, Bordeaux, France.
[8] Institut Bergonié, Bordeaux, France
[9] Université de Paris, Inserm UMR 1141, Robert-Debré Hospital, 75019 Paris, France.

Table of Contents

Appendix Figure S1……1
Appendix Figure S2……2
Appendix Figure S3……3
Appendix Figure S4……4
Appendix Figure S5……5
Appendix Figure S6……6
Appendix Figure S7……6
Appendix Figure S8……7
Appendix Figure S9……8
Appendix Figure S10……9
Appendix Figure S11…… 10
Appendix Figure S12A……11
Appendix Figure S12B……12
Appendix Figure S12C…… 13
Appendix Figure S13…… 14
Appendix_pharmacological_studies_1…… 15
Appendix_pharmacological_studies_2…… 18
Appendix_pharmacological_studies_3…… 21
Appendix_pharmacological_studies_4…… 37

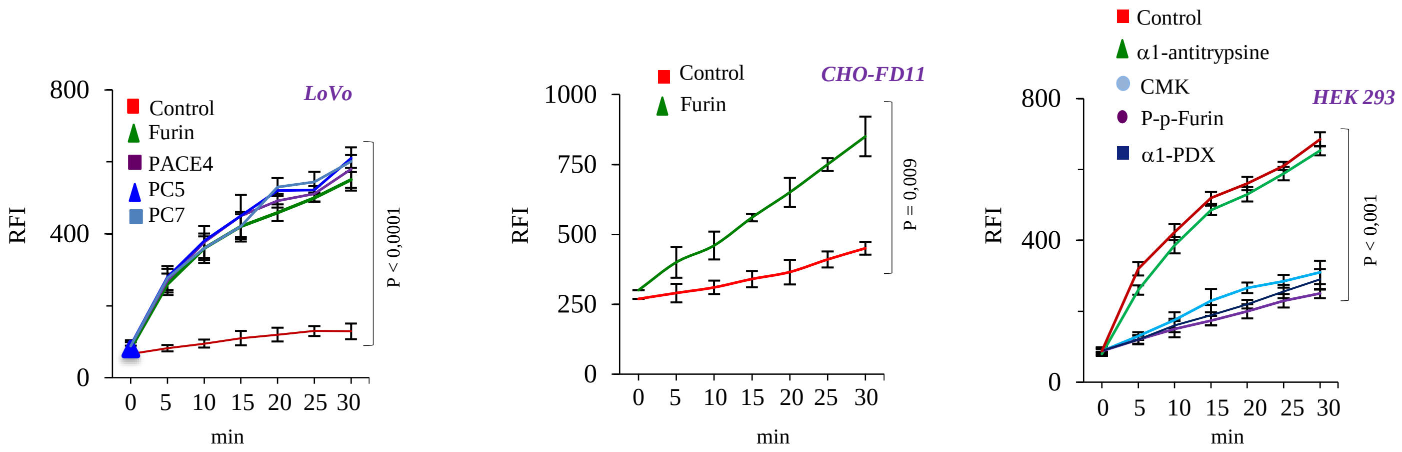

Appendix Figure S1. A, Activity of convertases in transfected LoVo, CHO-FD11 and HEK 293 cells was assessed by enzymatic digestion assay using the substrate, pERTKR-MCA. The data are representative of three independent experiments. All values represent the mean±s.e.m. P values by two-tailed unpaired t-test. RFI : Relative Fluorescence Intensity

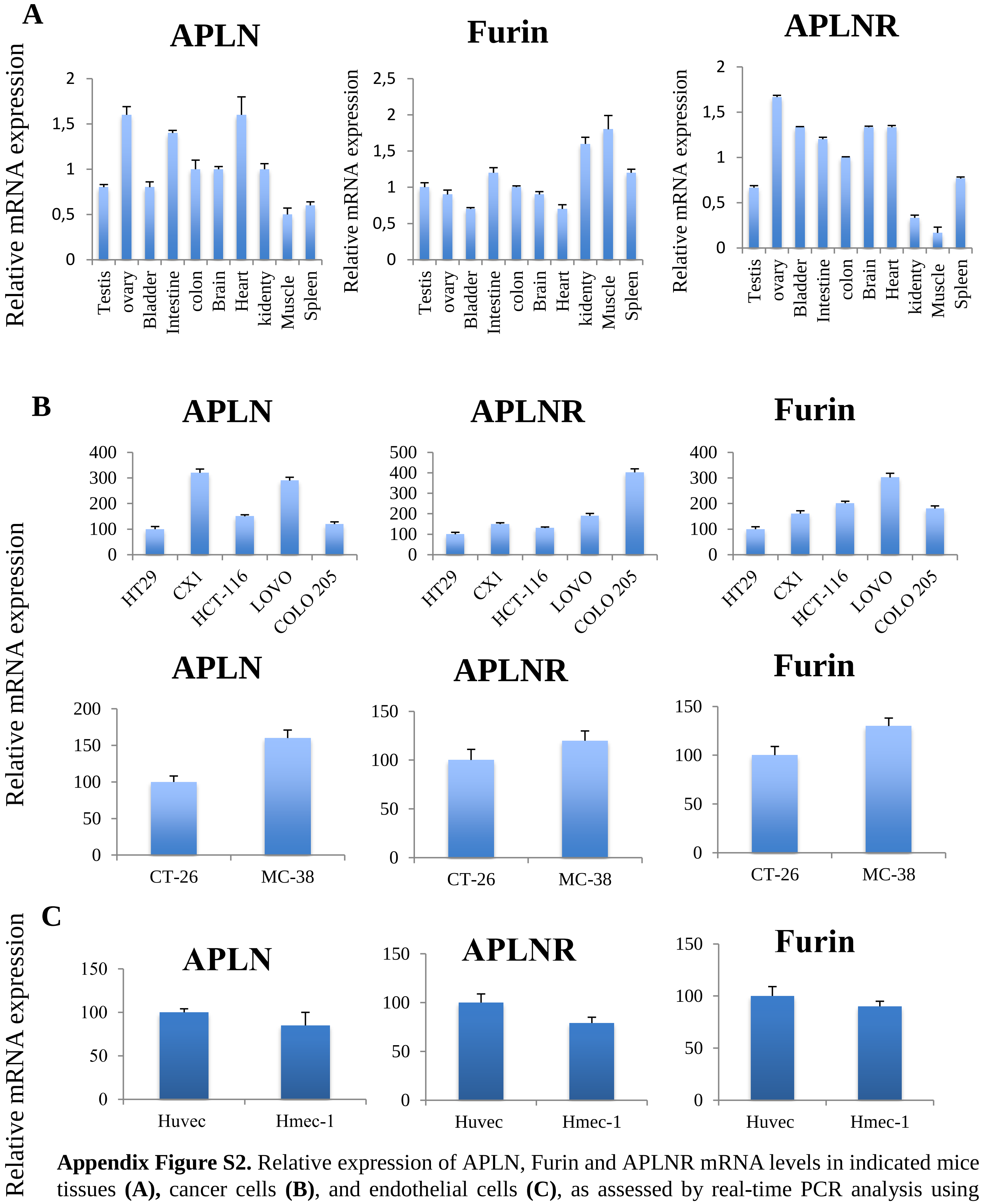

Appendix Figure S2. Relative expression of APLN, Furin and APLNR mRNA levels in indicated mice tissues **(A),** cancer cells **(B)**, and endothelial cells **(C)**, as assessed by real-time PCR analysis using specific primers for APLN, Furin, APLNR or GAPDH. Expression of GAPDH that was evaluated in each sample was used as endogenous control. The relative amounts of mRNA were normalized against GAPDH mRNA and expressed relative to the mRNA abundance in kidney tissue assigned 1. The data are representative of three independent experiments.

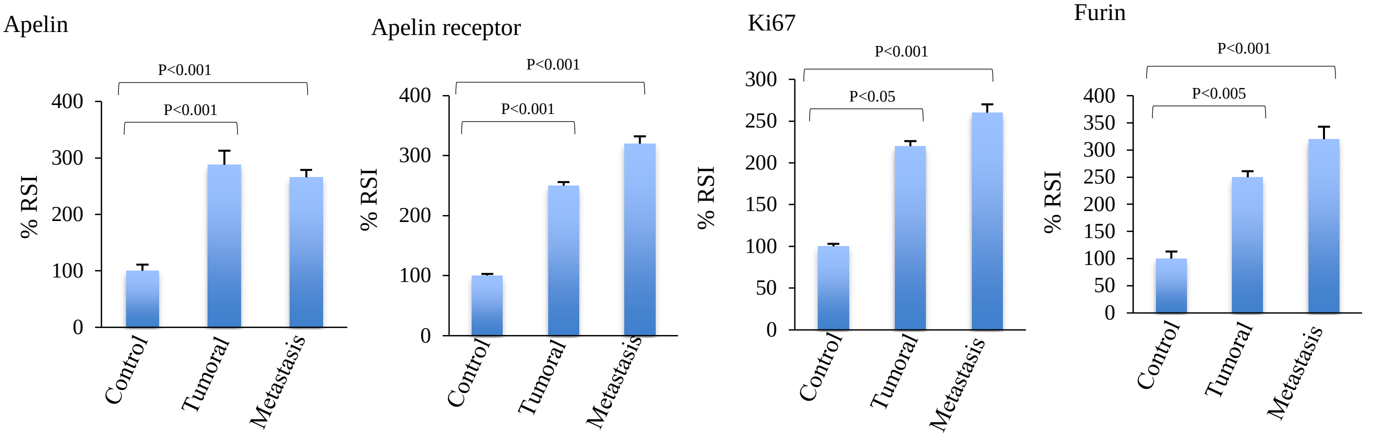

Appendix Figure S3. The percentage of relative staining intensity of apelin, apelin receptor, Ki67, and Furin for indicated tissue derived from 35 pairs of CRC tumors and their corresponding adjacent noncancerous tissues colorectal liver metastasis deduced from **(Fig. 1G, 1H)** . Data are representative of 3 independent experiments as mean±s.e.m. P values by two-tailed unpaired t-test.

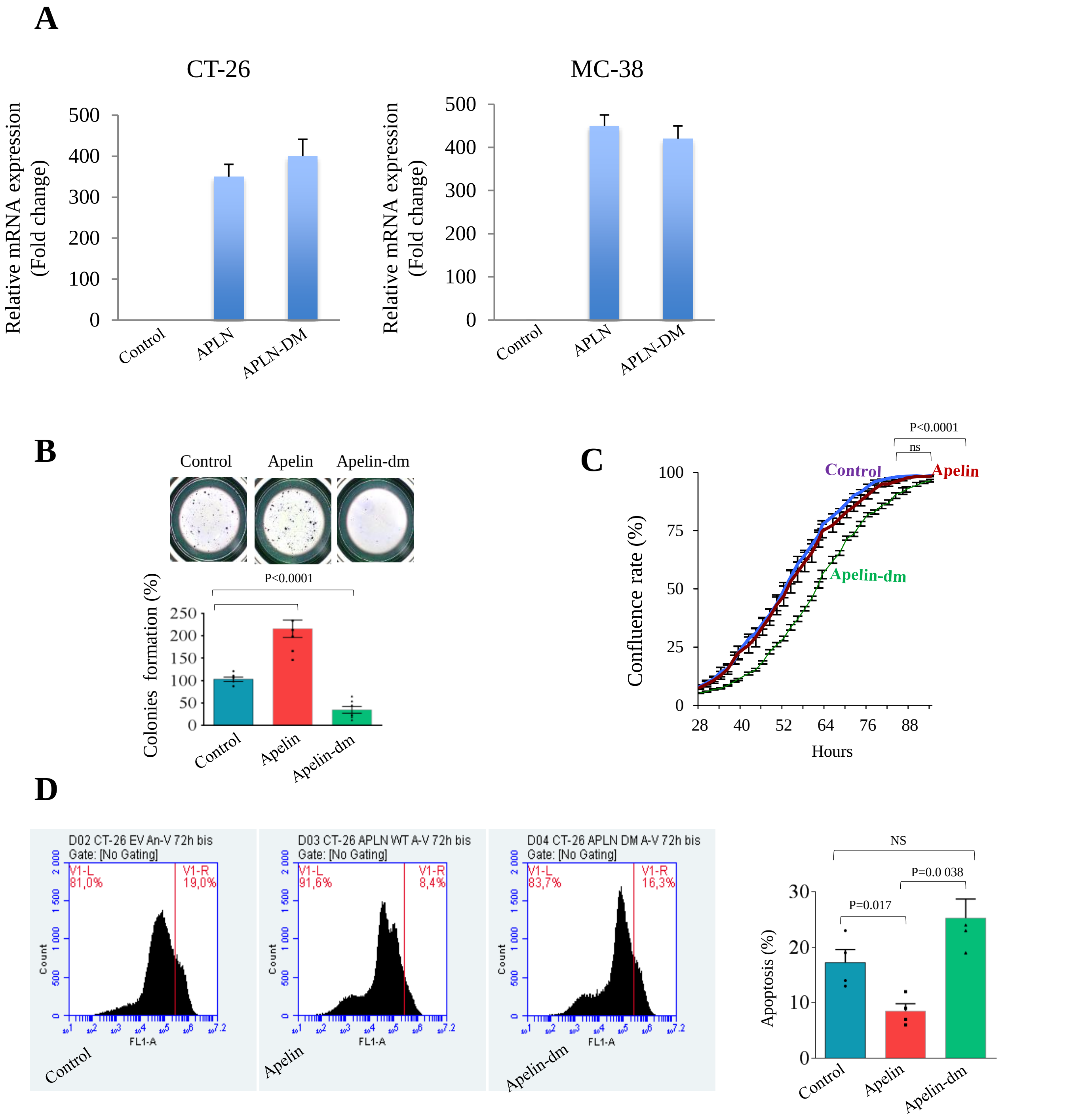

Appendix Figure S4. A, RT-PCR analysis of APLN and APLN-DM expression in colon cancer cells CT-26 and MC-38 infected with lentivirus containing APLN and APLN DM cDNA. **B,** Soft-agar colony forming ability was compared between APLN and APLN-DM-expressing cells. The bars show the percentage of the number of colonies (*n* = 3 per group). **C,** Proliferation of CT-26 cells expressing APLN and APLN-DM-expressing cells as assessed by IncuCyte live-cell microscopy incubator (Essen Bioscience). **D.** Quantification of apoptosis in APLN and APLN-DM-expressing cells (Annexin V-positive cells). All values represent the mean±s.e.m. P values by two-tailed unpaired t-test.

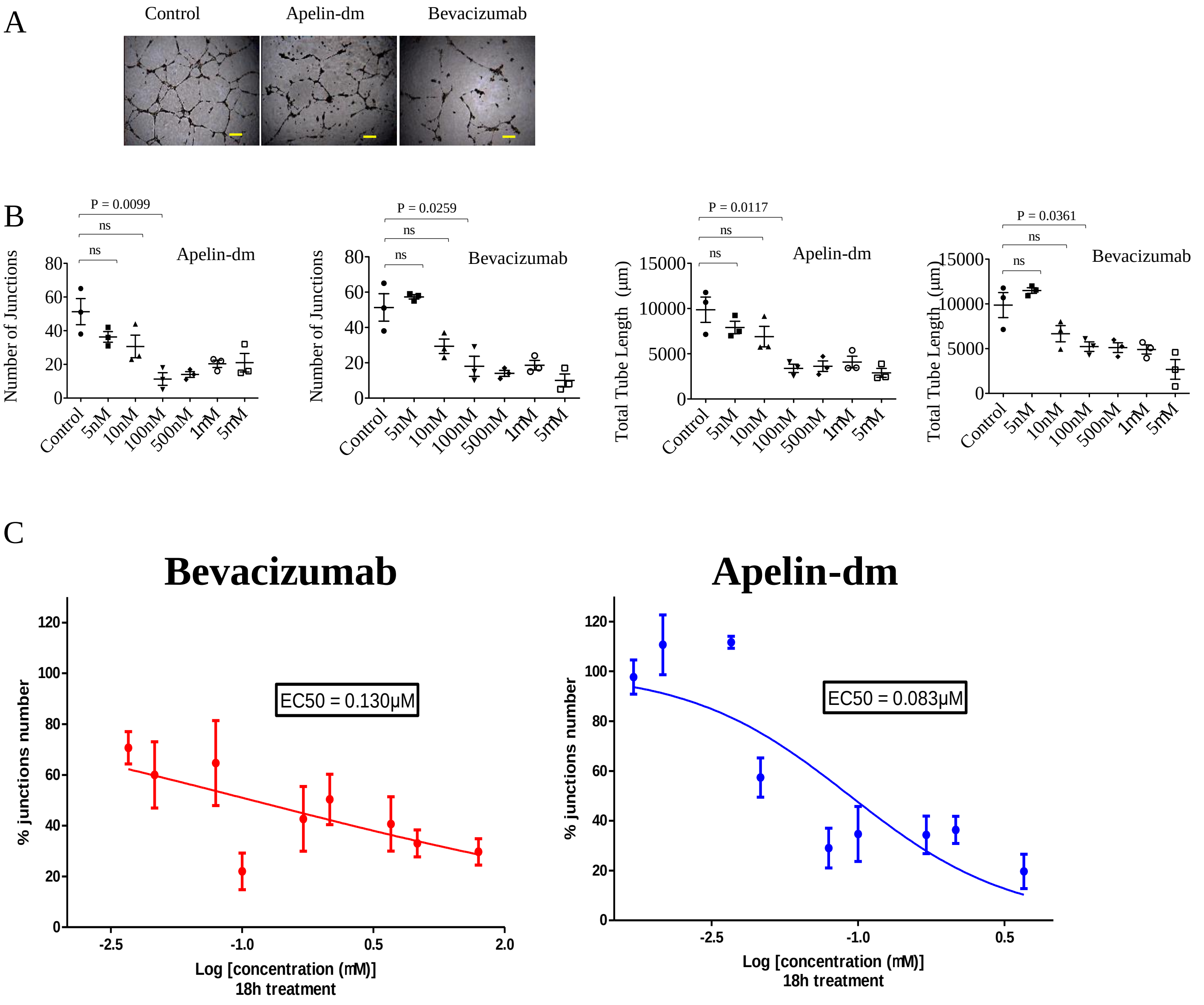

Appendix Figure S5. A, Representative images of apelin-dm and Bevacizumab on tube-like structure formation by HUVEC cells. **B,** Comparative analysis of various concentrations of apelin-dm peptide and Bevacizumab on tube-like structure formation measured by the number of junctions and tubule length. Scale bar indicates 250 µm. The data are representative of three independent experiments. n.s.=not significant. All values represent the mean±s.e.m. Significant differences *P* were determined by Two-way ANOVA. **C,** Determination of EC50 of Bevacizumab and apelin-dm to inhibit angiogenesis on HUVEC cells. EC50 corresponding to a reduction of 50% of junction number has been calculated for Bevacizumab and apelin-dm using GraphPad Prism. Only junctions number have been considered for this calculation as it gives a clear and rapid response of the tube formation inhibition. EC50 of Bevacizumab is equal to 0.083 µM while EC50 of apelin-dm is equal to 0.130 µM.

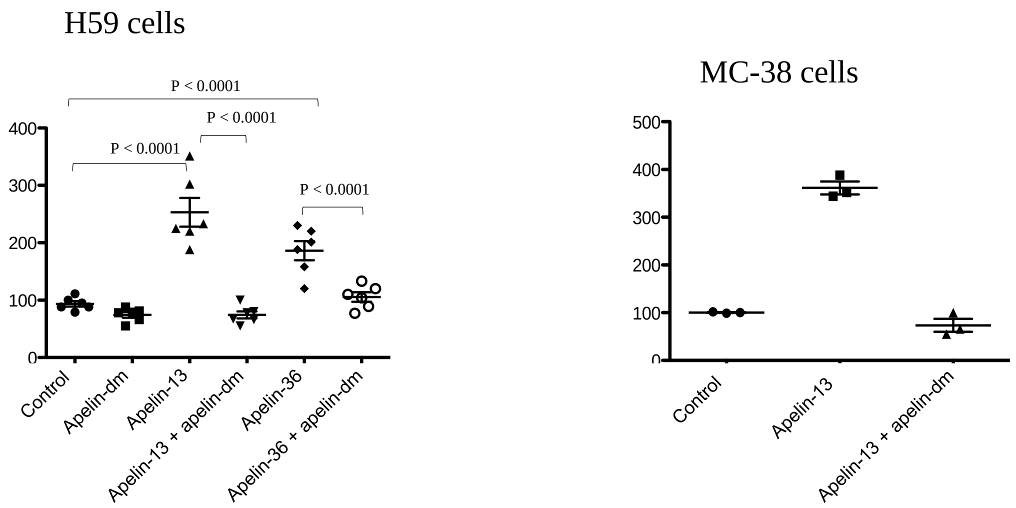

Appendix Figure S6. Effect of apelin-dm on cell migration. Boyden chamber assays were done on H59 ad MC38 cells to analyze the effect of apelin-dm on basal and apelin peptides-induced migration. Three independents experiments were done (n = 3) and the results are represented as the percentage of the migrating cells. The data are representative of three independent experiments. n.s.=not significant. All values represent the mean±s.e.m. Significant differences P were determined by Two-way ANOVA.

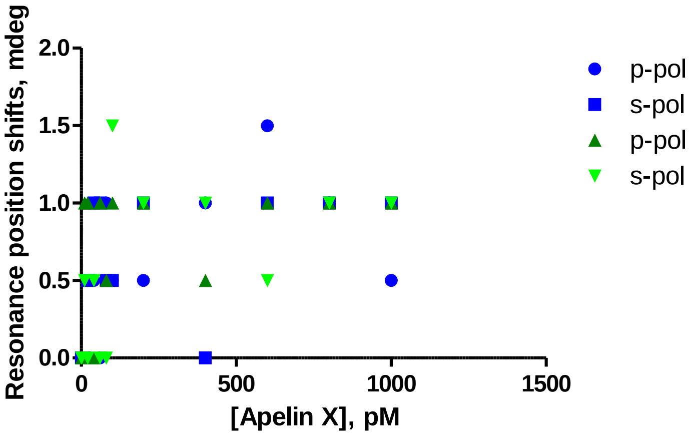

Appendix Figure S7. Non-specific binding of apelin ligands to cell membranes. PWR spectral changes obtained upon addition of incremental apelin ligand concentration (apelin DM in blue and apelin 13 in green) to cell membrane fragments that do not overexpress the apelin receptor and that are captured in the PWR sensor.

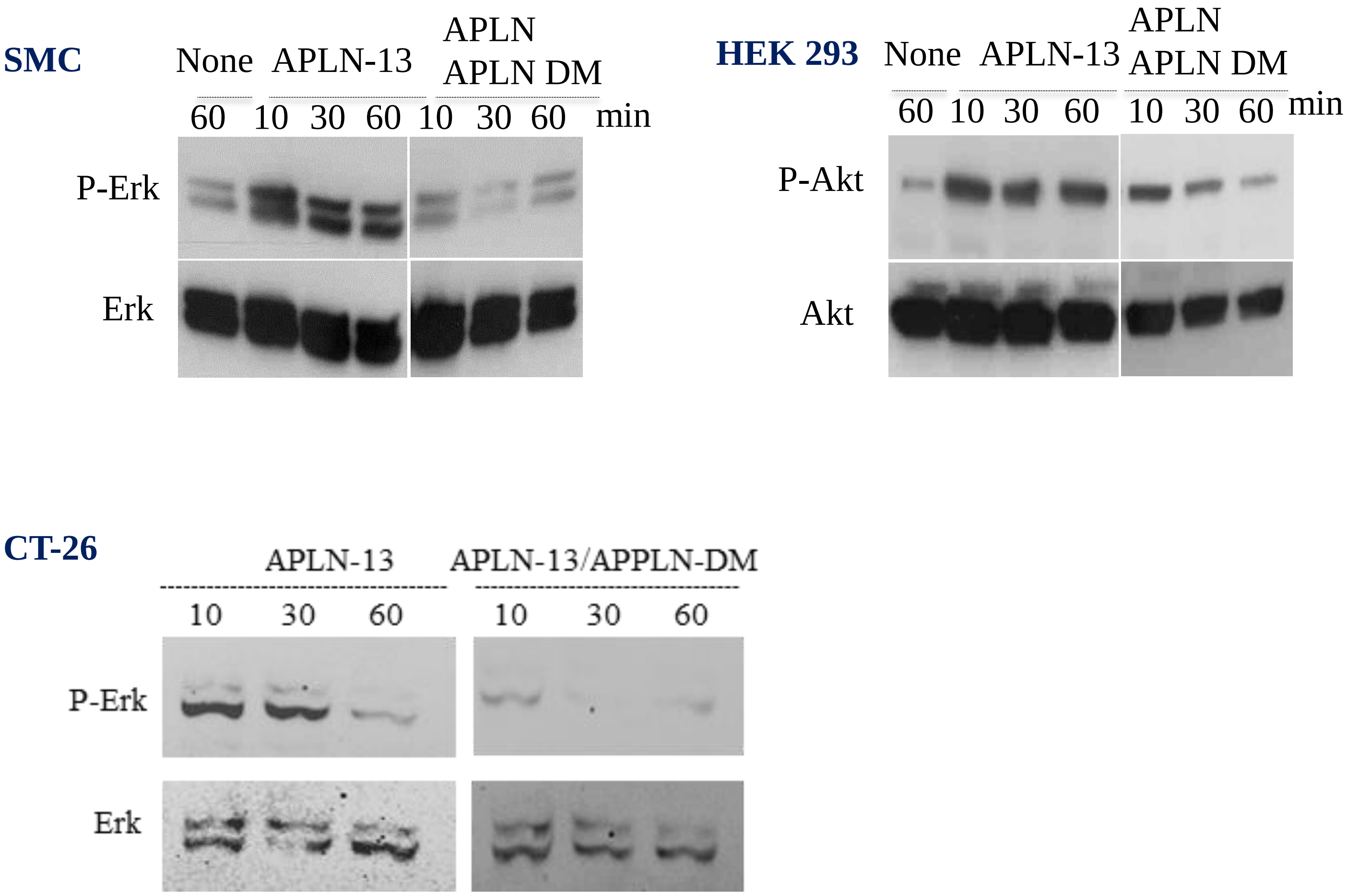

Appendix Figure S8. Phosphorylation of Erk and Akt in SMC, HEK 293 and CT-26 cells treated with APLN and/or APLN DM.

Assay Type	PTK		STK	
Comparisons	Up	Down	Up	Down
APLN vs Control[a]	28	22	18	40
APLN-DM vs Control[a]	7	8	10	27
APLN-DM vs APLN	5	17	3	4

[a]Significance was obtained using a one-way ANOVA followed by a post-hoc Dunnett's test, p<0.05; [b]Significance was obtained using a two-sided unpaired Student's T-test, p<0.05.

Assay Type	PTK		STK	
Comparisons	Up	Down	Up	Down
APLN vs Control	InSR, Lyn, Lck, Src, IRR, HCK, BLK, Fyn, TEC	FLT1	ERK2	PKA[alpha], PKG2, PKG1, p70S6K[beta], PRKX, PKC[alpha], SGK2, CaMK4, AMPK[alpha]1
APLN-DM vs Control	Lyn, InSR, RYK, Lck, HER3, HER2, BLK, KDR	TXK, JAK1~b	CDK9	PKA[alpha], PKG2, PKG1, CaMK4, PRKX, p70S6K[beta], PKC[alpha], CHK2, AMPK[alpha]1
APLN-DM vs APLN	FLT4, FLT1	Lyn, InSR, Src, Syk, Lck, Etk/BMX, Ron, Fgr	PKG1, PKC[alpha], PKA[alpha], PKG2, IKK[alpha], SGK2, PKC[delta], PRKX	DYRK1A, CHK1

Appendix Figure S9. The top 10 kinases above the default threshold (Kinase Score > 1.3) are shown

Kinase Score Plot (family)

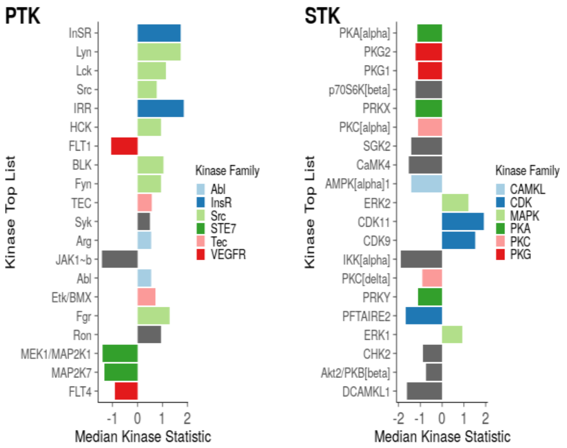

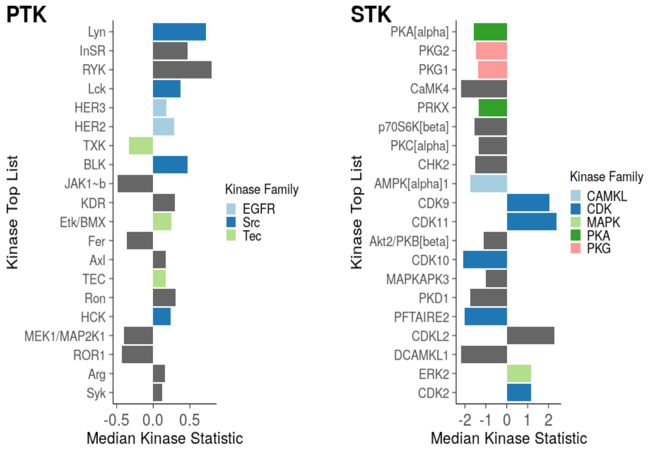

APLN vs Control **APLN DM vs Control**

Kinase Score Plot (specificity)

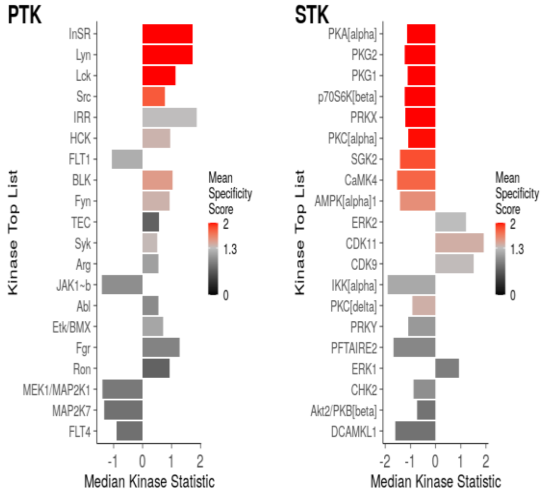

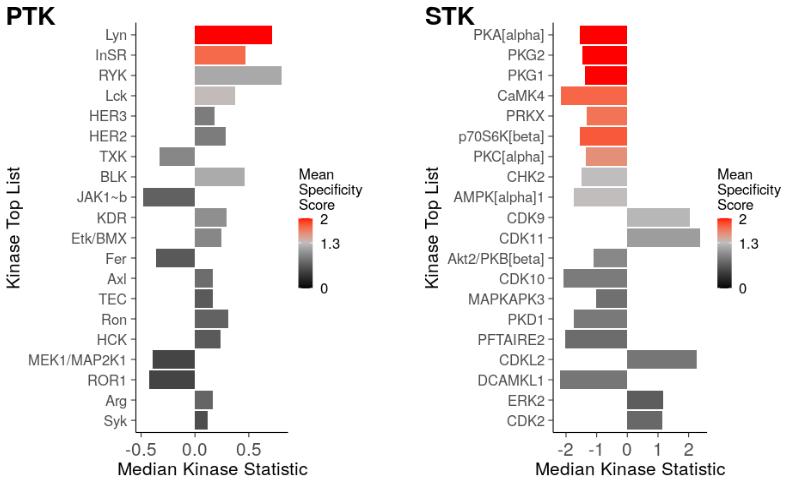

APLN vs Control **APLN DM vs Control**

Appendix Figure S10. Upstream kinase analysis of PTK and STK of APLN and APLN DM-treated cells showing the top 20 ranked kinases (normalized kinase statistic (log2) < 0: less kinase activity in treated cells; specificity score (log2) >1.3; white to red bars: statistically significant changes).
Kinase Score Plot that identify the effect size and direction of the top 20 kinases. Plots are colored by specificity score or kinase family. The Score Plots show the top 20 kinases, regardless of the Kinase Scores.

Kinase Score Plot (family)

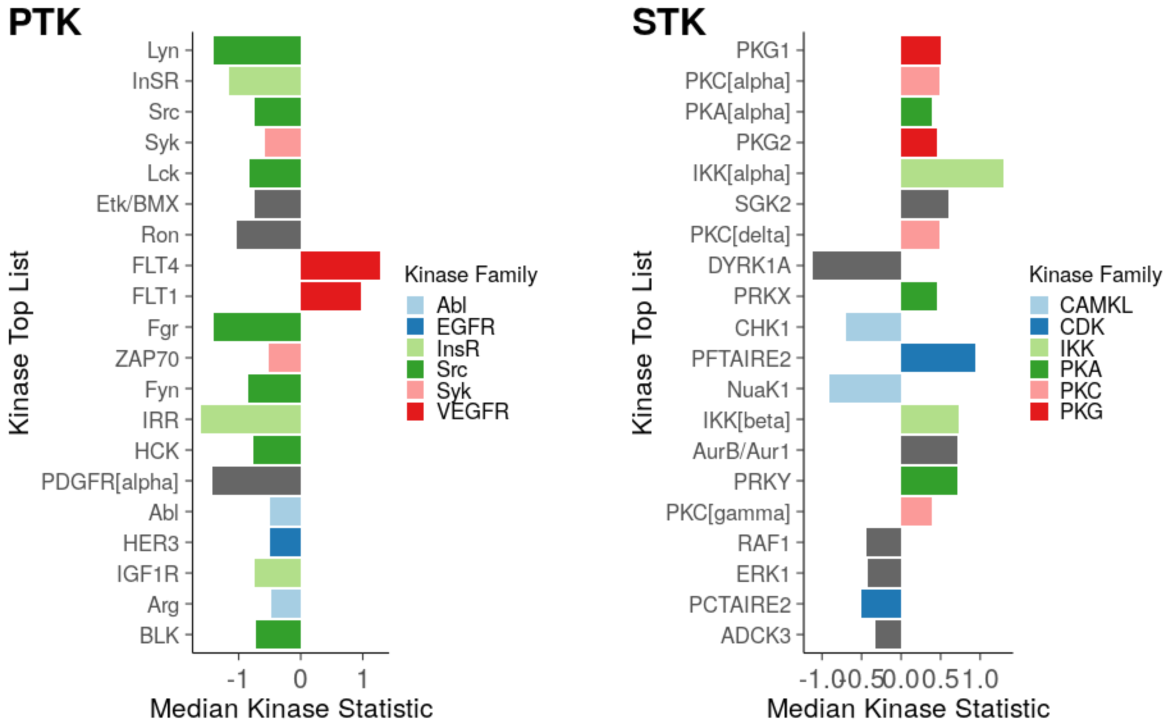

APLN DM vs APLN

Kinase Score Plot (specificity)

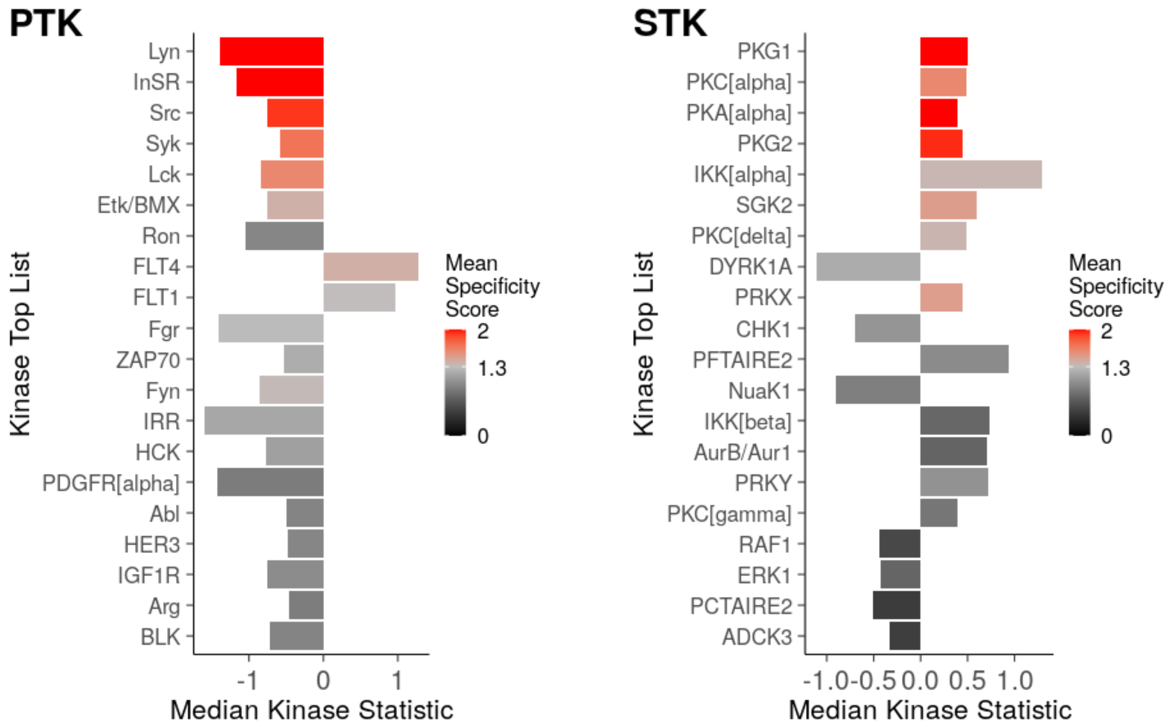

APLN DM vs APLN

Appendix Figure S11. APLN DM vs APLN top 20 ranked kinases (normalized kinase statistic (log2) < 0: less kinase activity in treated cells; specificity score (log2) >1.3; white to red bars: statistically significant changes).

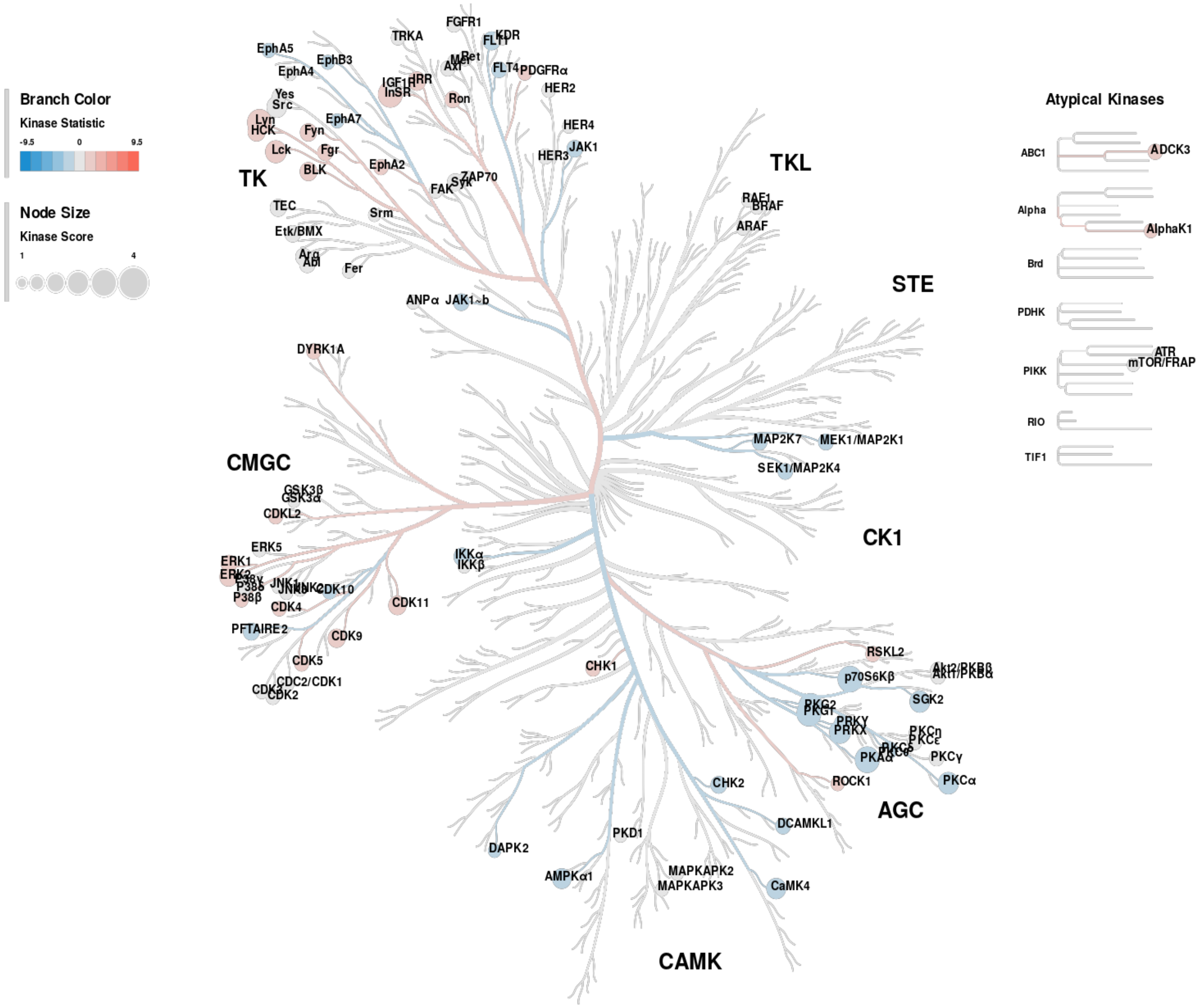

Apelin vs Control

Appendix Figure S12A, Kinome Tree, groping the kinases in apelin treated HEK cells-expressing apelin receptor into phylogenetic families. The Coral Trees show all kinases above the default threshold (Kinase Score > 1.3). The Coral Trees are generated by: http://phanstiel-lab.med.unc.edu/CORAL/.

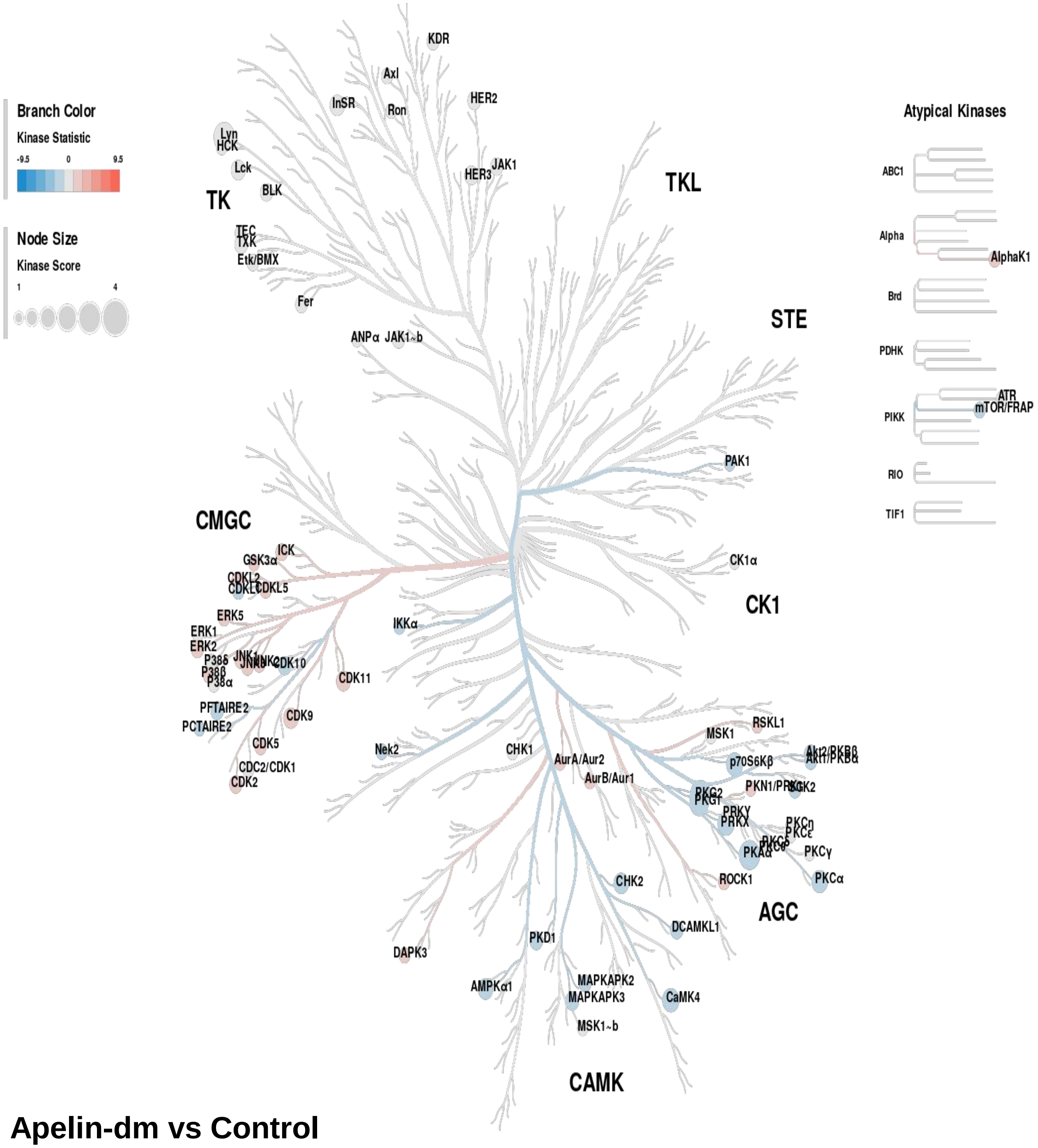

Apelin-dm vs Control

Appendix Figure S12B, Kinome Tree, groping the kinases in apelin treated HEK cells-expressing apelin receptor into phylogenetic families. The Coral Trees show all kinases above the default threshold (Kinase Score > 1.3). The Coral Trees are generated by: http://phanstiel-lab.med.unc.edu/CORAL/.

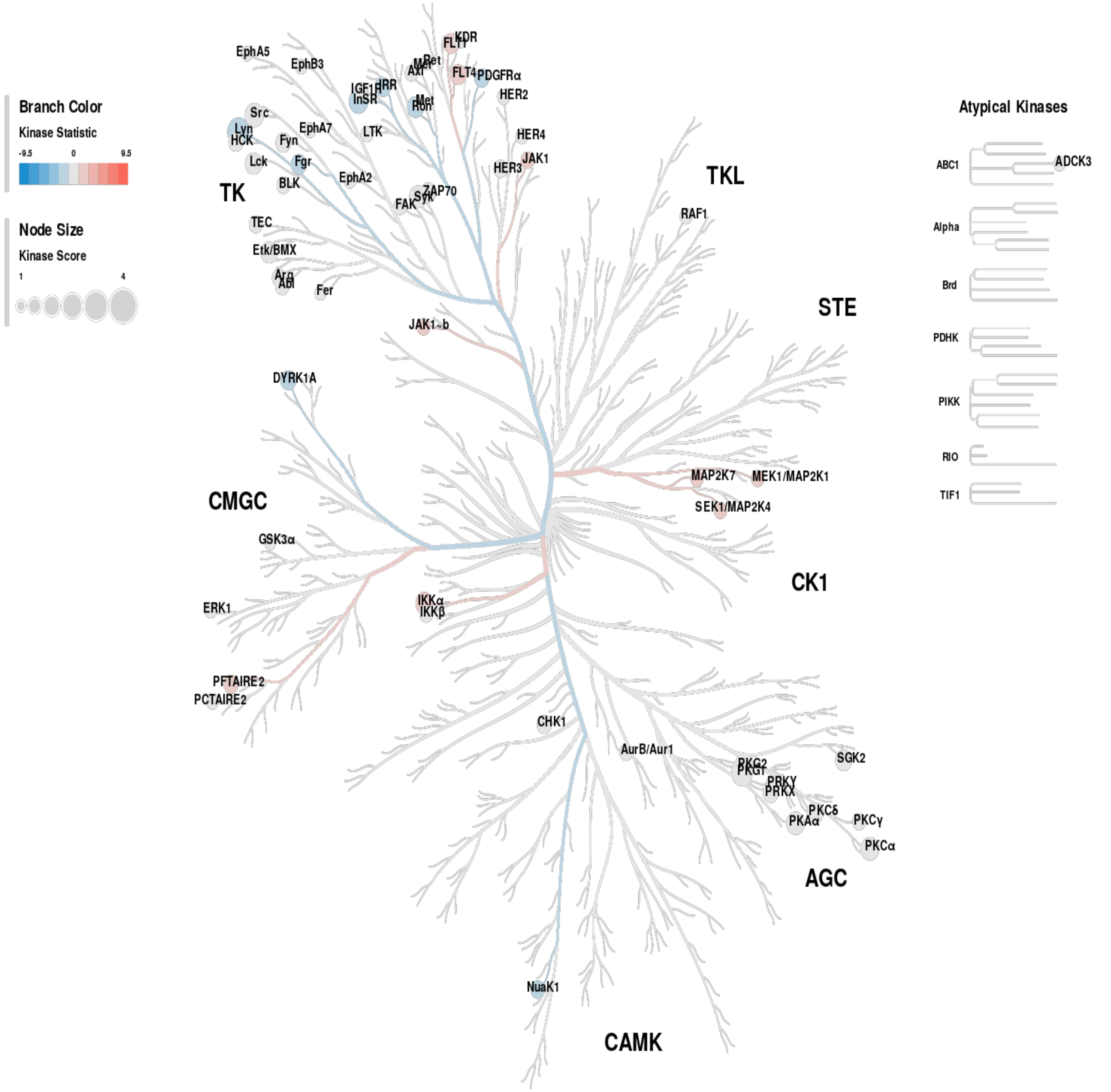

Apelin Vs Apelin-dm

Appendix Figure S12C, Kinome Tree, groping the kinases in apelin treated HEK cells-expressing apelin receptor into phylogenetic families. The Coral Trees show all kinases above the default threshold (Kinase Score > 1.3). The Coral Trees are generated by: http://phanstiel-lab.med.unc.edu/CORAL/.

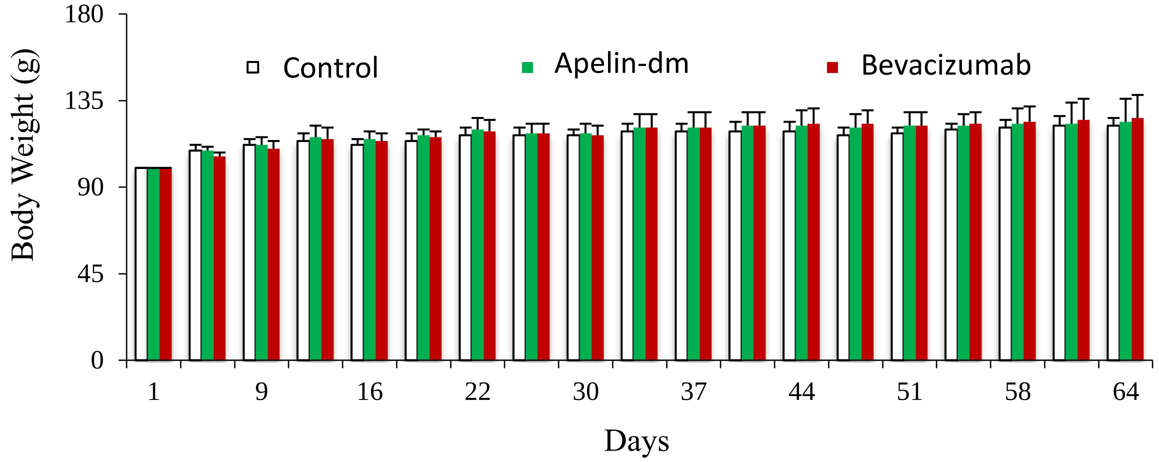

Appendix Figure S13. The body weights of the control, apelin-dm and Bevacizumab-treated mice groups.

Supplemental study 1
Maximal Tolerated Dose (MTD) study of APLN-DM in mice

Methods

Six groups comprising six Balb/C mice per group (3 males and 3 females) were assigned to the study. In each gender, one control group (1 Male (M)/1 female (F)) received the reference solution (vehicle) and 5 treated groups received APLN-DM peptide at dose levels of 200 mg/kg (2M/2F), 500 mg/kg (3M/3F), 1000 mg/kg (4M/4F), 2000 mg/kg (5M/5F) and 1500 mg/kg (6M/6F). APLN-DM peptide was administrated via intra-peritoneal (IP) single injection at day 1 followed by observation during 14 days. During the study, mortality, morbidity, general clinical signs and body weight were recorded. In the end of the study pathology and organ necropsy examination were performed. Thereby, APLN-DM peptide and vehicle were administered to mice according to **Table-1** and their effects were analyzed according to **Table-2**.

Each test item dose was administrated to 3 female and 3 male mice. The administration was performed IP at a dose volume of 10 ml/kg. Dosing was performed according to **Figure-1** and each increase in dose pending on the outcome of the previous dose (results of clinical signs). The lag time between the increasing doses was approximately 48h.

**Group No.**	***(n=)***	**Mouse#**	**Treatment**	**Dose Level**	**Route of administration**	**Volume administration**
1M<br>1F	*3+3*	4, 5, 6<br>24, 25, 26	Vehicle	-	IP	10ml/kg
2M<br>2F	*3+3*	1, 2, 3<br>21, 22, 23	APLN-DM	200 mg/kg	IP	
3M<br>3F	*3+3*	7, 8, 9<br>27, 28, 29		500 mg/kg	IP	
4M<br>4F	*3+3*	10, 11, 12<br>30, 31, 32		1000 mg/kg	IP	
5M<br>5F	*3+3*	13, 14, 15<br>33, 34, 35		2000 mg/kg	IP	
6M<br>6F	*3+3*	16, 17, 18<br>36, 37, 38		1500 mg/kg	IP	

Table-1. Study design-1. M = Male, M = Male

Study Day*	Procedure	Body Weight	Clinical Sign	Mortality & Morbidity	Organ observation
Acclimation	-	√	-	-	-
$1^{\#}$	IP Dosing	√	√	Once daily	-
2-14	-	Twice weekly	Once daily		-
15	Termination	√	√		Gross pathology for all animals

Table-2. Study design-2. $^{\#}$ = The animals were observed individually after 30min, 1h, 2h, and 4h post administration.

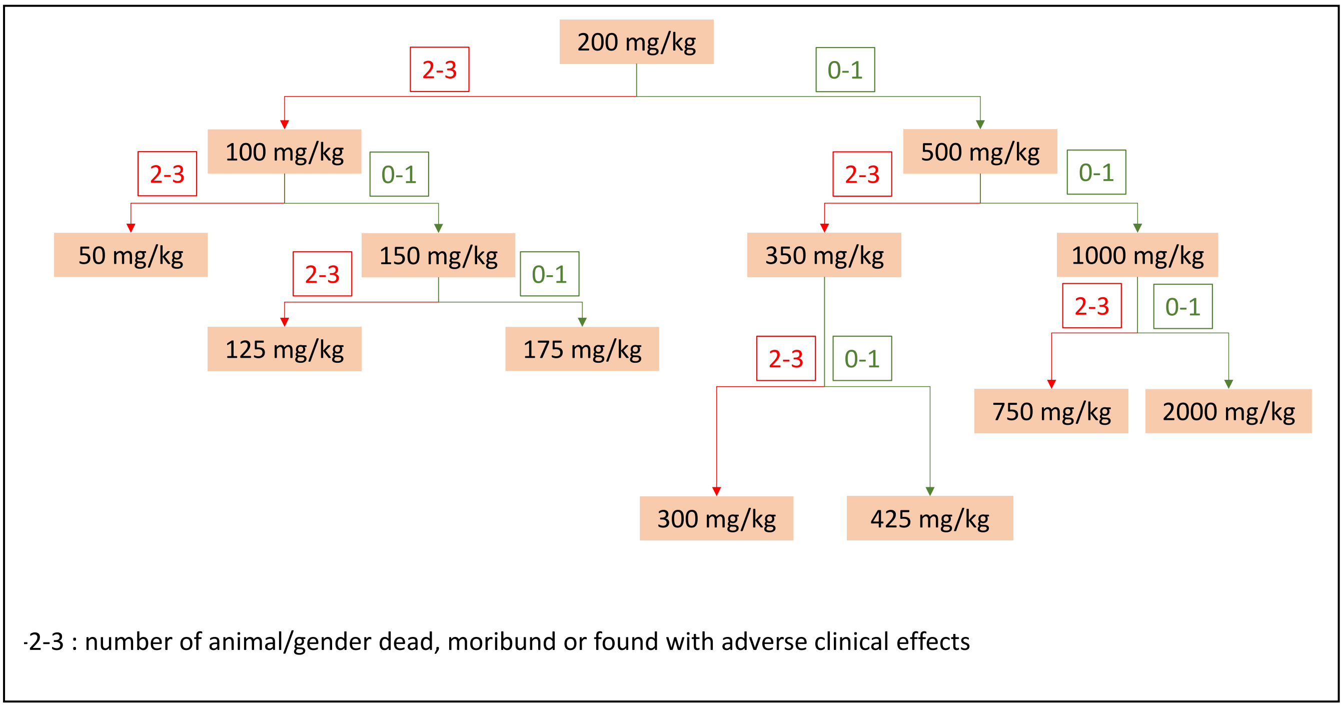

Figure 1 - Decisional tree for successive dosing for MTD determination

Results of the study

Morbidity and mortality were observed in all animals from groups 5M/5F treated with 2000 mg/kg, and in female mice from group 6M/6F treated with 1500 mg/kg. At study termination, body weight (Figure 2 and Figure 3) and gross pathology abnormalities were analyzed in all animals. Main pathological finding were blood clots in intestine and chest cavity. Treatment-related clinical adverse side effects (purple tail, lethargy, decreased motor activity) were observed also in groups treated either with 200, 500, 1000 and 1500 mg/kg. The tail recovered a normal color after 1 hour in all dose groups. The other clinical adverse side effects were reversed 4 hours after administration for the 200 and 500 mg/kg dose groups and the day after, or two days after the administration in the 1000 and 1500 mg/kg dose groups. No gross pathology abnormalities were detected in all male and female animals from these groups at the scheduled termination on day 15.

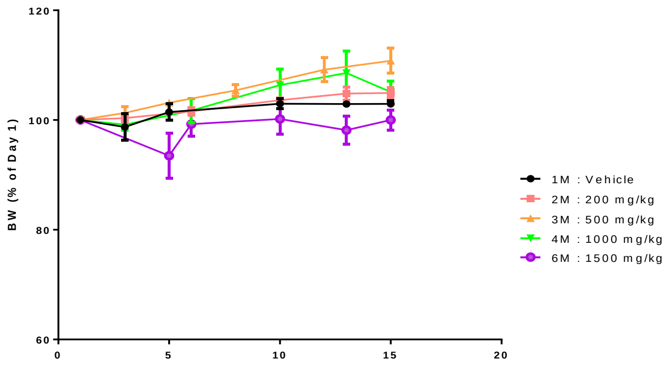

Figure 1- Group average body weight (BW) measurements (% of day 1) in males.

Group 5M is not represented in the graph because all mice died on Day 1.

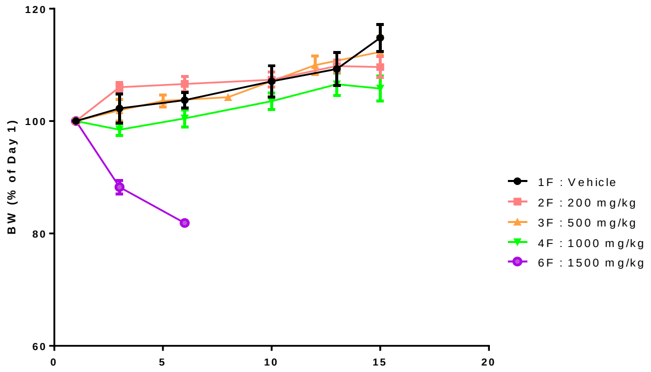

Figure 2- Group average BW measurements (% of day 1) in females

Group 5F is not represented in the graph because all mice died on Day 1.

Conclusion

Based on these findings, APLN-DM peptide acute MTD following IP administration in Balb/C mice was determined to be at 1500 mg/kg for males and 1000 mg/kg for females.

Supplemental study 2
Repeated dosing toxicity study of APLN-DM

The potential toxicity of APLN DM intraperitoneally injected into Balb/C mice once daily for 2 weeks, at doses of 50 mg/kg (groups named 2M+2F), 150 mg/kg (groups 3M+3F) and 300 mg/kg (groups 4M+4F), respectively, was assessed in the present study.

Methods: 4 groups (5 males and 5 females per group) were assigned for this study. In each gender, reference item control groups (1F+1M) received the reference item (Saline Solution, NaCl 0,9%) and 3 treated groups received APLN-DM at dose levels of 50 (low dose: 2F+2M), 150 (medium dose: 3F+3M) and 300 (high dose: 4F+4M) mg/kg. All animals were IP dosed and followed daily for 2 weeks (5 days a week). During the study, mortality and general clinical observation, body weight, food consumption, hematology, blood chemistry, gross pathology, selected organ necropsy and weight and histopathology examination of control and high dose groups were performed.

Results: One animal (#21) from group 2F died 15 minutes after the administration on day 10. This effect appeared to not be related to APLN-DM administration. Minor and reversible adverse side effects could be observed during 1 hour following test item administration. No treatment-related effects on food consumption and mean BW gain were observed during the study. There were no treatment-related significant changes for any clinical pathology parameters (hematology and blood chemistry) evaluated on study day 15. At terminal necropsies, we could not find any pathological signs in any tested groups. Mean absolute and Body weight (BW) relative organ weights in both genders were not affected by the treatment.

Conclusion: Under these study conditions APLN-DM was found to be safe in both genders Balb/C mice up to tested dose of 300 mg/kg/day administered for 14 days.

Methods

Total of 40 Balb/C mice (20 males and 20 females) were utilized and divided into 4 groups for each dosing of five female and five male mice **(Table 1 and Table 2)**. The choice of the number and the gender of the animals were based on OECD guidelines.

Study variables and end points

- Mortality and morbidity: daily
- Clinical sign observations: daily
- Body weight (BW) measurements: twice a week
- Food consumption: once a week
- Necropsy and gross pathology evaluation at study termination (animals from all groups)
- Terminal blood sampling for hematology and biochemistry analysis (animals from vehicle and high dose groups only)
- Selected organ weighing and fixation for histopathology analysis (animals from vehicle and high dose groups only)

○ **Humane End Points**

No animals were found in a moribund condition or with severe prolonged pain and enduring signs of severe prolonged distress.

○ **Duration of the Experimental Period**

Day of first item administration was considered "Day 1" and study termination "Day 15".

○ **Administration of the Test Items**

APLN-DM and vehicle were administered according to group design **(Table 1)**, study schedule **(Table 2)**. Each test item dose was administrated to 5 female and 5 males mice. The administration was performed IP at a dose volume of 10 ml/kg, 5 days a week for 2 weeks.

Table 1 - Study design

**Group No.**	***(n=)***	**Treatment**	**Dose Level**	**ROA**	**Volume administration**	**Individual animal #**
1M+1F	*5+5*	Vehicle	-	IP	10ml/kg	M 16, 17, 18, 19, 20<br>F 36, 37, 38, 39, 40
2M+2F	*5+5*	Test Item (APLN-DM)	50 mg/kg	IP		M 1, 2, 3, 4, 5<br>F 21, 22, 23, 24, 25
3M+3F	*5+5*		150 mg/kg	IP		M 6, 7, 8, 9, 10<br>F 26, 27, 28, 29, 30
4M+4F	*5+5*		300 mg/kg	IP		M 11, 12, 13, 14, 15<br>F 31, 32, 33, 34, 35

M = Male
F = Female

Study schedule

Table 2- Study schedule

Study Day*	Procedure	BW	Clinical Signs#	Food consumption	Mortality & Morbidity	Organ observation	Blood sampling	Selected Organ weighing and harvesting
Acclimation	-	√	-	√	-	-		
1-5, 8-12	IP Dosing	Before dosing	√	-	Once a day	-		
2-14	-	Twice a week	Once a day	Once a week		-		
15	Termination	√	√	-		Gross pathology for all animals	√ for hematology and biochemistry analysis (vehicle and high dose group only)	√ for histopathology analysis (vehicle and high dose group only)

# = The animals were observed individually: after 30min, 1h, 2h, and 3h post administration.

Tests and Evaluations

Morbidity and Mortality Observation

Observations for signs of morbidity and mortality were performed daily. Moribund animals or animals obviously in pain, showing signs of severe and enduring distress, and animals showing a decrease of body weight larger than 20% from initial body weight determination were euthanized. The time of death was recorded as precisely as possible. The animals, which were humanely killed during the test, were considered for the interpretation of test results in the same way as animals that die on test.

Clinical Observations

The animals were observed during 3 hours following each administration. Thereafter, the animals were observed once daily until study termination on day 15, for a total of 14 days, except where they needed to be removed from the study and humanely killed for animal welfare reasons or were found dead. The duration of observation was not fixed rigidly. It was determined by the toxic reactions and time of onset and length of recovery period, and may thus be extended when considered necessary. The times at which signs of toxicity appeared and disappeared are important especially if there was a tendency for toxic signs to be delayed. All observations were systematically recorded and individual records were maintained for each animal.

Additional observations were necessary if the animals continued to display signs of toxicity. Observations included changes in skin and fur, eyes and mucous membranes, and also respiratory, circulatory, autonomic and central nervous systems, and somatomotor activity and behavior pattern. Attention was directed to observations of tremors, convulsions, salivation, diarrhea, lethargy, sleep and coma. The principles and criteria summarized in the Humane Endpoints Guidance Document were taken into consideration. Animals found in moribund condition and animals showing severe pain or enduring signs of severe distress were humanely killed. When animals were killed for humane reasons or found dead, the time of death was recorded as precisely as possible.

Body Weight (BW)

BW was measured after animal arrival, prior to each administration to adjust the dose and twice weekly at fixed hours during the study. Individual body weight changes were calculated.

Food Consumption: Food consumption for a 24 hours period was performed once during acclimation and thereafter once a week until study termination on day 15 (total of 3 measurements). Determinations of food consumption (calculated value) were based on a provided diet placed in hoppers and the remaining unused diet per group of 5 animals per cage.

Study Termination: On termination day (day 15), animals were euthanized and gross pathology was performed evaluating the major tissue and organ systems. All animals (including those which died during the test or were removed from the study for animal welfare reasons) were subjected to gross necropsy. All gross lesions in organs and major tissue were documented. Selected organs (colon, intestine, gonads, pancreas, liver, kidneys, spleen, lungs and heart) from 3 males and 3 females from control and high dose groups were isolated, weighed and conserved in buffered 4% formaldehyde for histological evaluation. Paraffin blocks were produced and sections were stained with Hematoxilin-Eosin-Saffron. This evaluation was done by Oncovet Clinical Research (see underneath).

Blood samples were taken from orbital sinus on day 15 after isoflurane anesthesia for control and high dose groups. The samples were analyzed as follow:

- Sample 1 for Hematology (Table 3): Blood samples were collected into EDTA tubes and directly used for hematology analysis.

Table 3 - Hematology analysis parameters

Sl. No.	Parameter	Abbreviations
1	Haematocrit	HCT
2	Haemoglobin	HGB
3	Mean Corpuscular Haemoglobin	MCH
4	Mean Corpuscular Haemoglobin Concentration	MCHC
5	Mean Corpuscular Volume	MCV

6	Platelets	PLT
7	Red Blood Corpuscles	RBC
8	White Blood Corpuscles	WBC
9	Differential leukocyte count (Monocyte, Lymphocyte, Neutrophil, Eosinophil, Basophil)	DLC (Mono, Lymph, Neut, Eo, Baso)

- Sample 2 for Clinical chemistry (Table 4): Blood samples were collected into Lithium Heparinate tubes and directly used for clinical chemistry analysis.

Table 4 - Clinical chemistry analysis parameters

Sl. No.	Parameter	Abbreviations
1	Alkaline Phosphatase	PAL
2	Alanine aminotransferase	ALT
3	Albumin	ALB
4	Blood Urea Nitrogen	BUN
5	Creatinine	Creat
6	Glucose	Glu
7	Total Plasma Protein	T.Pro

RESULTS

Clinical sign observation

No adverse clinical signs were observed in the animals of groups 2M/2F (50 mg/kg) during the study period. Adverse clinical signs (purple tail, decreased motor activity) were observed in all mice of groups 3M/3F (150 mg/kg) during the first hour after the first and the second administration of APLN-DM (Days 1 and 2). The same adverse clinical signs occurred in all mice of groups 4M/4F (300 mg/kg) during the first hour after the first and the third administration (Days 1 and 3). These effects disappeared 1 hour after the administration and were not observed from Day 4 in all treated groups.

Body weight (BW)

For each group, absolute BW (g) and BW percentage of day 1 were compared to the reference item group 1M for males and 1F for females until study day 14 using One-Way ANOVA followed by Dunnett's post-hoc test. BW gain compared to day 1 was comparable and steady in test-item treated male groups **(**.

Group 5M is not represented in the graph because all mice died on Day 1.

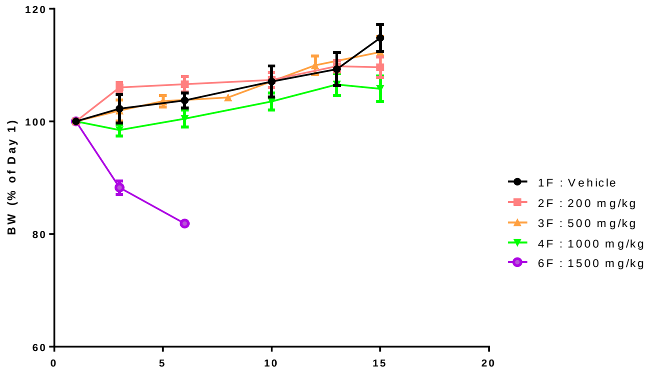

Figure 2) and female (.

Group 5M is not represented in the graph because all mice died on Day 1.

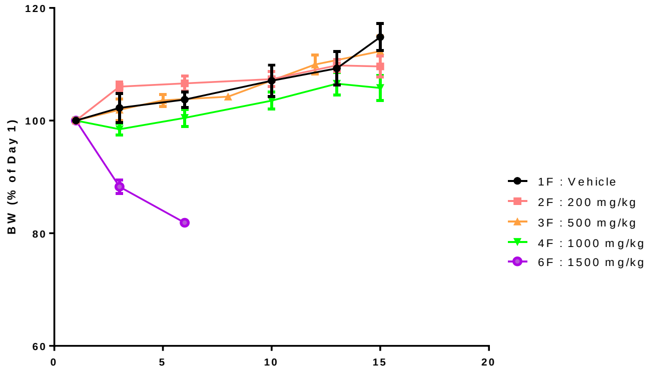

Figure 2) mice compared to the matched vehicle group until study day 14.

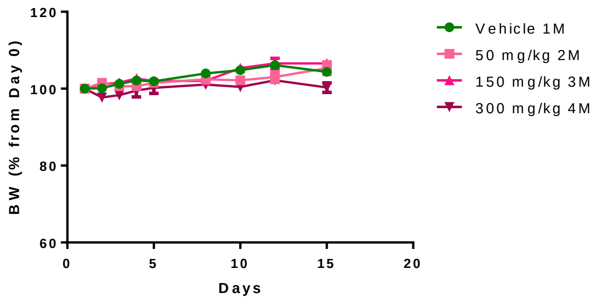

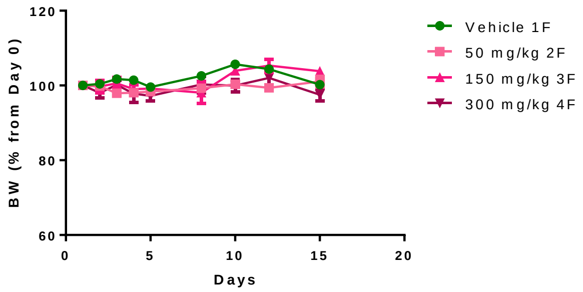

Figure 1- Group average BW measurements (% of day 1) in males

Figure 2- Group average BW measurements (% of day 1) in females

Food Consumption (FC)

Food consumption was comparable and steady in all test-item treated groups in both males and females compared to the matched vehicle group and age of animals at all-time points tested.

Organ weights

Organ weights of groups 4M/4F (300 mg/kg) were compared to the vehicle group (1M/1F) using One-Way ANOVA followed by Dunnett’s post-hoc test. No test-item related changes in organ body weight were observed in the mean absolute organ weights in both female and male animals. The group average weight of

the selected organs (g) of the male mice were calculated and presented in **Table 5**. The group average weight of selected organs (g) of the female mice were calculated and presented in **Table-6**.

Table 5- Group average of selected organ weight (g) from male mice

**Group**		**Vehicle (1M)**			**300mg/kg m-Apelin-36 (4M)**		
**Animal #**		**16, 17, 18, 19, 20**			**11, 12, 13, 14, 15**		
**Organs**		Average	SD	N	Average	SD	N
**Heart**		0.1478	0.0345	5	0.1622	0.0455	5
**Lung**		0.1504	0.0091	5	0.1548	0.0209	5
**Pancreas**		0.1576	0.0082	5	0.1888	0.0357	5
**Spleen**		0.1162	0.0243	5	0.1062	0.0125	5
**Liver**		1.3494	0.0813	5	1.6730	0.1479	5
**Kidney**	R	0.2210	0.0168	5	0.2150	0.0242	5
	L	0.2350	0.0157	5	0.2266	0.0248	5
**Testis**	R	0.0978	0.0183	5	0.1058	0.0073	5
	L	0.0954	0.0197	5	0.1084	0.0135	5

Table 6- Group average of selected organ weight (g) from female mice

**Group**		**Vehicle (1F)**			**300mg/kg m-Apelin-36 (4F)**		
**Animal #**		**36, 37, 38, 39, 40**			**31, 32, 33, 34, 35**		
**Organs**		Average	SD	N	Average	SD	N
**Heart**		0.1154	0.0148	5	0.1076	0.0063	5
**Lung**		0.1576	0.0830	5	0.1342	0.0080	5
**Pancreas**		0.1294	0.0423	5	0.1542	0.0423	5
**Spleen**		0.1178	0.0091	5	0.1145	0.0092	5
**Liver**		0.9236	0.0673	5	1.0914	0.0391	5
**Kidney**	R	0.1268	0.0121	5	0.1358	0.0058	5
	L	0.1344	0.0105	5	0.1332	0.0067	5
**Ovary**	R	0.0136	0.0032	5	0.0150	0.0046	5
	L	0.0174	0.0054	5	0.0148	0.0036	5

Blood Hematology analysis

Hematology results of groups 4M/4F (300 mg/kg) were compared to the vehicle group (1M/1F) using One-Way ANOVA followed by Dunnett's post-hoc test and to the normal range. No test-item related or significant effects on any tested parameter could be observed in both male and female mice.

Summary of male group average hematology results are presented in **Table 7** and summary of female group average hematology results are presented in **Table 8**.

Table 7- Summary of group average blood hematology results at day 15 in male groups

**Groups**		**Vehicle (1M)**			**300mg/kg m-Apelin-36 (4M)**		
**Animal #**		**16, 17, 18, 19, 20**			**11, 12, 13, 14, 15**		
	**Units**	**Average**	**SD**	**N**	**Average**	**SD**	**N**
**RBC**	M/µL	9.87	0.48	5	10.04	0.32	5
**HGB**	g/dL	15.16	0.63	5	15.00	0.34	5
**HCT**	%	47.38	2.80	5	48.40	1.78	5

**MCV**	fl	47.98	0.75	5	47.98	0.38	5
**MCH**	pg	15.36	0.46	5	14.96	0.15	5
**MCHC**	g/dL	32.04	1.20	5	31.08	0.51	5
**PLT**	K/µL	486.40	190.57	5	641.60	69.80	5
**WBC**	K/µL	6.10	2.45	3	7.28	1.10	4
**NEUT**	K/µl	1.67	0.73	3	1.42	0.13	4
**LYMPH**	K/µl	4.09	1.63	3	5.27	0.99	4
**MONO**	K/µl	0.12	0.06	3	0.12	0.03	4
**EO**	K/µl	0.20	0.07	3	0.29	0.08	4
**BASO**	K/µl	0.02	0.01	3	0.01	0.00	4
**NEUT**	%	27.00	3.18	3	19.78	2.22	4
**LYMPH**	%	67.20	2.19	3	72.18	4.80	4
**MONO**	%	2.17	1.01	3	1.55	0.26	4
**EO**	%	3.33	0.21	3	3.98	1.39	4
**BASO**	%	0.30	0.10	3	0.13	0.05	4

Table 8- Summary of group average blood hematology results at day 15 in female groups

**Groups**		**Vehicle (1F)**			**300mg/kg m-Apelin-36 (4F)**		
**Animal #**		**36, 37, 38, 39, 40**			**31, 32, 33, 34, 35**		
	**Units**	**Average**	**SD**	**N**	**Average**	**SD**	**N**
**RBC**	M/µL	9.59	0.20	5	9.25	0.58	5
**HGB**	g/dL	15.08	0.49	5	14.56	0.81	5
**HCT**	%	46.02	0.99	5	44.70	2.67	5
**MCV**	fl	48.00	0.58	5	47.74	0.55	5
**MCH**	pg	15.74	0.38	5	15.50	0.14	5
**MCHC**	g/dL	32.76	1.02	5	32.32	0.31	5
**PLT**	K/µL	543.80	123.96	5	508.60	55.41	5
**WBC**	K/µL	4.91	1.85	5	5.52	3.25	4
**NEUT**	K/µl	1.15	0.68	5	1.35	0.75	4
**LYMPH**	K/µl	3.56	1.24	5	3.67	2.23	4
**MONO**	K/µl	0.08	0.03	5	0.18	0.10	4
**EO**	K/µl	0.13	0.08	5	0.22	0.16	4
**BASO**	K/µl	0.01	0.01	5	0.02	0.01	4
**NEUT**	%	22.78	7.39	5	27.58	7.50	4
**LYMPH**	%	72.82	8.85	5	63.35	8.48	4
**MONO**	%	1.60	0.25	5	3.48	0.85	4
**EO**	%	2.64	1.52	5	3.78	1.09	4
**BASO**	%	0.16	0.26	5	0.63	0.59	4

Blood Clinical Chemistry analysis

Blood clinical chemistry results of groups 4M/4F were compared to the vehicle group (1M/1F) using One-Way ANOVA followed by Dunnett's post-hoc test and to the normal range. No test-item related changes in blood clinical chemistry could be observed in any tested group (both males and females). Summary of male group average blood clinical chemistry results are presented in **Table 9** and summary of female group average blood clinical chemistry results are presented in **Table 10**.

Table 9- Summary of group average blood chemistry results at day 15 in male groups

Groups		Vehicle (1M)			300mg/kg m-Apelin-36 (4M)		
Animal #		16, 17, 18, 19, 20			11, 12, 13, 14, 15		
	Units	Average	SD	N	Average	SD	N
ALB	g/L	24.60	0.89	5	24.20	0.84	5
PAL	U/L	158.00	30.14	5	178.80	17.95	5
ALT	U/L	91.20	58.29	5	88.20	48.26	5
BUN	mg/L	176.50	21.75	4	171.75	35.08	4
CREAT	mg/L	1.25	0.24	5	1.32	0.26	5
GLU	g/L	1.26	0.45	5	1.50	0.40	5
T Pro	g/L	49.40	2.41	5	49.40	2.41	5

Table 10- Summary of group average blood chemistry results at day 15 in female groups

Groups		Vehicle (1F)			300mg/kg m-Apelin-36 (4F)		
Animal #		36, 37, 38, 39, 40			31, 32, 33, 34, 35		
	Units	Average	SD	N	Average	SD	N
ALB	g/L	23.40	1.14	5	26.40	0.89	5
PAL	U/L	192.80	22.19	5	200.20	13.03	5
ALT	U/L	39.20	1.92	5	53.80	23.76	5
BUN	mg/L	189.30	52.54	5	185.12	59.82	5
CREAT	mg/L	1.54	0.32	5	1.40	0.14	5
GLU	g/L	1.23	0.30	5	1.46	0.34	5
T Pro	g/L	47.20	2.59	5	51.40	1.14	5

Histopathology evaluation

Histopathology results and evaluation were performed by Oncovet Clinical Research (Loos, France), Study phase report is presented in the end of this document.
Only minimal lesions were observed, sporadically and without any group related effect.
The main lesion observed was diffuse renal lipidosis. These lesions were observed in all groups including vehicle group and were minimal to discrete. We could also observe some hepatic diffuse, discrete extra medullary hematopoiesis in a few animals and finally some splenic moderate extra medullary hematopoiesis. All of these lesions are typical background lesions which are found in a physiological state in mice. Some congestive foci have been observed in lung. These were always focal extensive, acute, minimal to discrete. These lesion were of poor intensity, not related to any group and are considered as very common background lesions. They are mainly thought to be related to handling. A peracute hemorrage has also been noticed in one animal (4M-12), no inflammatory cells or fibrin was observed in association and is most likely artefactual, related to handling. Alimentary tract has shown a normal development without any inflammatory infiltrate. Intestinal villi, crypts, enterocytes, chorion, submucosa, musculosa and serosa were all normal and no histological changes could be observed in any animal. Exocrine and endocrine pancreas were also histologically normal. All gonadic tissues displayed normal unaltered and complete maturation.

No inflammatory infiltration and no cytotoxic or other pathological changes were observed in all tested animals. The findings that were noted are not directly related to the study tested items and should be regarded as, irrelevant changes only.

Conclusions

The potential toxicity of APLN-DM was evaluated after repeated administrations (5 days a week for 2 weeks) in Balb/C mice for various parameters: BW gain, food consumption, clinical signs, clinical pathology (clinical chemistry and hematology), gross pathology organs weights and histopathology. Under study conditions, there were no treatment-related and/or toxicologically significant adverse side effects of APLN-DM for any of the parameters evaluated, in both genders Balb/C up to tested dose of 300 mg/kg/day.

REFERENCES

1. OECD principles of Good Laboratory Practice ENV/MC/CHEM (98) 17.
2. OECD 423: Guideline For Testing Of Chemicals - Acute Toxic Class Method (2001).

Supplemental study 3
Pharmacokinetic profile of APLN-DM in mice

Summary

The aim of the current study was to evaluate pharmacokinetic properties of APLN-DM peptide administrated intravenously (IV) or intraperitoneously (IP) in Balb/C mice.

Methods: Mice received 100, 30, 10 mg/kg IV, or 30 mg/kg IP APLN-DM peptide and blood sampling was performed 5, 10, 15, 30, 60 minutes or 1, 2, 4, 8, 24 hours following administration (5, 15, 30, 60 minutes for the IP group). Development of quantification method was performed using liquid chromatography coupled to mass spectrometry (LC/MS).

Results: Samples obtained *in vivo* were analyzed according to quantification-developed method and the peptide was detectable until 15 minutes after administration. Analysis allowed determining that APLN-DM peptide estimated half-life was about 20 minutes.

Study Design

All animals were divided to three groups of 27 animals, one of 12 animals and one of 3 animals (vehicle) and will receive the corresponding test item according to **Table 1**. Each animal was weighed and administered at the appropriate volume.

<table>
<tr><th>Group No. (n=96)</th><th>Treatment</th><th>Concentration</th><th>ROA</th><th>Dose Volume</th><th>Blood Collection</th><th>Individual animal numbers</th></tr>
<tr><td>1F (n=3)</td><td>Vehicle (NaCl 0.9%)</td><td>-</td><td rowspan="4">IV</td><td rowspan="4">According to BW, 5 ml/kg</td><td rowspan="4">5min, 10 min, 15min, 30min, 1h, 2h, 4h, 8h, 24h</td><td>13, 14, 15</td></tr>
<tr><td>2F (n=27)</td><td>APLN-DM (100 mg/kg)</td><td>20 mg/ml</td><td>1st test: 85, 86, 87, 88, 89, 90, 91, 92, 93, 94, 95, 96, 97, 98, 99, 82, 83, 84, 79, 80, 81, 73, 74, 75, 70, 71, 72
2nd test: 126, 127, 128, 129, 130, 131, 132, 133, 134, 135, 136, 137, 138, 139, 140, 123, 124, 125, 120, 121, 122, 141, 142, 117, 118, 119</td></tr>
<tr><td>3F (n=27)</td><td>APLN-DM (30 mg/kg)</td><td>6 mg/ml</td><td>55, 56, 57, 58, 59, 60, 61, 62, 63, 64, 65, 66, 67, 68, 69, 52, 53, 54, 49, 50, 51, 46, 47, 48, 43, 44, 45</td></tr>
<tr><td>4F (n=27)</td><td>APLN-DM (10 mg/kg)</td><td>2 mg/ml</td><td>28, 29, 30, 31, 32, 33, 34, 35, 36, 37, 38, 39, 40, 41, 42, 25, 26, 27, 22, 23, 24, 76, 77, 78, 16, 17, 18</td></tr>
<tr><td>5F (n=12)</td><td>APLN-DM (30 mg/kg)</td><td>3 mg/ml</td><td>IP</td><td>According to BW, 10 ml/kg</td><td>5min, 15min, 30min, 1h</td><td>7, 8, 9, 10, 11, 12, 4, 5, 6, 1, 2, 3</td></tr>
</table>

Table 11 - Group allocation

Development of analytic method

Method-1
In a first step, the quantification method was developed and range of concentrations detectable and linearity were studied.

Chromatography
Several chromatographic columns were tested for APLN-DM in order to detect a symmetric peak of elution and as thinnest as possible, critical for the reliability of analytic method.
Thereby, a solution of APLN-DM was prepared (0.1556 mg in 397 µl water, and dilution 1/10). The chromatographic separation was made on a UPLC Acquity H-Class (Waters) with 4 different columns. The column and results of the assay are summarized in **Table 2**.

Column	RT (min)	Elution	Half-maximum width	A_s* (Peak symmetry factor)	Observation
BEH C18	1.55	52%	1.5s	1.09	2 peaks
Nucleoshell RP18	1.87	62%	18s	2.6	Too wide peak
PolarTec	2.56	85%	8s	4.7	Not acceptable
Kinetex F5	1.53	51%	2.1s	1.8	Acceptable

Table 12 - Chromatographic separation of APLN-DM and column selection. * A_s should be < 2

The selected column was Kinetex F5 2.3 µM 50x2.1 mm (Phenomenex).

Determination of mass spectrometry analysis conditions
The analysis was made with a LC-MS triple quadripole system (ESI QqQ). The chromatographic system was a UPLC activity (Waters) coupled to a mass spectrometer TSQ Quantum Ultra (Thermo) equipped with triple quadripole analyser and electrospray ionisation (ESI) system. The chromatographic conditions previously determined were used. A large mass spectrum was first registered for mass/charge (m/z) between 200 and 1200 Da in order to evaluate the charge states in these conditions. The spectrum obtained is presented in **Figure 1**.

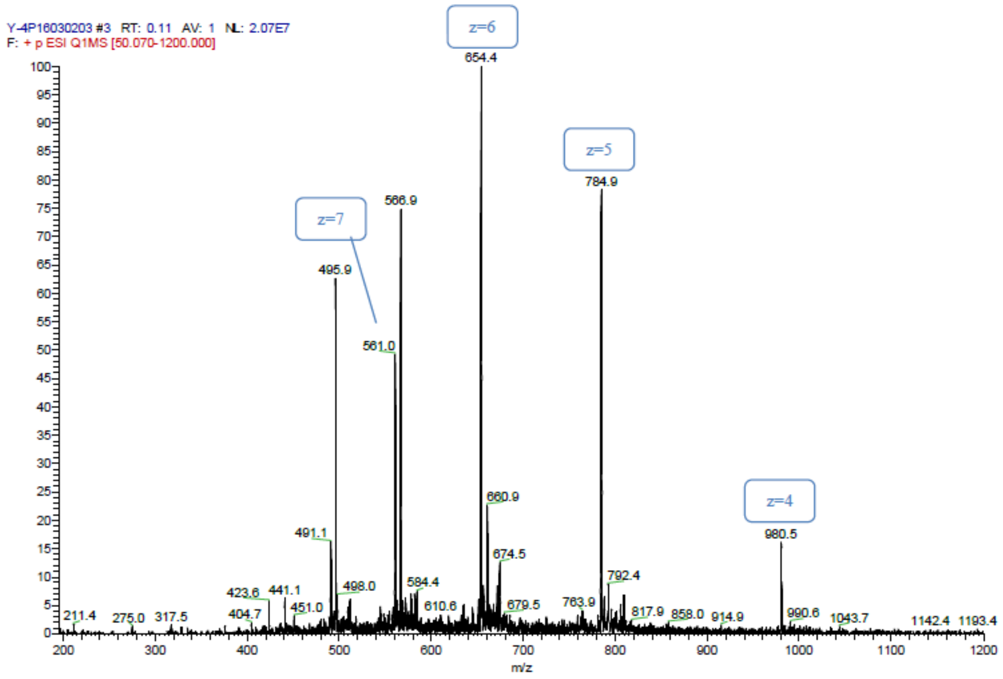

Figure 3 : Mass spectrum of APLN-DM in ESI-QqQ

This technique allowed us to determine 4 ions. The most abundant was ion 654.4 Da, corresponding to the species [M+6H]6+. This ion was selected for MS/MS analysis by collision-induced dissociation (CID) activation **(Figure 2).**

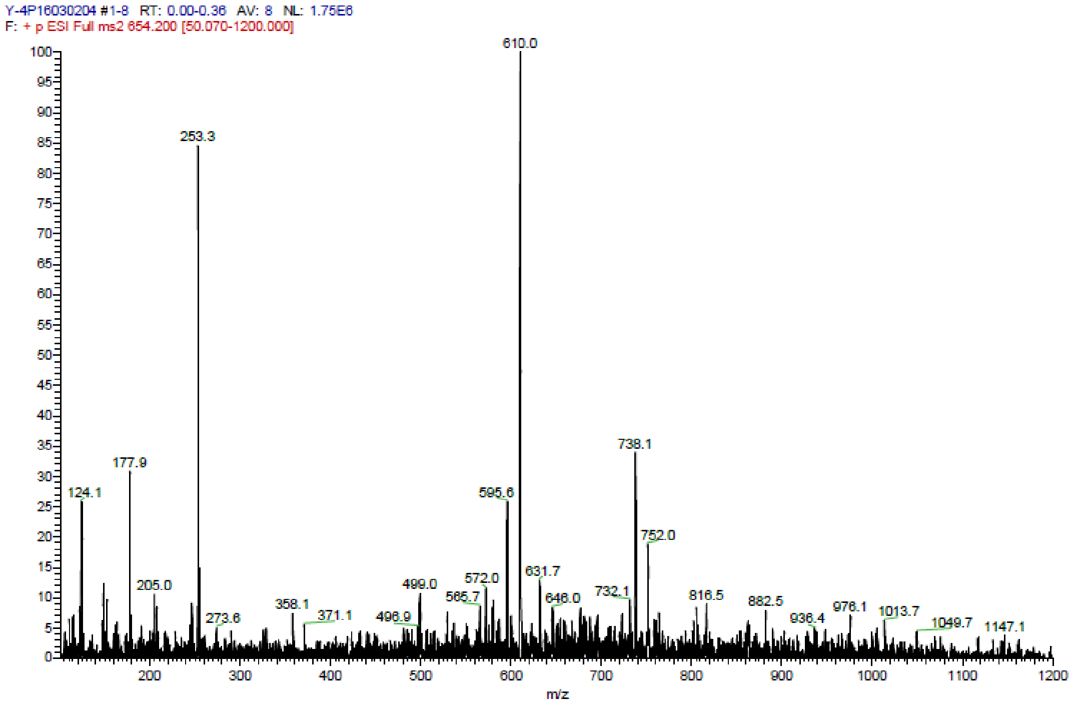

Spectre MS/MS de l'ion détecté à m/z 654.4 (z=6)

Figure 4 - MS/MS spectre of ion m/z 654.4 (z=6)

The most abundant fragmented ion was at 610.0 Da.
The following transition was chosen for the quantification monitoring of the compound:

654.4 → 610.0

E_{col} = 21eV

Determination of mass spectrometry analysis conditions
3 experiments were performed from 3 distinct APLN-DM solutions:
1) 0.1556 mg in 397 µl water, dilution 1/10 (1 x 10-5 M)
2) 0.0617 mg in 787 µl water (2 x 10-5 M)
3) 0.2090 mg in 533 µl water (1 x 10-4 M)
The 3 experiments gave the same results and the second is represented in the following part.
Lower limits of detection and quantification
The lower limit of detection (LOD) is defined as the signal with a ratio signal/background noise > 3.
The lower limit of detection (LOD) is defined as the signal with a ratio signal/background noise > 10.
The obtained values were:
- LOD = 2.5 x 10-7 M
- LOQ = 5 x 10-7 M

Method linearity
A standard range was prepared with triplicate injection of solutions between 0.5 and 100 µM. **(Table 3, Figure-1).**

Concentration (µM)	Area	Mean	SD
100	7838235	7907569	0.89
	7905614		
	7978858		
50	5860743	5703475	3.98
	5806614		
	5443069		
10	1441526	1474761	2.87
	1460305		
	1522452		
5	327679	351884.3	7.05
	350714		
	377260		
1	12769	12392.33	10.86
	13510		
	10898		
0.5	1652	1827.333	14.54
	2133		
	1697		

Table 13 - Quantification results of standard range

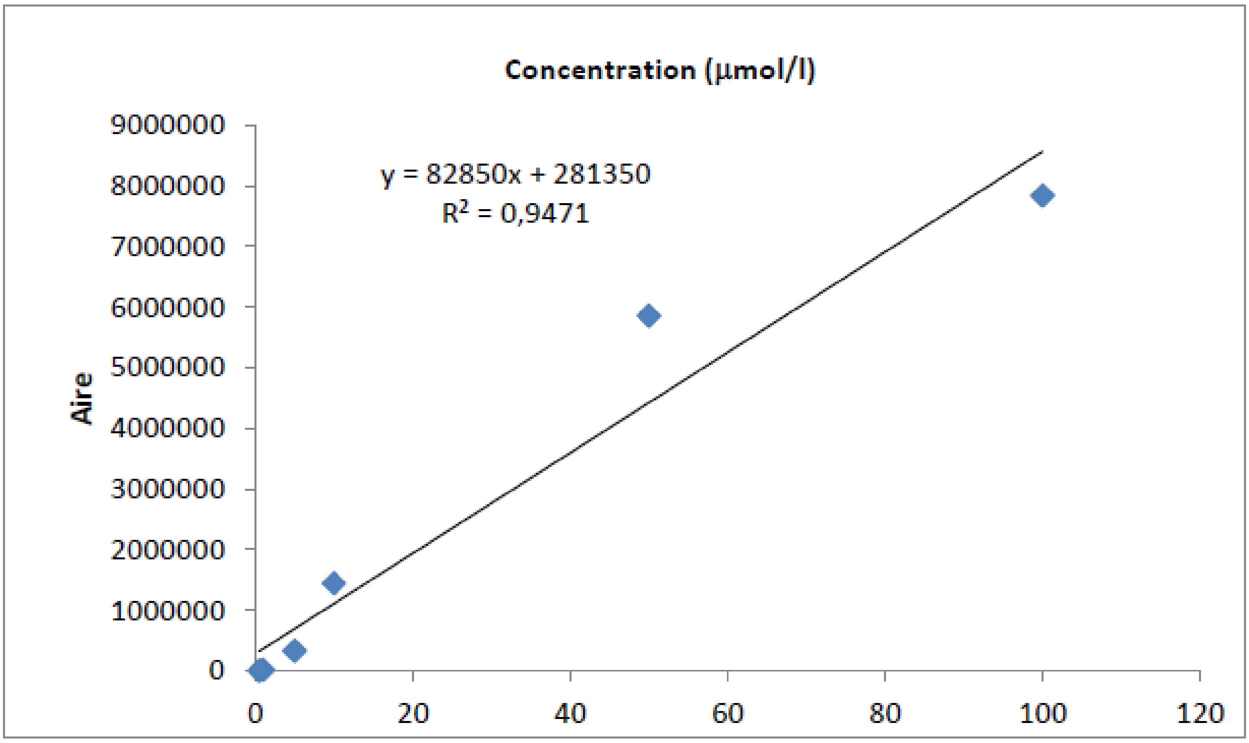

Figure 5: Standard curve representation

The standard deviations were below the acceptable values (<15%) showing that the accuracy is correct and compliant with standard quantification protocols. However, the R^2 coefficient did not allow the validation of the linearity of the method. Hence, it was concluded that this method does not allow quantifying APLN-DM for concentrations below 0.5 µM.

Stability of APLN-DM

The second step of the study consisted to analyse the stability of the peptide in order to evaluate the feasibility of the quantification of APLN-DM in the samples obtained in vivo.

Quantification in the plasma after protein precipitation

Before the injection for LC-MS analysis, the plasmas had to be processed for elimination of major proteins, which can disturb the analysis. Proteins were precipitated by an organic solvent.

The first method consisted to prepare APLN-DM solution at 3.45 x 10-5 M (0.1352 mg in 345 µl water). 7 µl of this solution were added to 70 µl plasma or 70 µl water (control).

10 µl of each sample were added to 30 µl acetonitrile, vortexed and centrifuged for 15 minutes. The supernatant was evaporated and the pellet was suspended in 30 µl water. The 2 samples (peptide in plasma or in water) were injected in LC-MS according to the conditions previously established. Results obtained are presented in **Table 4.**

Sample	RT (min)	Total area	Relative area
Water	1.10	232525	100%
Plasma	1.10	28323	12.1%

Table 14 - Quantification of APLN-DM by LC-MS (MRM) after protein precipitation according to the first method.

With this method, only 12.1% of the APLN-DM initially added were recovered. Consequently, this method was not applicable for the further study.

Method-2

The second method was adapted from the protocol described by O. Chertov et al. (Proteomics 2004, 4, 1195-1203). A solution at 10.5 x 10-5 M was prepared (0.2976 mg in 759 µl water). 7 µl of this solution were added to 70 µl plasma or 70 µl water (control).

10 µl of each sample were mixed to 20 µl water, vortexed, mixed to 45 µl d'acetonitrile + 0.1% trifluoroacetic acid, sonicated, vortexed, centrifuged for 15 minutes. The supernatant was evaporated and the pellet was re-suspended in 30 µl water. The 2 samples (peptide in plasma or in water) were injected in LC-MS according to the conditions previously established. Results obtained are presented in Table 5.

Sample	RT (min)	Total area	Relative area
Water	1.10	418957	100%
Plasma	1.10	378860	90.5%

Table 15 - Quantification of APLN-DM by LC-MS (MRM) after protein precipitation according to the second method

With this method, the recovery of APLN-DM in the plasma after protein precipitation was sufficient. The reproducibility of this method on 5 samples prepared in the same manner is presented in Table 6.

Sample	Area
1	3289947

2	3567715
3	3436496
4	3396997
5	3422064
Mean	3422644
Coefficient of variation	2.9%

Table 16 - Quantification of 5 samples of APLN-DM in plasma after protein precipitation

The coefficient of variation was acceptable. This method was used for the stability study in the plasma.

Stability of APLN-DM in water and in plasma

A solution at 10.5 x 10-5 M was prepared (0.2582 mg in 659 µl water). 7 µl were added to 3 samples of 70 µl plasma or 70 µl water.

Six solutions were obtained:

- W RT : solution in water stored at room temperature
- W 4°C : solution in water stored at 4°C
- W -18°C : solution in water stored at -18°C
- P RT : solution in plasma stored at room temperature
- P 4°C : solution in plasma stored at 4°C
- P -18°C : solution in plasma stored at -18°C.

At each time (0, 1h, 2h, 5h, 24h, 48h, 72h, 144h, 168h), 10 µl of these solutions were collected and processed for protein extraction (according to the method previously described), and then injected in LC-MS for APLN-DM quantification. Results obtained are presented in Table 7 and figures 4 et 5. For each sample, % were calculated according to the area obtained at T=0 which correspond to 100%.

	**T (h)**	**0**	**1**	**2**	**5**	**24**	**48**	**72**	**144**	**168**
**Samples**	**W RT**	100	60	43	36	31	20	12	8	5
	**W 4°C**	100	71	66	53	40	38	31	20	10
	**W -18°C**	100	79	57	51	49	48	62	54	54
	**P RT**	100	21	17	13	8	14	7	1	22

	**P 4°C**	100	66	51	5	3	4	5	0	6
	**P -18°C**	100	75	66	114	86	97	89	32	12

Table 17 - Evaluation of APLN-DM time-dependent stability in water and in plasma

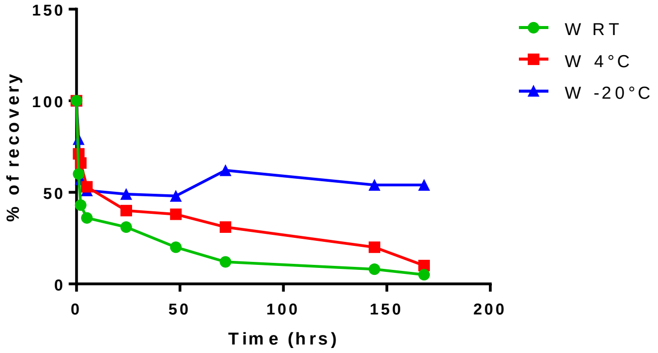

Figure 6: Stability of APLN-DM in water

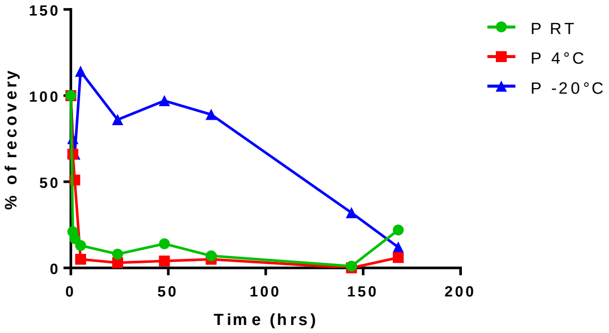

Figure 7: Stability of APLN-DM in plasma

These results showed fast degradation of the peptide in water even when the solution is stored at -18°C (less than 50% of APLN-DM is recovered after 5 hours in solution). In frozen plasma, about 90% of APLN-DM is recovered after 72h in solution.

Method-3 : Improvement of detection method:

The third step of the method development was aimed to improve the linearity of the method **(see Figure 3).** Studies were performed using the most abundant fragmented ion (z=6) and this step allowed to analyse the other ions as stated in the **Table 8.**

m/z	z
980.5	4
784.9	5
654.4	6

561.0	7

Table 18 - Fragmented ions obtained by APLN-DM analysis in mass spectrometry

Standard range from 0.5 to 100 µM was prepared and analysed for each fragmented ion as previously described in part 14.2. Standard curves obtained for each ion are represented in **Figure 6**.

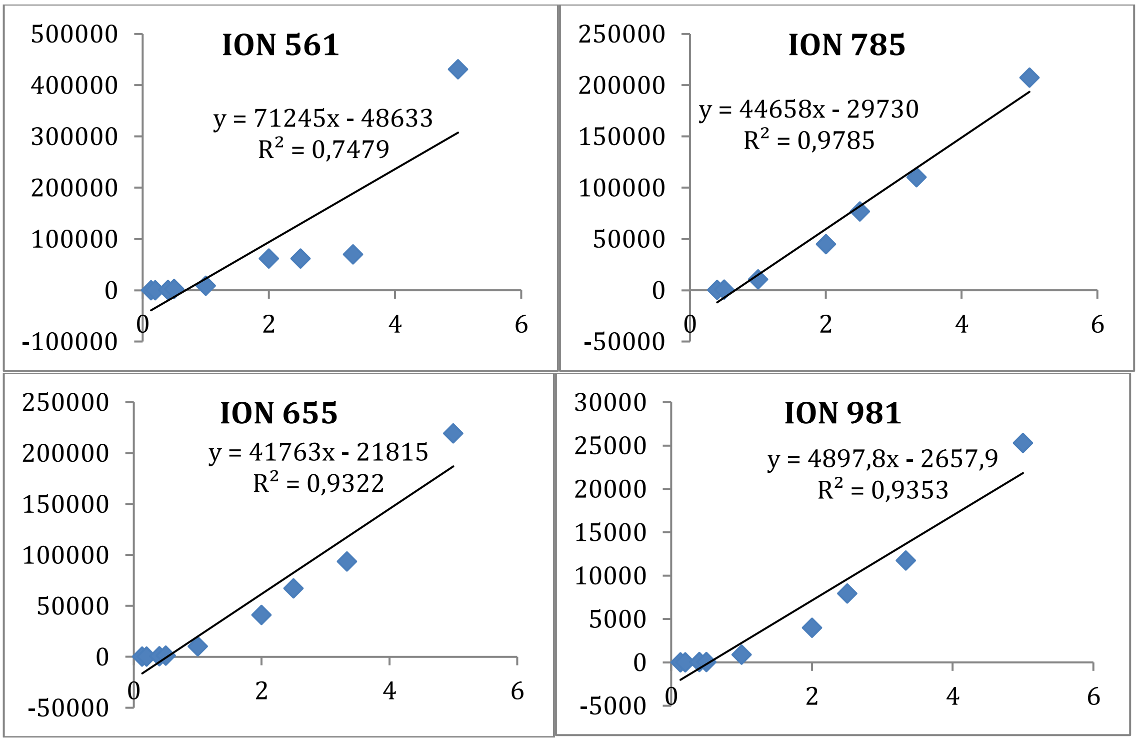

Figure 8: Standard curves obtained by quantification of each fragmented ion

The best linearity was obtained by quantifying the fragmented ion 785 (z=5).

Quantification of APLN-DM in samples

Summary of analytical method development:

Quantification method was performed according to previous work. In summary, samples were analysed with a LC-MS triple quadripole system (ESI QqQ). The chromatographic system was a UPLC activity (Waters) coupled to a mass spectrometer TSQ Quantum Ultra (Thermo) equipped with triple quadripole analyser and electrospray ionisation (ESI) system. The selected column was Kinetex F5 2.3 µM 50x2.1 mm (Phenomenex). The analysis was made by quantifying fragmented ion z=5 (m/z=785) in samples compared to a standard curve prepared with several concentrations of APLN-DM (0.5 to 100 µM). The limit of quantitation was previously determined to be 0.5 µM. Samples were analysed after protein precipitation as previously described.

***In vivo* phase samples analysis**

As the stability of peptide is low, even in frozen plasma, it was decided to re-run the in vivo phase for the highest dose and to use freshly prepared samples.

26 samples were used for analysis. Concentration of APLN-DM was measured less than 1 week after in vivo experiment.

Only samples taken 5 and 10 minutes after peptide administration presented detectable concentrations **(see Table 9)**. While 15 min results were on the level of accuracy (linearity threshold values). At a later time period 30 min to 24 hours values were below accurately detected level and hence peptide half-life was estimated based on the 5 and 10 min measurements.

Sample ID	Area	Calculated amount
2F 5' 1	127492.63	0.793
2F 5' 2	4002.30	0.661
2F 5' 3	4936.41	0.662
2F 10' 1	1647.27	0.658
2F 10' 2	889.11	0.658
2F 10' 3	839.06	0.657
2F 15' 1	BLD	BLD
2F 15' 2	BLD	BLD
2F 15' 3	BLD	BLD
2F 30' 1	BLD	BLD
2F 30' 2	BLD	BLD
2F 30' 3	BLD	BLD
2F 60' 1	BLD	BLD
2F 60' 2	BLD	BLD
2F 60' 3	BLD	BLD
2F 2H 1	BLD	BLD
2F 2H 2	BLD	BLD
2F 2H 3	BLD	BLD
2F 4H 1	BLD	BLD
2F 4H 2	BLD	BLD
2F 4H 3	BLD	BLD
2F 8H 1	BLD	BLD
2F 8H 2	BLD	BLD
2F 24H 1	BLD	BLD
2F 24H 2	BLD	BLD
2F 24H 3	BLD	BLD

Table 19 - Quantification of APLN-DM in samples obtained in vivo

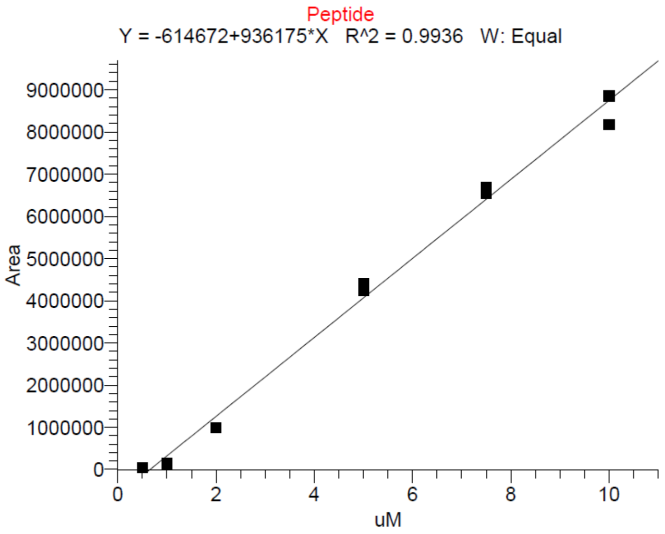

Figure 9 - Calibration curve for quantification of samples

These results allowed only to estimate concentrations of APLN-DM in samples and did not allow to calculate additional PK parameters. Half-life of APLN-DM in plasma of Balb/C mice was estimated to be 20 minutes

CONCLUSIONS

This study was aimed to evaluate pharmacokinetic parameters of APLN-DM in plasmas of Balb/C mice. The peptide was shown to not be stable in water or plasma and the quantification was performed in samples obtained within several days in order to avoid peptide loss. APLN-DM was detected only in samples obtained 5 and 15 minutes after the administration. The half-life of the peptide was estimated to be 20 minutes.

Supplemental study 4
ADME-Tox study of APLN-DM

Materials and methods

Experimental conditions

ADME-Tox: solution properties

Assay	Technique	Incubation	Detection method	Ref. 4
Aqueous solubility (PBS, pH 7.4)	Shake-flask	24h RT	HPLC-UV/VIS	327
Protein binding (plasma, human)	Equilibrium dialysis	4h 37°C	HPLC-MS/MS	640
Protein binding (plasma, mouse CD-1)	Equilibrium dialysis	4h 37°C	HPLC-MS/MS	640

Table 20- Solution property assay conditions

ADME-Tox: in vitro metabolism

Assay	Source	Substrate	Incubation	Measured component	Detection method	Ref. 4
**CYP1A2 inhibition (recombinant, CEC substrate)**	Human recombinant CYP	CEC (5 µM)	30min 37°C	CHC	Fluorimetry	51
**CYP2B6 inhibition (recombinant, EFC substrate)**	Human recombinant CYP	EFC (1.5 µM)	30min 37°C	HFC	Fluorimetry	62
**CYP2C8 inhibition (recombinant, DBF substrate)**	Human recombinant CYP	DBF (0.5 µM)	50 min 37°C	Fluorescein	Fluorimetry	532
**CYP2C9 inhibition (recombinant, MFC substrate)**	Human recombinant CYP	MFC (75 µM)	40 min 37°C	HFC	Fluorimetry	51
**CYP2C19 inhibition (recombinant, CEC substrate)**	Human recombinant CYP	CEC (10 µM)	50 min 37°C	CHC	Fluorimetry	178
**CYP2D6 inhibition (recombinant, MFC substrate)**	Human recombinant CYP	MFC (50 µM)	40 min 37°C	HFC	Fluorimetry	178
**CYP3A4 inhibition (recombinant, BFC substrate)**	Human recombinant CYP	BFC (50 µM)	30 min 37°C	HFC	Fluorimetry	513
**CYP3A4 inhibition (recombinant, BzRes substrate)**	Human recombinant CYP	BzRes (1 µM)	20 min 37°C	Resorufin	Fluorimetry	513
**Half-life (plasma, human)**	Human plasma	Test compound	0, 0.5, 1, 1.5, 2 hr 37°C	Test compound	HPLC-MS/MS	1127

**Intrinsic clearance (liver microsomes, human)**	Human liver microsomes (0.1 mg/mL)	Test compound	0, 15, 30, 45, 60 min 37°C	Test compound	HPLC-MS/MS	828
**Intrinsic clearance (liver microsomes, mouse, CD-1)**	Mouse liver microsomes (0.1 mg/mL)	Test compound	0, 15, 30, 45, 60 min 37°C	Test compound	HPLC-MS/MS	828

Table 21- In vitro metabolism assays conditions

ADME-Tox: cardiac toxicity

Assay	Source	Technique	Incubation	Detection method	Ref. 4
hERG (hERG-CHO, automated patch-clamp)	hERG CHO-K1 cell line	Patch clamp	5min RT, cumulatively	Automated whole-cell patch clamp	872

Table 22- Cardiac toxicity assays conditions

Analysis and results

ADME-Tox: solution properties

Aqueous Solubility

Aqueous solubility (µM) was determined by comparing the peak area of the principal peak in a calibration standard (200 µM) containing organic solvent (methanol/water, 60/40, v/v) with the peak area of the corresponding peak in a buffer sample. In addition, chromatographic purity (%) was defined as the peak area of the principal peak relative to the total integrated peak area in the HPLC chromatogram of the calibration standard. A chromatogram of the calibration standard of each test compound, along with a UV/VIS spectrum with labeled absorbance maxima, was generated.

Protein Binding

The peak areas of the test compound in the buffer and test samples were used to calculate percent binding and recovery according to the following formulas:

$$\text{Protein binding}(\%) = \frac{Area_p - Area_b}{Area_p} * 100$$

$$\text{Recovery}(\%) = \frac{Area_p + Area_b}{Area_c} * 100$$

where

$Area_p$ = Peak area of analyte in protein matrix

$Area_b$ = Peak area of analyte in buffer

$Area_C$ = Peak area of analyte in control sample

ADME-Tox: in vitro metabolism

Cytochrome P450 Inhibition (fluorometric detection).

The fluorescent intensity (*fu*) measured at (t = 0) was subtracted from that measured after the appropriate incubation time (t = final). The percent of control activity was then calculated by comparing the corrected fluorescence reading obtained in the presence of the test compound to that obtained in the absence of the test compound. Subsequently, the percent inhibition was calculated by subtracting the percent control activity from 100. IC_{50} values (concentration causing a half-maximal inhibition of control values) were determined by non-linear regression analysis of the concentration-response curves using Hill equation curve fitting.

Half-Life Determination (plasma or blood)

At the end of incubation at each of the time points, acetonitrile was added to the incubation mixture followed by centrifugation. Samples were analyzed by HPLC-MS/MS and peak areas were recorded for each analyte. The area of precursor compound remaining after each of the time points relative to the amount remaining at time zero, expressed as percent, was calculated. Subsequently, the half-life ($T_{1/2}$) is estimated from the slope of the initial linear range of the logarithmic curve of compound remaining (%) *versus* time, assuming first order kinetics.

Intrinsic Clearance (microsomes, S9, cryopreserved hepatocytes, recombinant CYP, recombinant UGT)

Metabolic stability, expressed as percent of the parent compound remaining, was calculated by comparing the peak area of the compound at the time point relative to that at time-0. The half-life ($T_{1/2}$) was estimated from the slope of the initial linear range of the logarithmic curve of compound remaining (%) vs. time, assuming the first-order kinetics.

The apparent intrinsic clearance (CL_{int}, in µL/min/pmol, µL/min/mg or µL/min/Mcell) was calculated according to the following formula:

$$CL_{int} = \frac{0.693}{T_{1/2} * (\text{mg protein/µL or million cells/µL or pmol CYP isoyme/µL})}$$

ADME-Tox: cardiac toxicity

hERG (automated patch-clamp)

The degree of inhibition (%) was obtained by measuring the tail current amplitude, which is induced by a one second test pulse to 40mV after a two second pulse to +20mV, before and

after drug incubation (the difference current was normalized to control and multiplied by 100 to obtain the percent of inhibition).

Results

ADME-Tox: solution properties results

Protein binding results

Assay	Test [C] APLN-DM	% Protein bound			% Recovery		
		1st	2nd	Mean	1st	2nd	Mean
Protein binding (plasma, human)	1.0E-05M	56.8	52.2	54	123	125	124
Protein binding (plasma, mouse CD-1)	1.0E-05M	26.1	26.9	26	93	93	93

Table 23- Protein binding assays results of M-Apelin-36

At the tested concentration of 10^{-5}M of APLN-DM peptide, mean protein bound was found to be 54% and 26% for human and mouse CD-1 plasma, respectively. Moreover, the % recovery was 124% for human plasma and 93% for mouse CD-1 plasma.

Reference compound results

Assay	Reference compound	Test [C]	% Protein bound			% Recovery		
			1st	2nd	Mean	1st	2nd	Mean
Protein binding (plasma, human)	Acebutolol	1.0E-05M	37.4	29.5	33	86	71	79
	Quinidine	1.0E-05M	75.1	75.1	75	91	88	89
	Warfarin	1.0E-05M	99.3	99.4	99	94	88	91
Protein binding (plasma, mouse CD-1)	Acebutolol	1.0E-05M	12.4	32.9	23	82	60	71
	Quinidine	1.0E-05M	39.2	44.6	42	54	43	48
	Warfarin	1.0E-05M	87.0	87.1	87	59	54	57

Table 24- Protein binding assays results of reference compounds for comparison

Aqueous solubility results

Assay	Test [C] APLN-DM	Wavelength of detection (nm)	Solubility (µM)			Chromatographic purity (%)
			1st	2nd	Mean	
Aqueous solubility (PBS, pH 7.4)	2.0E-04 M	230	183.14	191.37	187.3	100

Table 25- Aqueous solubility results of APLN-DM

At 2.10^{-4}M (200µM), the solubility of APLN-DM in PBS (aqueous solution) was found to be 187.3µM.

Reference compound results

Assay	Reference compound	Test [C]	Wavelength of detection (nm)	Solubility (µM)			Chromatographic purity (%)
				1st	2nd	Mean	
Aqueous solubility (PBS, pH 7.4)	Diethylstilbestrol	2.0E-04 M	230	49.32	13.64	31.5	100
	Ketoconazole	2.0E-04 M	230	134.79	138.59	136.7	100
	Metoprolol tartrate	2.0E-04 M	230	233.48	225.55	200*	100
	Phenytoin	2.0E-04 M	230	78.43	88.70	83.6	100
	Rifampicin	2.0E-04 M	230	227.69	234.78	200*	100
	Simvastatin	2.0E-04 M	230	9.99	11.22	10.6	100
	Tamoxifen	2.0E-04 M	230	19.07	17.15	18.1	100

Table 26- Aqueous solubility results of reference compounds. * when the observed mean solubility was greater than 200µM, the mean value was adjusted to the maximum assay concentration, which is 200µM.

ADME-Tox: In vitro metabolism results

Half-life and intrinsic clearance results

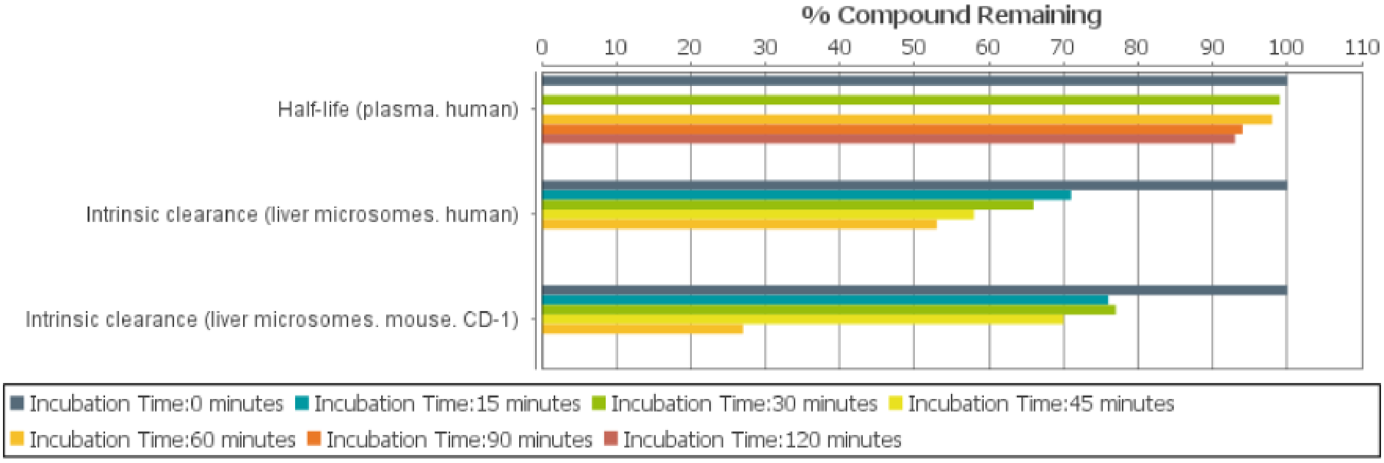

Figure 10- Half-life and intrinsic clearance histogram results for APLN-DM

Assay	Test [C] APLN-DM	Incubation time (min)	% Compound remaining			Half-life (min)			CL_{int}
			1st	2nd	Mean	1st	2nd	Mean	
Half-life (plasma, human)	1.0E-06M	0	100.0	100.0	100	1270.4	972.5	1121	
	1.0E-06M	30	97.6	99.8	99				
	1.0E-06M	60	97.8	98.5	98				
	1.0E-06M	90	94.6	93.0	94				
	1.0E-06M	120	(100.6)	93.1	93				

Intrinsic clearance (liver microsomes, human)	1.0E-07M	0	100.0	100.0	100	74.4	68.2	>60	<115.5
	1.0E-07M	30	62.7	79.6	71				
	1.0E-07M	60	63.8	67.3	66				
	1.0E-07M	90	(40.7)	58.3	58				
	1.0E-07M	120	52.2	54.5	53				
Intrinsic clearance (liver microsomes, mouse CD-1)	1.0E-07M	0	100.0	100.0	100	38.7	112.0	>60	<115.5
	1.0E-07M	30	68.4	82.7	76				
	1.0E-07M	60	77.4	(186.7)	77				
	1.0E-07M	90	66.0	74.2	70				
	1.0E-07M	120	26.6	(37.6)	27				

Table 27- Half-life and intrinsic clearance results of APLN-DM

Note: unit of CL_{int} is µL/min/mg for microsomes, S9 and UGT assays; µL/min/pmol for CYP assays; µL/min/million cells for hepatocyte assays.

Stability evaluation in human plasma at the concentration tested of 10^{-6}M resulted in half-life of 1121 minutes (> 18 hours).

Intrinsic clearance measurement in human or mouse CD-1 liver microsomes at 10^{-7}M resulted in CL_{int} of <115.5µL/min/mg.

Reference compound results

Assay	Reference compound	Test [C]	Half-life (min)			CL_{int}
			1st	2nd	Mean	
Half-life (plasma, human)	Propantheline	1.0E-06M	12.0	11.9	12	
	Propoxycaine	1.0E-06M	2.5	2.4	2	
Intrinsic clearance (liver microsomes, human)	Imipramine	1.0E-07M	124.4	130.0	>60	<115.5
	Propranolol	1.0E-07M	188.8	104.0	>60	<115.5
	Terfenadine	1.0E-07M	25.0	25.3	25	275.5
	Verapamil	1.0E-07M	31.7	35.3	34	207.3
Intrinsic clearance (liver microsomes, mouse CD-1)	Imipramine	1.0E-07M	21.2	22.4	22	317.9
	Terfenadine	1.0E-07M	17.2	17.6	17	397.7
	Verapamil	1.0E-07M	28.7	27.1	28	248.4

Table 28- Half-life and intrinsic clearance results of reference compounds

CYP inhibition results

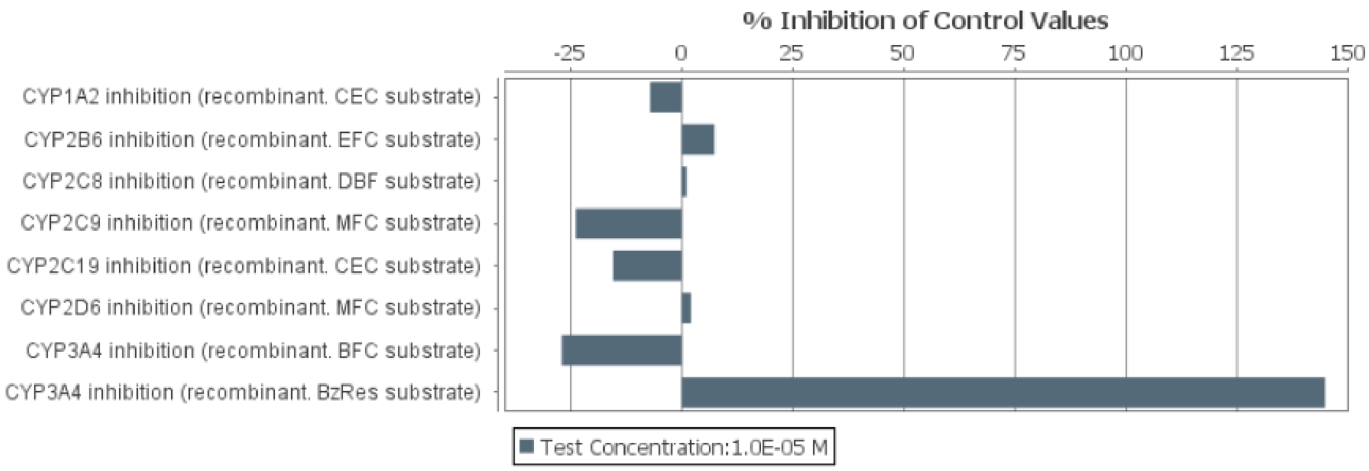

Figure 11- CYP inhibition histogram results

Cytochrome P450 in vitro assay results discovered an enzyme inhibition only for the target CYP3A4 (145% inhibition of control values).

Assay	Test [C] APLN-DM	% Inhibition of control values		
		1st	2nd	Mean
**CYP1A2 inhibition (recombinant, CEC substrate)**	1.0E-05M	-12.0	-2.2	-7.1
**CYP2B6 inhibition (recombinant, EFC substrate)**	1.0E-05M	1.3	13.5	7.4
**CYP2C8 inhibition (recombinant, DBF substrate)**	1.0E-05M	-1.4	3.6	1.1
**CYP2C9 inhibition (recombinant, MFC substrate)**	1.0E-05M	-33.1	-14.6	-23.9
**CYP2C19 inhibition (recombinant, CEC substrate)**	1.0E-05M	-24.6	-6.2	-15.4
**CYP2D6 inhibition (recombinant, MFC substrate)**	1.0E-05M	4.3	-0.2	2.1
**CYP3A4 inhibition (recombinant, BFC substrate)**	1.0E-05M	-30.1	-23.9	-27.0
**CYP3A4 inhibition (recombinant, BzRes substrate)**	1.0E-05M	109.5	180.5	**145.0**

Table 29- CYP inhibition results for APLN-DM

Reference compounds results

Assay	Reference compound	IC_{50} (M)	nH
**CYP1A2 inhibition (recombinant, CEC substrate)**	Furafylline	3.6E-06M	1.1
**CYP2B6 inhibition (recombinant, EFC substrate)**	Ketoconazole	5.4E-06M	0.9
**CYP2C8 inhibition (recombinant, DBF substrate)**	Quercetin	2.6E-06M	2.5
**CYP2C9 inhibition (recombinant, MFC substrate)**	Sulfaphenazole	1.1E-07M	0.6
**CYP2C19 inhibition (recombinant, CEC substrate)**	Tranylcypromine	5.1E-06M	0.7
**CYP2D6 inhibition (recombinant, MFC substrate)**	Quinidine	1.2E-08M	1.7
**CYP3A4 inhibition (recombinant, BFC substrate)**	Ketoconazole	1.9E-07M	2.3
**CYP3A4 inhibition (recombinant, BzRes substrate)**	Ketoconazole	4.2E-09M	>3

Table 30- CYP inhibition results of reference compounds

ADME-Tox: cardiac toxicity results

Assay	Test [C] APLN-DM	% Inhibition of tail current		
		1st	2nd	Mean
hERG (hERG-CHO, automated patch-clamp)	1.0E-07M	6.7	3.3	5.0
	1.0E-06M	6.0	4.5	5.2
	1.0E-05M	13.4	8.4	10.9

Table 31- hERG patch-clamp results for APLN-DM

APLN-DM at 10^{-5}M, 10^{-6}M and 10^{-7}M induced 10.9%, 5.2% and 5.0% (respectively) inhibition of tail current in hERG cardiac toxicity assay.

Reference compounds results

Assay	Reference compound	IC50 (M)	nH
hERG (hERG-CHO, automated patch-clamp)	E-4031	4.5E-08M	2.1

Table 32- hERG patch-clamp results of reference compounds

Conclusions

In solution properties

- **Plasma binding protein**

The binding of therapeutic compounds to plasma or serum proteins is a reversible, saturable process which can be an important factor in assessing the pharmacokinetic and pharmacodynamic profile of a drug. Indeed, the extent of binding to plasma influences the way in which a drug distributes into tissues in the body (*e.g.* if a compound is highly bound, it is retained in the plasma, which results in a low volume of distribution. On the contrary, low protein binding means more drug is free to partition into tissues and will result in a high volume of distribution). High protein binding of a compound can have important clinical effects with regard to drug toxicity (drug interactions or disease state). When greater than 90% of the compound is bound to plasma proteins, this compound is considered to bind extensively to plasma proteins and plasma protein binding is likely to have a significant impact. Protein binding results in human or mouse CD-1 plasma indicated that it is not the case for the peptide M-Apelin-36. Moreover, in theory, the recovery should be around 100%. As the % recovery for APLN-DM was found to be 124% and 93% for human and mouse CD-1 plasma, respectively, no indication of binding to the dialysis equipment or solubility issues was found.

- **Aqueous solubility**

APLN-DM was found to be soluble in hydrophilic (PBS) solution. This might be an important parameter in future studies and potential clinical formulations preparation.

<u>In vitro metabolism</u>

- **Half-life in plasma and intrinsic clearance in liver microsomes**

Stability evaluation in human plasma at the concentration tested of 10^{-6}M resulted in half-life of >18 hours suggesting relatively high stability to plasma degrading enzymes (such as proteases).

The liver is the main organ of drug metabolism in the body. Subcellular fractions such as liver microsomes are useful *in vitro* models as they contain many of the drug metabolizing enzymes

found in the liver. The intrinsic clearance (CL_{int}) is a kinetic parameter that can be used to evaluate hepatic clearance and to determine if a drug is stable in liver microsomes in the presence of NADPH. Unless the compound is a pro-drug, very highly cleared compounds are generally considered to be unfavorable as they are likely to be rapidly cleared *in vivo* resulting in a short duration of action. Classification bands can be used to categorize compounds into low, medium or high clearance. Low clearance is considered when CL_{int} in human or mouse is <8.6 µL/min/mg low and high clearance when CL_{int} is >47.0 µL/min/mg. For APLN-DM, intrinsic clearance measurement in human or mouse CD-1 liver microsomes resulted in CL_{int} of <115.5 µL/min/mg indicating that the clearance category of the compound is high and that the drug is probably metabolized by CYPs.

- **CYP inhibition**

Cytochrome P450 are a family of enzymes which play a major role in the metabolism of drugs. Assessment of the potential of a compound to inhibit a specific cytochrome P450 enzyme is important as this may affect plasma levels *in vivo* and potentially lead to adverse drug reactions or toxicity. Therefore, CYP phenotyping (to identify which CYP isoform is responsible for the metabolism) appears crucial in early drug discovery. The result of CYP phenotyping will provide information whether the drug is susceptible to CYP polymorphic effects or is a victim of potential drug-drug interactions. Although the criteria for acceptance is isoform-specific, potent inhibition is considered unfavorable and may preclude the development of a compound. Cytochrome P450 in vitro assay with the peptide APLN-DM discovered enzyme inhibition for the CYP3A4 isoform. It is not surprising as this enzyme is involved in the metabolism (deactivation) of approximately half the drugs that are used today. Knowing that, it will be important for the future to take care of possible drug-drug interactions. Indeed, it has been shown that some substances are activated by the enzyme -like grapefruit juice- interfering with its action (either amplify or weaken the action of those drugs that are modified by CYP3A4).

- **Cardiac toxicity**

The human ether-a-go-go related gene (hERG) encodes the inward rectifying voltage gated potassium channel in the heart (I_{Kr}) which is involved in cardiac repolarization. Inhibition of the hERG current causes QT interval prolongation resulting in potentially fatal ventricular tachyarrhythmia called *Torsade de Pointes*. As a number of drugs have been withdrawn from late stage clinical trials due to these cardio-toxic effects, it is important to identify inhibitors early in drug discovery. Using hERG patch-clamp automated technique, APLN-DM potential

cardiac toxicity was investigated at the concentrations of 10^{-5}M, 10^{-6}M and 10^{-7}M. Results revealed negligible dose-related % inhibition of tail current (5-10.9%). This range of concentration is acceptable in the literature for cardio-toxicity assays. It is considered that a drug that does not produce a signal (50% of inhibition), within a 30-fold margin of the clinically relevant concentration, is unlikely to produce a QT prolongation effect.

In summary, overall ADME-Tox study results revealed that APLN-DM peptide is soluble in aqueous solution and stable in the plasma. Protein binding assay discovered that APLN-DM is not bound extensively to plasma protein. Tested item appeared to be metabolized by the liver and to affect CYP3A4 inhibiting metabolism of subset of substrates being metabolized by this cytochrome (as revealed by the difference inhibition profile between BzRes and BFC). Finally, the hERG cardiac channel assay results suggested that APLN-DM does not interact with this channel.

References

References relative to experimental conditions:

51. Crespi, C.L. et al. (1997), *Anal. Biochem.*, 248 : 188-190.
62. Ekins, S. et al. (1997), *Pharmacogenetics*, 7 : 165-179.
178. Ono, S. et al. (1996), *Xenobiotica*, 26 : 681-693.
327. Lipinski, C.A. et al. (1997), *Adv. Drug Del. Rev.*, 46 : 3-26.
513. Stresser, D.M. et al. (2000), *Drug Metab. Dispos.*, 28 : 1440-1448.
532. Miller, V.P. et al. (2000), *Ann. N.Y. Acad. Sci.*, 919 : 26-32.
640. Banker, M.J. et al. (2003), *J. Pharm. Sci.*, 92 : 967-974.
828. Obach, R.S. et al. (1997), *J. Pharmacol. Exp. Ther.*, 283 : 46-58.
872. Mathes, C. (2006), *Expert Opin. Ther. Targets*, 10 (2) : 230-241.
1127. Di, L. et al. (2005), *Int. J. Pharm.*, 297 (1-2): 110-9.
