# Supplementary figures and images for: Repression of apelin Furin cleavage sites provides antimetastatic strategy in colorectal cancer (part 1 of 2)
Source: EMBO Mol Med. 2025 Feb 17;17(3):504–34. doi: 10.1038/s44321-025-00196-5 (PMC11904221; doi:10.1038/s44321-025-00196-5)

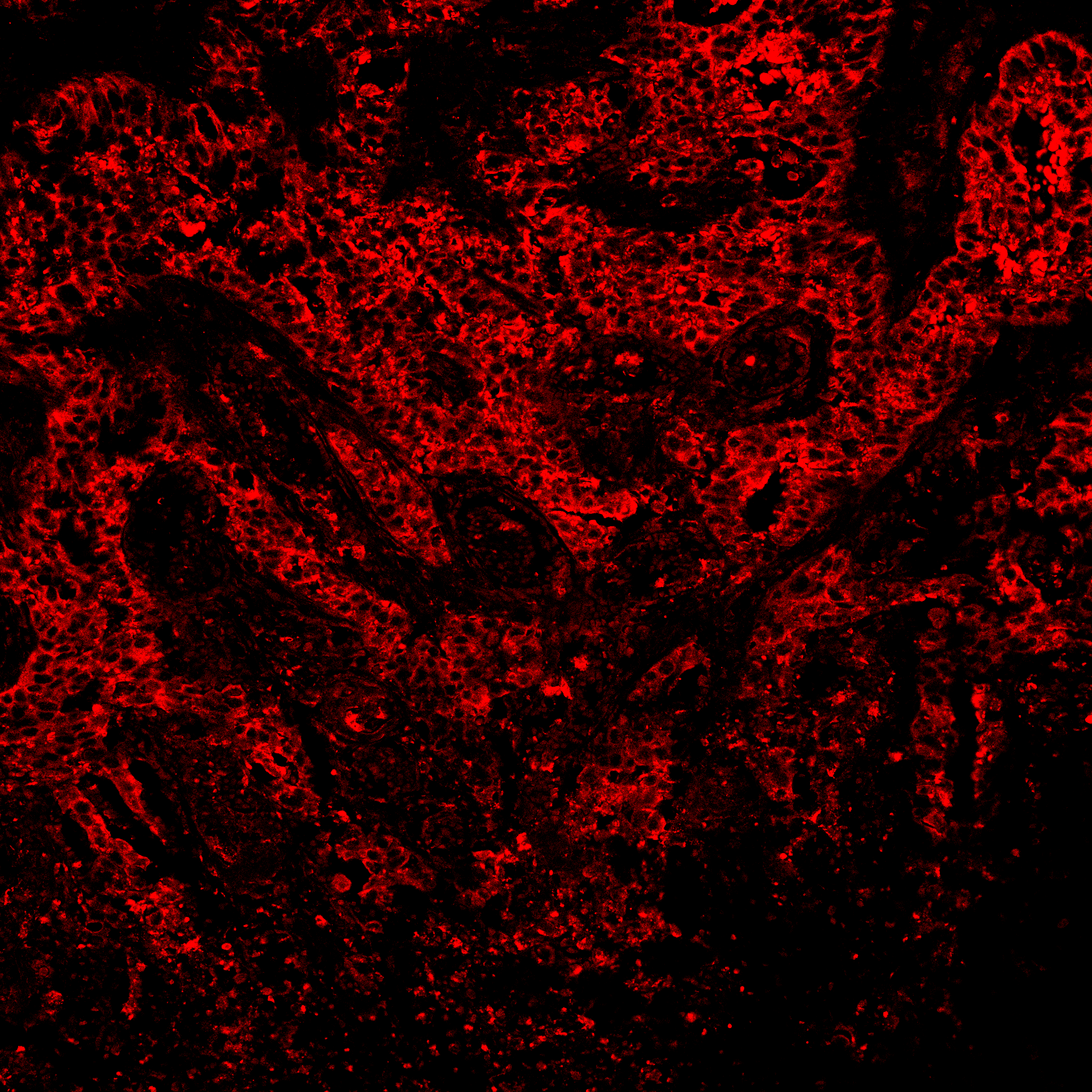

Supplement: Supplementary file 4 — Source data Fig. 1 [file 44321_2025_196_MOESM4_ESM.zip › MM-2024-19448_SourceDataForFig 1/EMM-2024-19448_SourceDataForFig 1E/CRC Apelin.png]

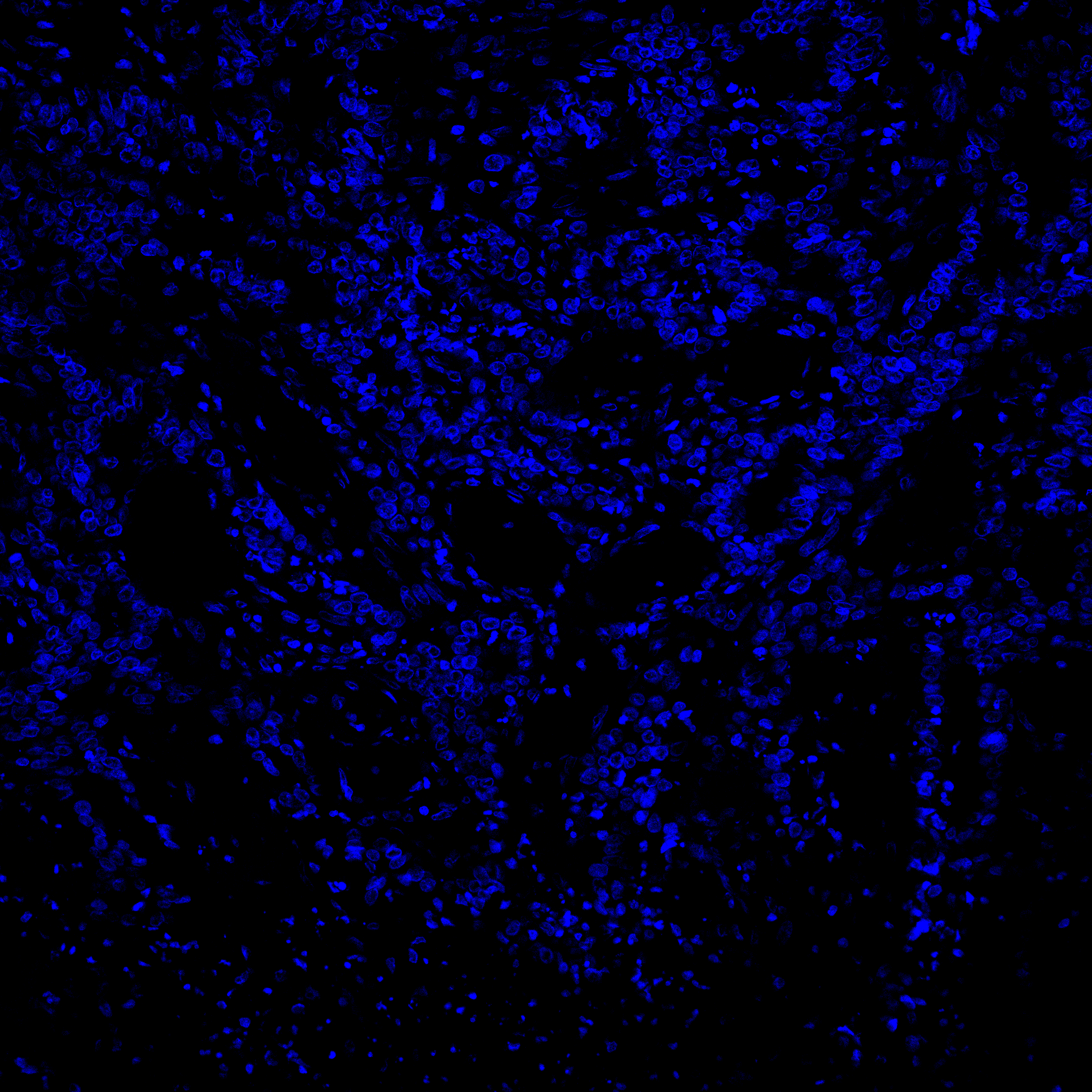

Supplement: Supplementary file 4 — Source data Fig. 1 [file 44321_2025_196_MOESM4_ESM.zip › MM-2024-19448_SourceDataForFig 1/EMM-2024-19448_SourceDataForFig 1E/CRC Dapi.png]

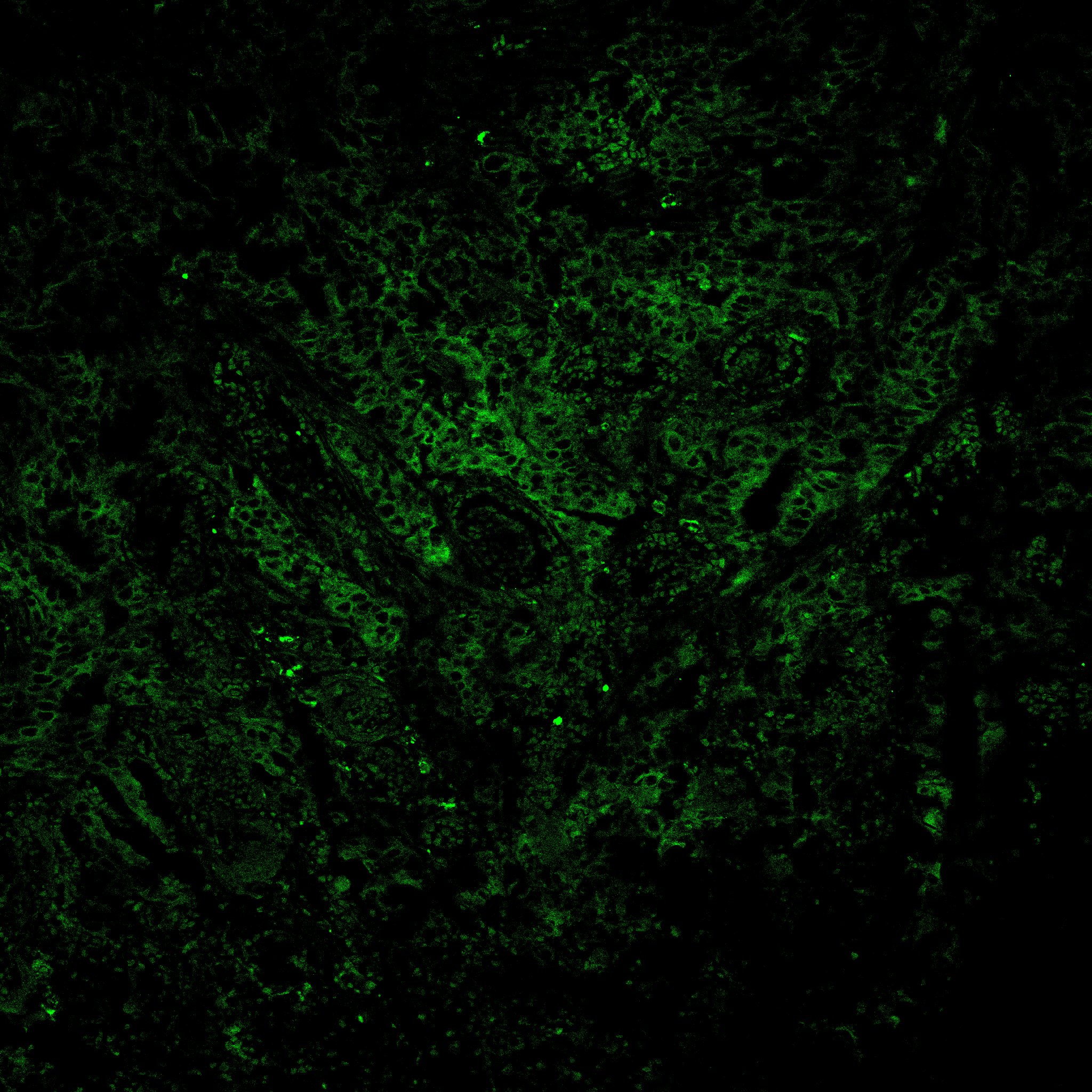

Supplement: Supplementary file 4 — Source data Fig. 1 [file 44321_2025_196_MOESM4_ESM.zip › MM-2024-19448_SourceDataForFig 1/EMM-2024-19448_SourceDataForFig 1E/CRC Furin.png]

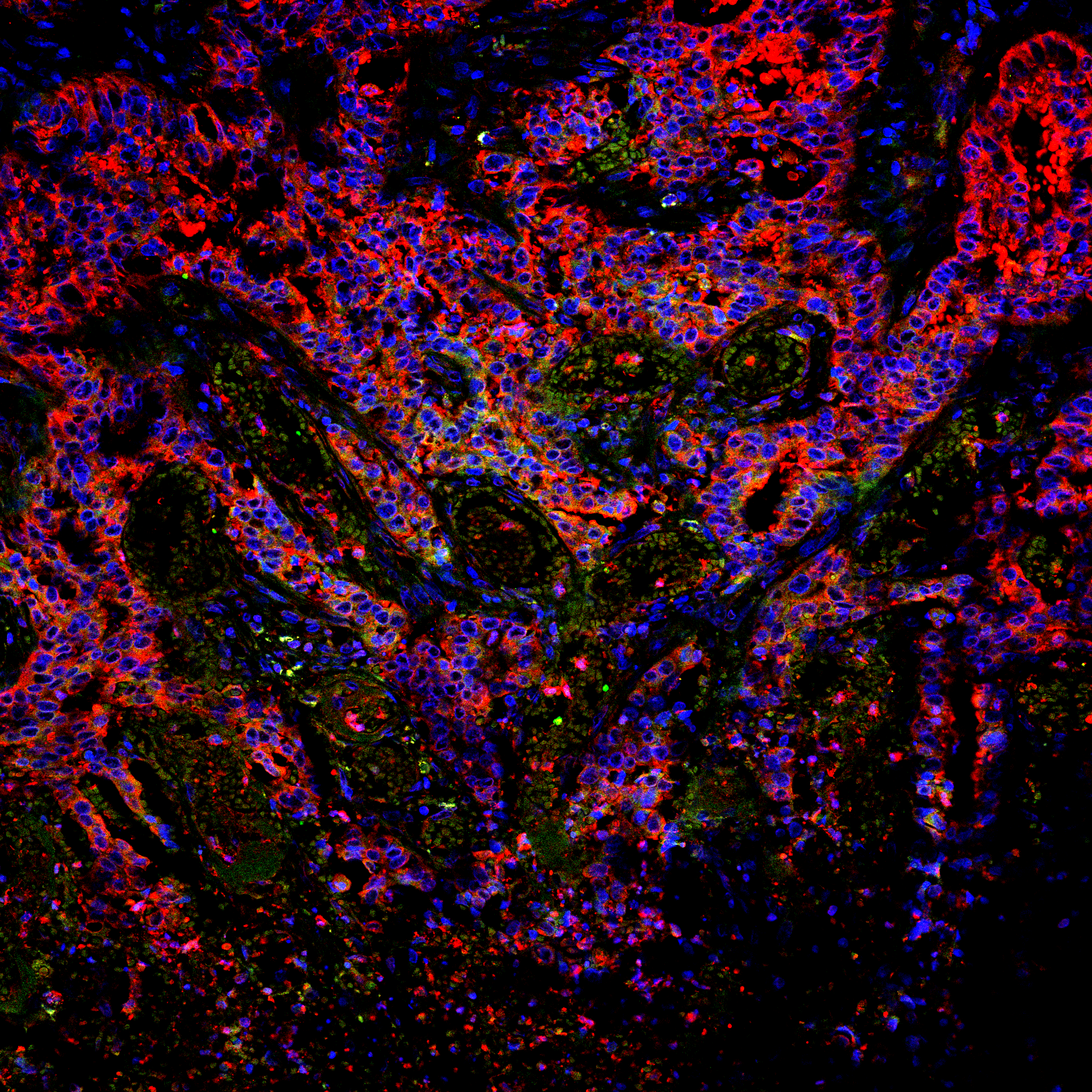

Supplement: Supplementary file 4 — Source data Fig. 1 [file 44321_2025_196_MOESM4_ESM.zip › MM-2024-19448_SourceDataForFig 1/EMM-2024-19448_SourceDataForFig 1E/CRC Merge.png]

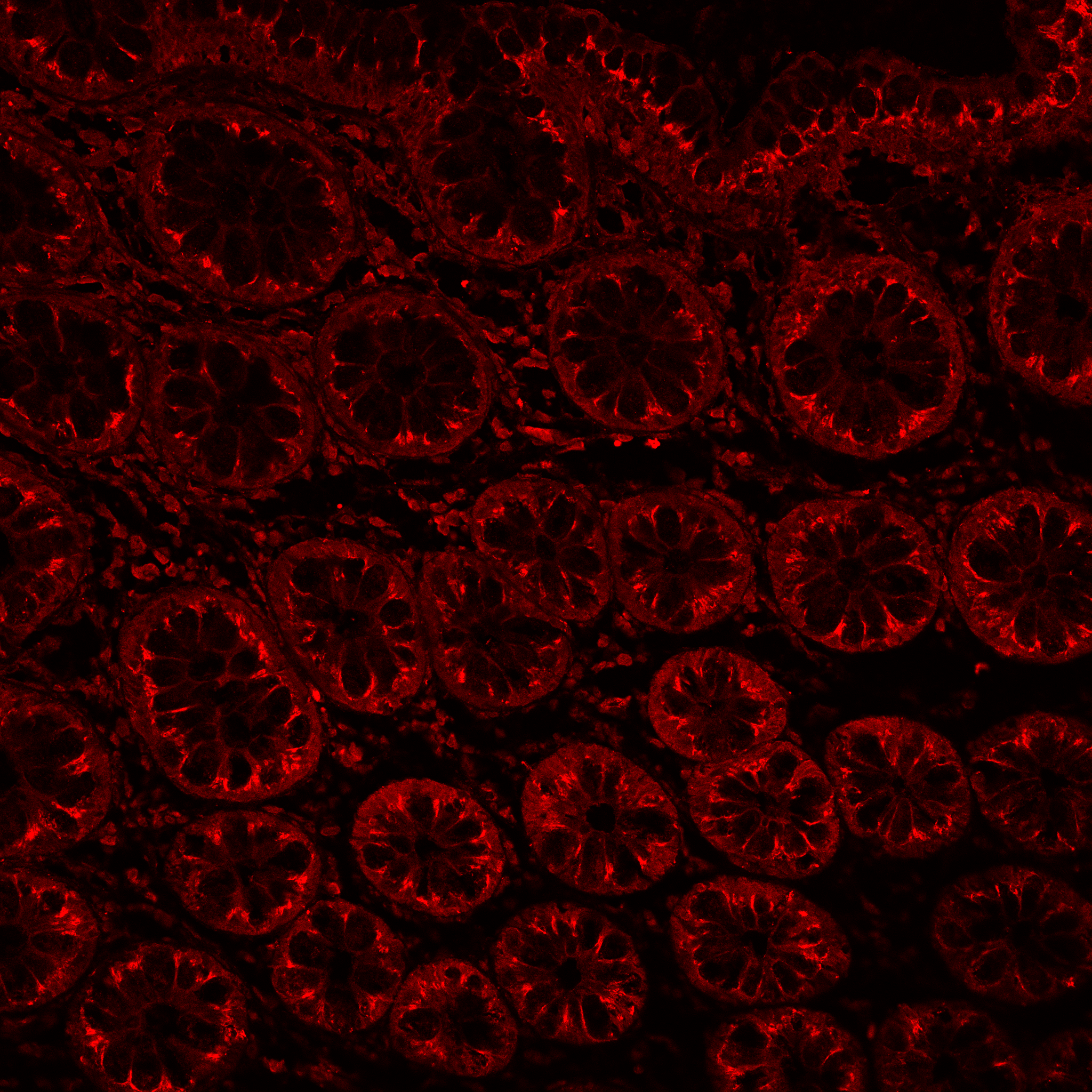

Supplement: Supplementary file 4 — Source data Fig. 1 [file 44321_2025_196_MOESM4_ESM.zip › MM-2024-19448_SourceDataForFig 1/EMM-2024-19448_SourceDataForFig 1E/Normal apelin.png]

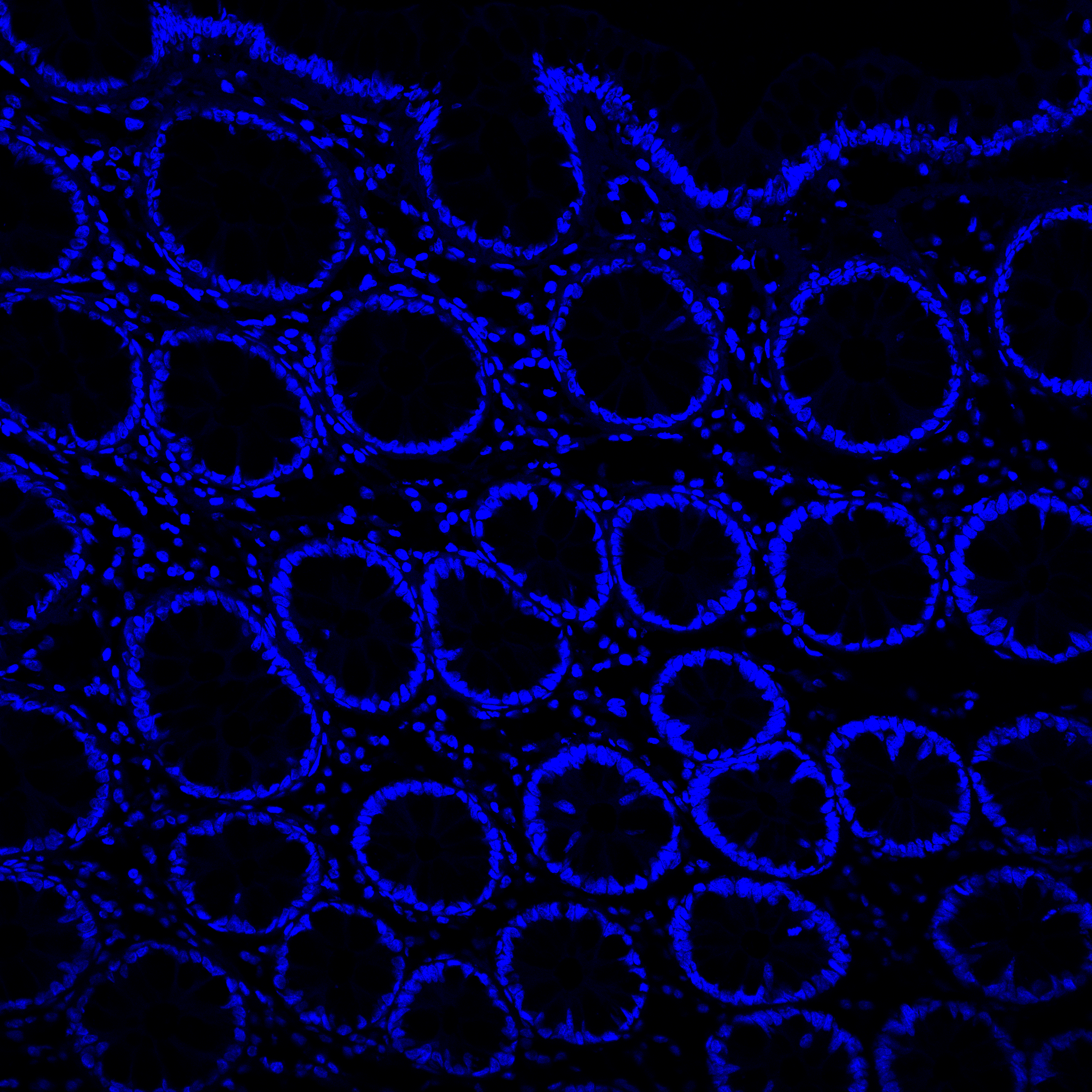

Supplement: Supplementary file 4 — Source data Fig. 1 [file 44321_2025_196_MOESM4_ESM.zip › MM-2024-19448_SourceDataForFig 1/EMM-2024-19448_SourceDataForFig 1E/Normal Dapi.png]

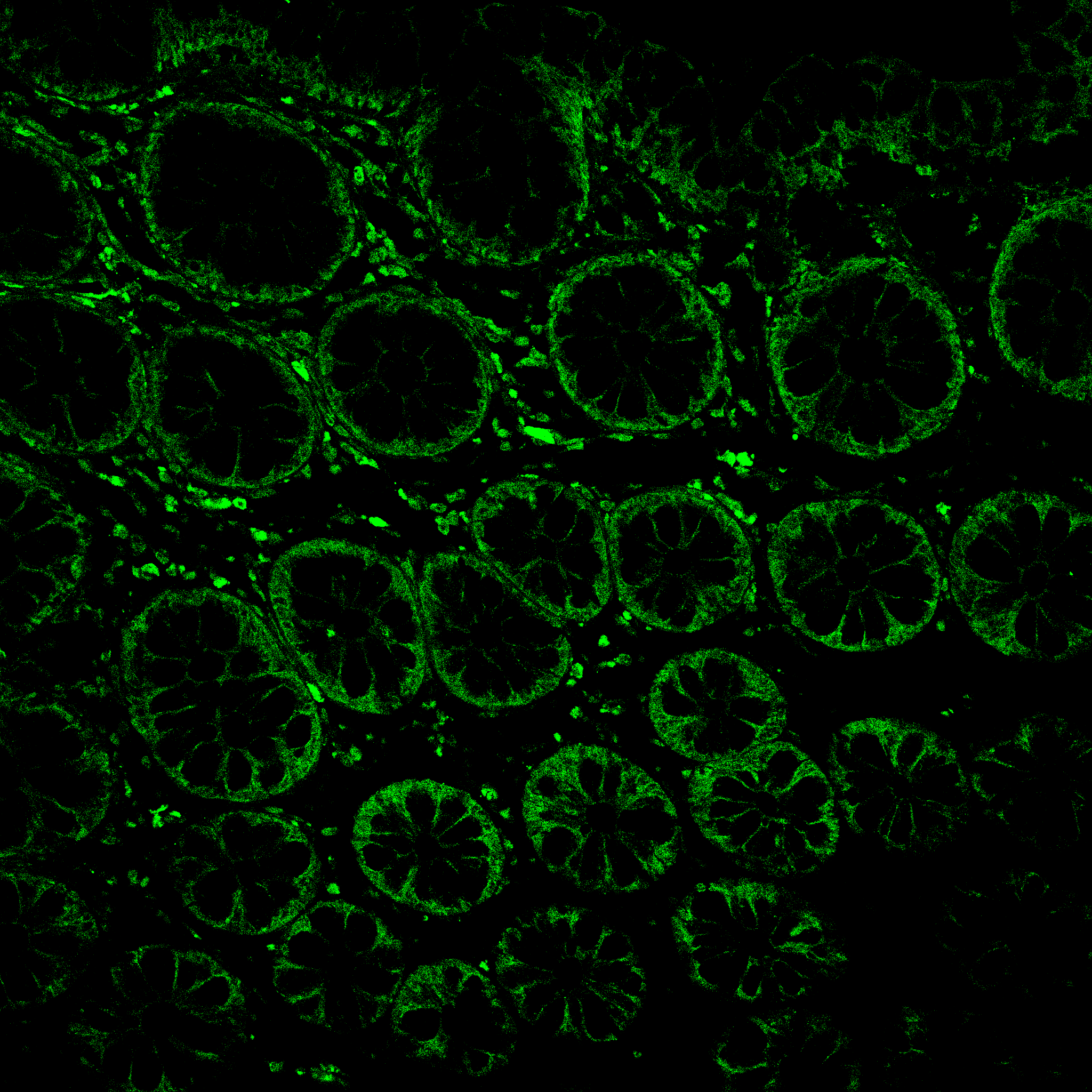

Supplement: Supplementary file 4 — Source data Fig. 1 [file 44321_2025_196_MOESM4_ESM.zip › MM-2024-19448_SourceDataForFig 1/EMM-2024-19448_SourceDataForFig 1E/Normal Furin.png]

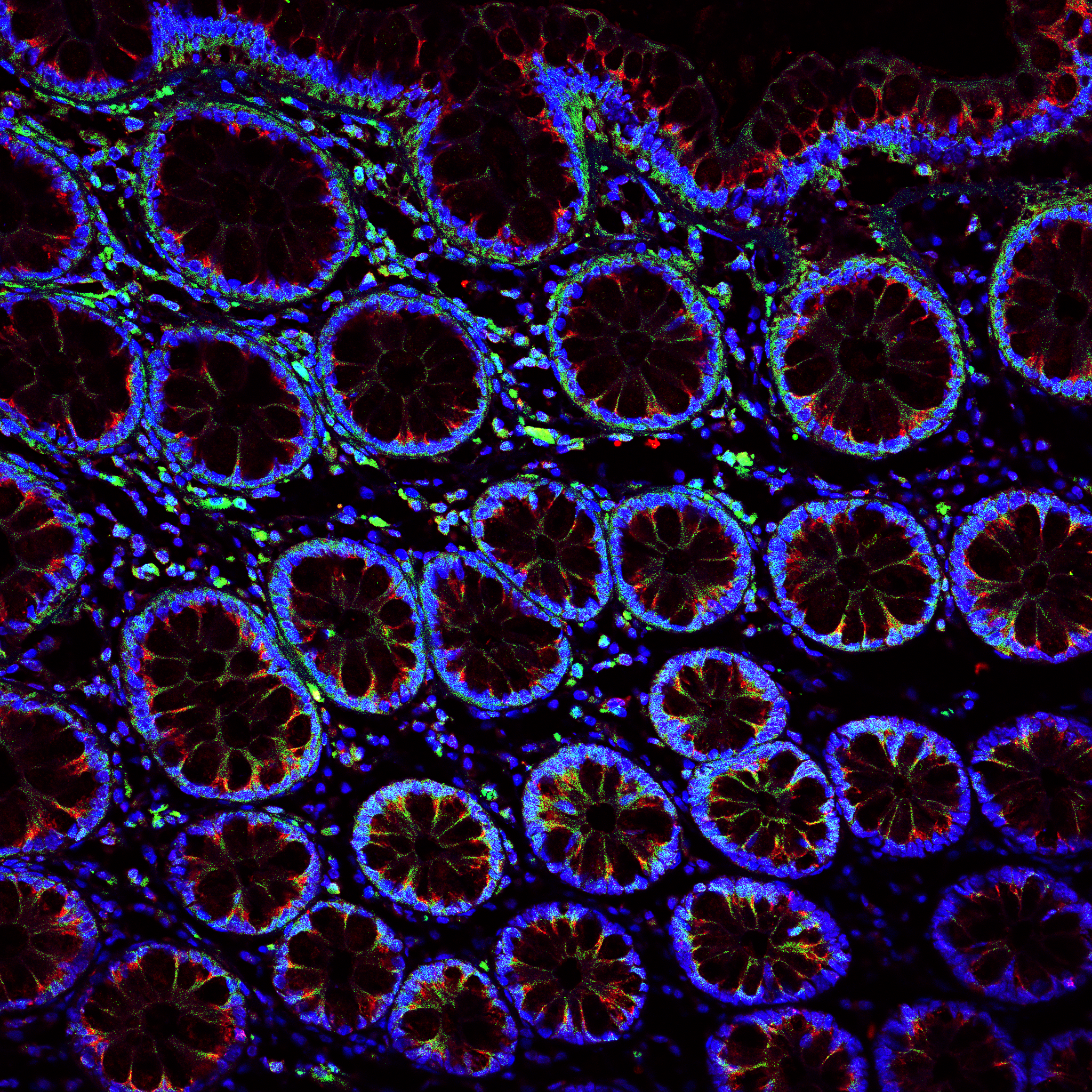

Supplement: Supplementary file 4 — Source data Fig. 1 [file 44321_2025_196_MOESM4_ESM.zip › MM-2024-19448_SourceDataForFig 1/EMM-2024-19448_SourceDataForFig 1E/Normal Merge.png]

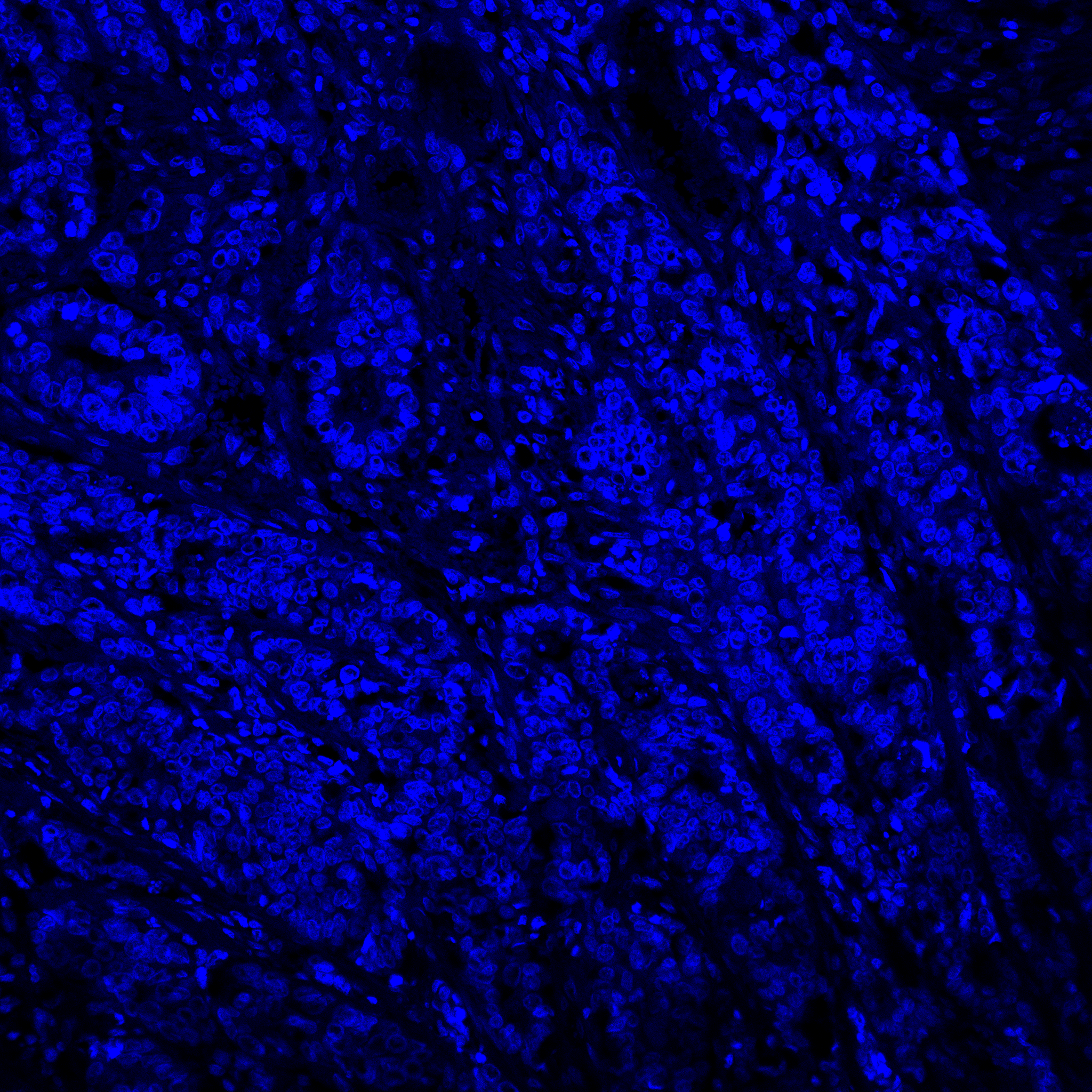

Supplement: Supplementary file 4 — Source data Fig. 1 [file 44321_2025_196_MOESM4_ESM.zip › MM-2024-19448_SourceDataForFig 1/Fig MM-2024-19448_SourceDataForFig 1F/CRC Dapi.png]

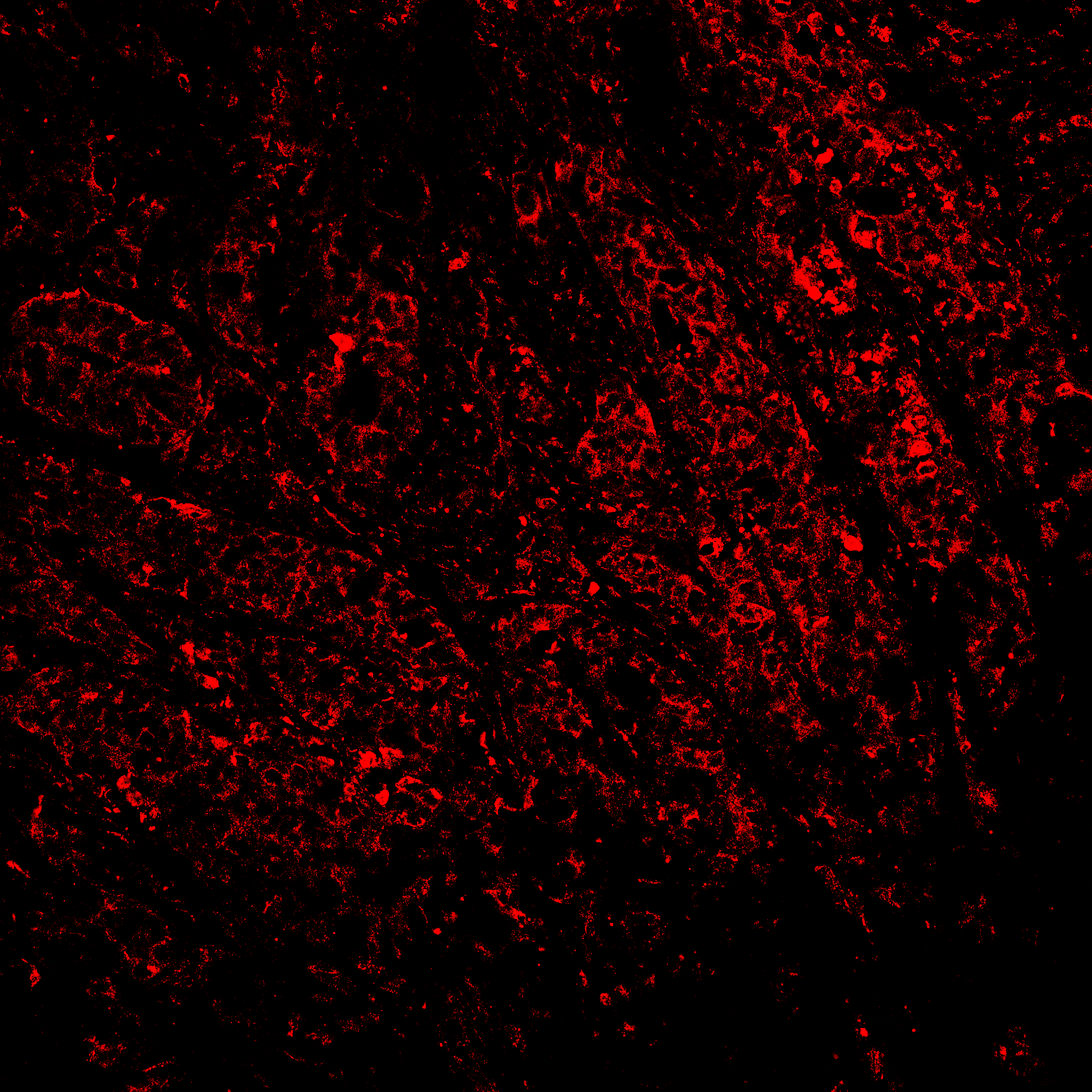

Supplement: Supplementary file 4 — Source data Fig. 1 [file 44321_2025_196_MOESM4_ESM.zip › MM-2024-19448_SourceDataForFig 1/Fig MM-2024-19448_SourceDataForFig 1F/CRC Apelin.png]

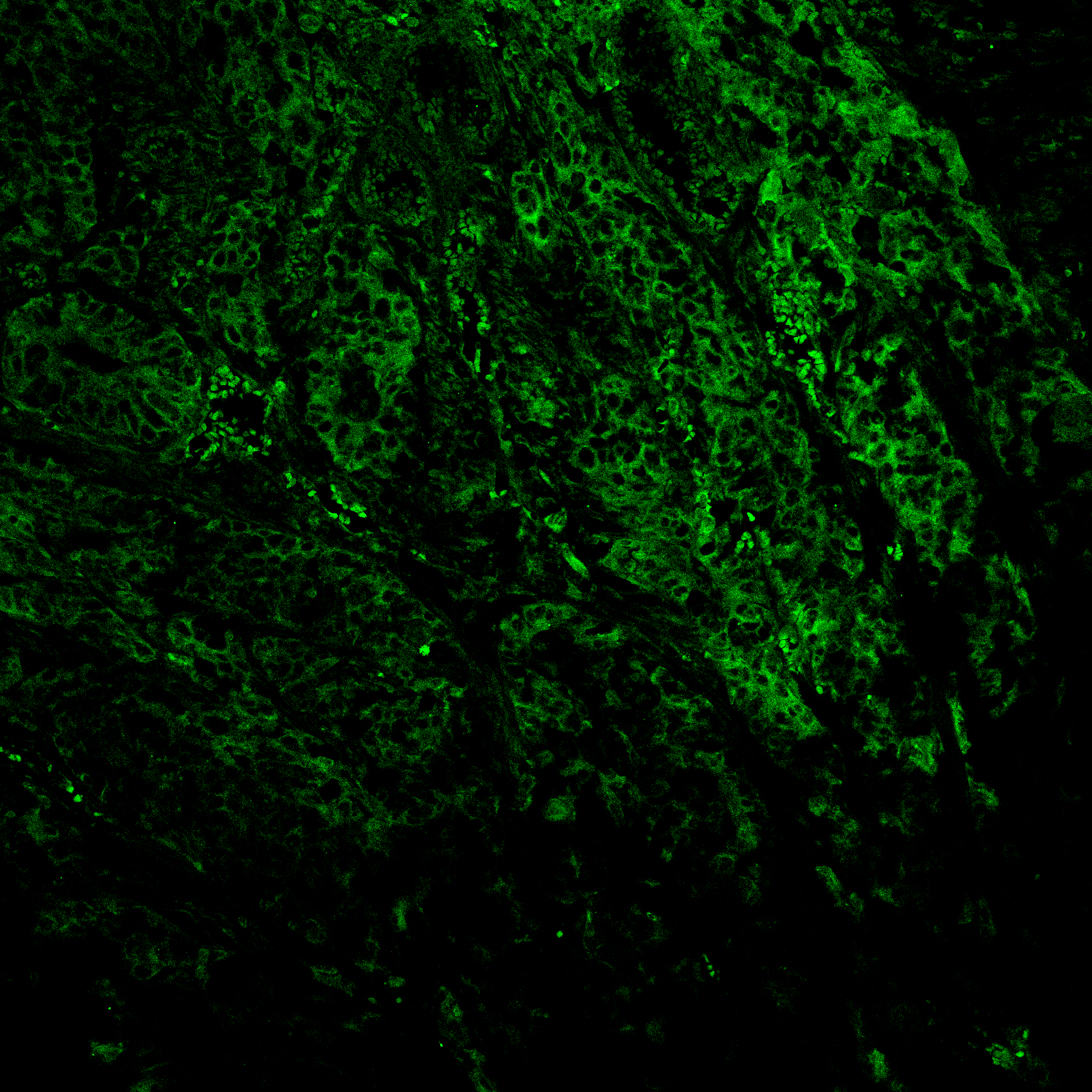

Supplement: Supplementary file 4 — Source data Fig. 1 [file 44321_2025_196_MOESM4_ESM.zip › MM-2024-19448_SourceDataForFig 1/Fig MM-2024-19448_SourceDataForFig 1F/CRC Furin.png]

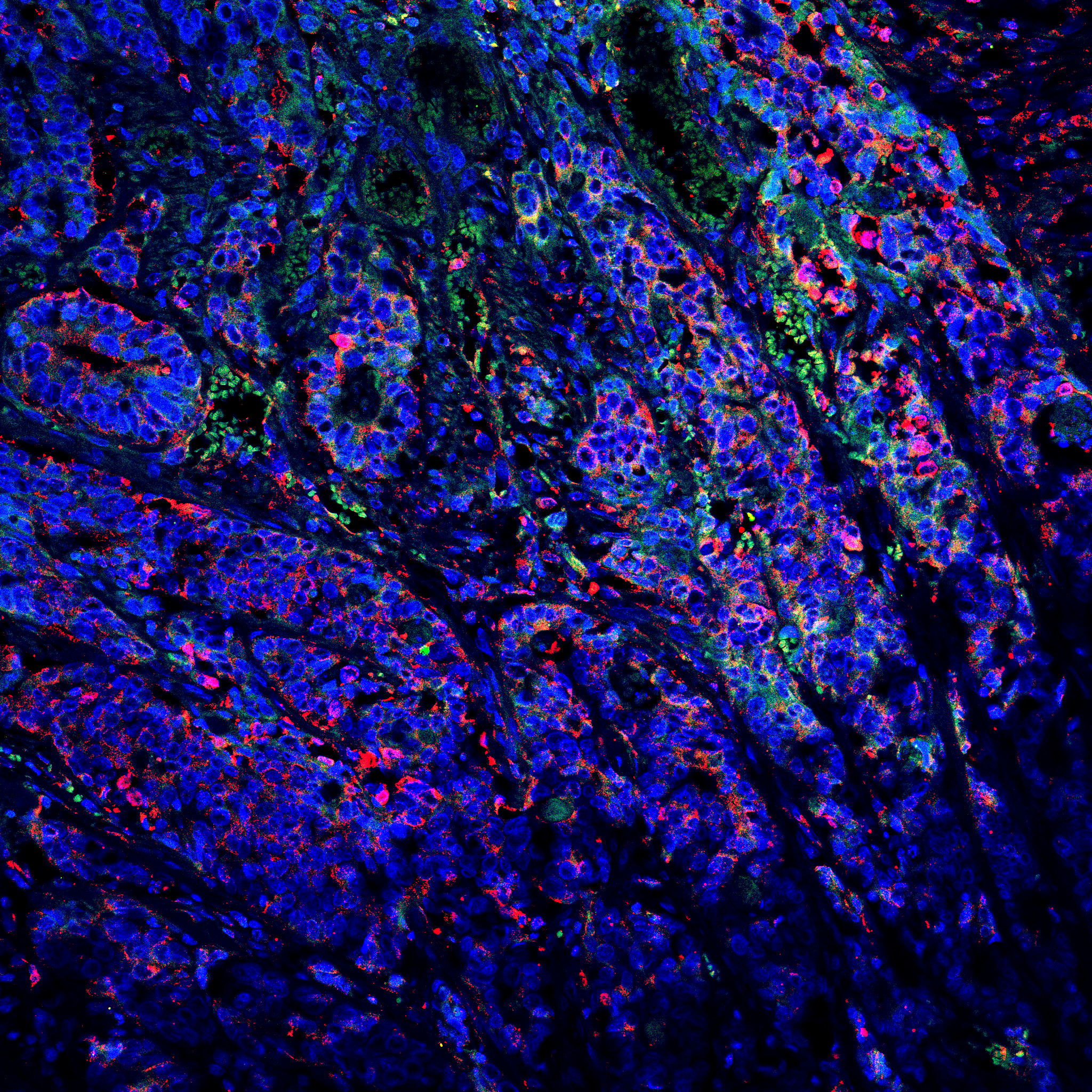

Supplement: Supplementary file 4 — Source data Fig. 1 [file 44321_2025_196_MOESM4_ESM.zip › MM-2024-19448_SourceDataForFig 1/Fig MM-2024-19448_SourceDataForFig 1F/CRC Merge.png]

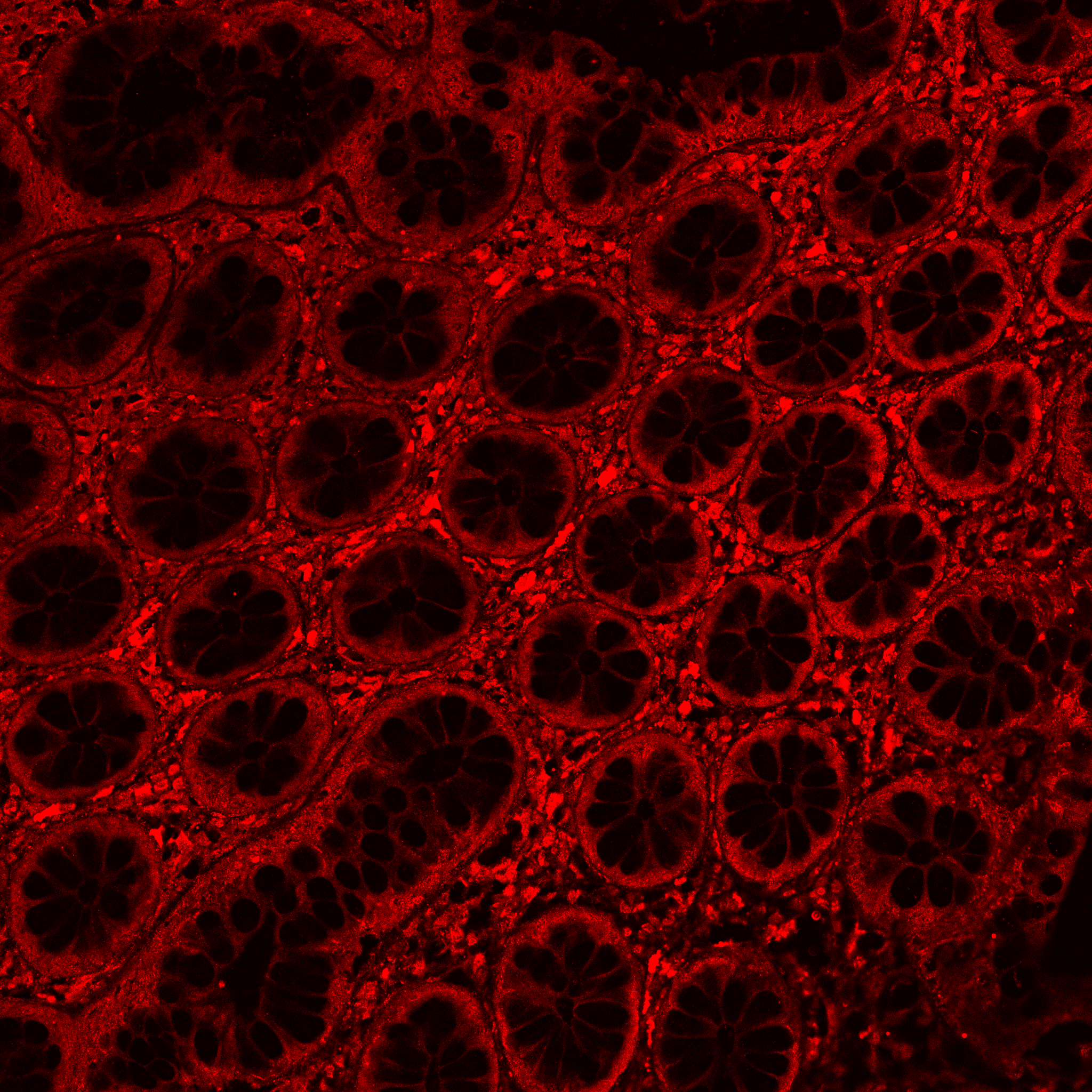

Supplement: Supplementary file 4 — Source data Fig. 1 [file 44321_2025_196_MOESM4_ESM.zip › MM-2024-19448_SourceDataForFig 1/Fig MM-2024-19448_SourceDataForFig 1F/Normal Apelin receptor.png]

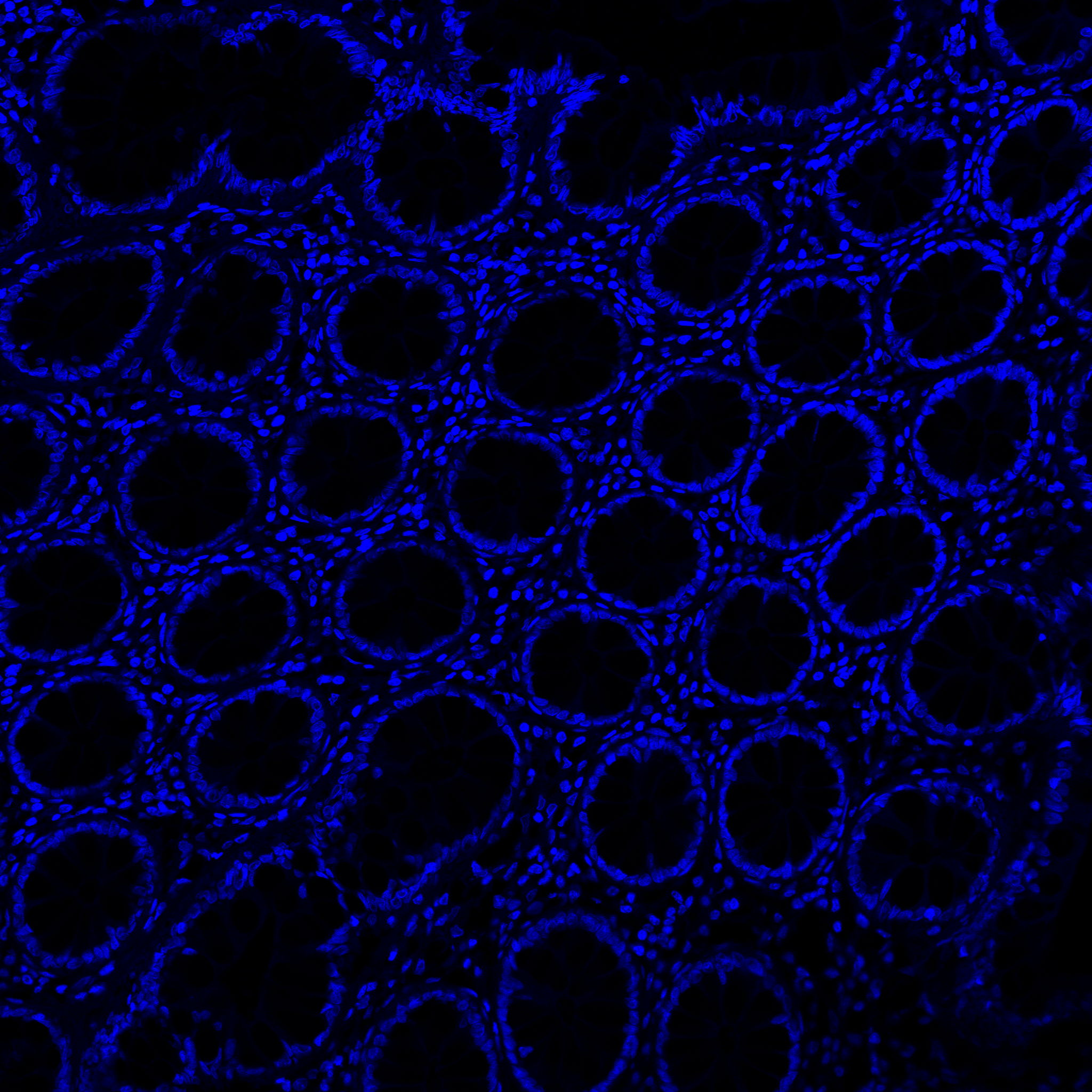

Supplement: Supplementary file 4 — Source data Fig. 1 [file 44321_2025_196_MOESM4_ESM.zip › MM-2024-19448_SourceDataForFig 1/Fig MM-2024-19448_SourceDataForFig 1F/Normal Dapi.png]

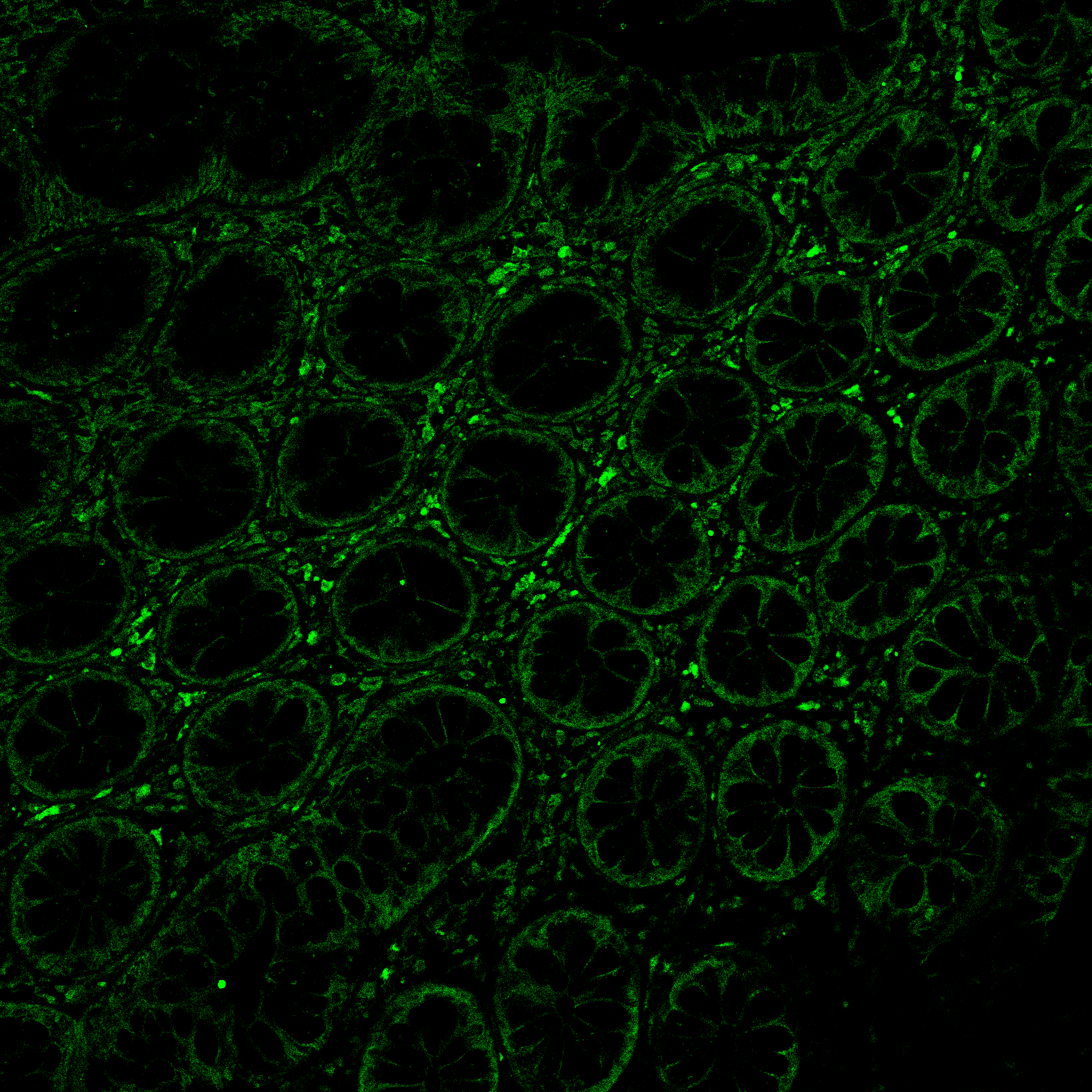

Supplement: Supplementary file 4 — Source data Fig. 1 [file 44321_2025_196_MOESM4_ESM.zip › MM-2024-19448_SourceDataForFig 1/Fig MM-2024-19448_SourceDataForFig 1F/Normal Furin.png]

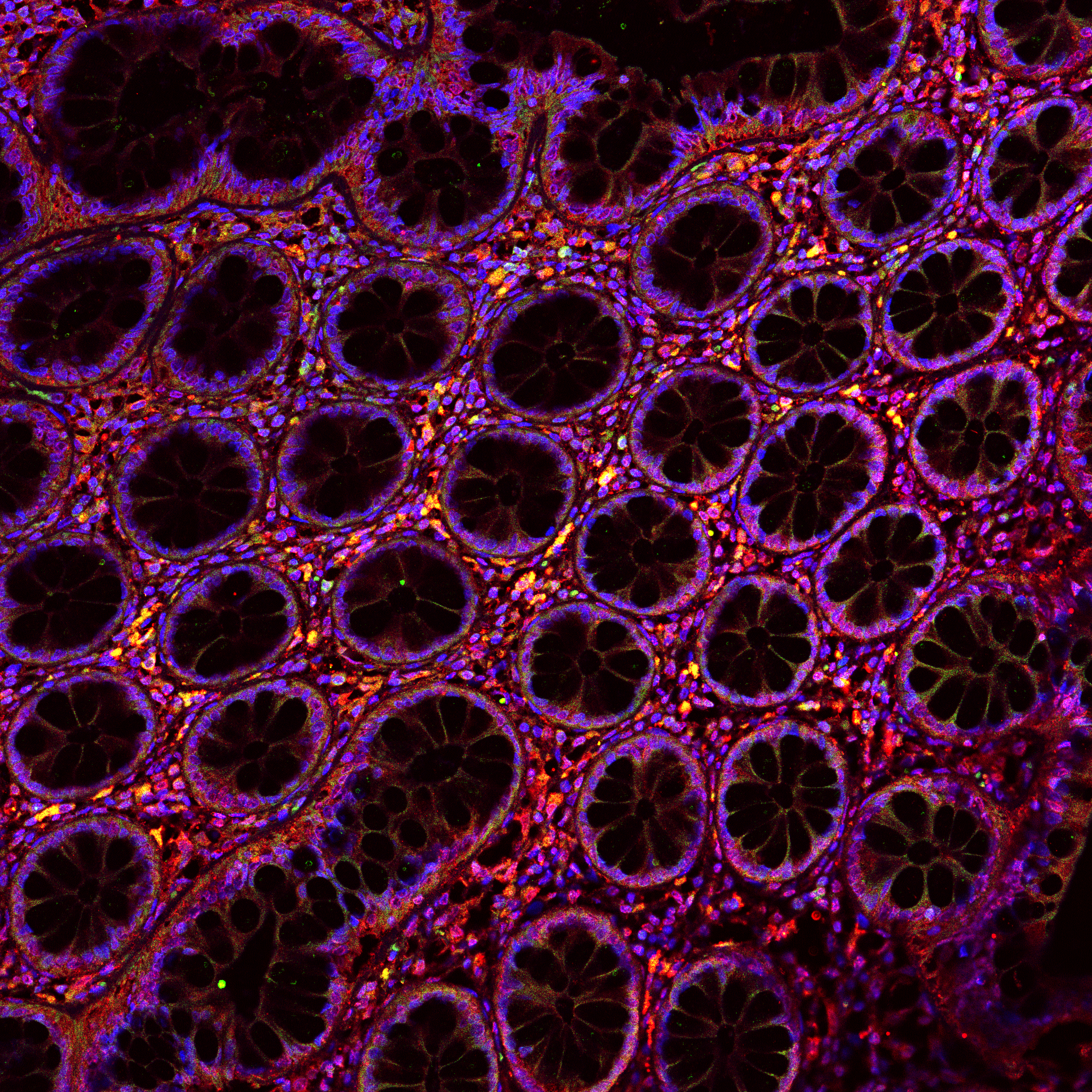

Supplement: Supplementary file 4 — Source data Fig. 1 [file 44321_2025_196_MOESM4_ESM.zip › MM-2024-19448_SourceDataForFig 1/Fig MM-2024-19448_SourceDataForFig 1F/Normal Merge.png]

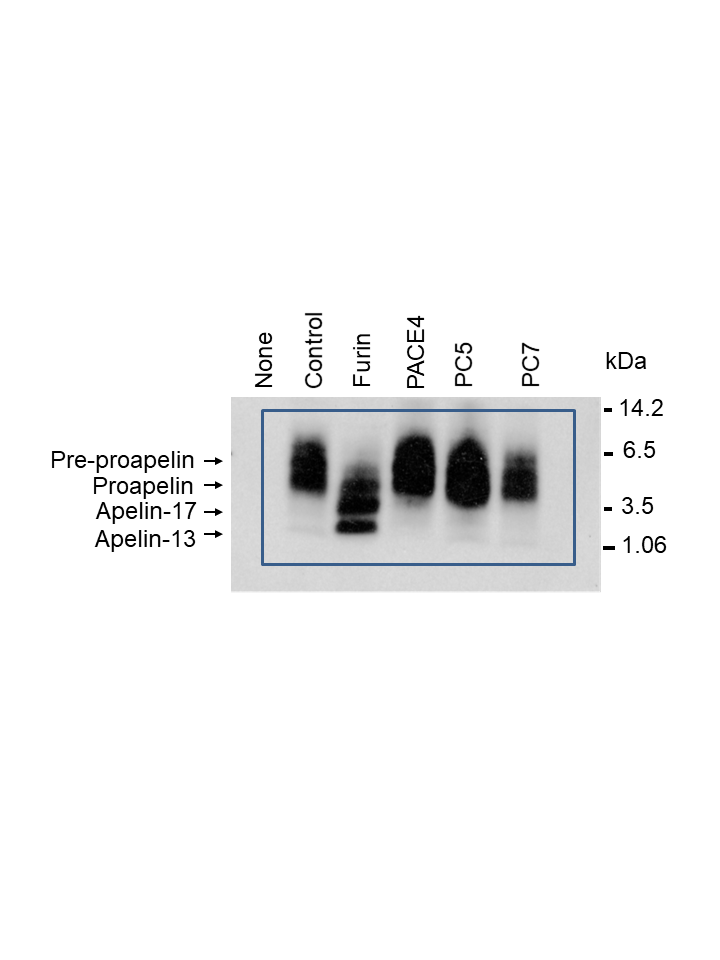

Supplement: Supplementary file 4 — Source data Fig. 1 [file 44321_2025_196_MOESM4_ESM.zip › MM-2024-19448_SourceDataForFig 1/MM-2024-19448_SourceDataForFig 1B.tif]

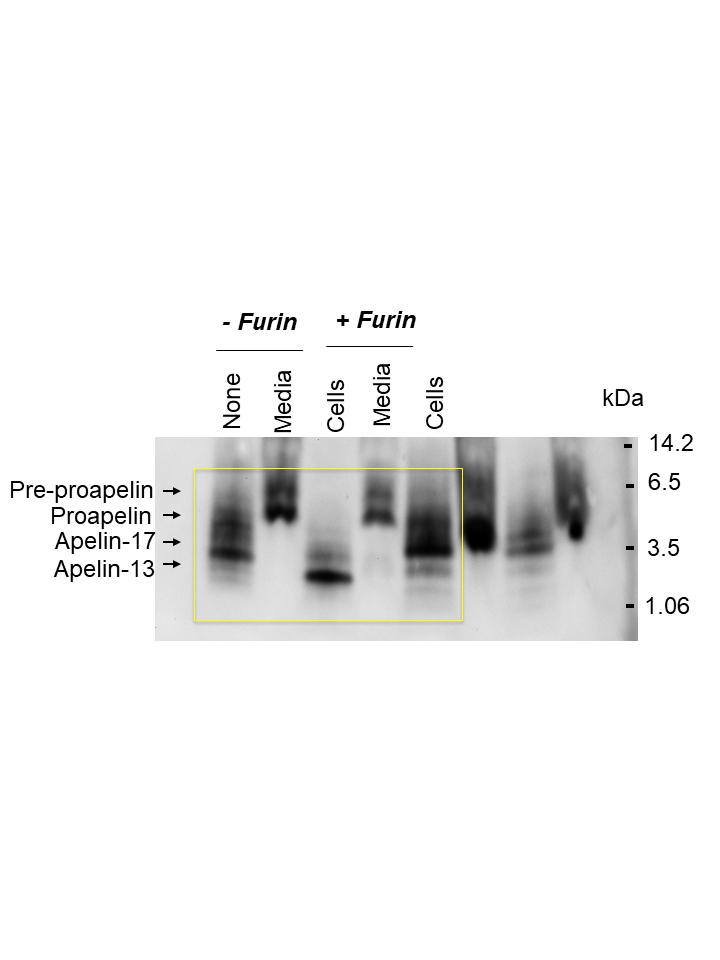

Supplement: Supplementary file 4 — Source data Fig. 1 [file 44321_2025_196_MOESM4_ESM.zip › MM-2024-19448_SourceDataForFig 1/MM-2024-19448_SourceDataForFig 1C.tif]

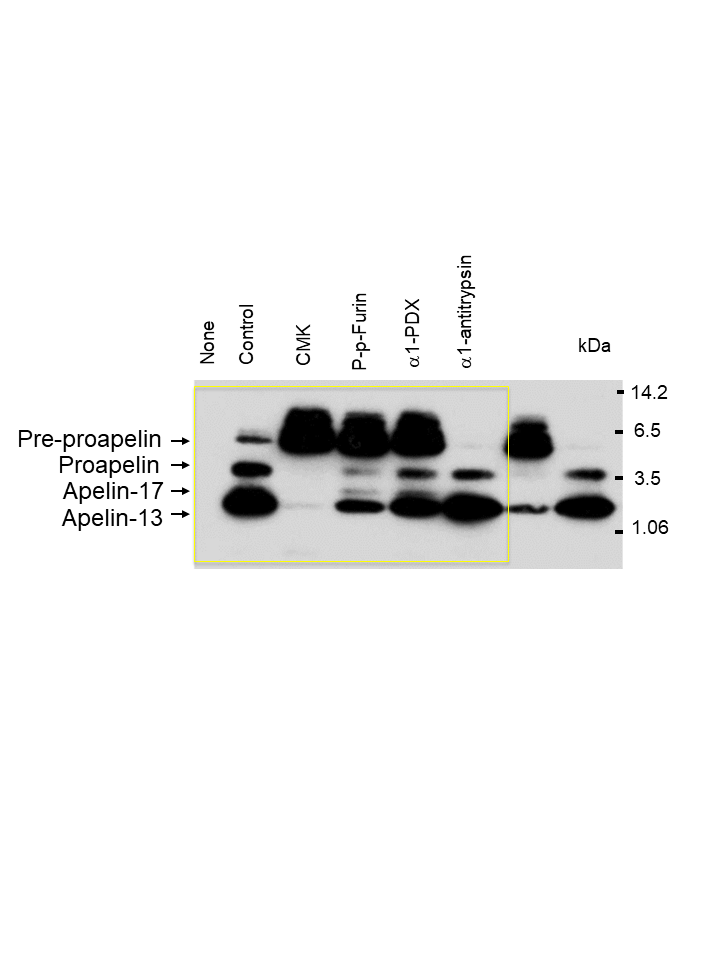

Supplement: Supplementary file 4 — Source data Fig. 1 [file 44321_2025_196_MOESM4_ESM.zip › MM-2024-19448_SourceDataForFig 1/MM-2024-19448_SourceDataForFig 1D.tif]

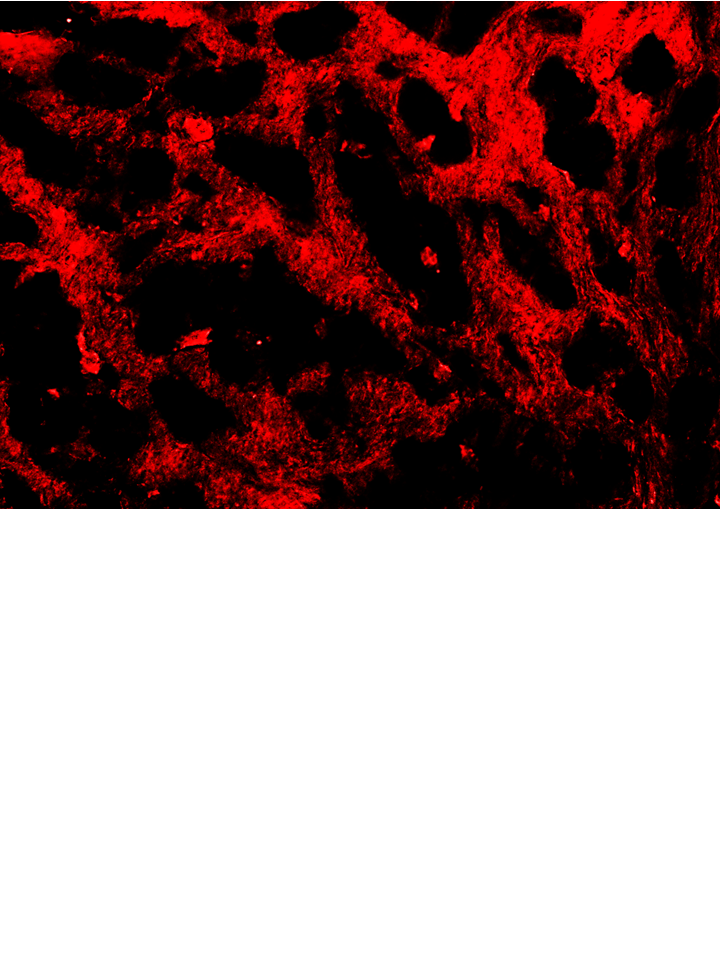

Supplement: Supplementary file 4 — Source data Fig. 1 [file 44321_2025_196_MOESM4_ESM.zip › MM-2024-19448_SourceDataForFig 1/MM-2024-19448_SourceDataForFig 1G/CRC Apelin.TIF]

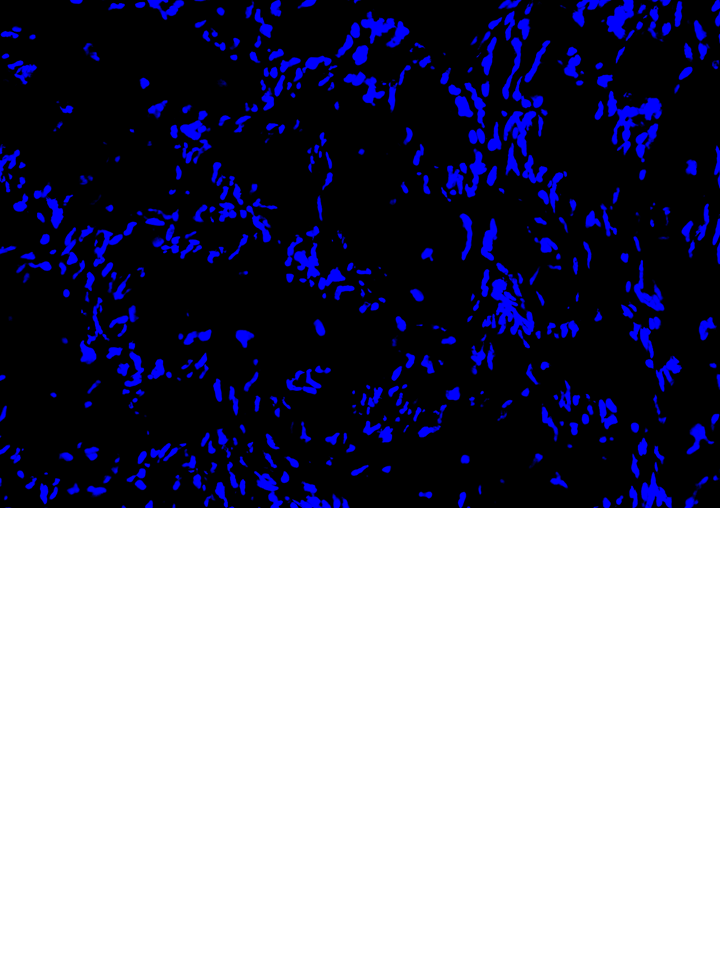

Supplement: Supplementary file 4 — Source data Fig. 1 [file 44321_2025_196_MOESM4_ESM.zip › MM-2024-19448_SourceDataForFig 1/MM-2024-19448_SourceDataForFig 1G/CRC Dapi.TIF]

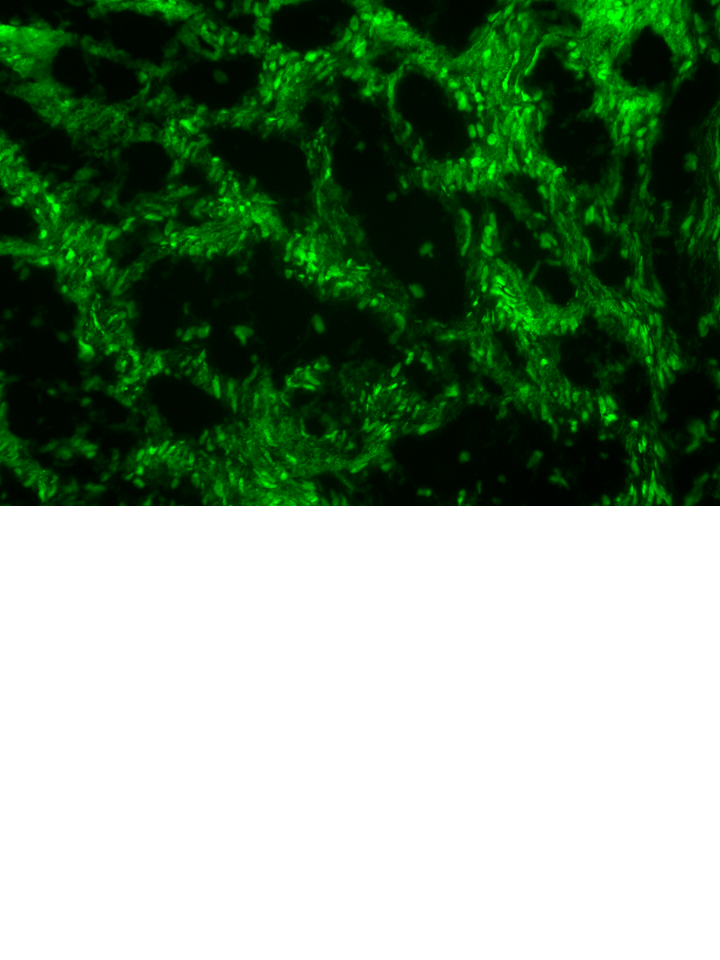

Supplement: Supplementary file 4 — Source data Fig. 1 [file 44321_2025_196_MOESM4_ESM.zip › MM-2024-19448_SourceDataForFig 1/MM-2024-19448_SourceDataForFig 1G/CRC KI-67.TIF]

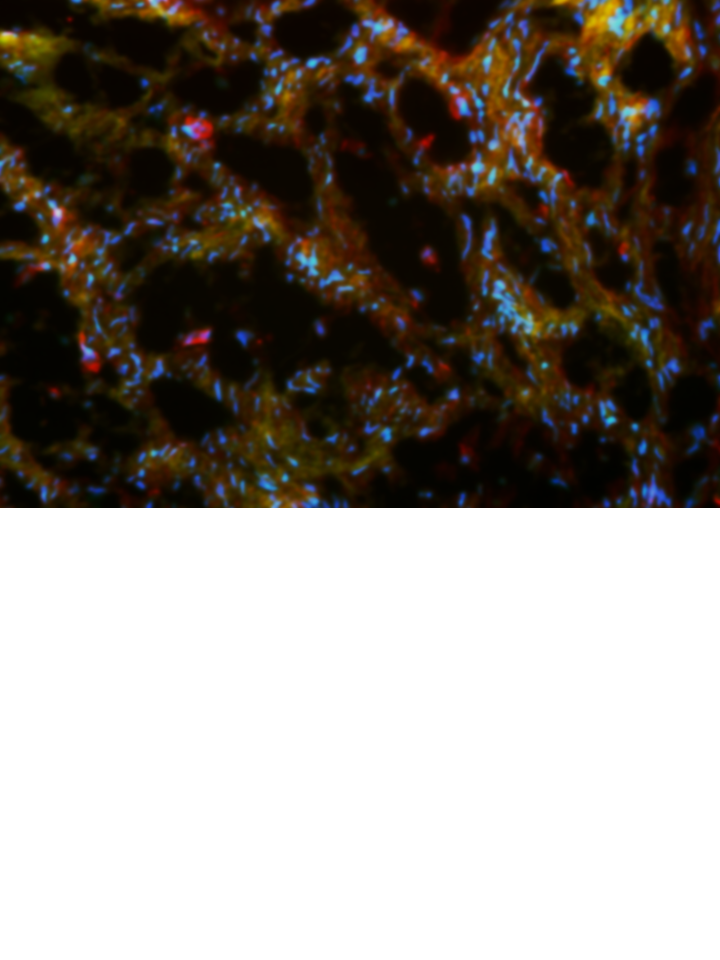

Supplement: Supplementary file 4 — Source data Fig. 1 [file 44321_2025_196_MOESM4_ESM.zip › MM-2024-19448_SourceDataForFig 1/MM-2024-19448_SourceDataForFig 1G/CRC Merge.TIF]

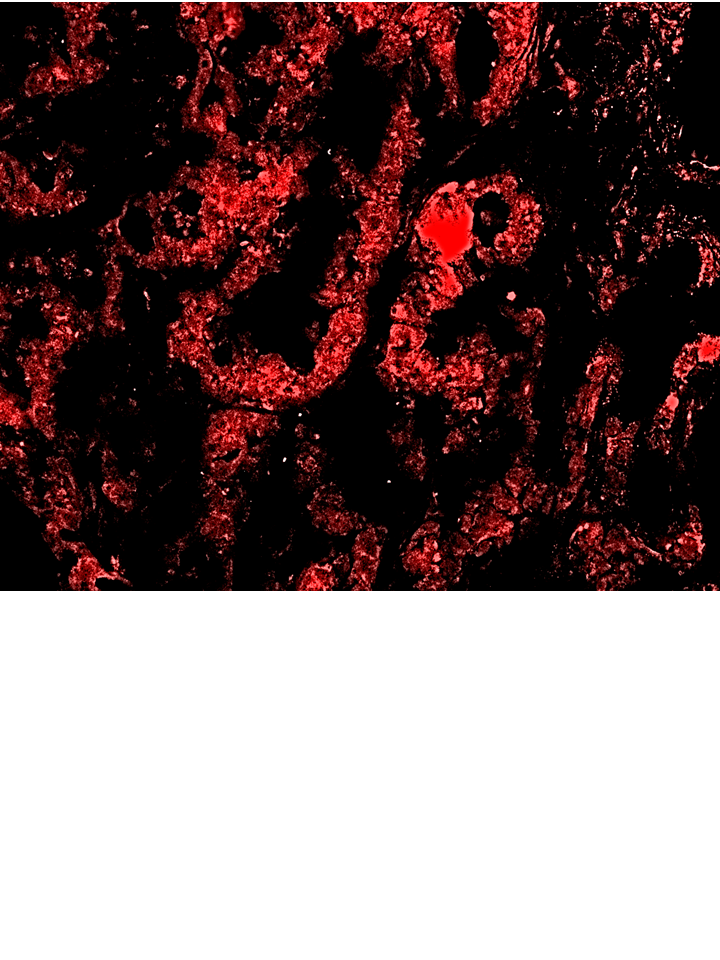

Supplement: Supplementary file 4 — Source data Fig. 1 [file 44321_2025_196_MOESM4_ESM.zip › MM-2024-19448_SourceDataForFig 1/MM-2024-19448_SourceDataForFig 1G/mCRC Apelin.TIF]

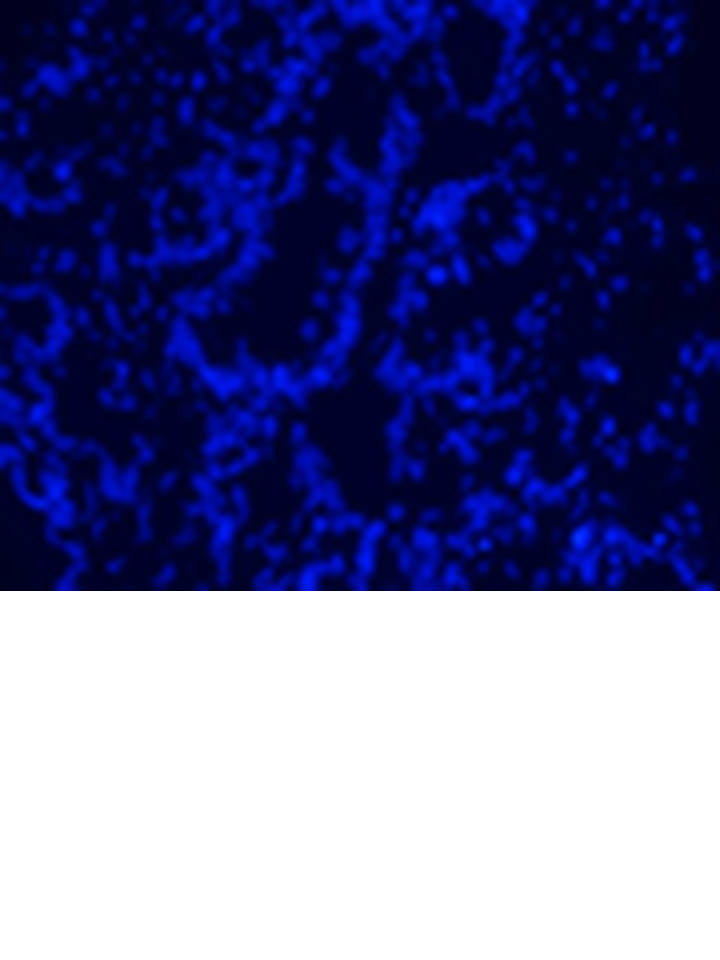

Supplement: Supplementary file 4 — Source data Fig. 1 [file 44321_2025_196_MOESM4_ESM.zip › MM-2024-19448_SourceDataForFig 1/MM-2024-19448_SourceDataForFig 1G/mCRC Dapi.TIF]

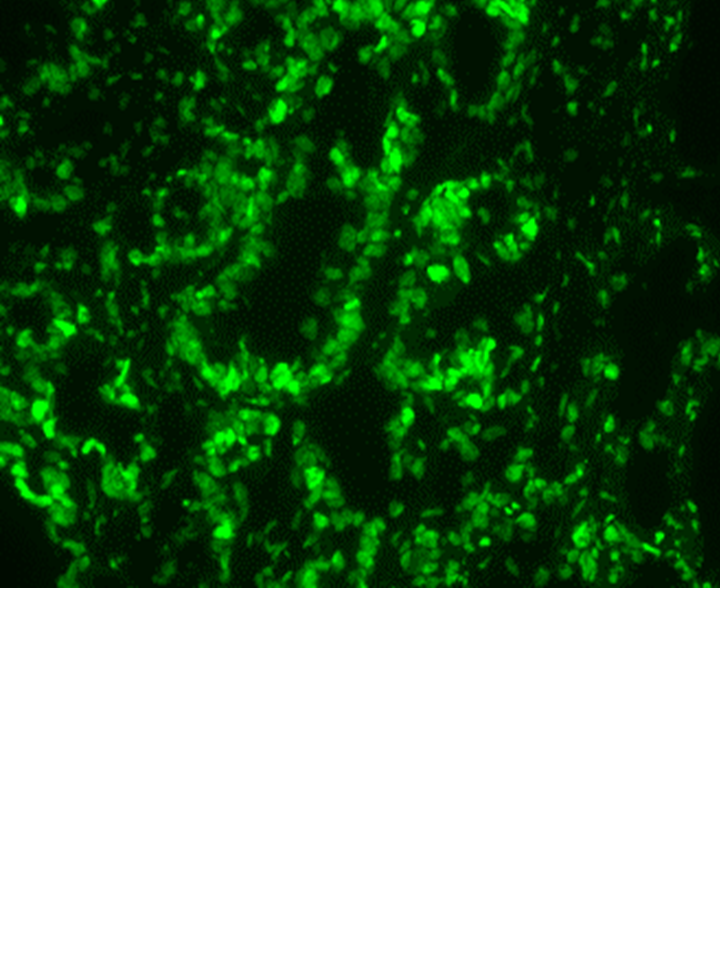

Supplement: Supplementary file 4 — Source data Fig. 1 [file 44321_2025_196_MOESM4_ESM.zip › MM-2024-19448_SourceDataForFig 1/MM-2024-19448_SourceDataForFig 1G/mCRC KI-67.TIF]

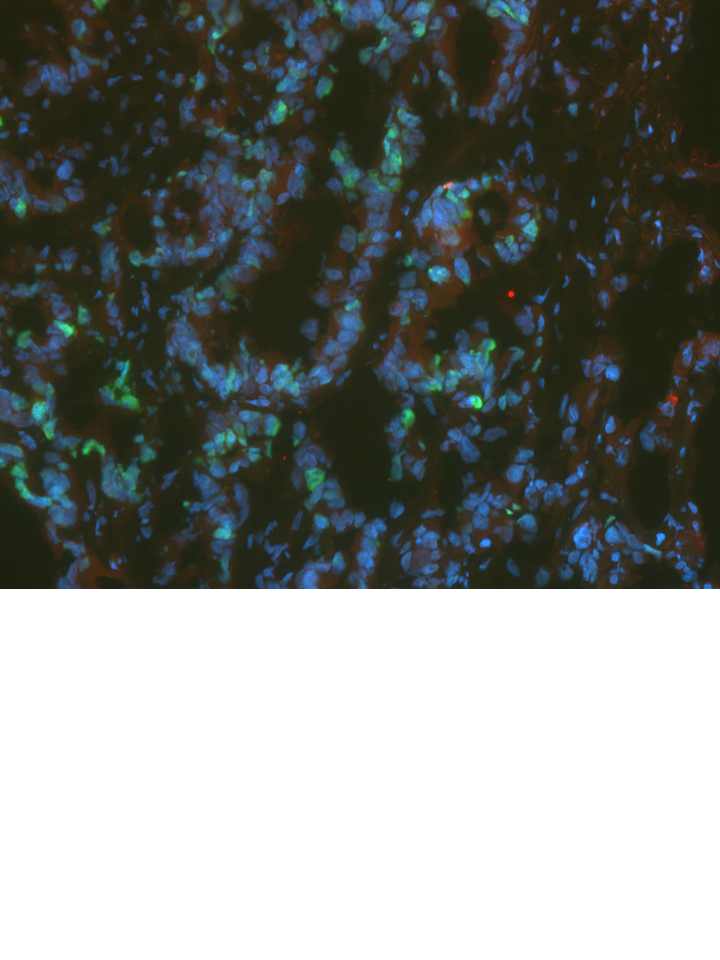

Supplement: Supplementary file 4 — Source data Fig. 1 [file 44321_2025_196_MOESM4_ESM.zip › MM-2024-19448_SourceDataForFig 1/MM-2024-19448_SourceDataForFig 1G/mCRC Merge.TIF]

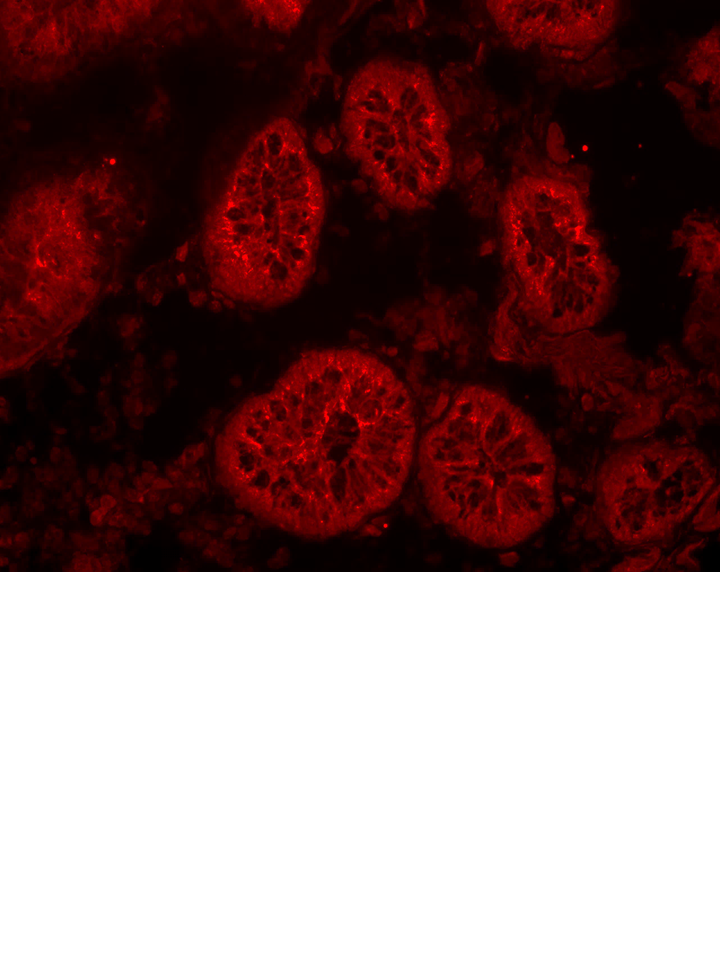

Supplement: Supplementary file 4 — Source data Fig. 1 [file 44321_2025_196_MOESM4_ESM.zip › MM-2024-19448_SourceDataForFig 1/MM-2024-19448_SourceDataForFig 1G/Normal Apelin.TIF]

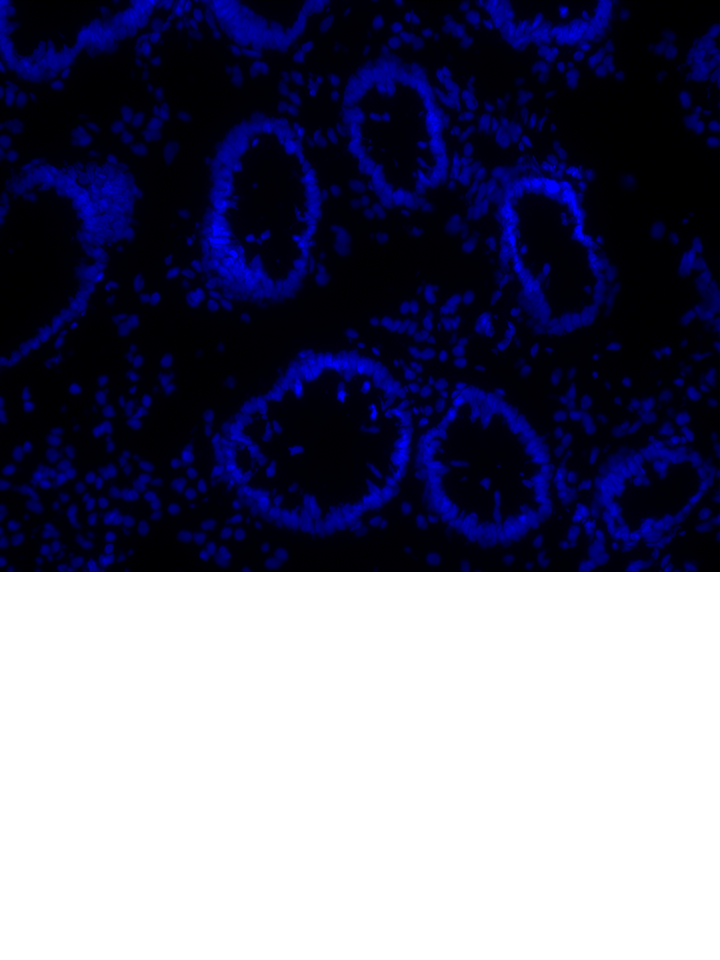

Supplement: Supplementary file 4 — Source data Fig. 1 [file 44321_2025_196_MOESM4_ESM.zip › MM-2024-19448_SourceDataForFig 1/MM-2024-19448_SourceDataForFig 1G/Normal Dapi.TIF]

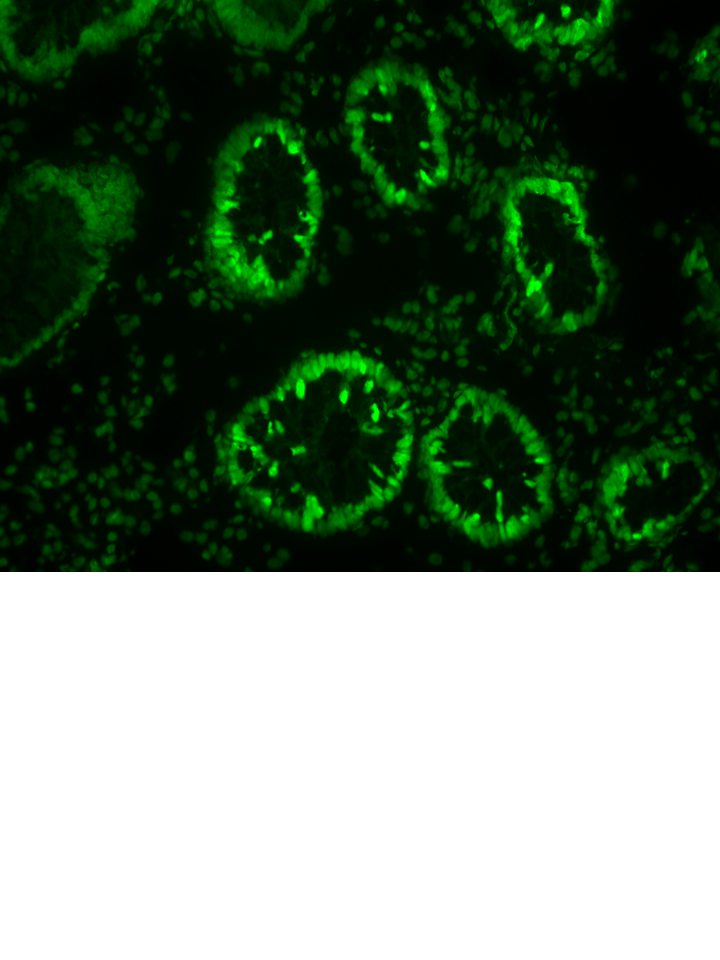

Supplement: Supplementary file 4 — Source data Fig. 1 [file 44321_2025_196_MOESM4_ESM.zip › MM-2024-19448_SourceDataForFig 1/MM-2024-19448_SourceDataForFig 1G/Normal KI67.TIF]

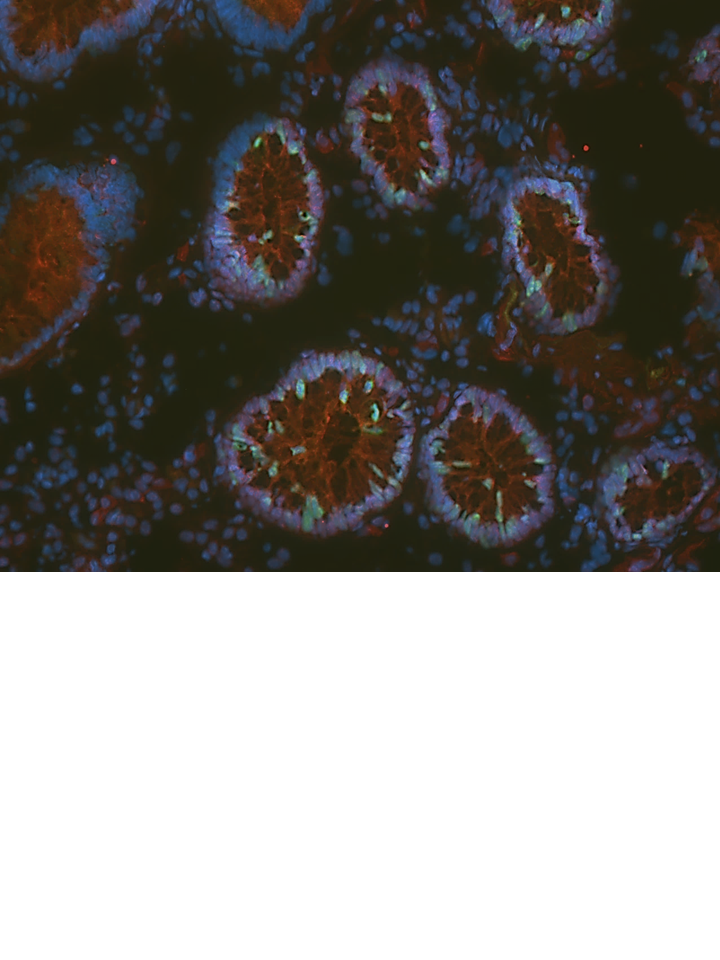

Supplement: Supplementary file 4 — Source data Fig. 1 [file 44321_2025_196_MOESM4_ESM.zip › MM-2024-19448_SourceDataForFig 1/MM-2024-19448_SourceDataForFig 1G/Normal Merge.TIF]

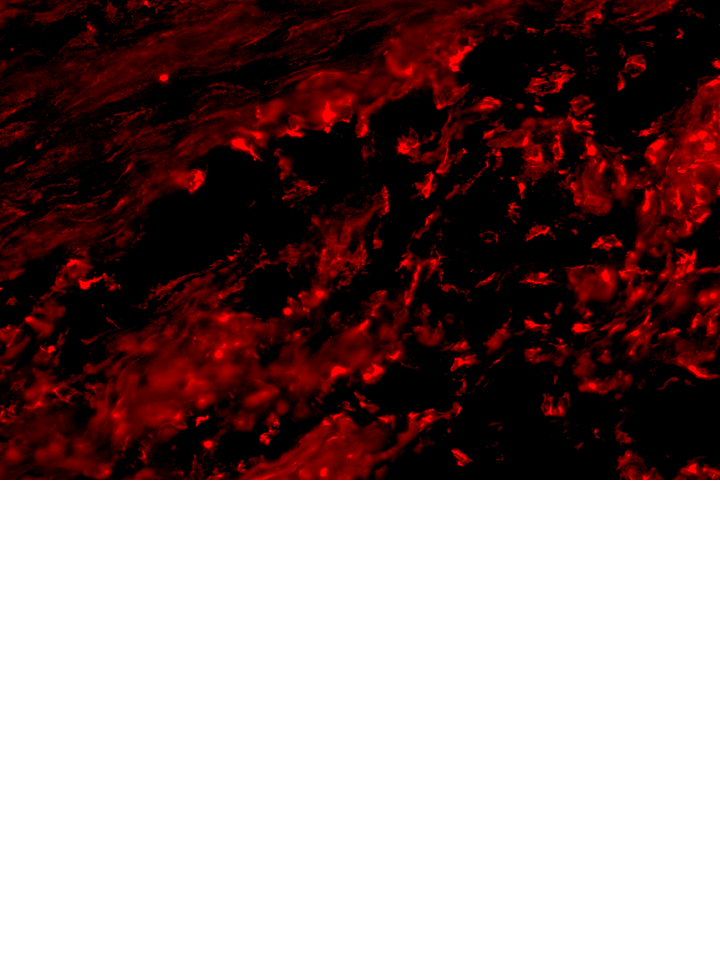

Supplement: Supplementary file 4 — Source data Fig. 1 [file 44321_2025_196_MOESM4_ESM.zip › MM-2024-19448_SourceDataForFig 1/MM-2024-19448_SourceDataForFig 1H/CRC Apelin receptor.TIF]

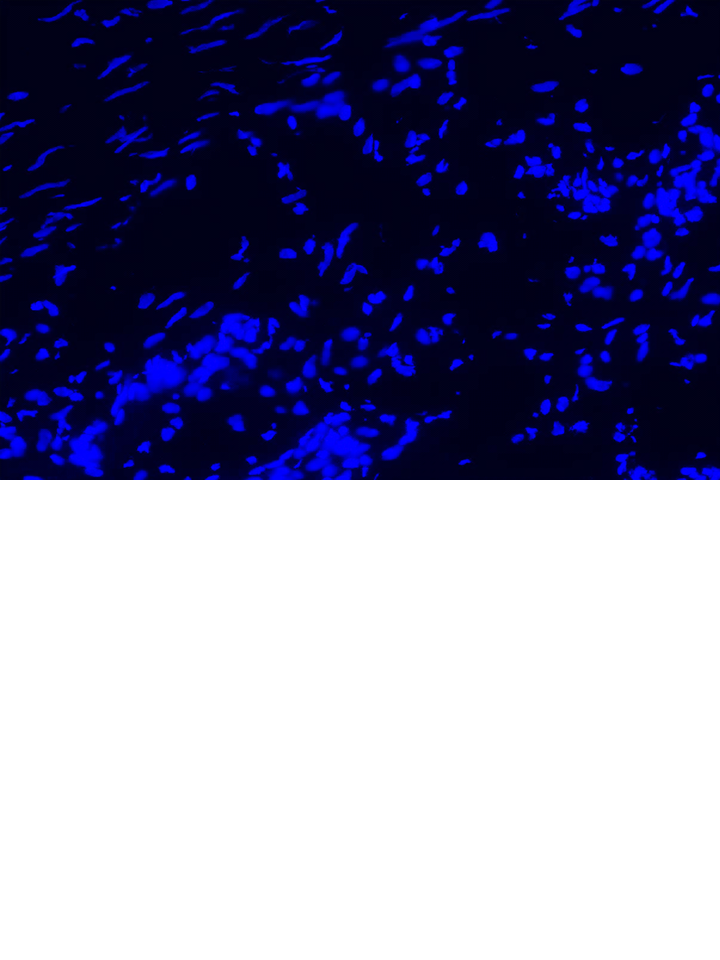

Supplement: Supplementary file 4 — Source data Fig. 1 [file 44321_2025_196_MOESM4_ESM.zip › MM-2024-19448_SourceDataForFig 1/MM-2024-19448_SourceDataForFig 1H/CRC Dapi.TIF]

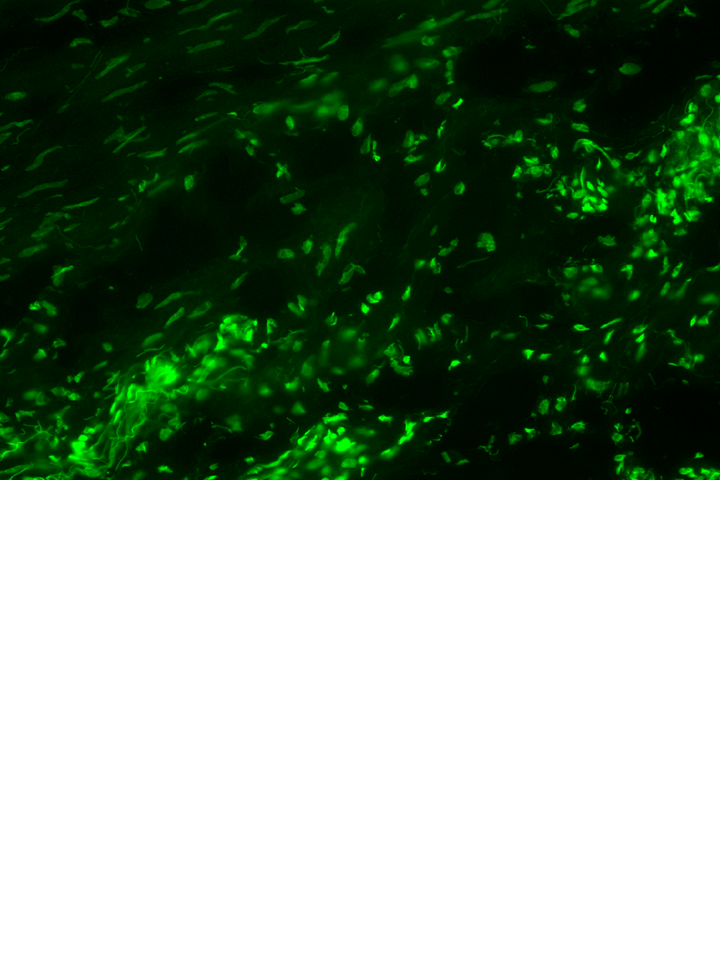

Supplement: Supplementary file 4 — Source data Fig. 1 [file 44321_2025_196_MOESM4_ESM.zip › MM-2024-19448_SourceDataForFig 1/MM-2024-19448_SourceDataForFig 1H/CRC Furin.TIF]

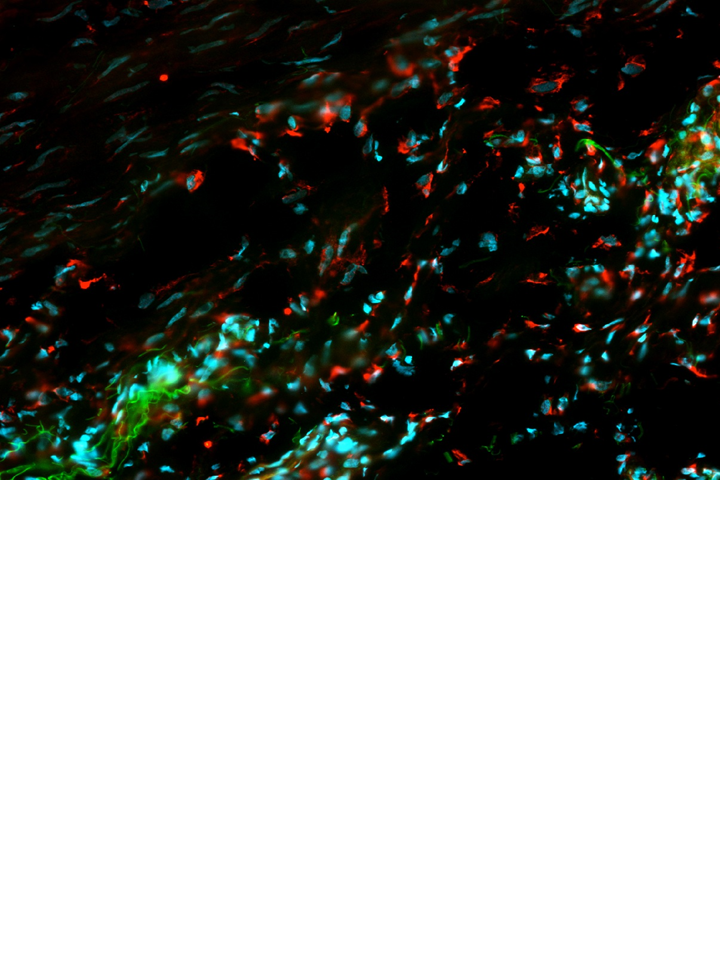

Supplement: Supplementary file 4 — Source data Fig. 1 [file 44321_2025_196_MOESM4_ESM.zip › MM-2024-19448_SourceDataForFig 1/MM-2024-19448_SourceDataForFig 1H/CRC Merge.TIF]

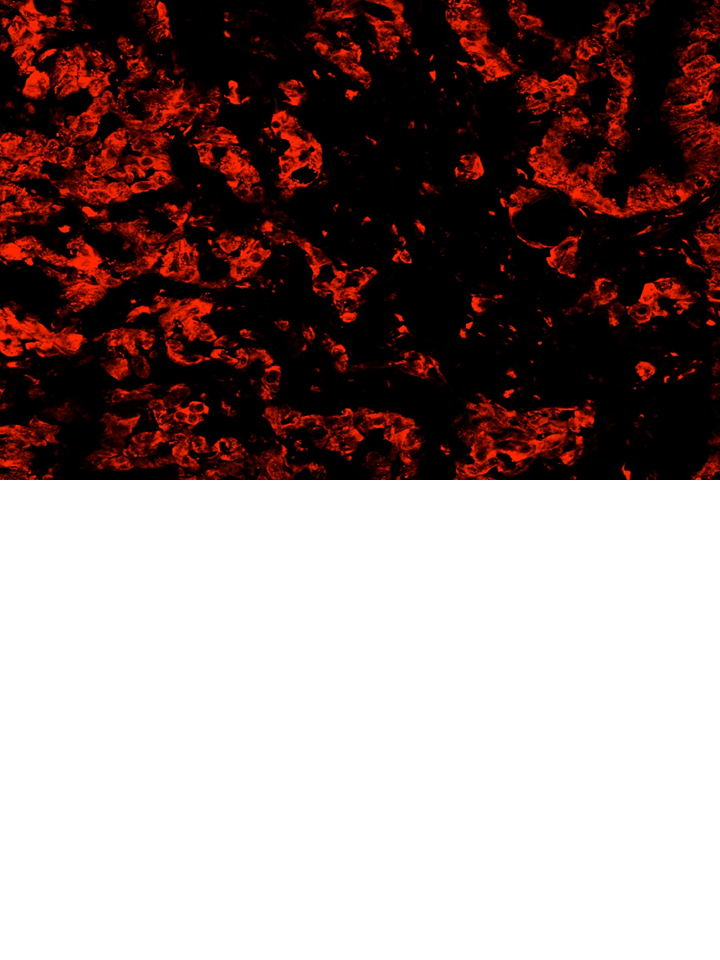

Supplement: Supplementary file 4 — Source data Fig. 1 [file 44321_2025_196_MOESM4_ESM.zip › MM-2024-19448_SourceDataForFig 1/MM-2024-19448_SourceDataForFig 1H/mCRC Apelin receptor.TIF]

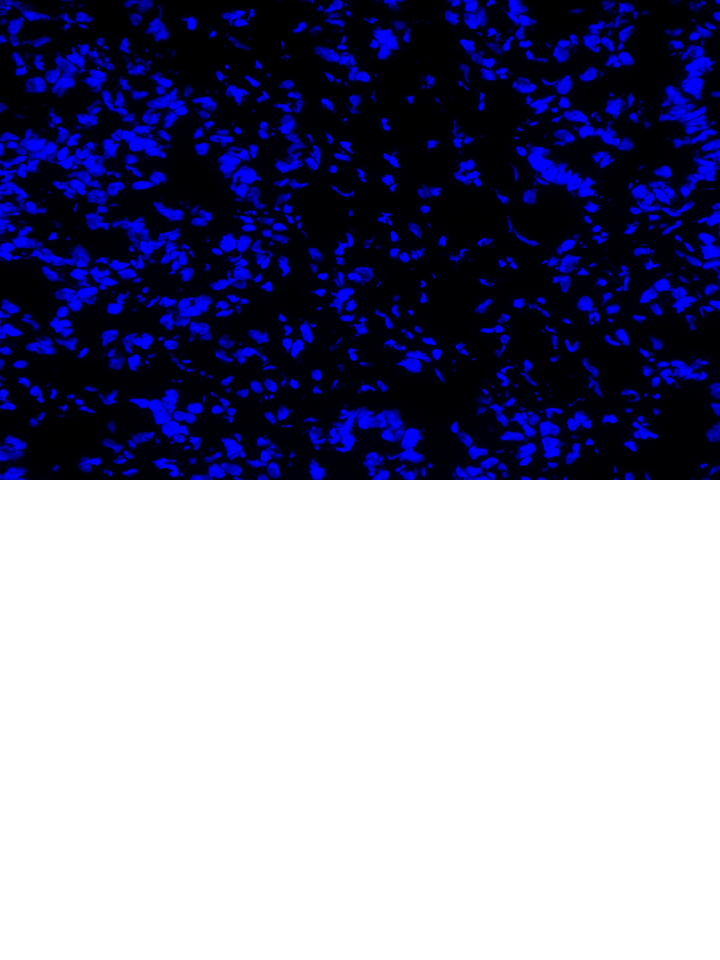

Supplement: Supplementary file 4 — Source data Fig. 1 [file 44321_2025_196_MOESM4_ESM.zip › MM-2024-19448_SourceDataForFig 1/MM-2024-19448_SourceDataForFig 1H/mCRC Dapi.TIF]

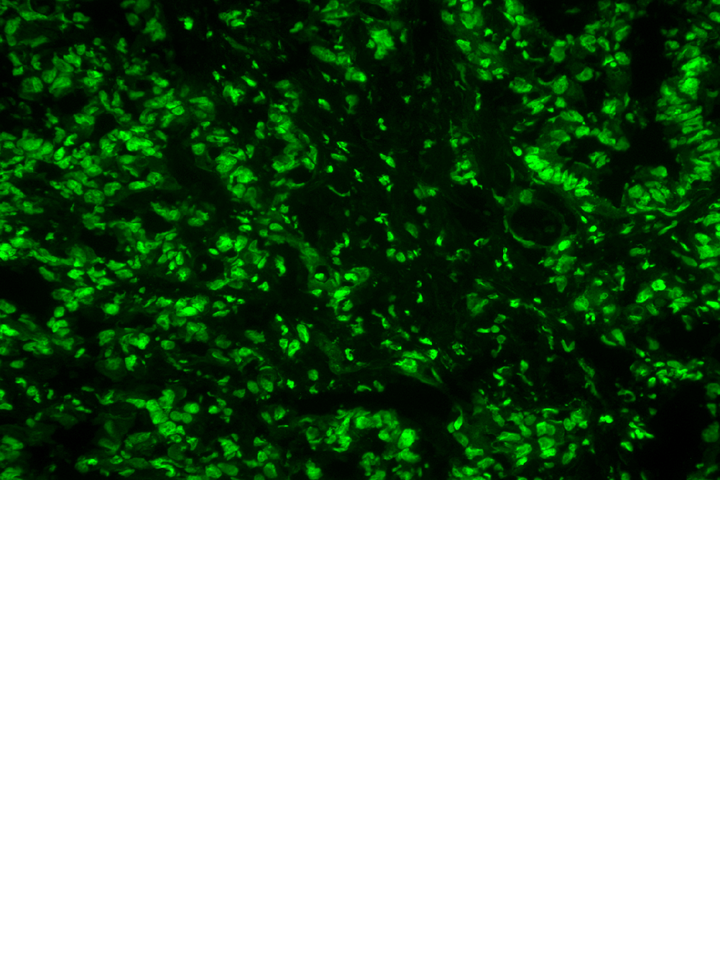

Supplement: Supplementary file 4 — Source data Fig. 1 [file 44321_2025_196_MOESM4_ESM.zip › MM-2024-19448_SourceDataForFig 1/MM-2024-19448_SourceDataForFig 1H/mCRC Furin.TIF]

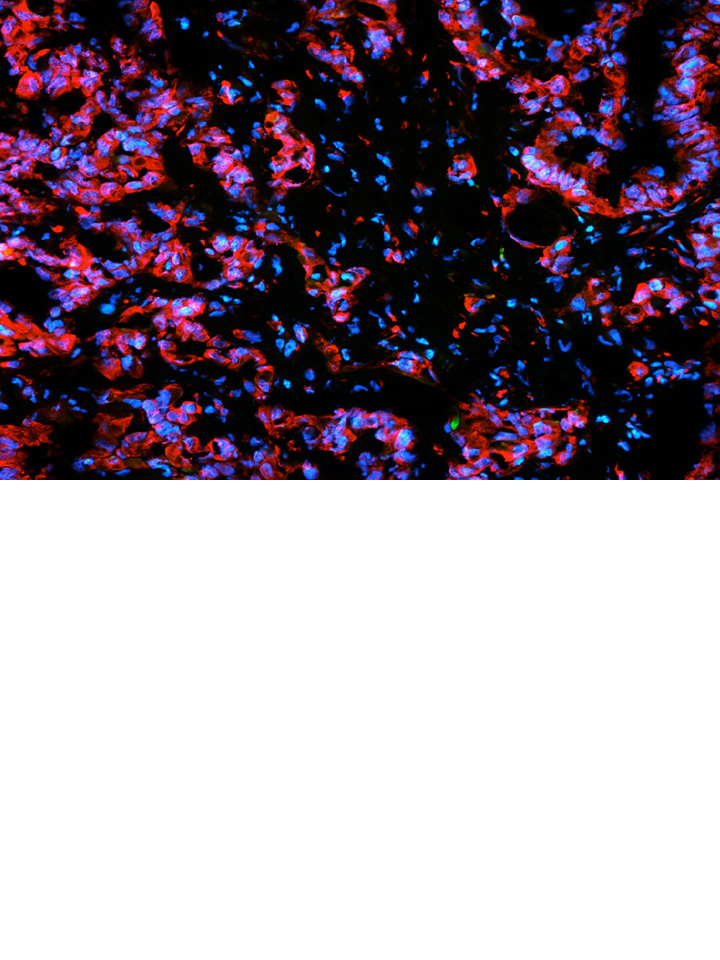

Supplement: Supplementary file 4 — Source data Fig. 1 [file 44321_2025_196_MOESM4_ESM.zip › MM-2024-19448_SourceDataForFig 1/MM-2024-19448_SourceDataForFig 1H/mCRC Merge.TIF]

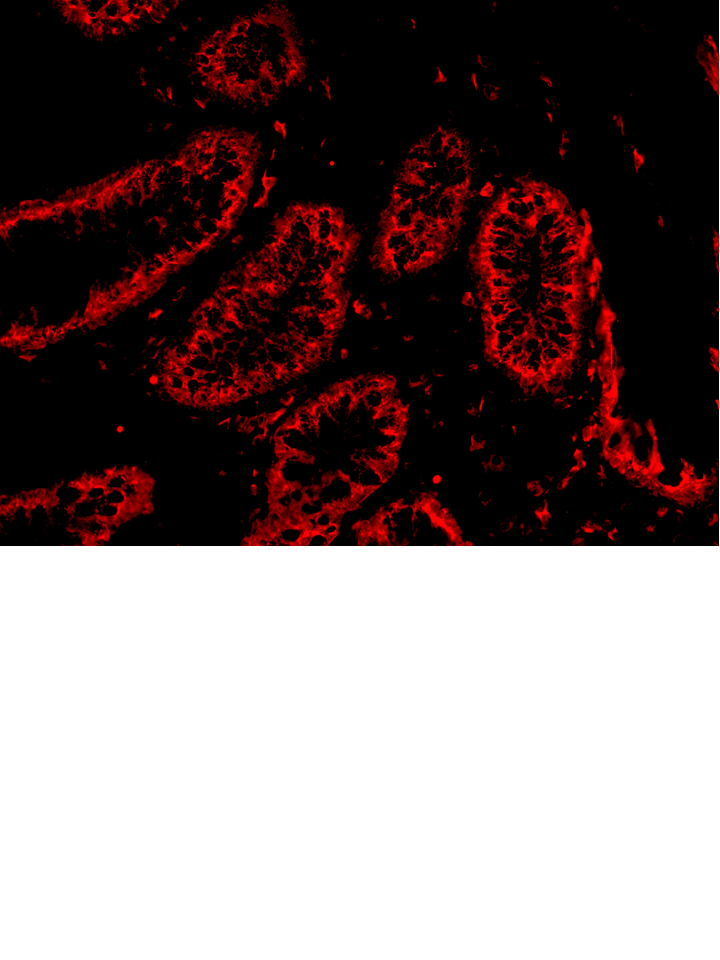

Supplement: Supplementary file 4 — Source data Fig. 1 [file 44321_2025_196_MOESM4_ESM.zip › MM-2024-19448_SourceDataForFig 1/MM-2024-19448_SourceDataForFig 1H/Normal Apelin Receptor.TIF]

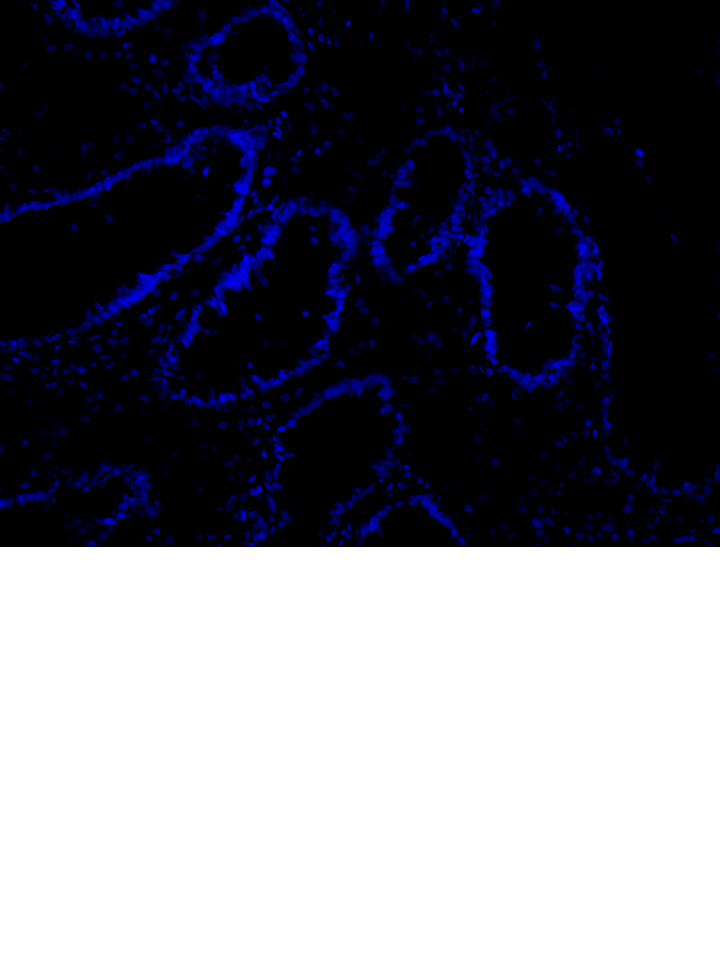

Supplement: Supplementary file 4 — Source data Fig. 1 [file 44321_2025_196_MOESM4_ESM.zip › MM-2024-19448_SourceDataForFig 1/MM-2024-19448_SourceDataForFig 1H/Normal Dapi.TIF]

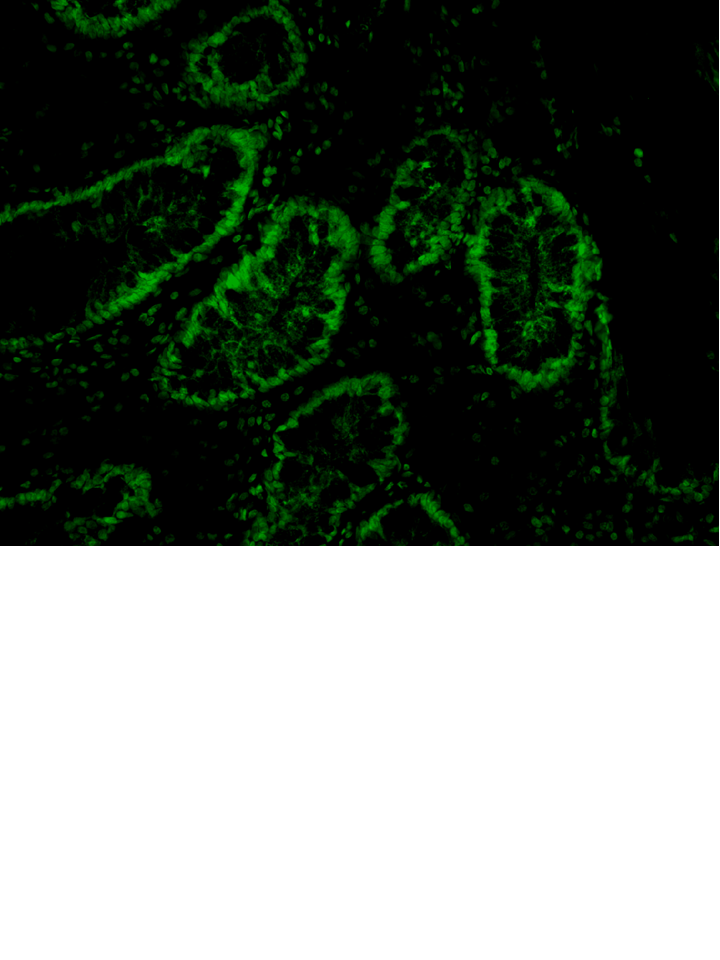

Supplement: Supplementary file 4 — Source data Fig. 1 [file 44321_2025_196_MOESM4_ESM.zip › MM-2024-19448_SourceDataForFig 1/MM-2024-19448_SourceDataForFig 1H/Normal Furin.TIF]

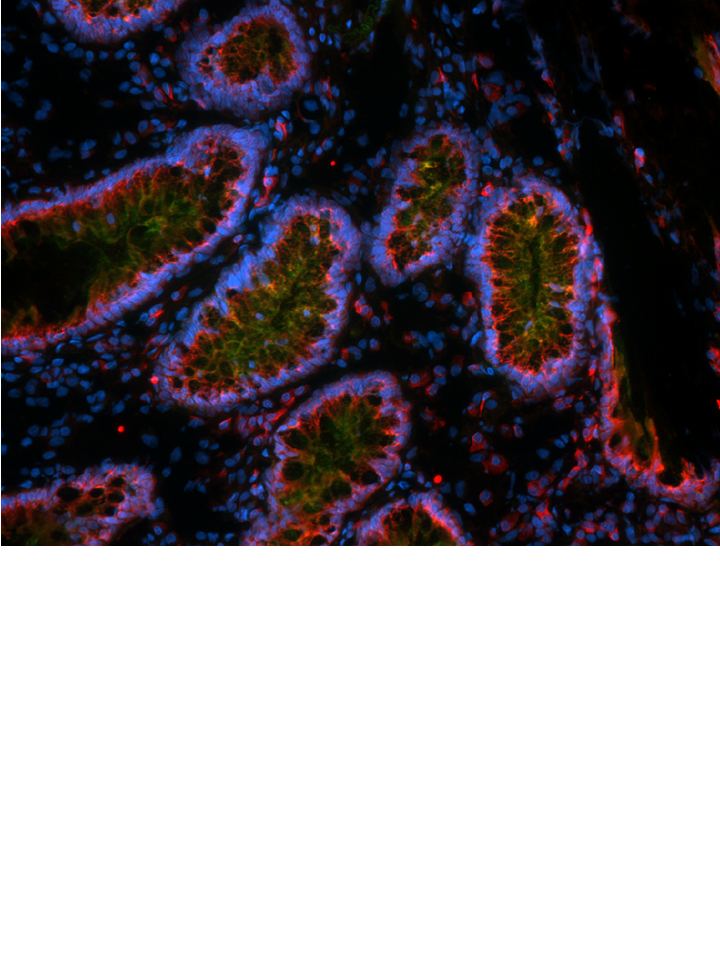

Supplement: Supplementary file 4 — Source data Fig. 1 [file 44321_2025_196_MOESM4_ESM.zip › MM-2024-19448_SourceDataForFig 1/MM-2024-19448_SourceDataForFig 1H/Normal Merge.TIF]

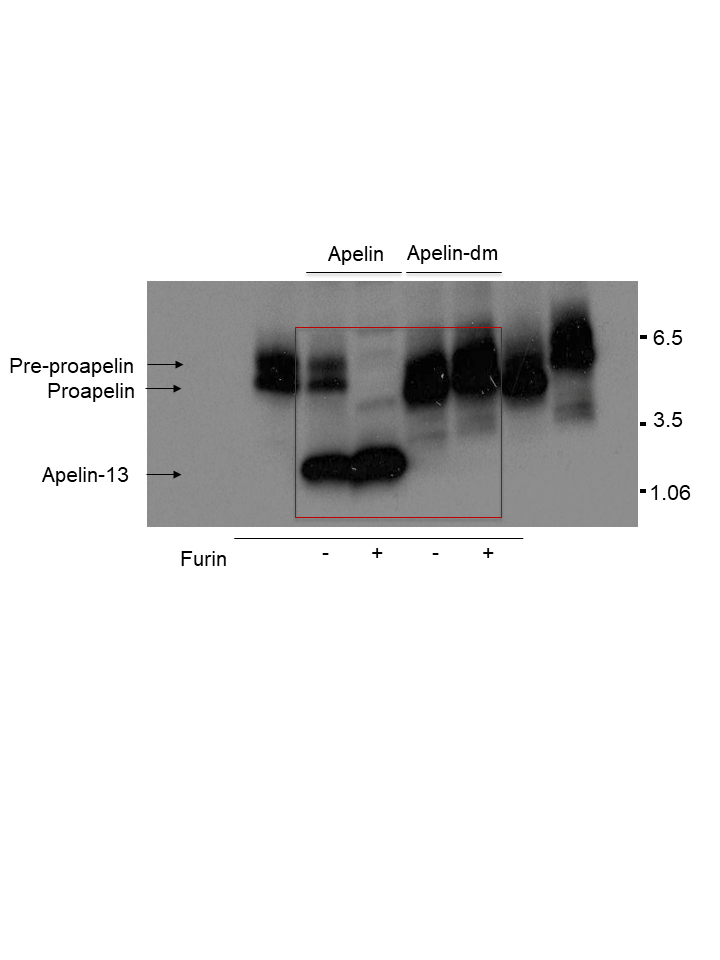

Supplement: Supplementary file 5 — Source data Fig. 2 [file 44321_2025_196_MOESM5_ESM.zip › MM-2024-19448_SourceDataForFig 2/MM-2024-19448_SourceDataForFig 2A.tif]

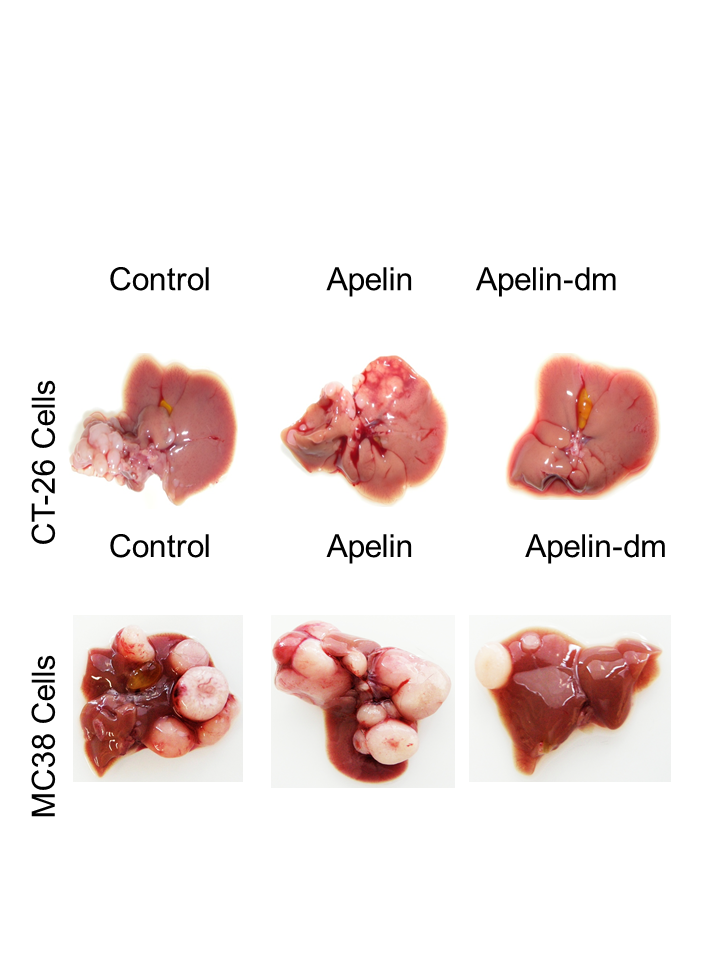

Supplement: Supplementary file 5 — Source data Fig. 2 [file 44321_2025_196_MOESM5_ESM.zip › MM-2024-19448_SourceDataForFig 2/MM-2024-19448_SourceDataForFig 2F.tif]

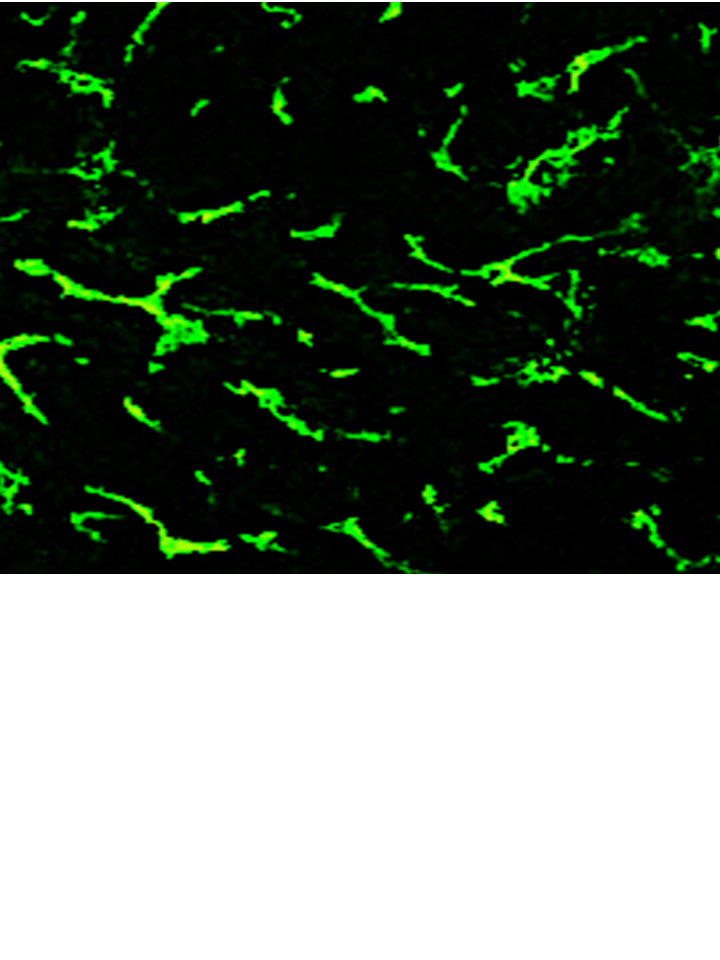

Supplement: Supplementary file 5 — Source data Fig. 2 [file 44321_2025_196_MOESM5_ESM.zip › MM-2024-19448_SourceDataForFig 2/MM-2024-19448_SourceDataForFig 2H/Apelin CD31.TIF]

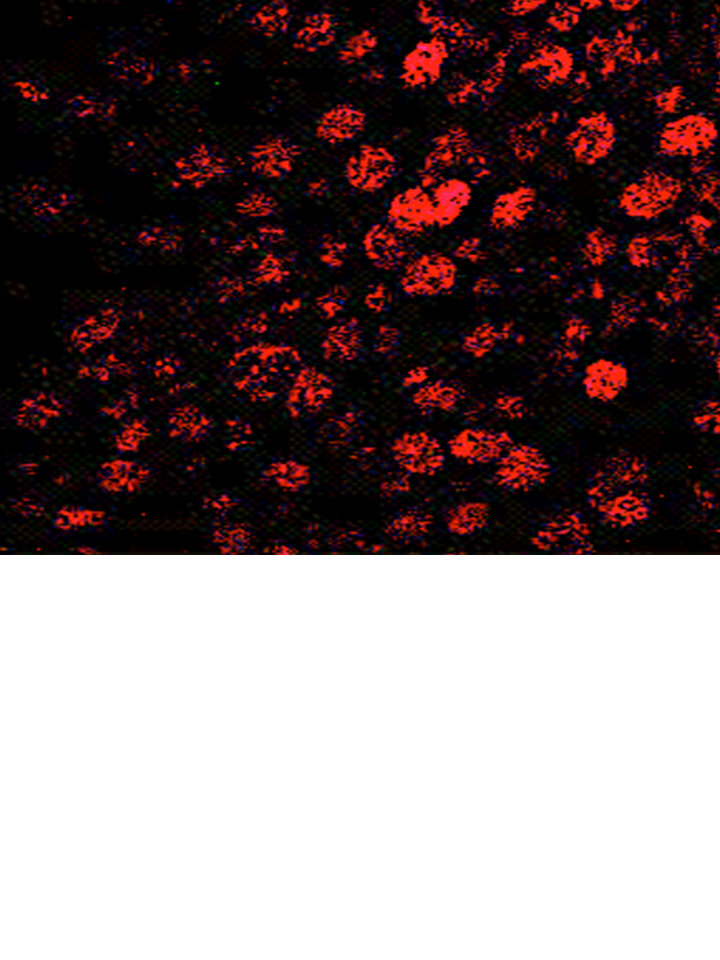

Supplement: Supplementary file 5 — Source data Fig. 2 [file 44321_2025_196_MOESM5_ESM.zip › MM-2024-19448_SourceDataForFig 2/MM-2024-19448_SourceDataForFig 2H/Apelin KI-67.TIF]

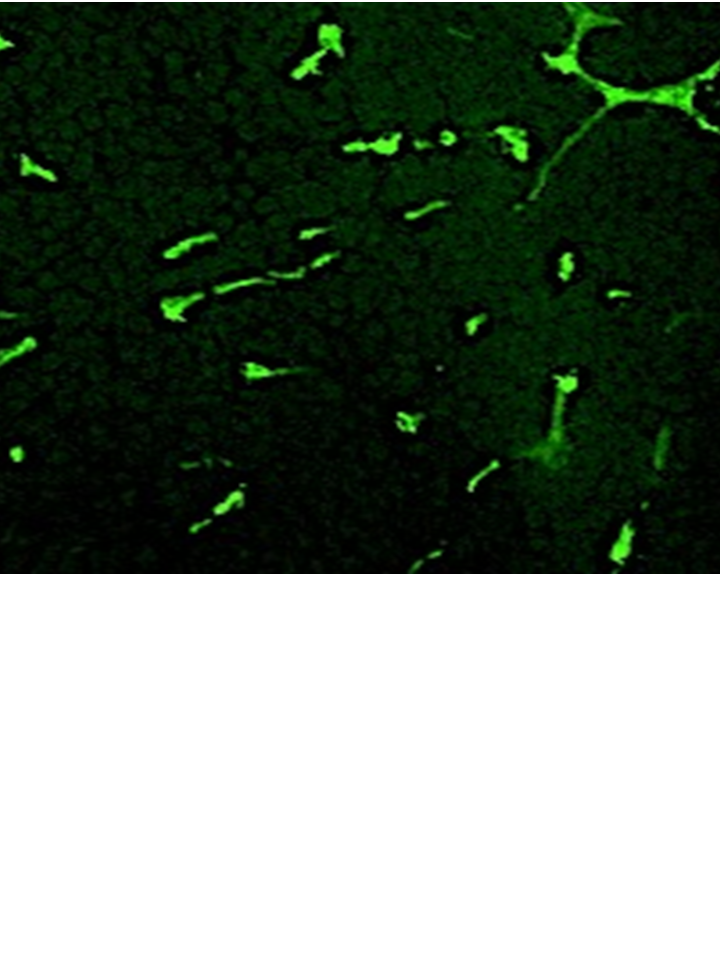

Supplement: Supplementary file 5 — Source data Fig. 2 [file 44321_2025_196_MOESM5_ESM.zip › MM-2024-19448_SourceDataForFig 2/MM-2024-19448_SourceDataForFig 2H/Apelin-dm CD31.TIF]

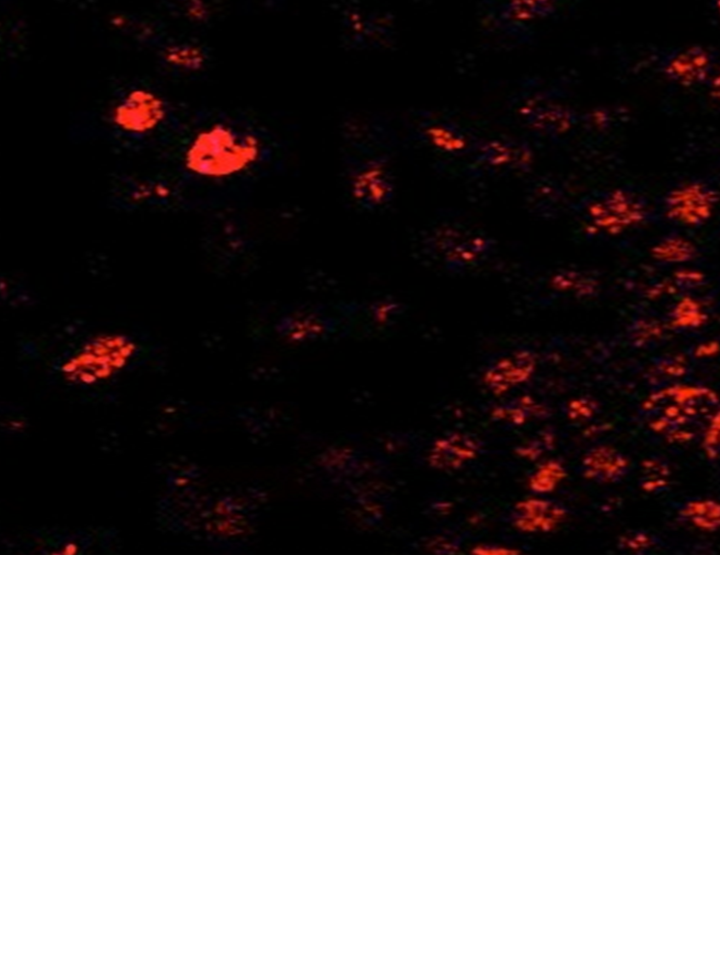

Supplement: Supplementary file 5 — Source data Fig. 2 [file 44321_2025_196_MOESM5_ESM.zip › MM-2024-19448_SourceDataForFig 2/MM-2024-19448_SourceDataForFig 2H/Apelin-dm KI-67.TIF]

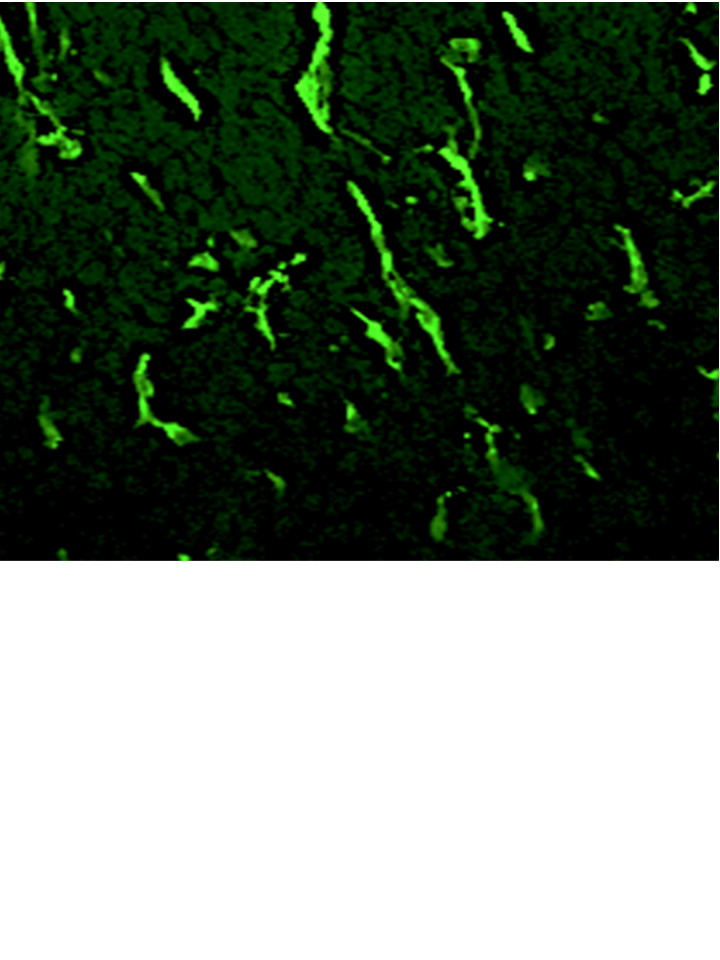

Supplement: Supplementary file 5 — Source data Fig. 2 [file 44321_2025_196_MOESM5_ESM.zip › MM-2024-19448_SourceDataForFig 2/MM-2024-19448_SourceDataForFig 2H/Control CD31.TIF]

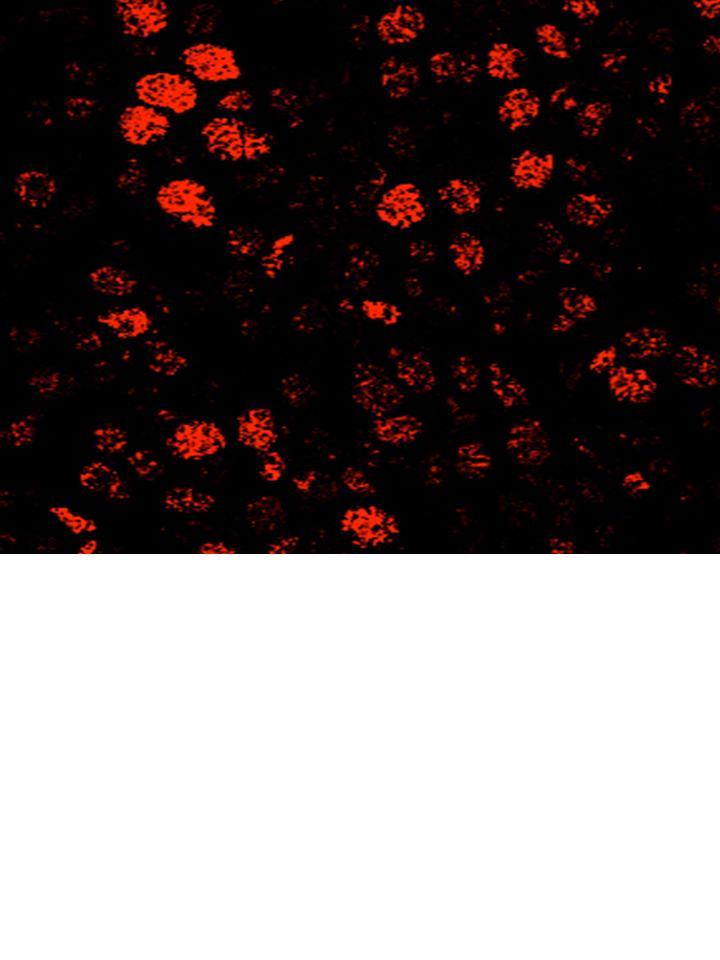

Supplement: Supplementary file 5 — Source data Fig. 2 [file 44321_2025_196_MOESM5_ESM.zip › MM-2024-19448_SourceDataForFig 2/MM-2024-19448_SourceDataForFig 2H/Control KI-67.TIF]

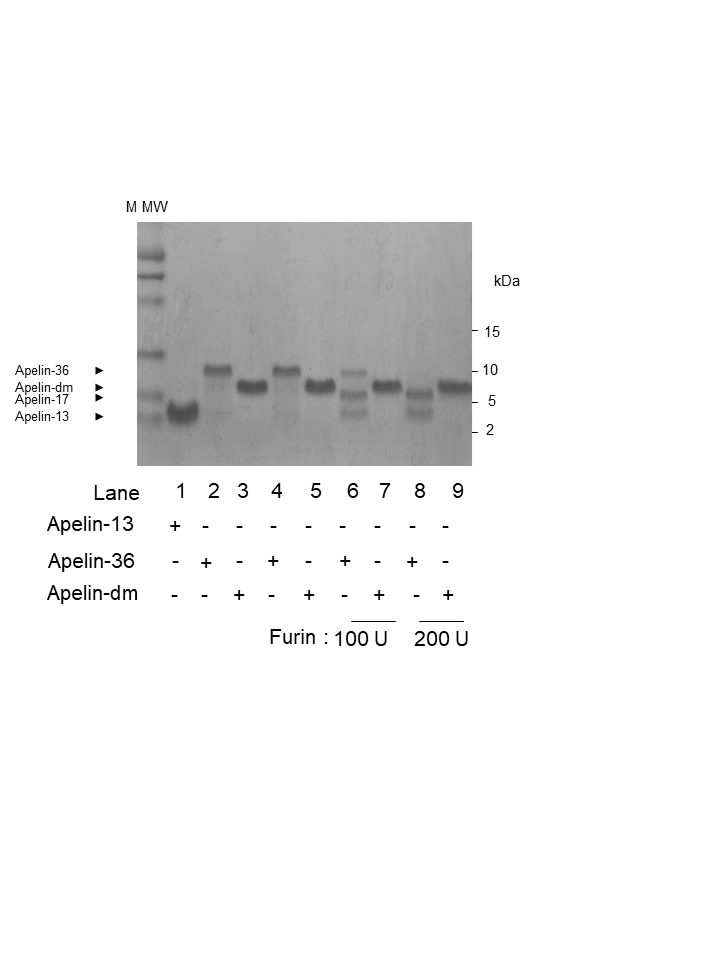

Supplement: Supplementary file 6 — Source data Fig. 3 [file 44321_2025_196_MOESM6_ESM.zip › MM-2024-19448_SourceDataForFig 3/MM-2024-19448_SourceDataForFig 3B.tif]

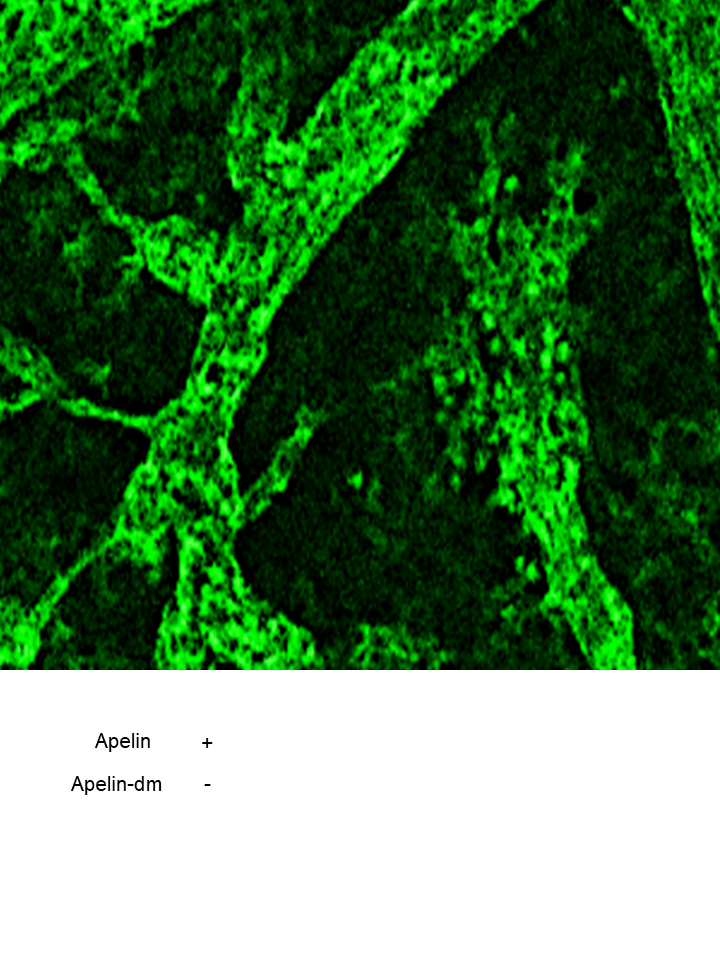

Supplement: Supplementary file 6 — Source data Fig. 3 [file 44321_2025_196_MOESM6_ESM.zip › MM-2024-19448_SourceDataForFig 3/MM-2024-19448_SourceDataForFig 3D/apelin alone zoom.TIF]

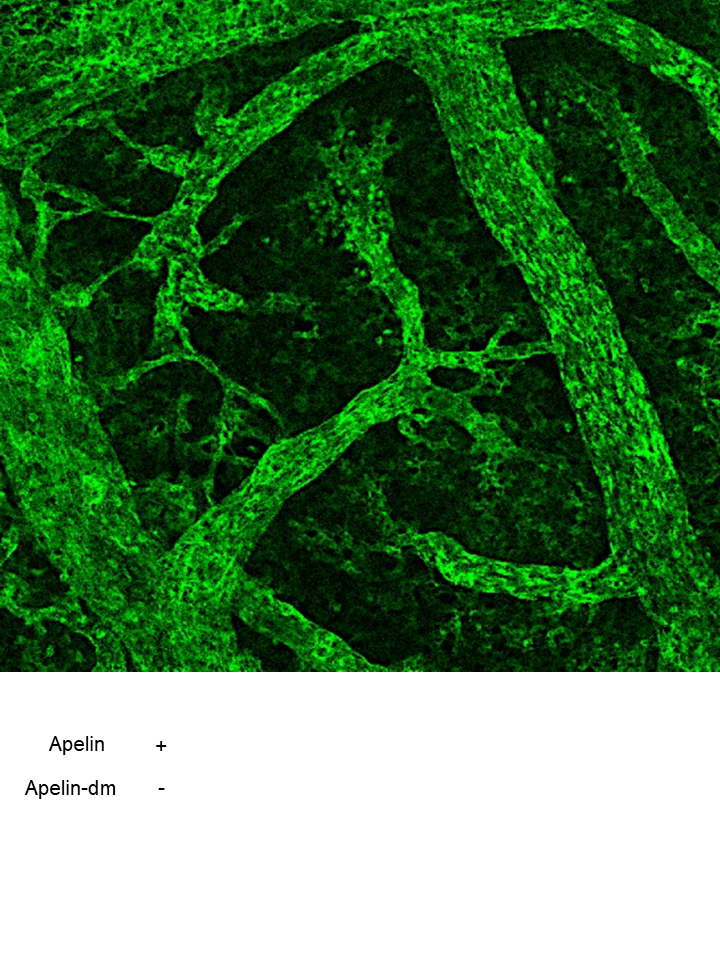

Supplement: Supplementary file 6 — Source data Fig. 3 [file 44321_2025_196_MOESM6_ESM.zip › MM-2024-19448_SourceDataForFig 3/MM-2024-19448_SourceDataForFig 3D/apelin alone.TIF]

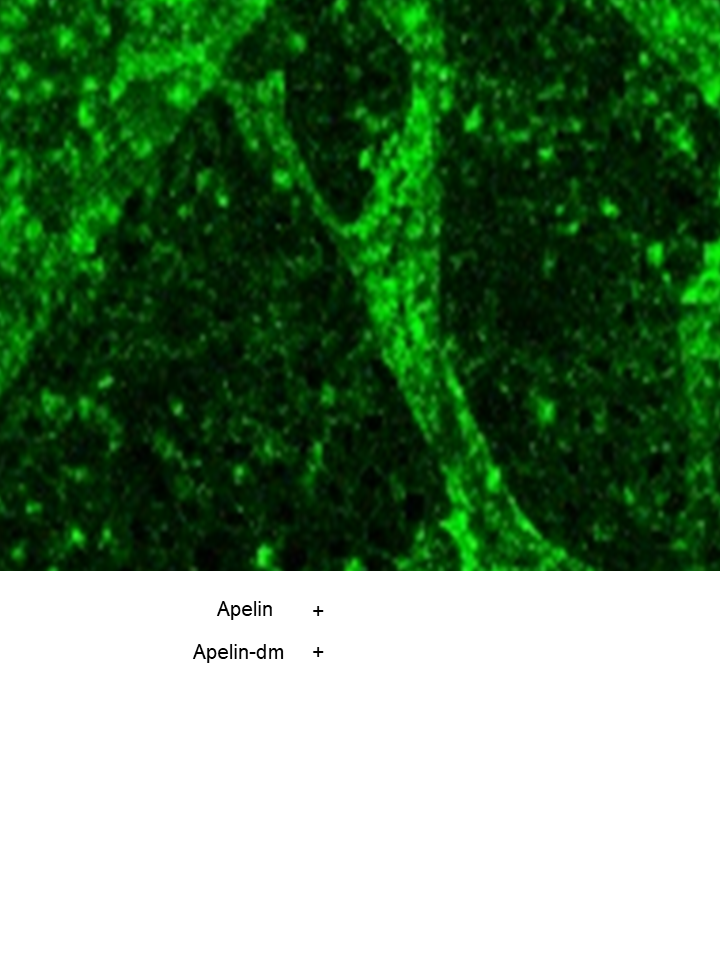

Supplement: Supplementary file 6 — Source data Fig. 3 [file 44321_2025_196_MOESM6_ESM.zip › MM-2024-19448_SourceDataForFig 3/MM-2024-19448_SourceDataForFig 3D/apelin and apelin dm zoom.TIF]

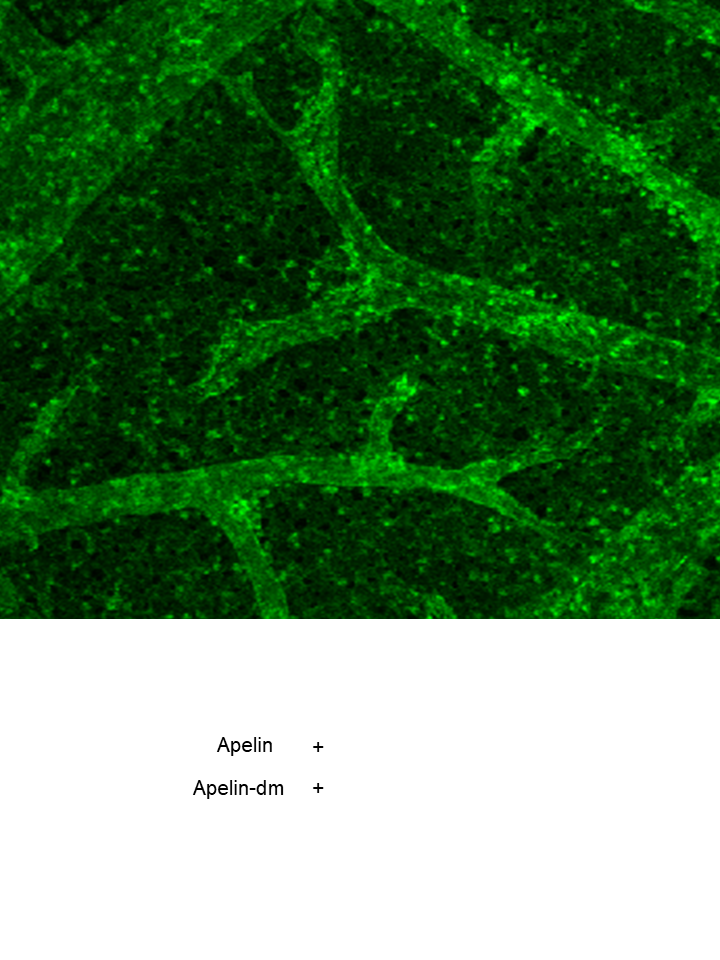

Supplement: Supplementary file 6 — Source data Fig. 3 [file 44321_2025_196_MOESM6_ESM.zip › MM-2024-19448_SourceDataForFig 3/MM-2024-19448_SourceDataForFig 3D/apelin and apelin dm.TIF]

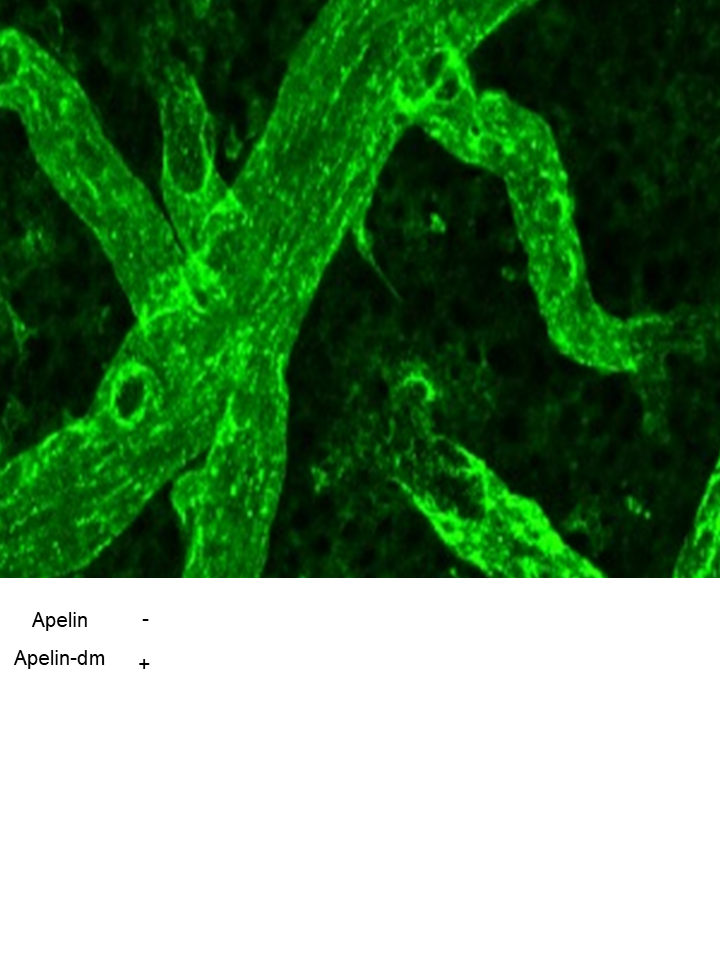

Supplement: Supplementary file 6 — Source data Fig. 3 [file 44321_2025_196_MOESM6_ESM.zip › MM-2024-19448_SourceDataForFig 3/MM-2024-19448_SourceDataForFig 3D/apelin-dm alone zoom.TIF]

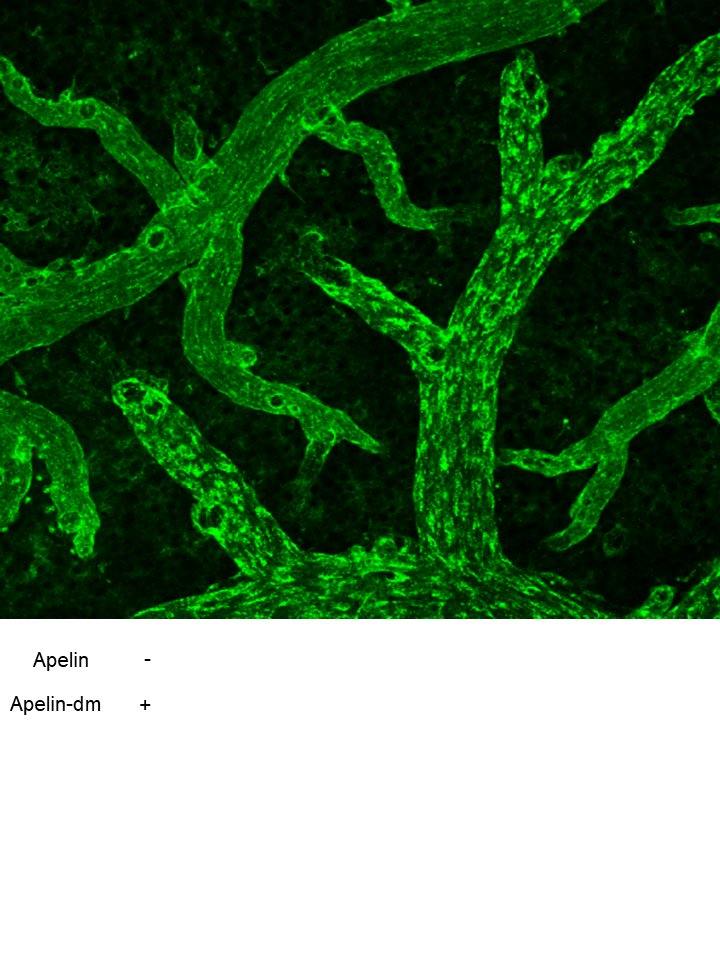

Supplement: Supplementary file 6 — Source data Fig. 3 [file 44321_2025_196_MOESM6_ESM.zip › MM-2024-19448_SourceDataForFig 3/MM-2024-19448_SourceDataForFig 3D/apelin-dm alone.TIF]

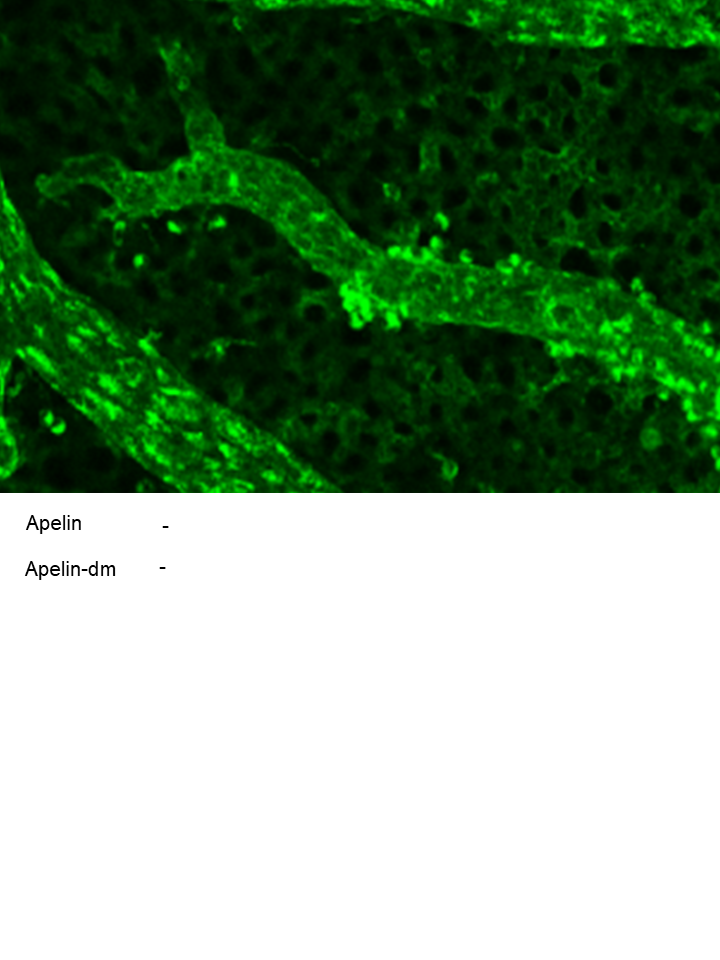

Supplement: Supplementary file 6 — Source data Fig. 3 [file 44321_2025_196_MOESM6_ESM.zip › MM-2024-19448_SourceDataForFig 3/MM-2024-19448_SourceDataForFig 3D/Control apelin and apelin dm zoom.TIF]

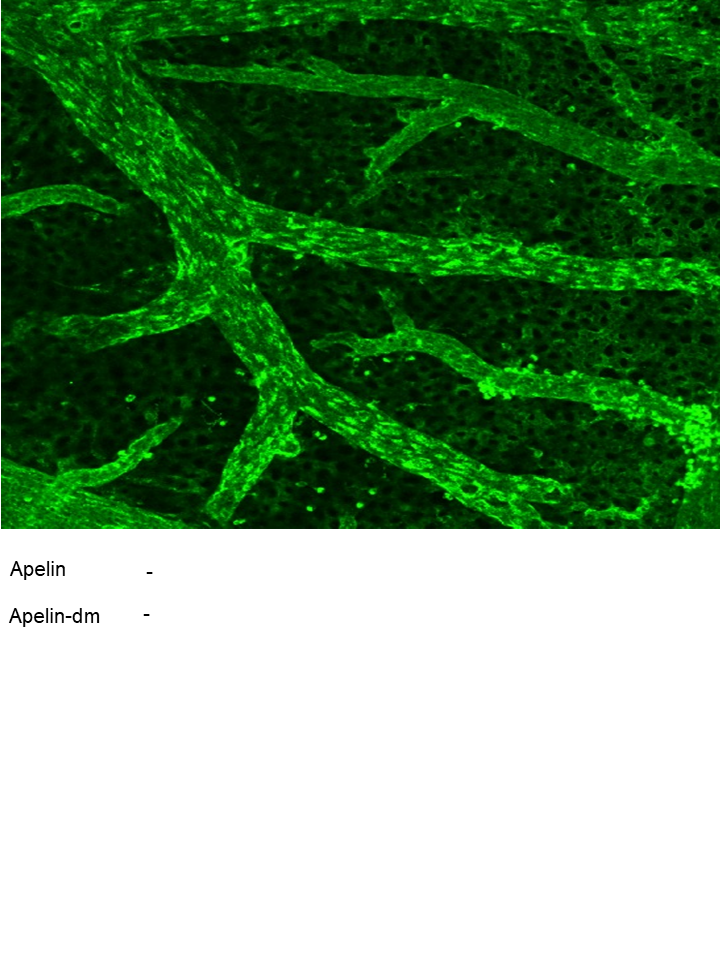

Supplement: Supplementary file 6 — Source data Fig. 3 [file 44321_2025_196_MOESM6_ESM.zip › MM-2024-19448_SourceDataForFig 3/MM-2024-19448_SourceDataForFig 3D/Control apelin and apelin dm.TIF]

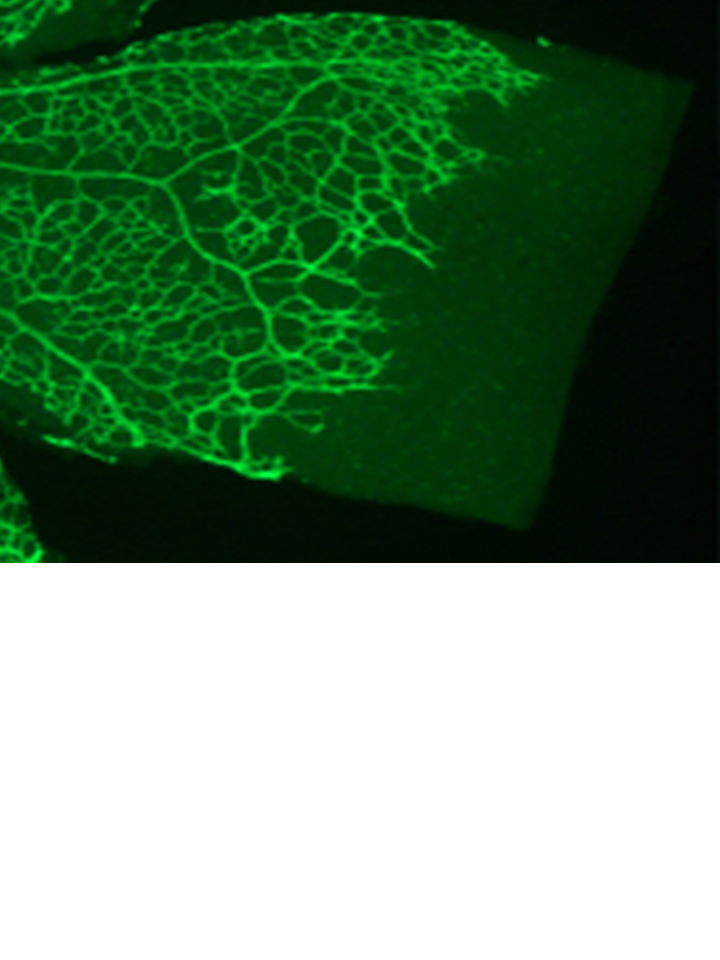

Supplement: Supplementary file 6 — Source data Fig. 3 [file 44321_2025_196_MOESM6_ESM.zip › MM-2024-19448_SourceDataForFig 3/MM-2024-19448_SourceDataForFig 3F/apelin and Apelin-dm.TIF]

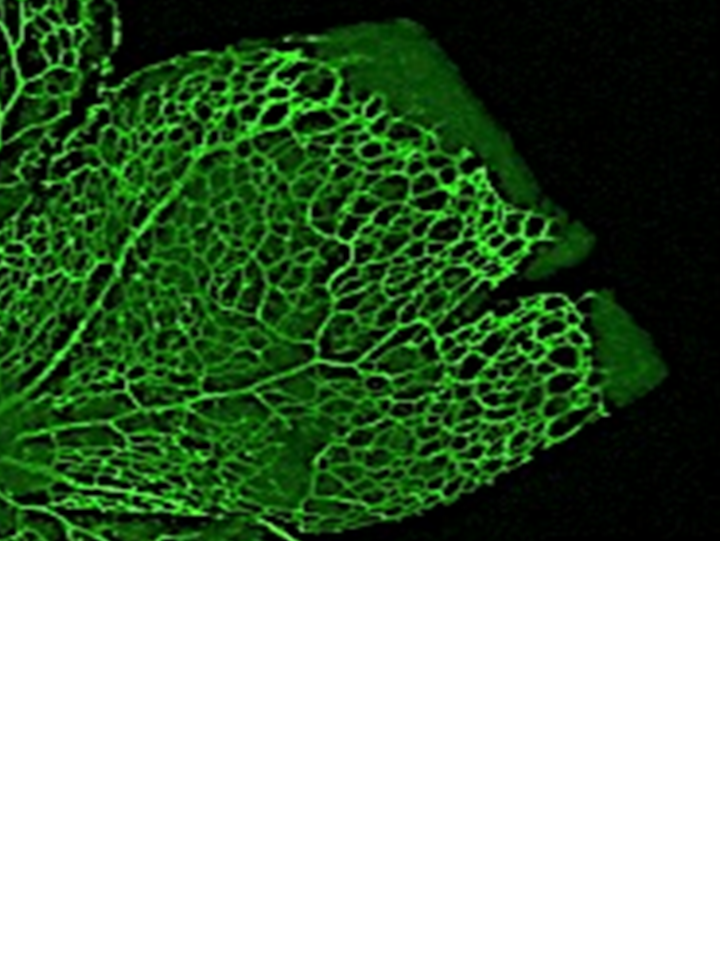

Supplement: Supplementary file 6 — Source data Fig. 3 [file 44321_2025_196_MOESM6_ESM.zip › MM-2024-19448_SourceDataForFig 3/MM-2024-19448_SourceDataForFig 3F/Apelin.TIF]

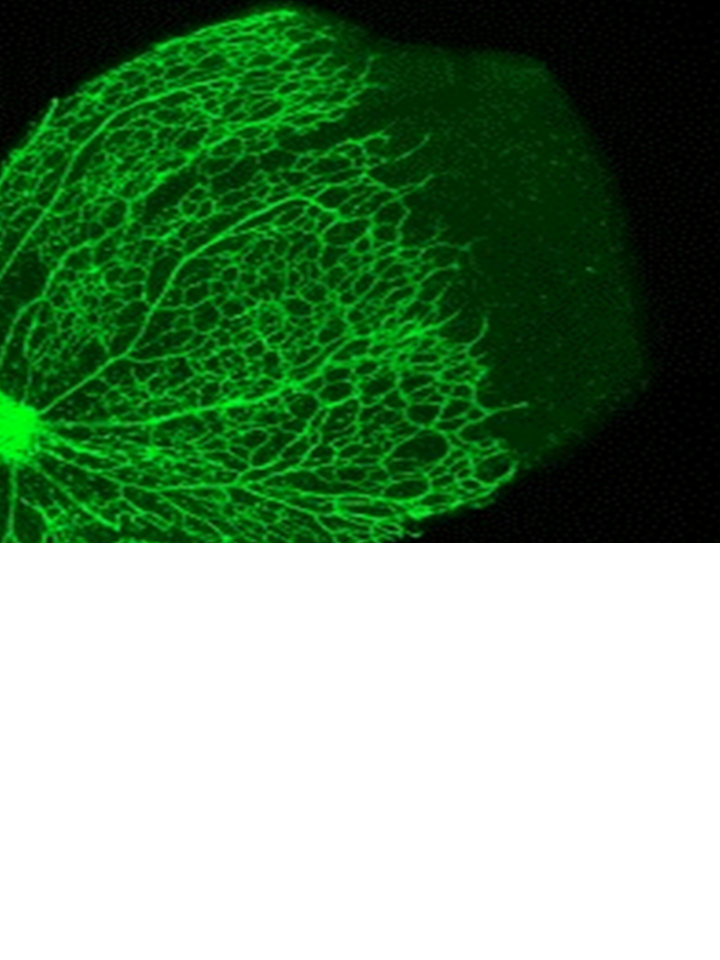

Supplement: Supplementary file 6 — Source data Fig. 3 [file 44321_2025_196_MOESM6_ESM.zip › MM-2024-19448_SourceDataForFig 3/MM-2024-19448_SourceDataForFig 3F/Apelin-dm.TIF]

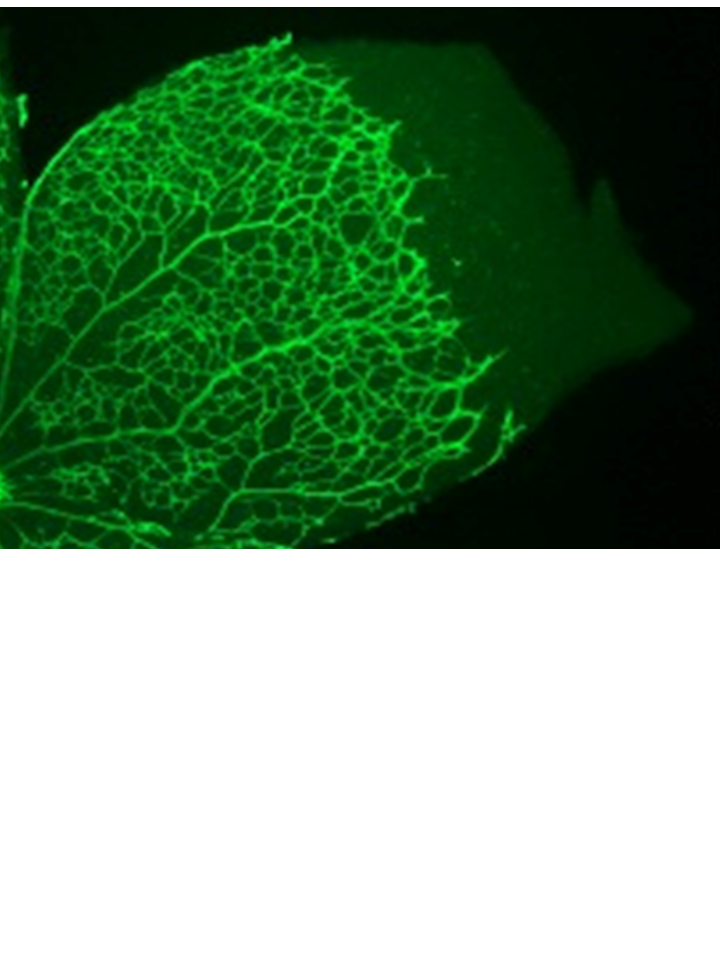

Supplement: Supplementary file 6 — Source data Fig. 3 [file 44321_2025_196_MOESM6_ESM.zip › MM-2024-19448_SourceDataForFig 3/MM-2024-19448_SourceDataForFig 3F/Control.TIF]

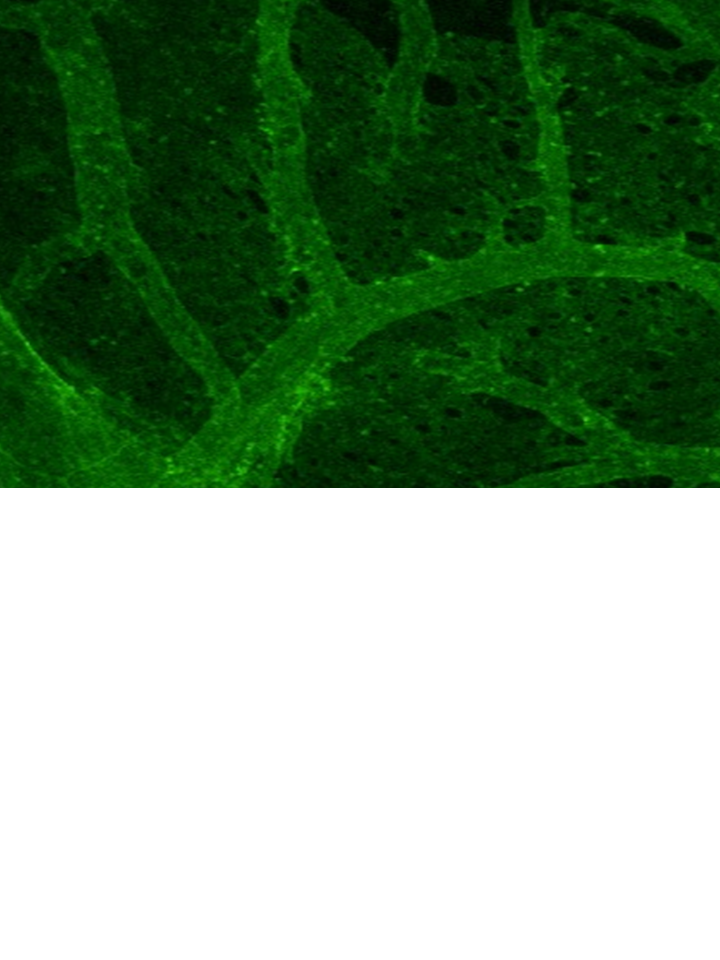

Supplement: Supplementary file 6 — Source data Fig. 3 [file 44321_2025_196_MOESM6_ESM.zip › MM-2024-19448_SourceDataForFig 3/MM-2024-19448_SourceDataForFig 3H/Control Zoom.TIF]

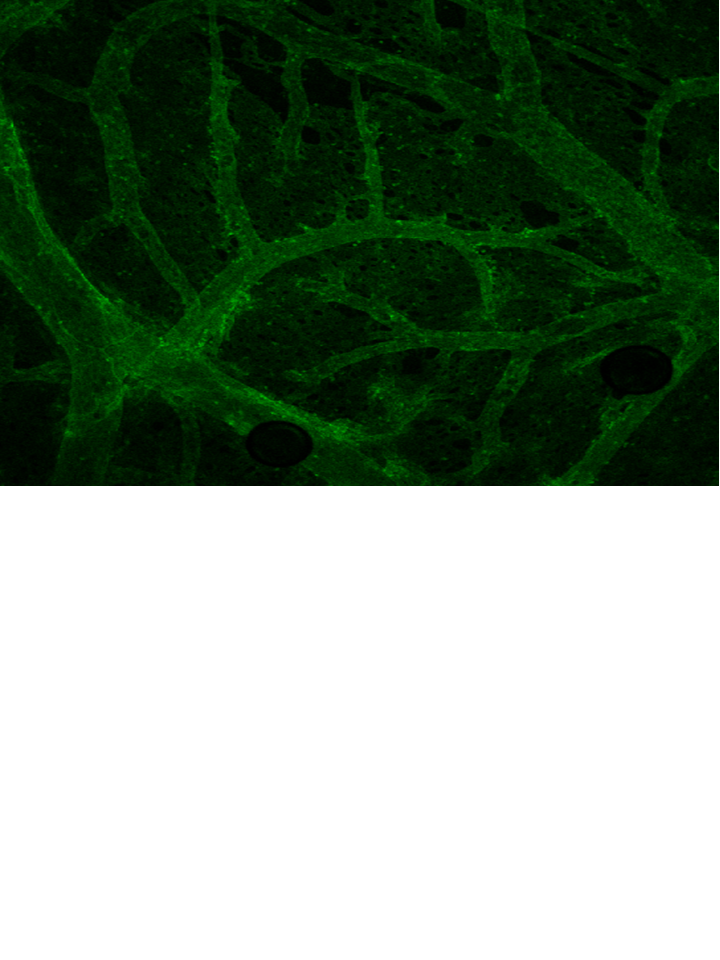

Supplement: Supplementary file 6 — Source data Fig. 3 [file 44321_2025_196_MOESM6_ESM.zip › MM-2024-19448_SourceDataForFig 3/MM-2024-19448_SourceDataForFig 3H/Control.TIF]

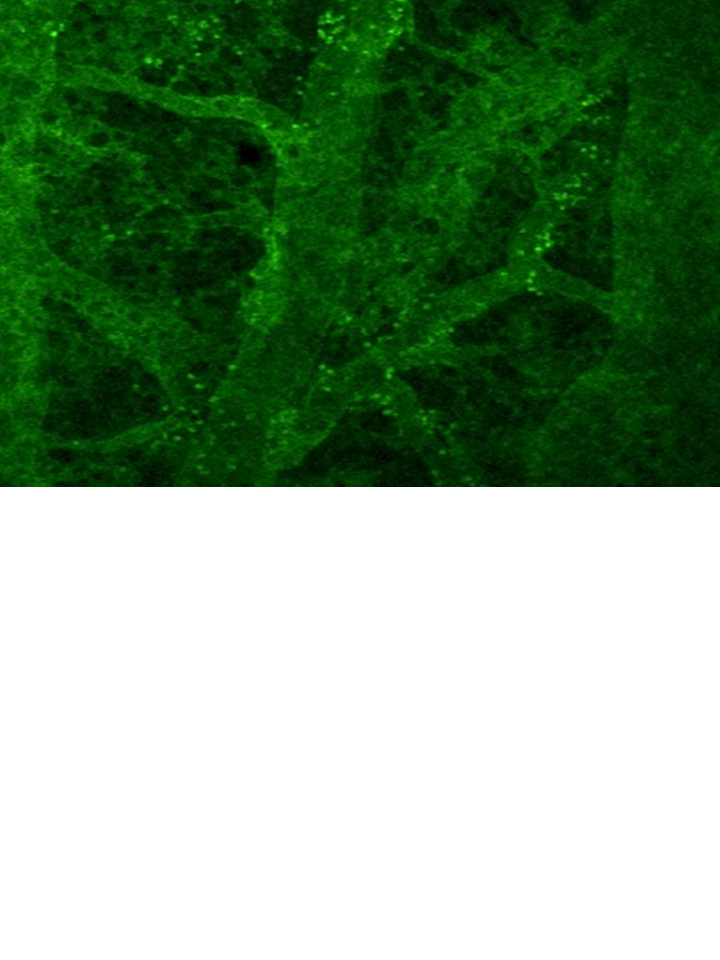

Supplement: Supplementary file 6 — Source data Fig. 3 [file 44321_2025_196_MOESM6_ESM.zip › MM-2024-19448_SourceDataForFig 3/MM-2024-19448_SourceDataForFig 3H/VEGF abd Apelin-dm zoom.TIF]

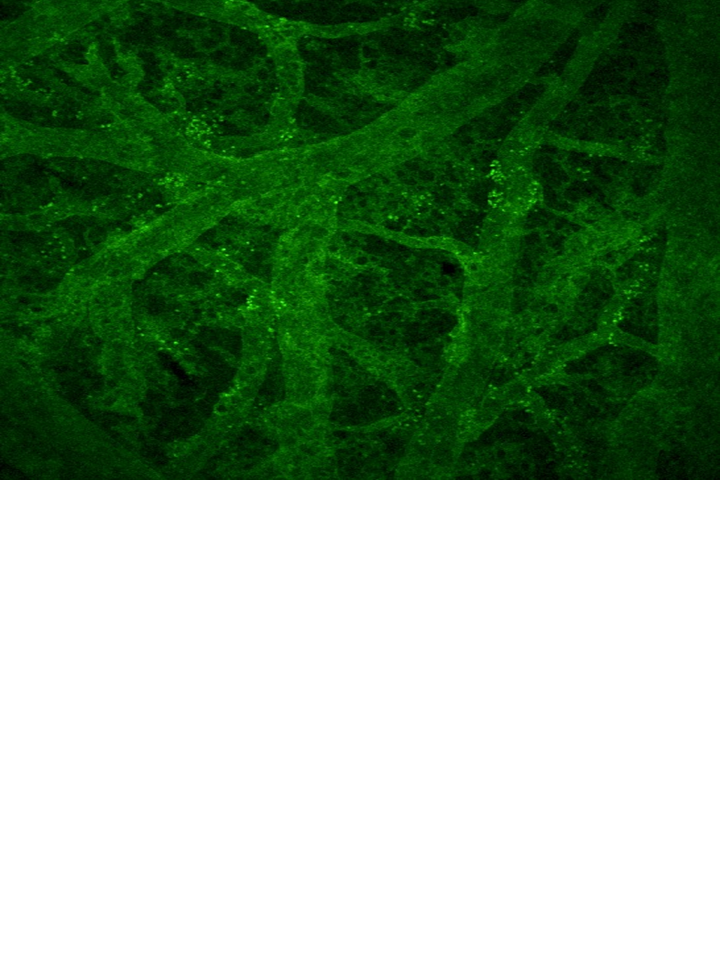

Supplement: Supplementary file 6 — Source data Fig. 3 [file 44321_2025_196_MOESM6_ESM.zip › MM-2024-19448_SourceDataForFig 3/MM-2024-19448_SourceDataForFig 3H/VEGF and Apelin-dm.TIF]

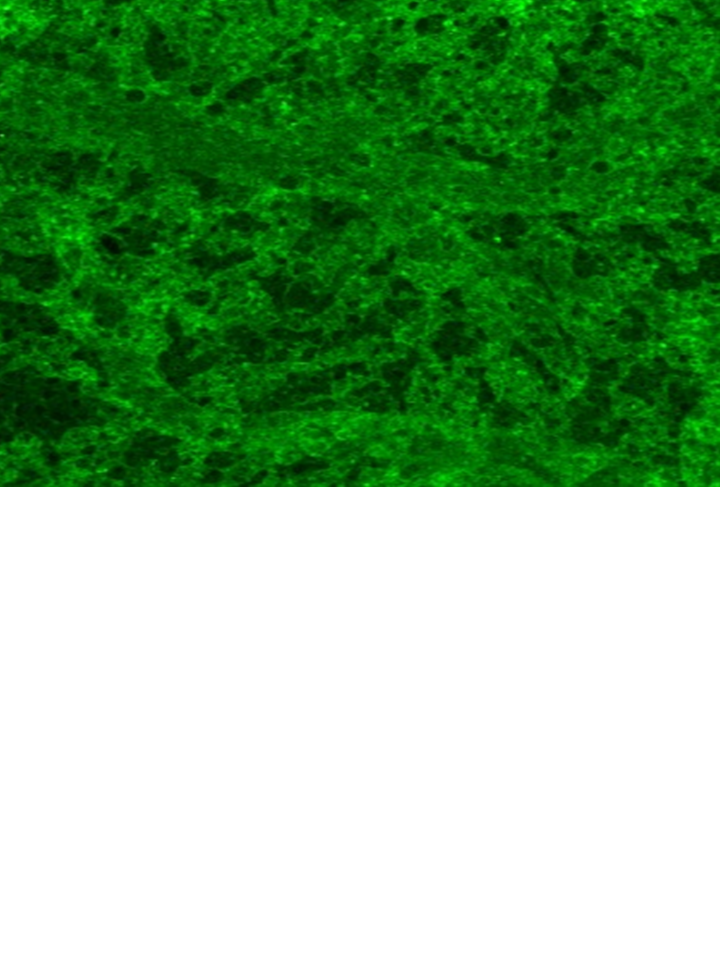

Supplement: Supplementary file 6 — Source data Fig. 3 [file 44321_2025_196_MOESM6_ESM.zip › MM-2024-19448_SourceDataForFig 3/MM-2024-19448_SourceDataForFig 3H/VEGF Zoom.TIF]

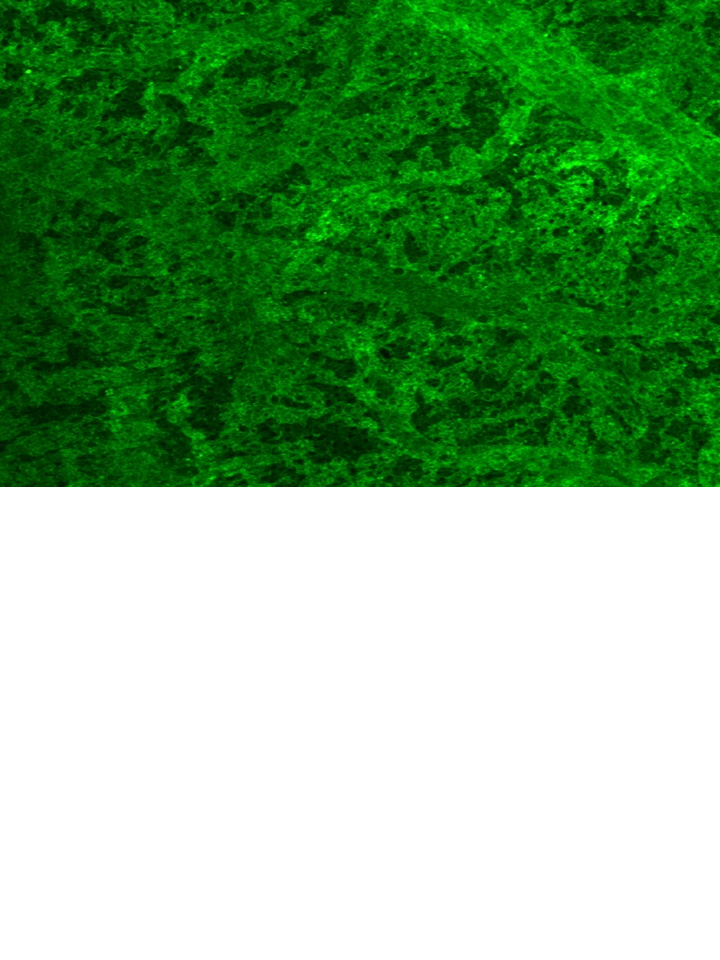

Supplement: Supplementary file 6 — Source data Fig. 3 [file 44321_2025_196_MOESM6_ESM.zip › MM-2024-19448_SourceDataForFig 3/MM-2024-19448_SourceDataForFig 3H/VEGF.TIF]

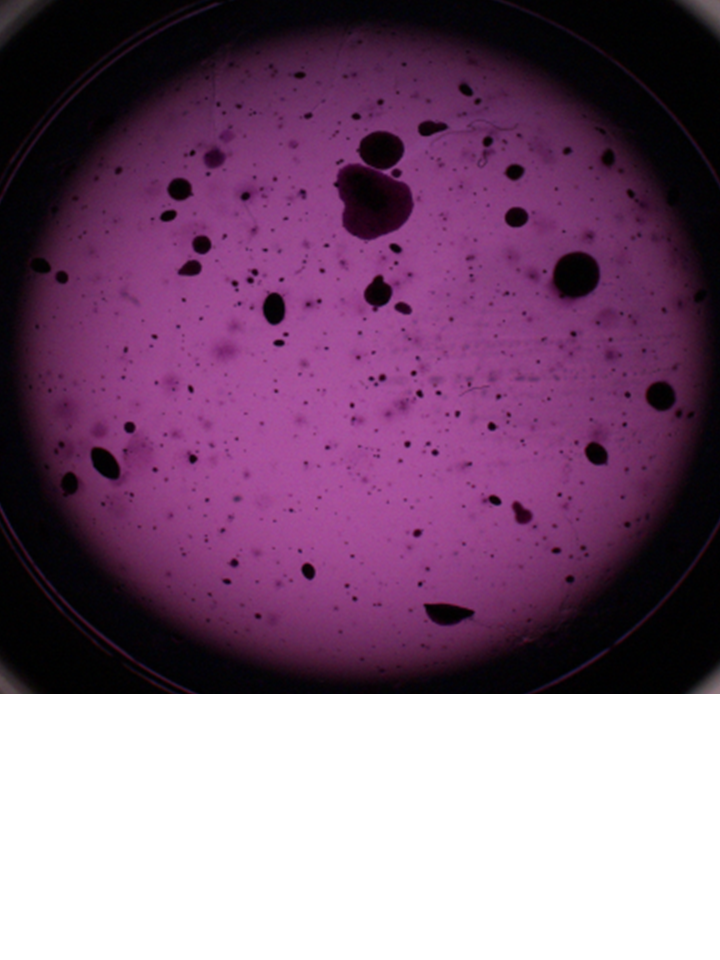

Supplement: Supplementary file 6 — Source data Fig. 3 [file 44321_2025_196_MOESM6_ESM.zip › MM-2024-19448_SourceDataForFig 3/MM-2024-19448_SourceDataForFig 3L/apelin and apelin-dm.TIF]

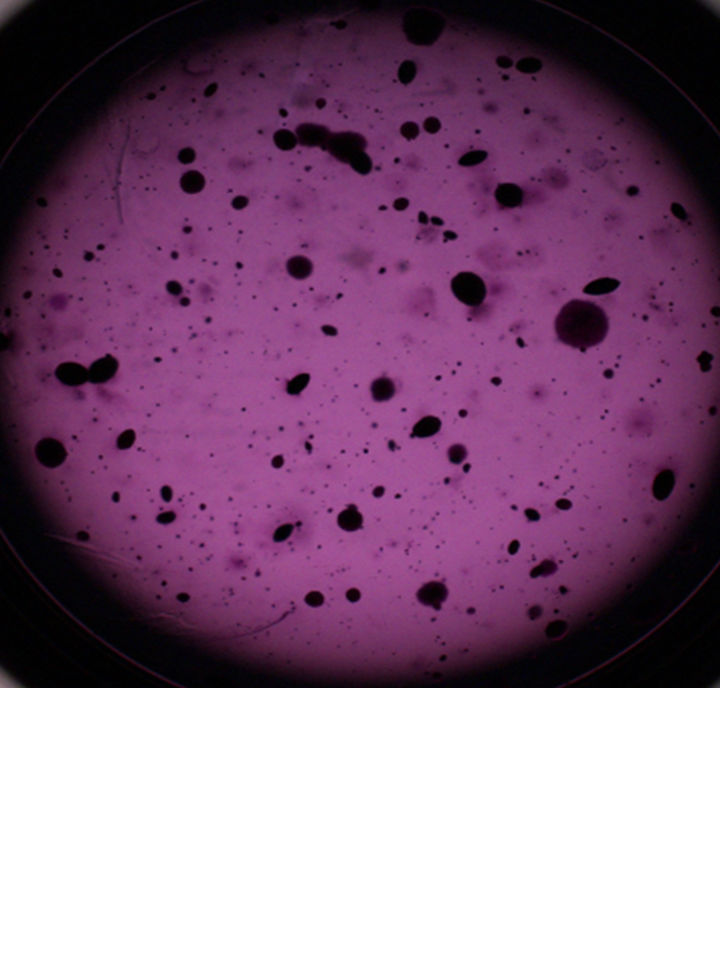

Supplement: Supplementary file 6 — Source data Fig. 3 [file 44321_2025_196_MOESM6_ESM.zip › MM-2024-19448_SourceDataForFig 3/MM-2024-19448_SourceDataForFig 3L/Apelin.TIF]

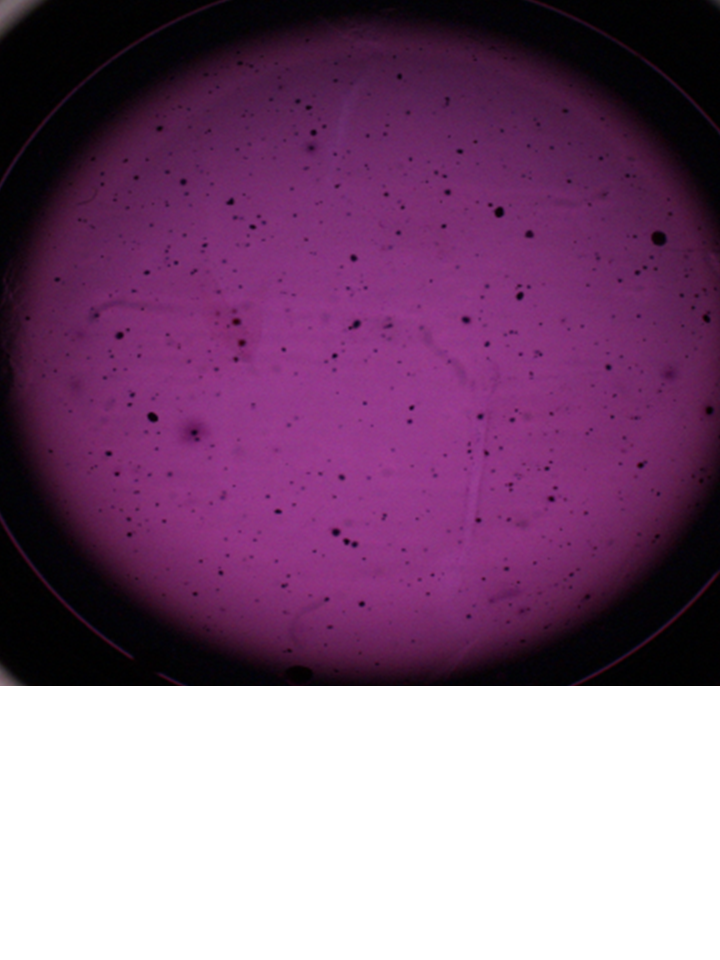

Supplement: Supplementary file 6 — Source data Fig. 3 [file 44321_2025_196_MOESM6_ESM.zip › MM-2024-19448_SourceDataForFig 3/MM-2024-19448_SourceDataForFig 3L/Apelin-dm.TIF]

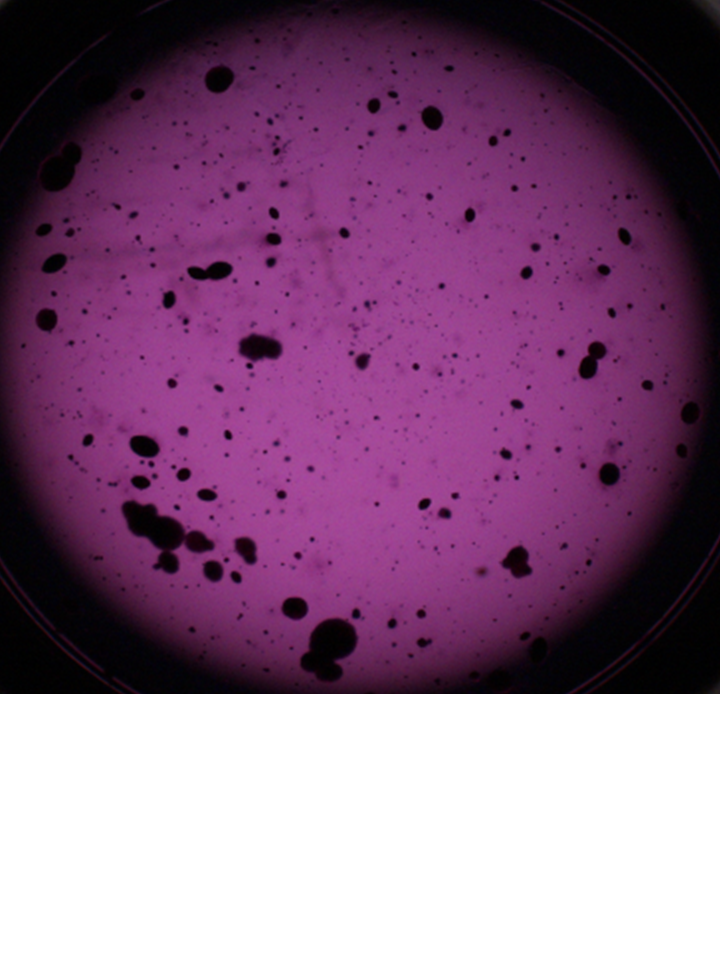

Supplement: Supplementary file 6 — Source data Fig. 3 [file 44321_2025_196_MOESM6_ESM.zip › MM-2024-19448_SourceDataForFig 3/MM-2024-19448_SourceDataForFig 3L/Control.TIF]

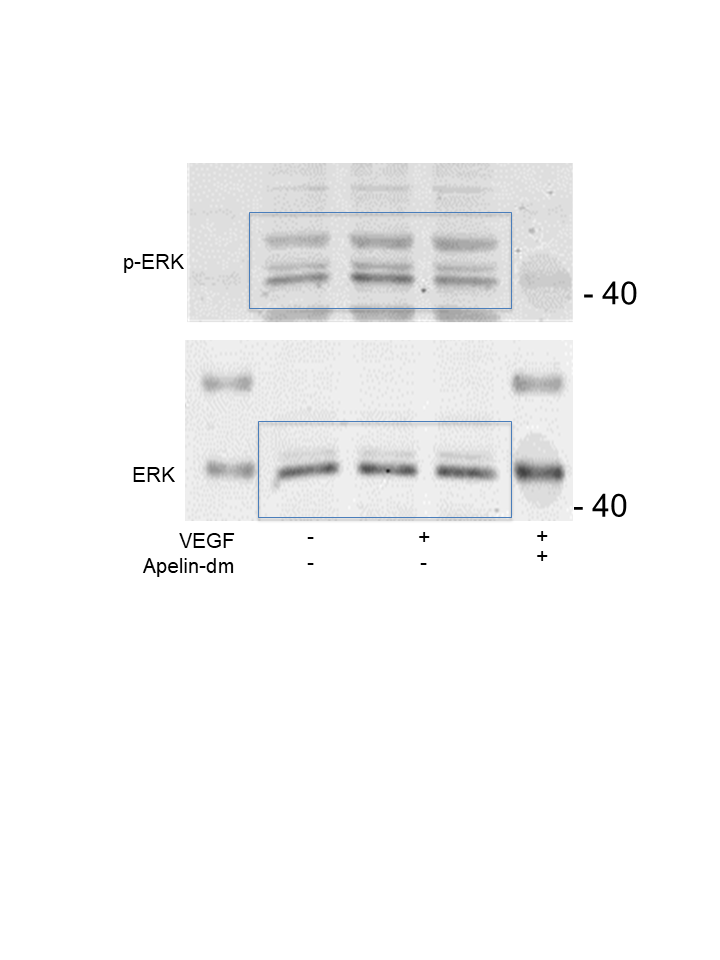

Supplement: Supplementary file 6 — Source data Fig. 3 [file 44321_2025_196_MOESM6_ESM.zip › MM-2024-19448_SourceDataForFig 3/MM-2024-19448_SourceDataForFig 3M.tif]

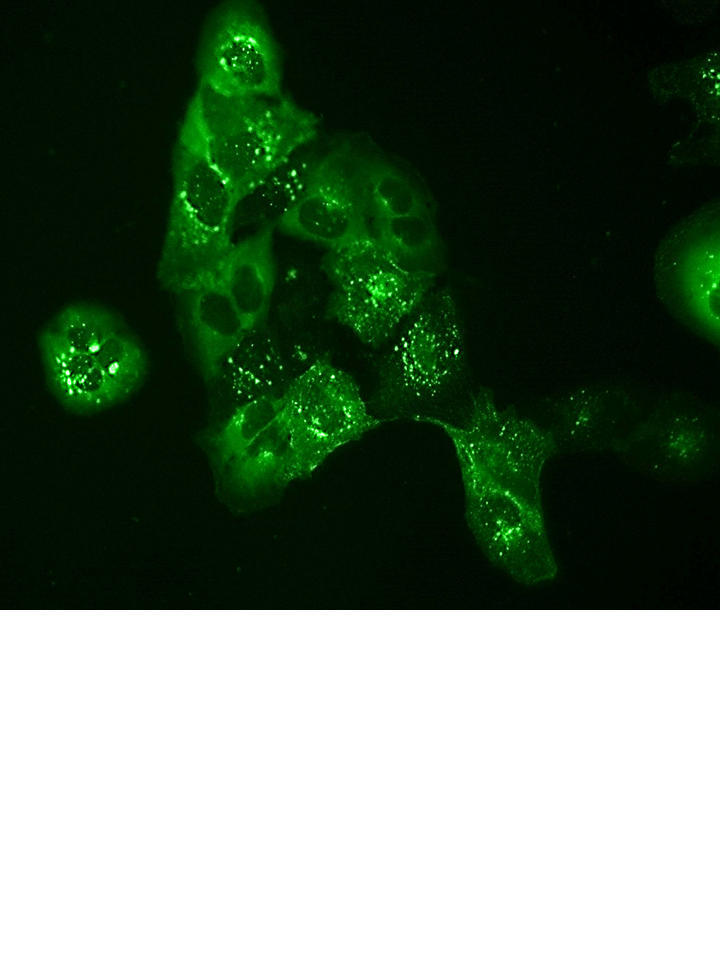

Supplement: Supplementary file 7 — Source data Fig. 4 [file 44321_2025_196_MOESM7_ESM.zip › MM-2024-19448_SourceDataForFig 4/MM-2024-19448_SourceDataForFig 4A/Apelin and APELIN-DM Arrestin.TIF]

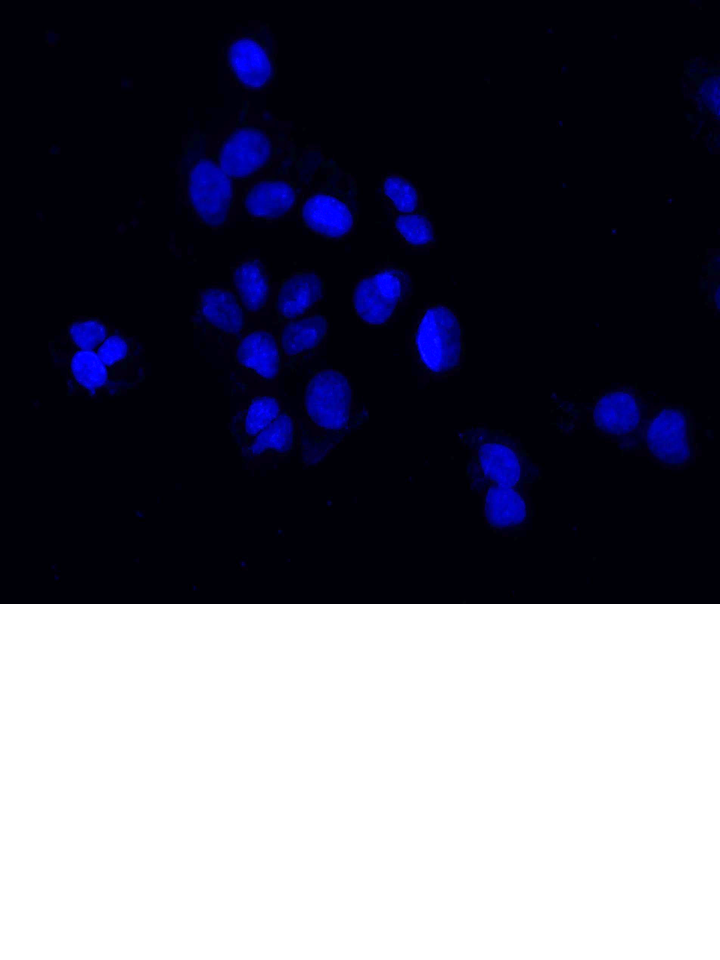

Supplement: Supplementary file 7 — Source data Fig. 4 [file 44321_2025_196_MOESM7_ESM.zip › MM-2024-19448_SourceDataForFig 4/MM-2024-19448_SourceDataForFig 4A/Apelin and APELIN-DM Dapi.TIF]

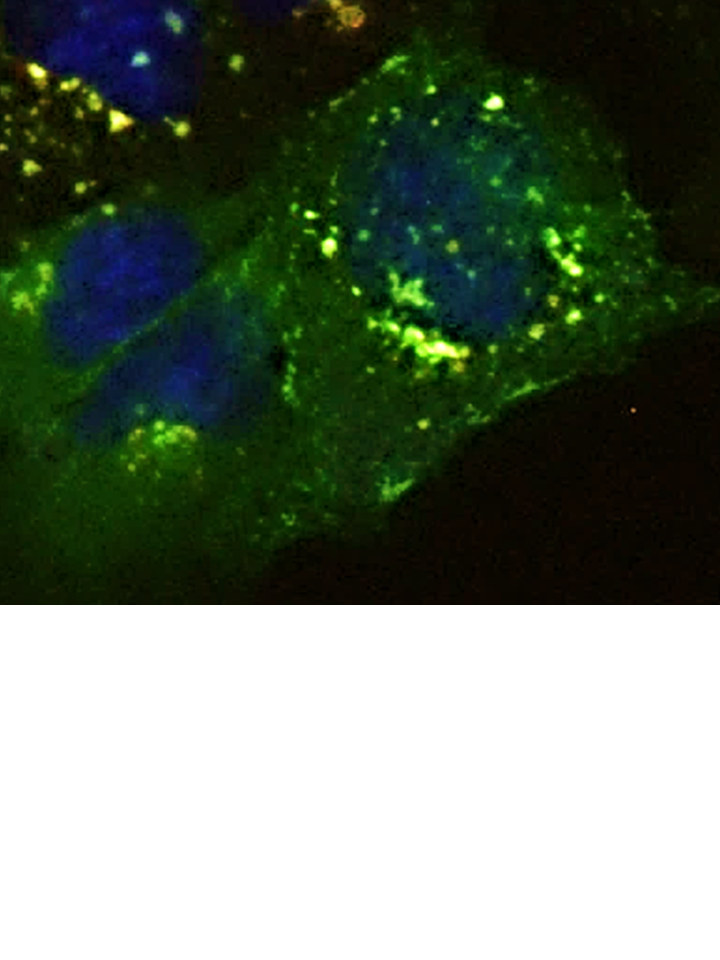

Supplement: Supplementary file 7 — Source data Fig. 4 [file 44321_2025_196_MOESM7_ESM.zip › MM-2024-19448_SourceDataForFig 4/MM-2024-19448_SourceDataForFig 4A/Apelin and Apelin-dm Merge Zoom.TIF]

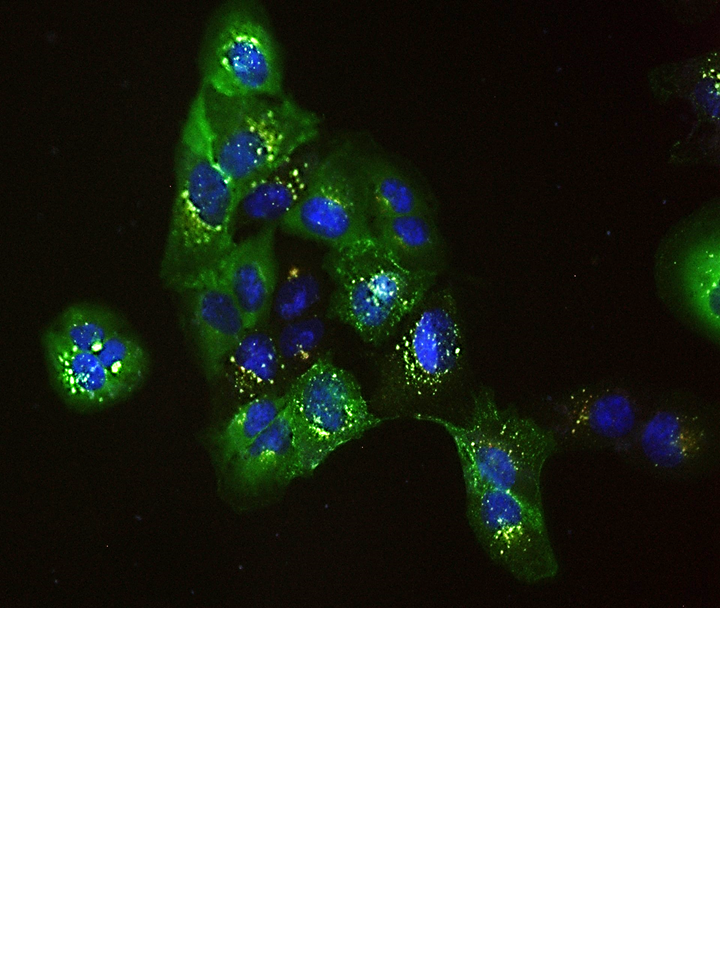

Supplement: Supplementary file 7 — Source data Fig. 4 [file 44321_2025_196_MOESM7_ESM.zip › MM-2024-19448_SourceDataForFig 4/MM-2024-19448_SourceDataForFig 4A/Apelin and Apelin-dm Merge.TIF]

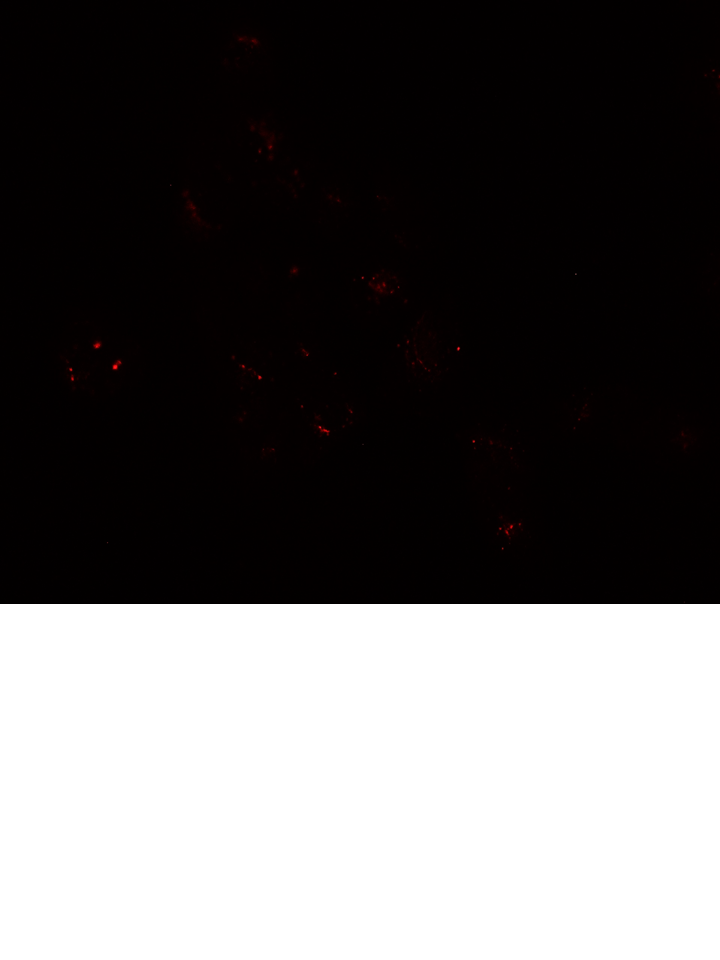

Supplement: Supplementary file 7 — Source data Fig. 4 [file 44321_2025_196_MOESM7_ESM.zip › MM-2024-19448_SourceDataForFig 4/MM-2024-19448_SourceDataForFig 4A/Apelin and APELIN-DM Tamara.TIF]

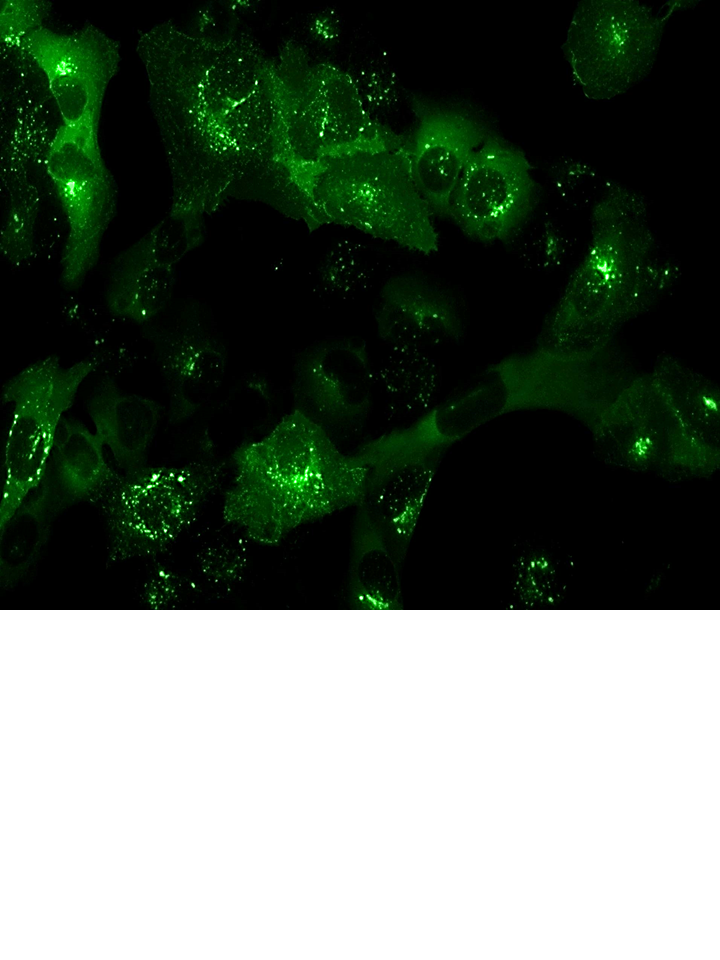

Supplement: Supplementary file 7 — Source data Fig. 4 [file 44321_2025_196_MOESM7_ESM.zip › MM-2024-19448_SourceDataForFig 4/MM-2024-19448_SourceDataForFig 4A/Apelin Arrestin.TIF]

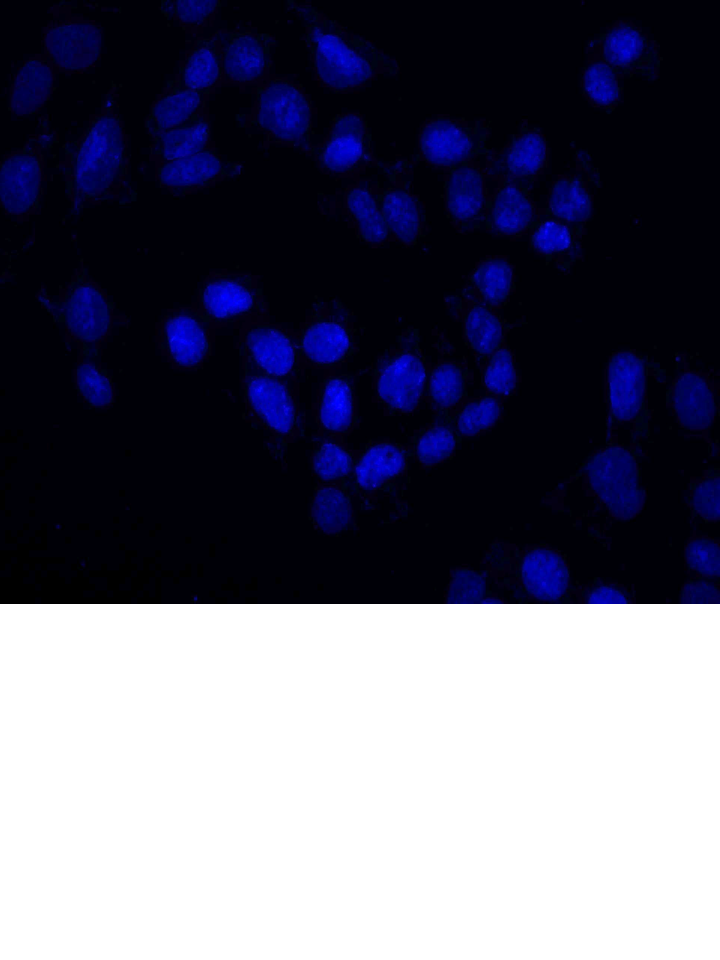

Supplement: Supplementary file 7 — Source data Fig. 4 [file 44321_2025_196_MOESM7_ESM.zip › MM-2024-19448_SourceDataForFig 4/MM-2024-19448_SourceDataForFig 4A/Apelin Dapi.TIF]

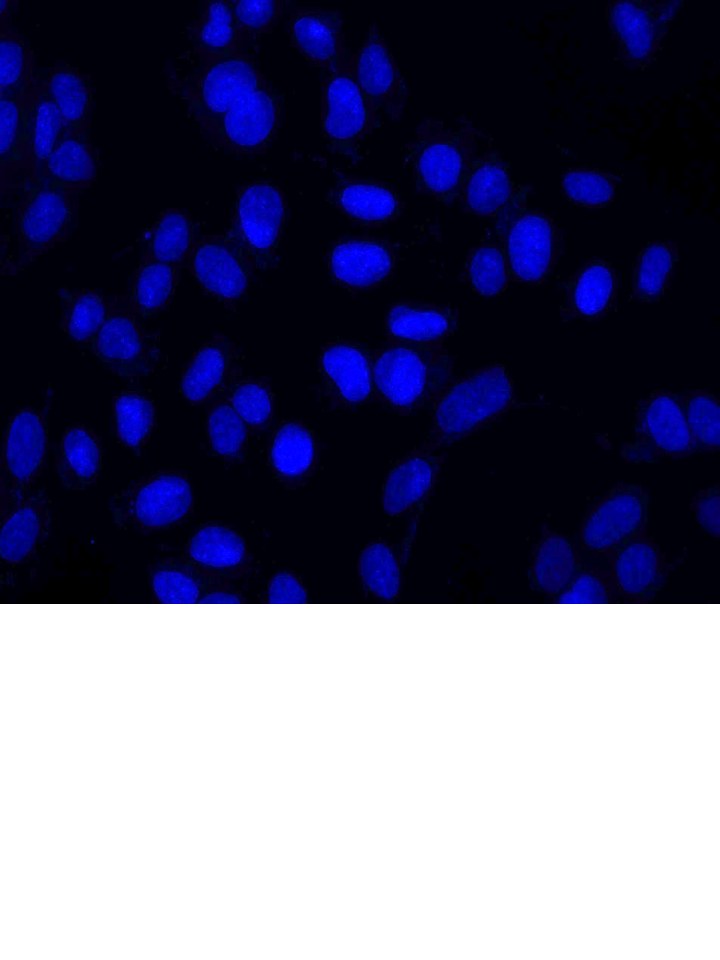

Supplement: Supplementary file 7 — Source data Fig. 4 [file 44321_2025_196_MOESM7_ESM.zip › MM-2024-19448_SourceDataForFig 4/MM-2024-19448_SourceDataForFig 4A/Apelin dm Dapi.TIF]

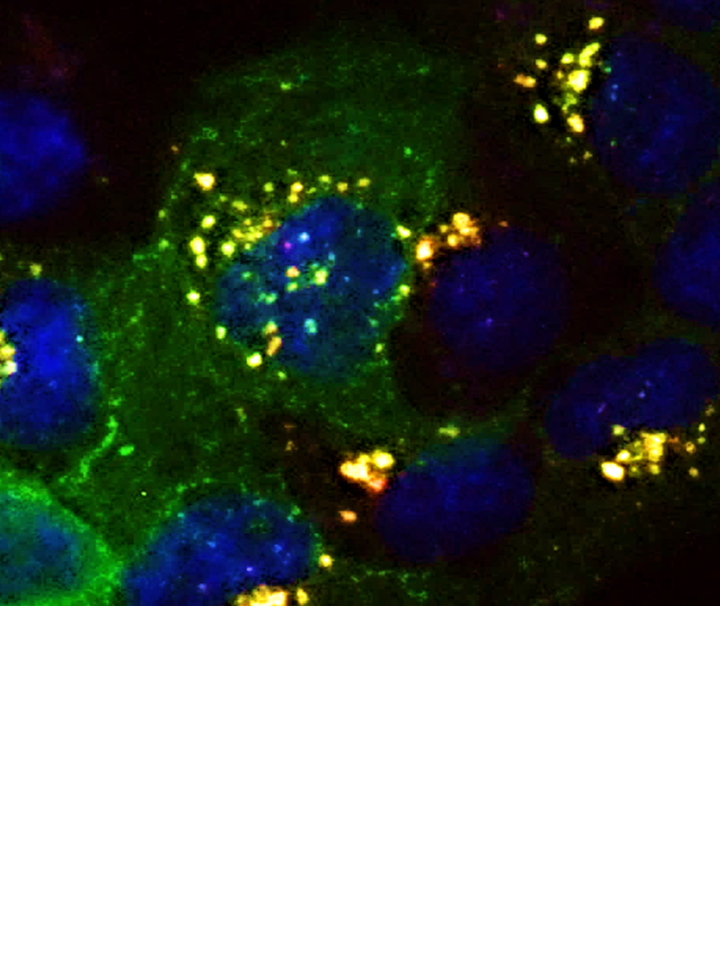

Supplement: Supplementary file 7 — Source data Fig. 4 [file 44321_2025_196_MOESM7_ESM.zip › MM-2024-19448_SourceDataForFig 4/MM-2024-19448_SourceDataForFig 4A/Apelin Merge Zoom.TIF]

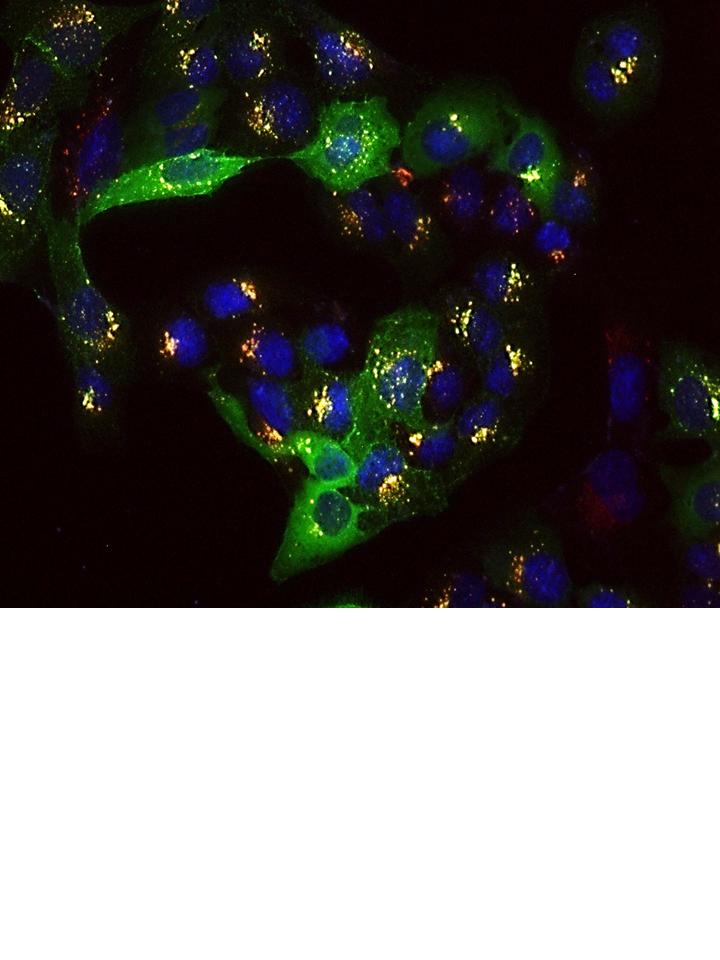

Supplement: Supplementary file 7 — Source data Fig. 4 [file 44321_2025_196_MOESM7_ESM.zip › MM-2024-19448_SourceDataForFig 4/MM-2024-19448_SourceDataForFig 4A/Apelin Merge.TIF]

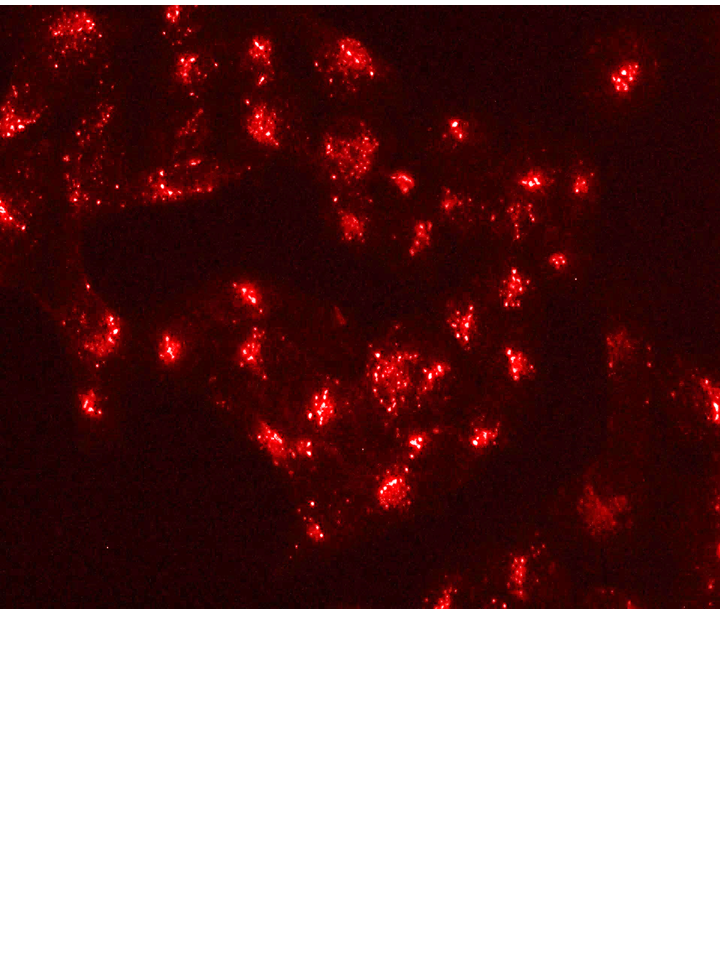

Supplement: Supplementary file 7 — Source data Fig. 4 [file 44321_2025_196_MOESM7_ESM.zip › MM-2024-19448_SourceDataForFig 4/MM-2024-19448_SourceDataForFig 4A/Apelin Tamara.TIF]

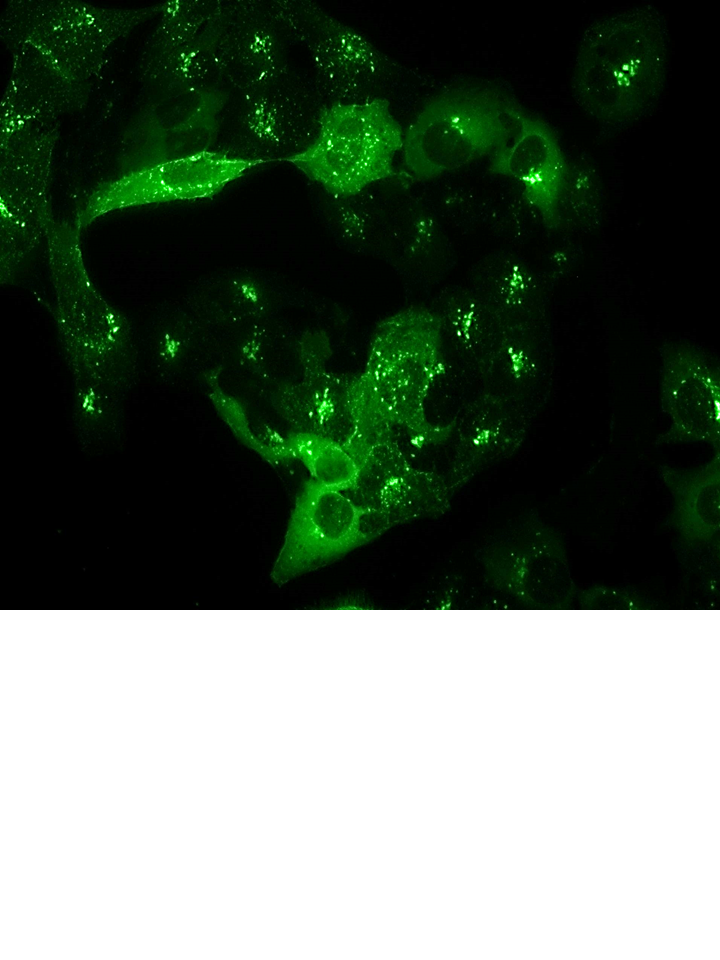

Supplement: Supplementary file 7 — Source data Fig. 4 [file 44321_2025_196_MOESM7_ESM.zip › MM-2024-19448_SourceDataForFig 4/MM-2024-19448_SourceDataForFig 4A/Apelin-dm Arrestin.TIF]

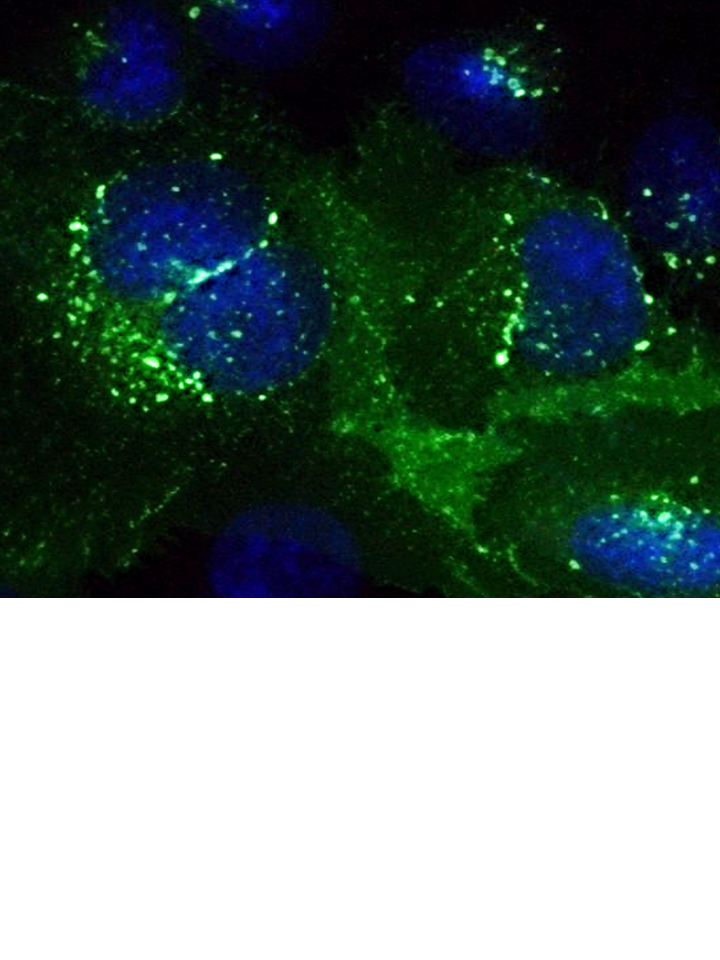

Supplement: Supplementary file 7 — Source data Fig. 4 [file 44321_2025_196_MOESM7_ESM.zip › MM-2024-19448_SourceDataForFig 4/MM-2024-19448_SourceDataForFig 4A/Apelin-dm Merge Zoom.TIF]

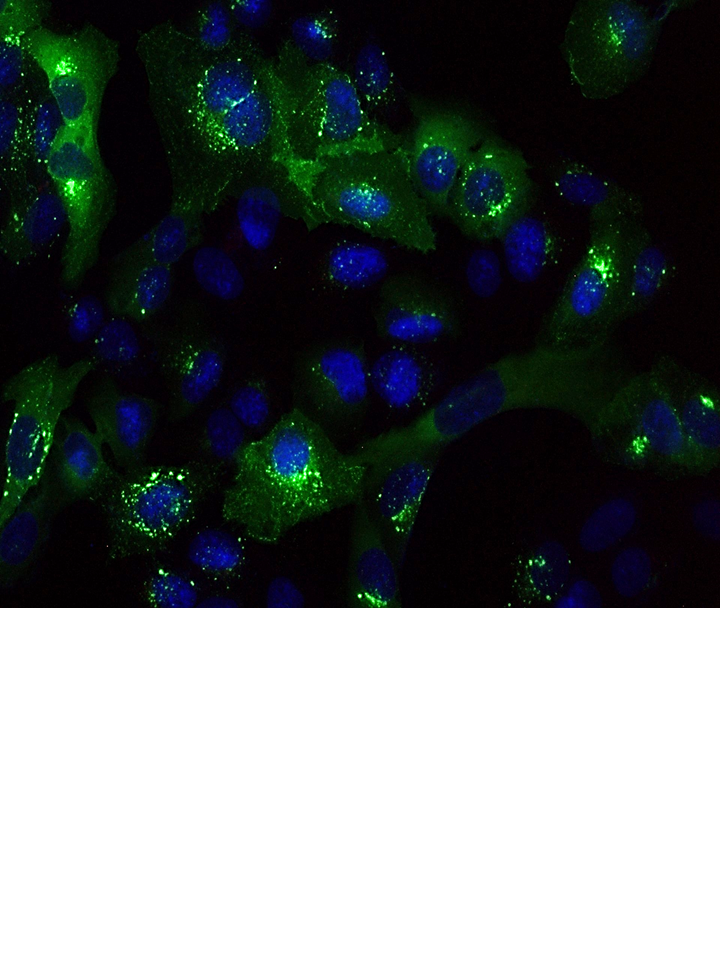

Supplement: Supplementary file 7 — Source data Fig. 4 [file 44321_2025_196_MOESM7_ESM.zip › MM-2024-19448_SourceDataForFig 4/MM-2024-19448_SourceDataForFig 4A/Apelin-dm Merge.TIF]

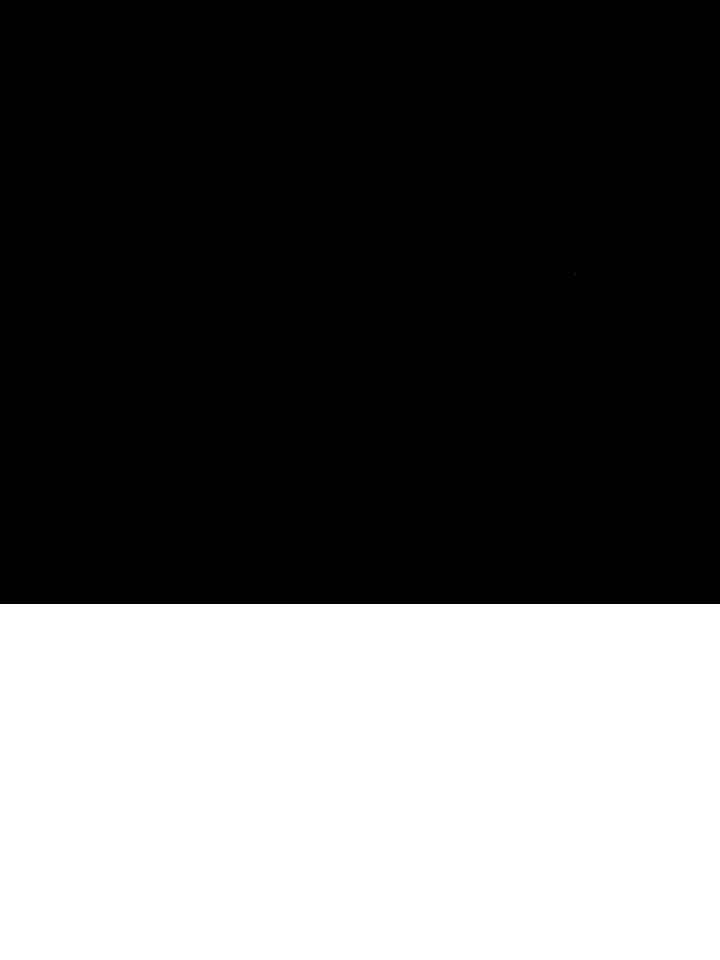

Supplement: Supplementary file 7 — Source data Fig. 4 [file 44321_2025_196_MOESM7_ESM.zip › MM-2024-19448_SourceDataForFig 4/MM-2024-19448_SourceDataForFig 4A/Apelin-dm Tamara.TIF]

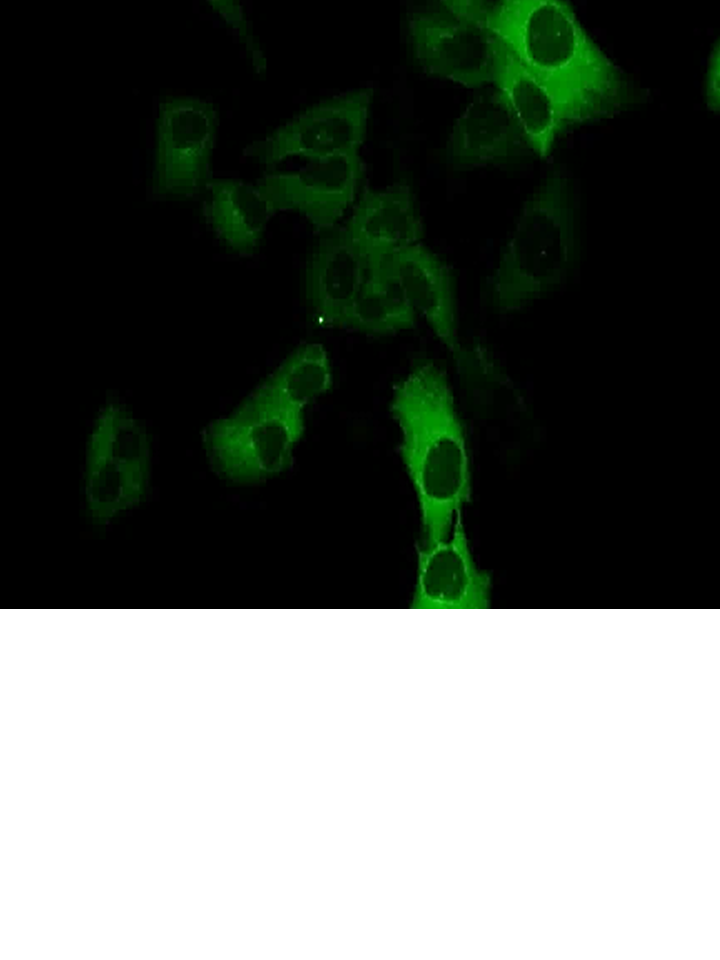

Supplement: Supplementary file 7 — Source data Fig. 4 [file 44321_2025_196_MOESM7_ESM.zip › MM-2024-19448_SourceDataForFig 4/MM-2024-19448_SourceDataForFig 4A/Control Arrestin.TIF]

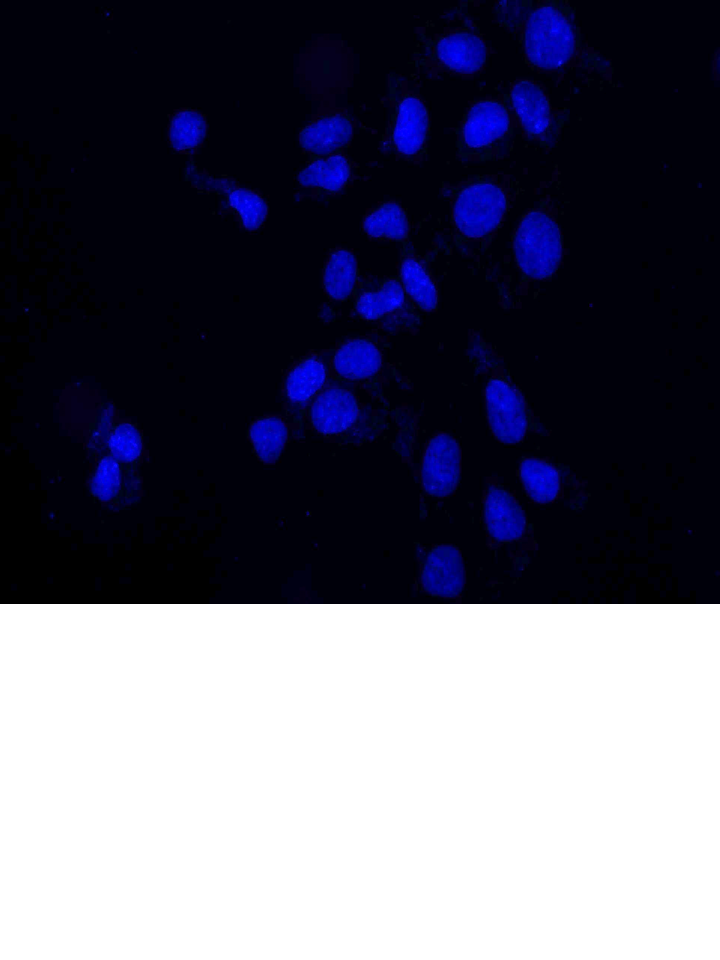

Supplement: Supplementary file 7 — Source data Fig. 4 [file 44321_2025_196_MOESM7_ESM.zip › MM-2024-19448_SourceDataForFig 4/MM-2024-19448_SourceDataForFig 4A/Control Dapi.TIF]

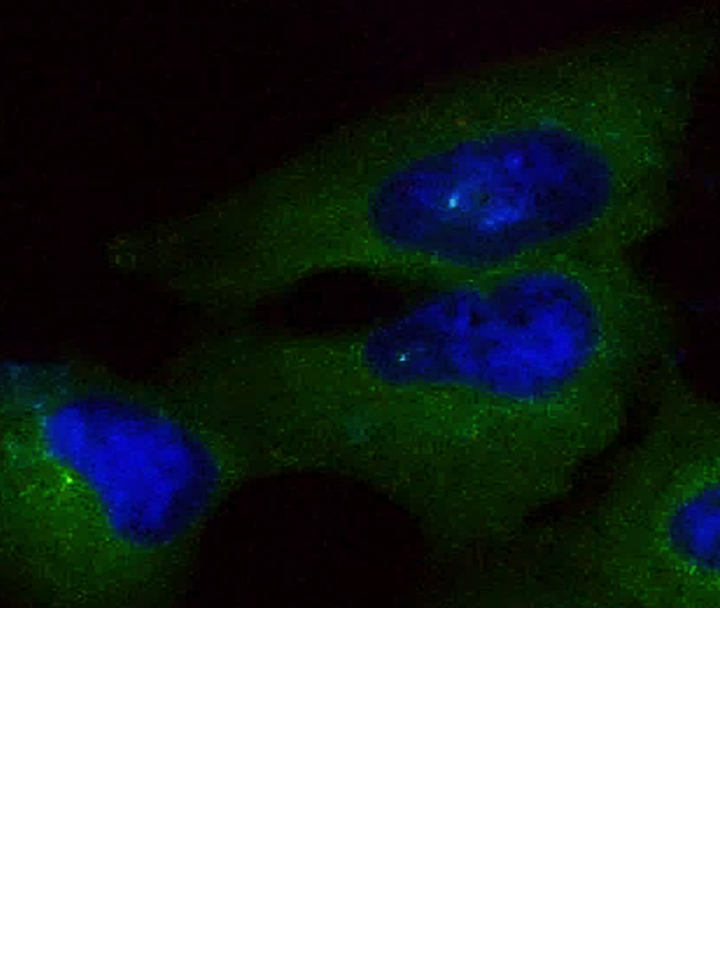

Supplement: Supplementary file 7 — Source data Fig. 4 [file 44321_2025_196_MOESM7_ESM.zip › MM-2024-19448_SourceDataForFig 4/MM-2024-19448_SourceDataForFig 4A/Control Merge zoom.TIF]

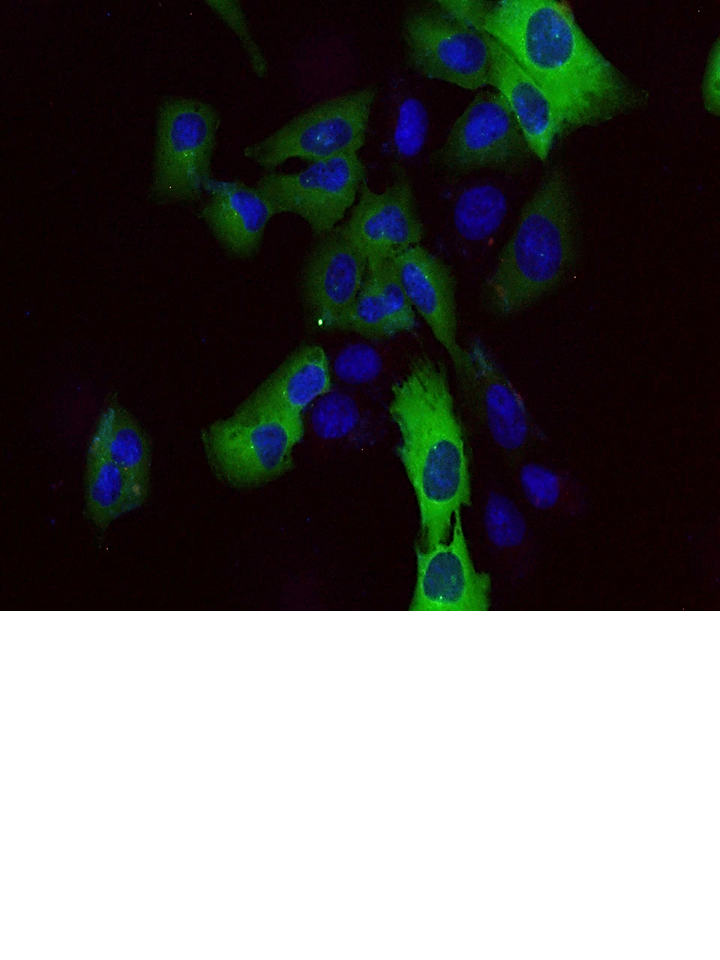

Supplement: Supplementary file 7 — Source data Fig. 4 [file 44321_2025_196_MOESM7_ESM.zip › MM-2024-19448_SourceDataForFig 4/MM-2024-19448_SourceDataForFig 4A/Control Merge.TIF]

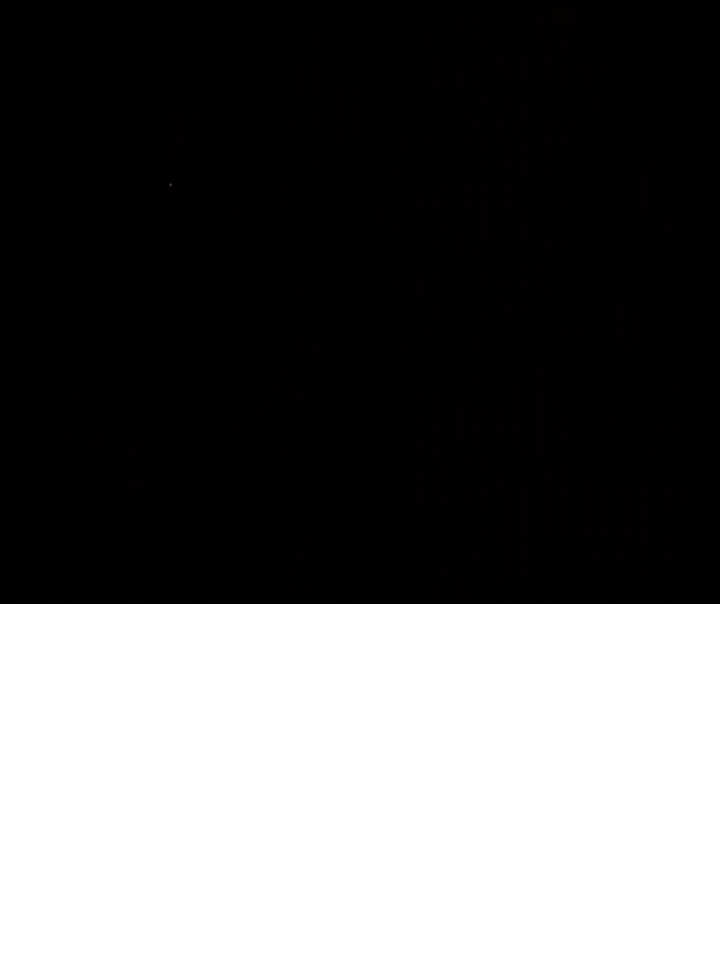

Supplement: Supplementary file 7 — Source data Fig. 4 [file 44321_2025_196_MOESM7_ESM.zip › MM-2024-19448_SourceDataForFig 4/MM-2024-19448_SourceDataForFig 4A/Control Tamara.TIF]

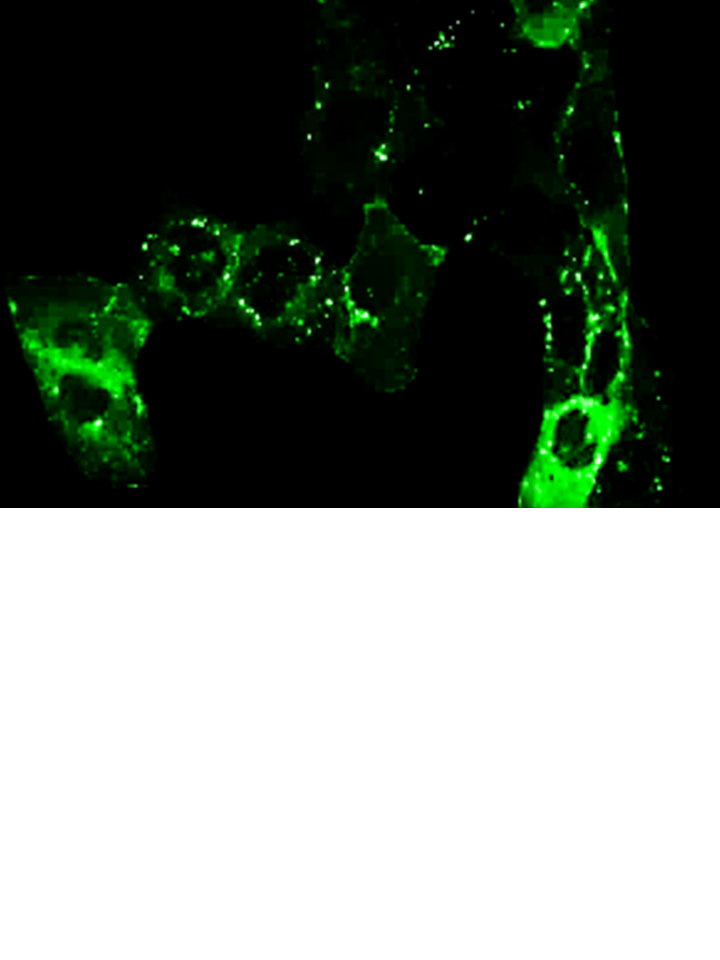

Supplement: Supplementary file 7 — Source data Fig. 4 [file 44321_2025_196_MOESM7_ESM.zip › MM-2024-19448_SourceDataForFig 4/MM-2024-19448_SourceDataForFig 4B/Arrestin Apelin.TIF]

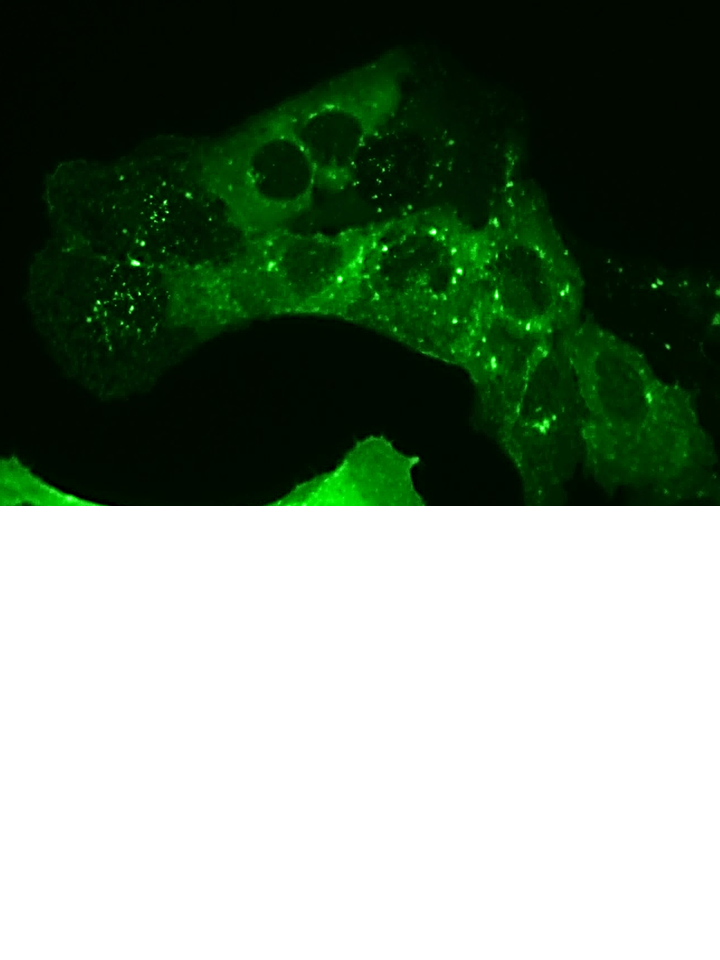

Supplement: Supplementary file 7 — Source data Fig. 4 [file 44321_2025_196_MOESM7_ESM.zip › MM-2024-19448_SourceDataForFig 4/MM-2024-19448_SourceDataForFig 4B/Arrestin Apelin-dm.TIF]

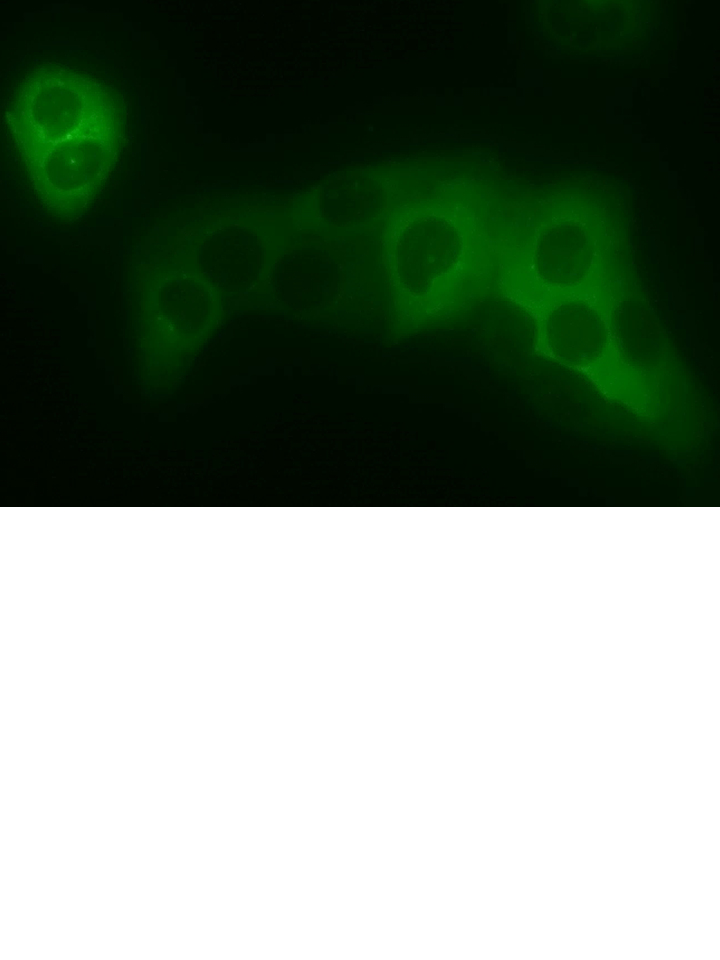

Supplement: Supplementary file 7 — Source data Fig. 4 [file 44321_2025_196_MOESM7_ESM.zip › MM-2024-19448_SourceDataForFig 4/MM-2024-19448_SourceDataForFig 4B/Arrestin Control.TIF]

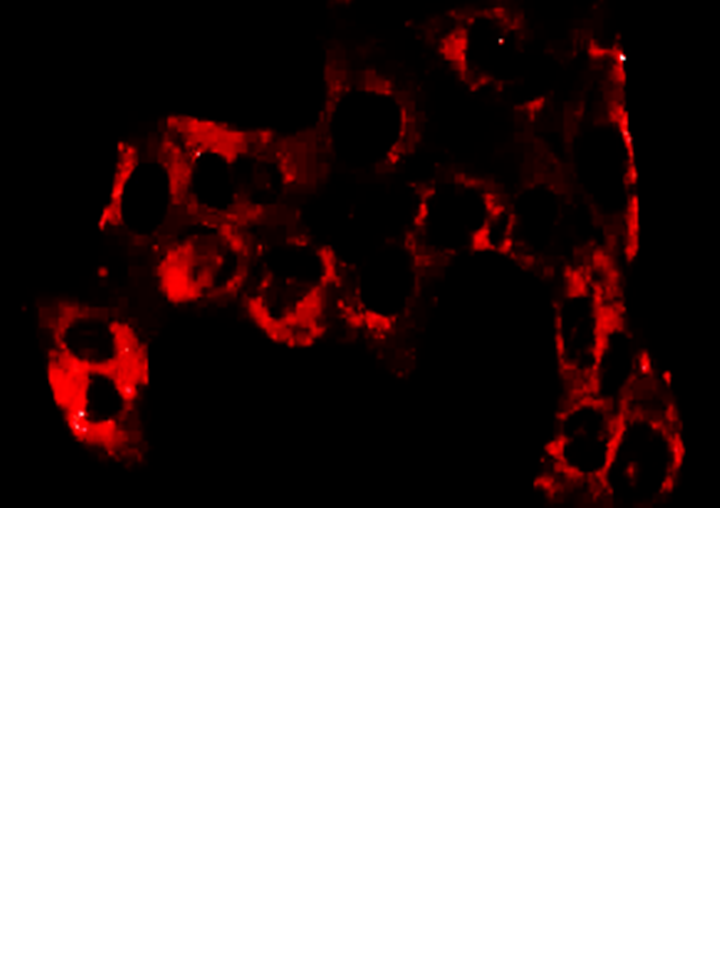

Supplement: Supplementary file 7 — Source data Fig. 4 [file 44321_2025_196_MOESM7_ESM.zip › MM-2024-19448_SourceDataForFig 4/MM-2024-19448_SourceDataForFig 4B/Clathrin Apelin.TIF]

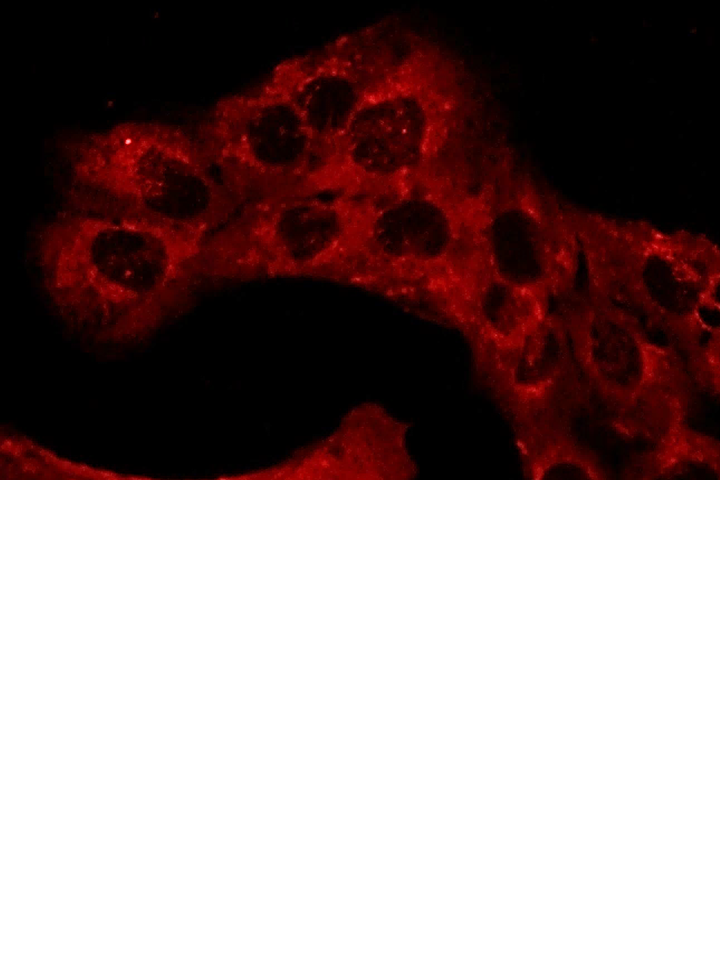

Supplement: Supplementary file 7 — Source data Fig. 4 [file 44321_2025_196_MOESM7_ESM.zip › MM-2024-19448_SourceDataForFig 4/MM-2024-19448_SourceDataForFig 4B/Clathrin Apelin-dm.TIF]
