# Supplementary figures and images for: Repression of apelin Furin cleavage sites provides antimetastatic strategy in colorectal cancer (part 2 of 2)
Source: EMBO Mol Med. 2025 Feb 17;17(3):504–34. doi: 10.1038/s44321-025-00196-5 (PMC11904221; doi:10.1038/s44321-025-00196-5)

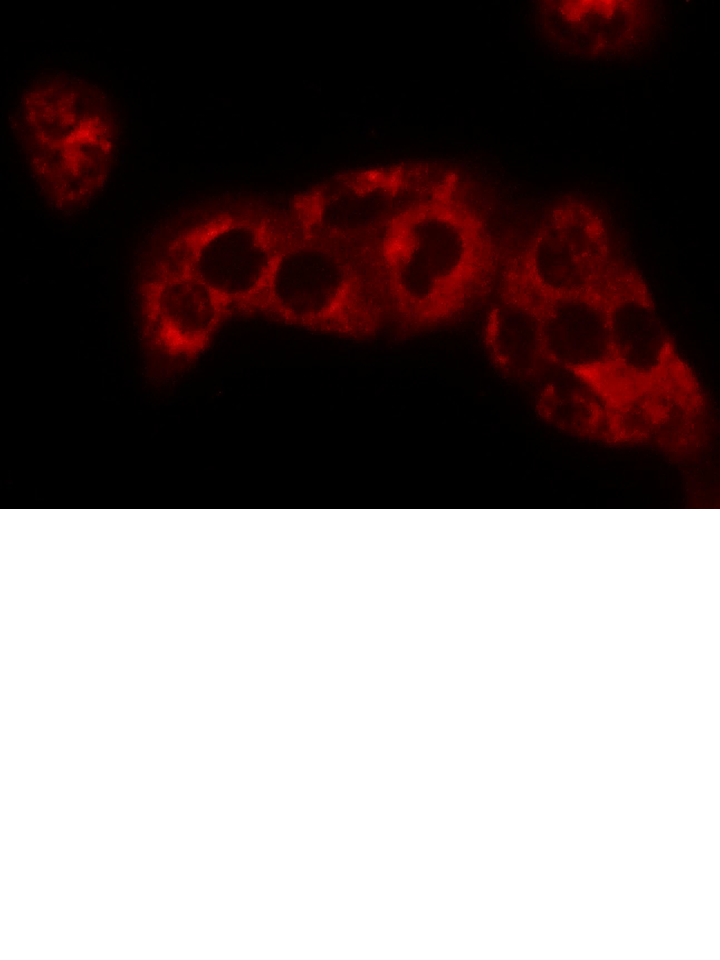

Supplement: Supplementary file 7 — Source data Fig. 4 [file 44321_2025_196_MOESM7_ESM.zip › MM-2024-19448_SourceDataForFig 4/MM-2024-19448_SourceDataForFig 4B/Clathrin Control.TIF]

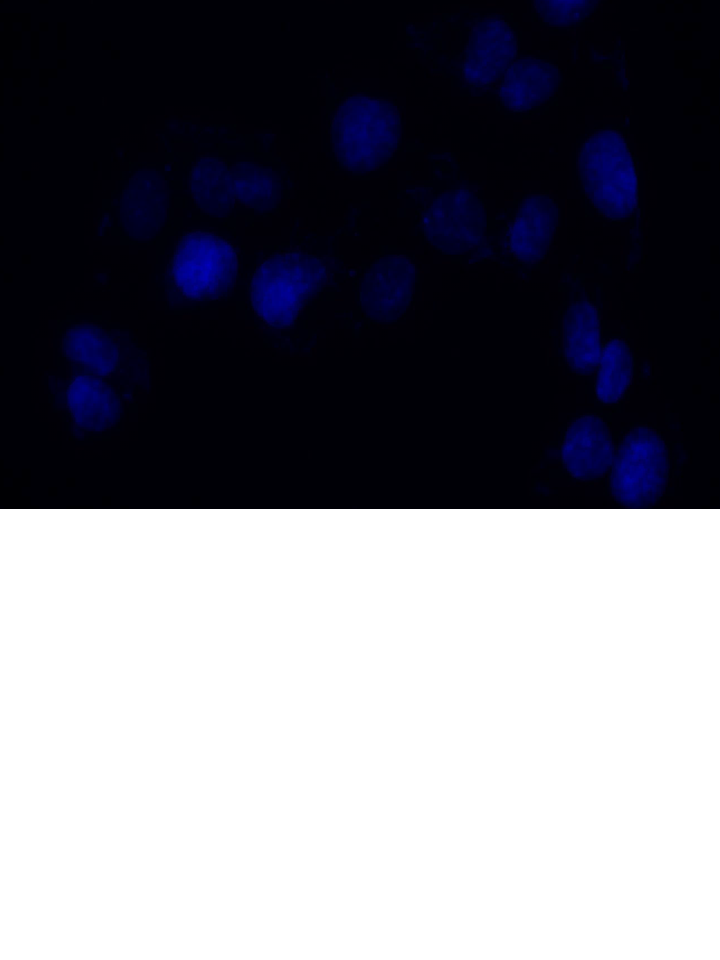

Supplement: Supplementary file 7 — Source data Fig. 4 [file 44321_2025_196_MOESM7_ESM.zip › MM-2024-19448_SourceDataForFig 4/MM-2024-19448_SourceDataForFig 4B/Dapi Apelin.TIF]

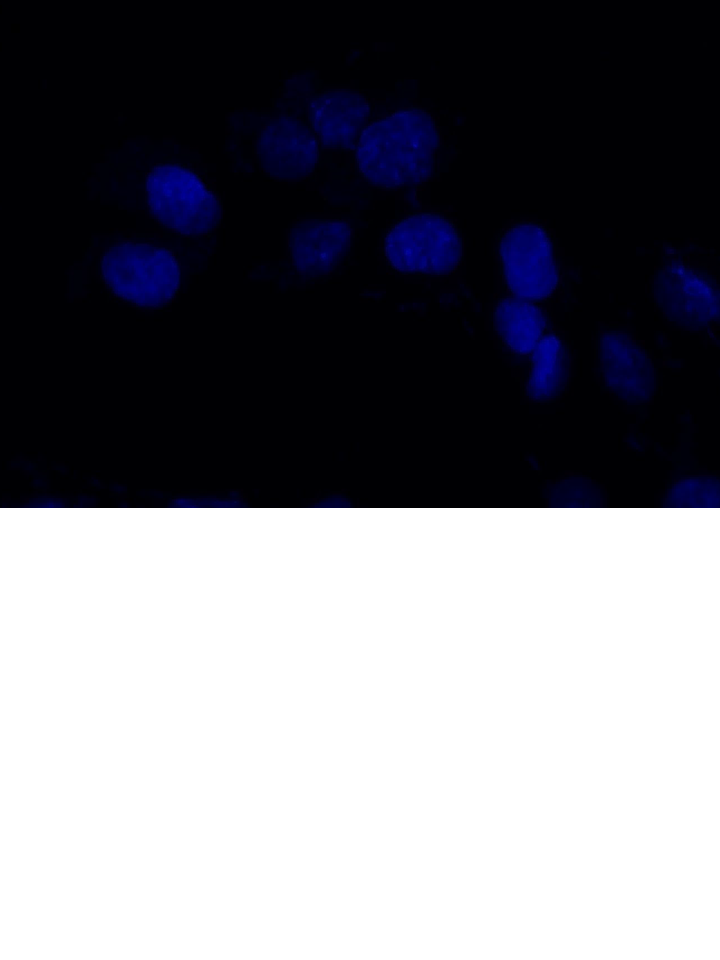

Supplement: Supplementary file 7 — Source data Fig. 4 [file 44321_2025_196_MOESM7_ESM.zip › MM-2024-19448_SourceDataForFig 4/MM-2024-19448_SourceDataForFig 4B/Dapi Apelin-dm.TIF]

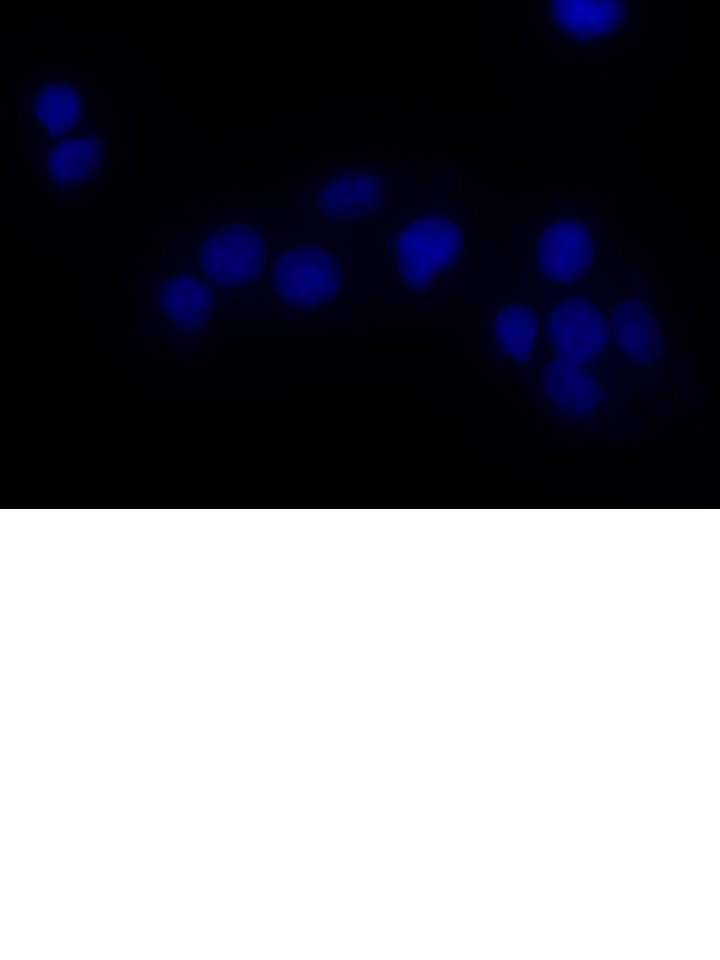

Supplement: Supplementary file 7 — Source data Fig. 4 [file 44321_2025_196_MOESM7_ESM.zip › MM-2024-19448_SourceDataForFig 4/MM-2024-19448_SourceDataForFig 4B/Dapi Control.TIF]

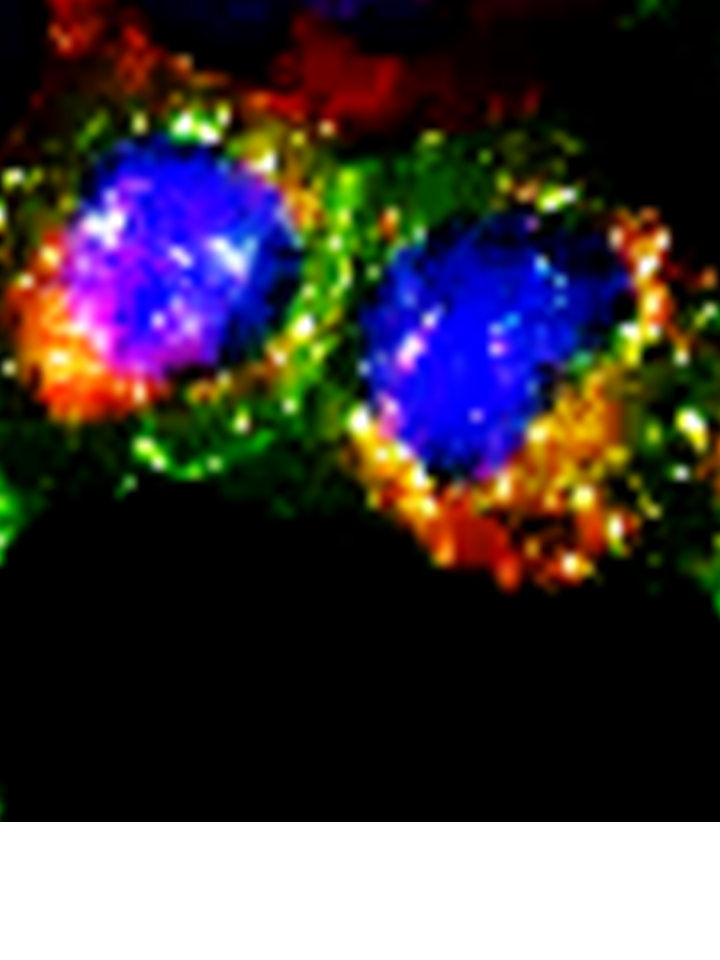

Supplement: Supplementary file 7 — Source data Fig. 4 [file 44321_2025_196_MOESM7_ESM.zip › MM-2024-19448_SourceDataForFig 4/MM-2024-19448_SourceDataForFig 4B/Merge Apelin Zoom.TIF]

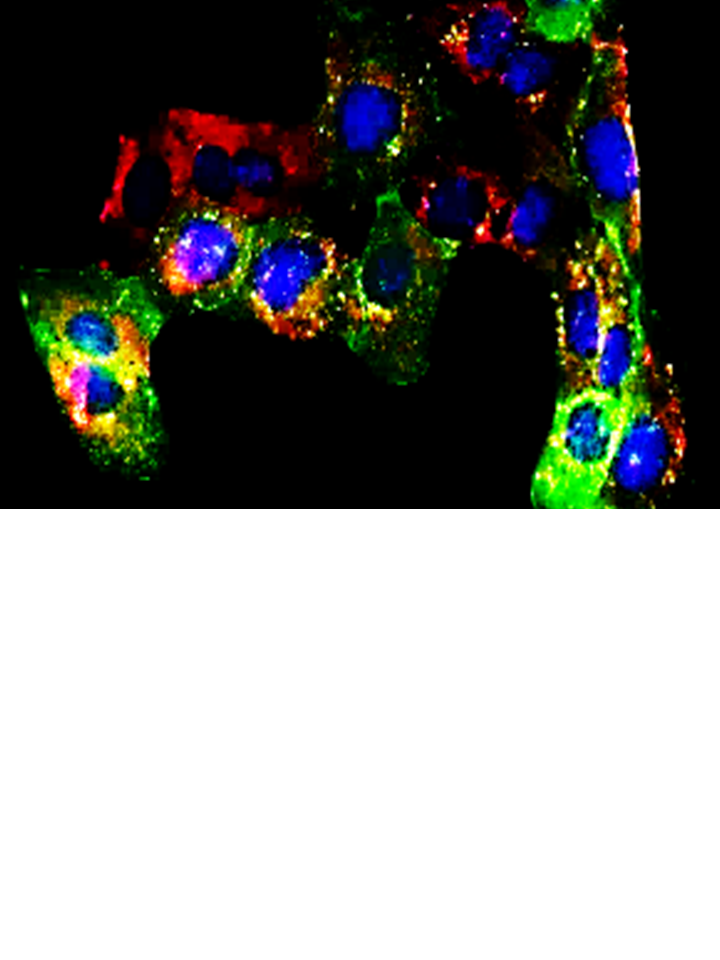

Supplement: Supplementary file 7 — Source data Fig. 4 [file 44321_2025_196_MOESM7_ESM.zip › MM-2024-19448_SourceDataForFig 4/MM-2024-19448_SourceDataForFig 4B/Merge Apelin.TIF]

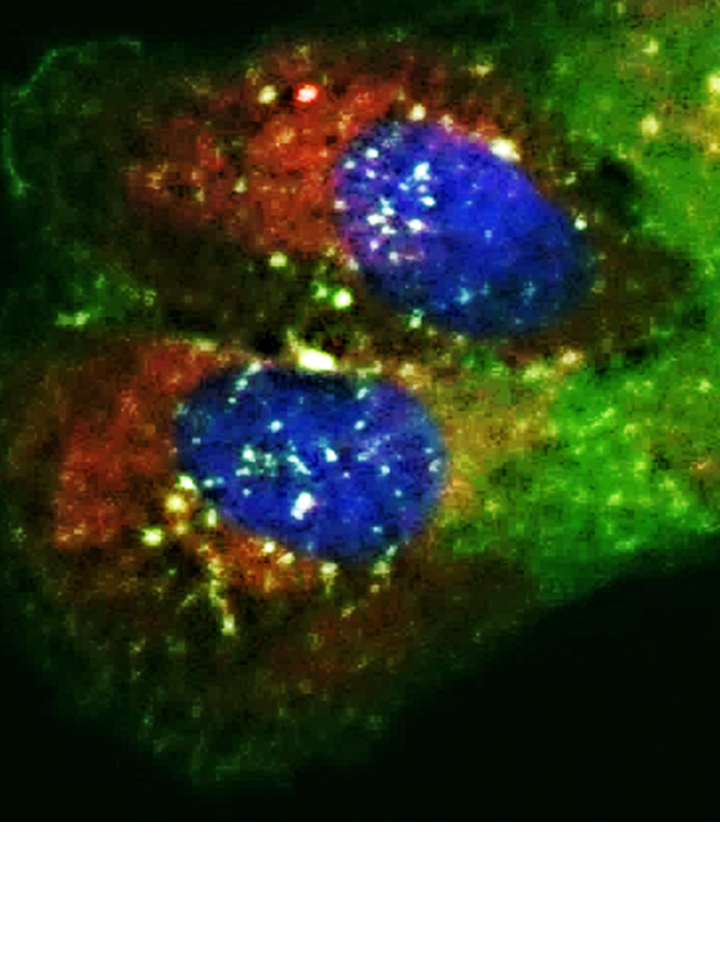

Supplement: Supplementary file 7 — Source data Fig. 4 [file 44321_2025_196_MOESM7_ESM.zip › MM-2024-19448_SourceDataForFig 4/MM-2024-19448_SourceDataForFig 4B/Merge Apelin-dm Zoom.TIF]

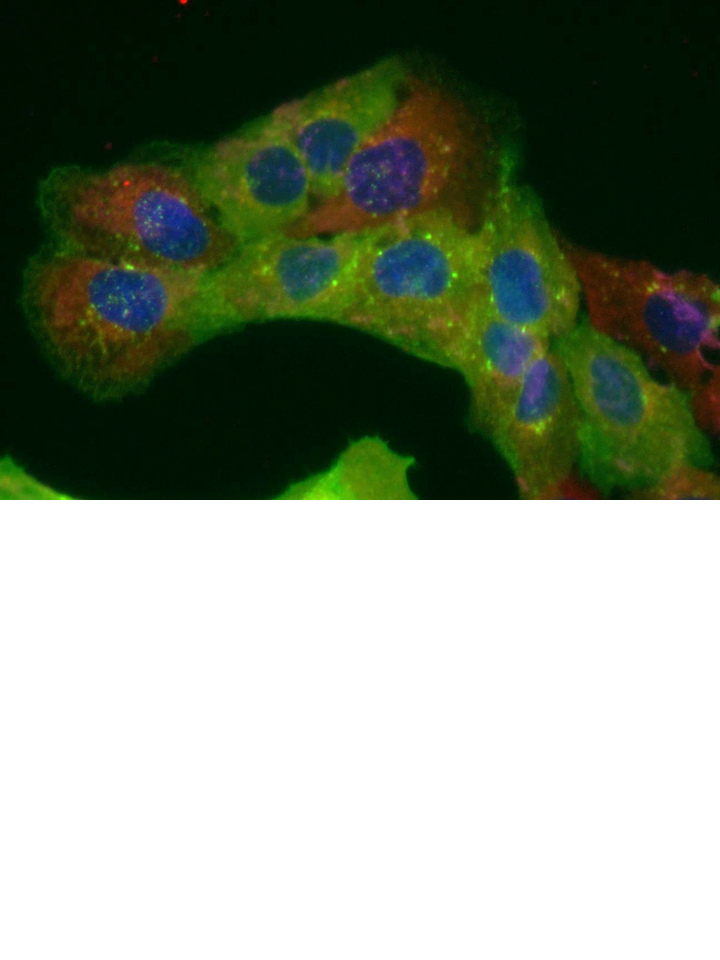

Supplement: Supplementary file 7 — Source data Fig. 4 [file 44321_2025_196_MOESM7_ESM.zip › MM-2024-19448_SourceDataForFig 4/MM-2024-19448_SourceDataForFig 4B/Merge Apelin-dm.TIF]

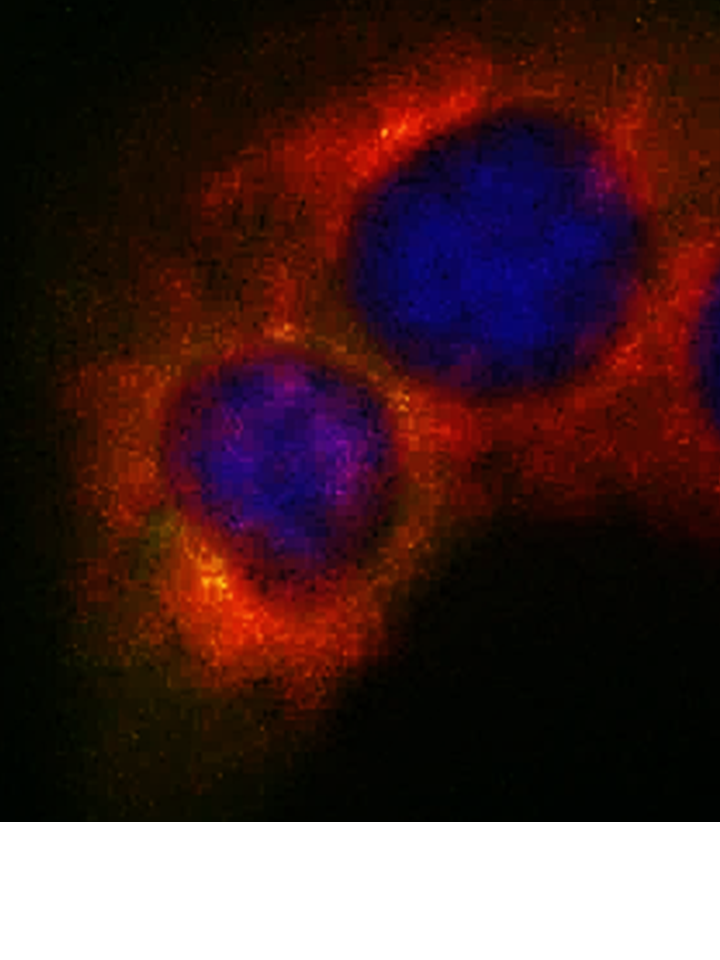

Supplement: Supplementary file 7 — Source data Fig. 4 [file 44321_2025_196_MOESM7_ESM.zip › MM-2024-19448_SourceDataForFig 4/MM-2024-19448_SourceDataForFig 4B/Merge Control Zoom.TIF]

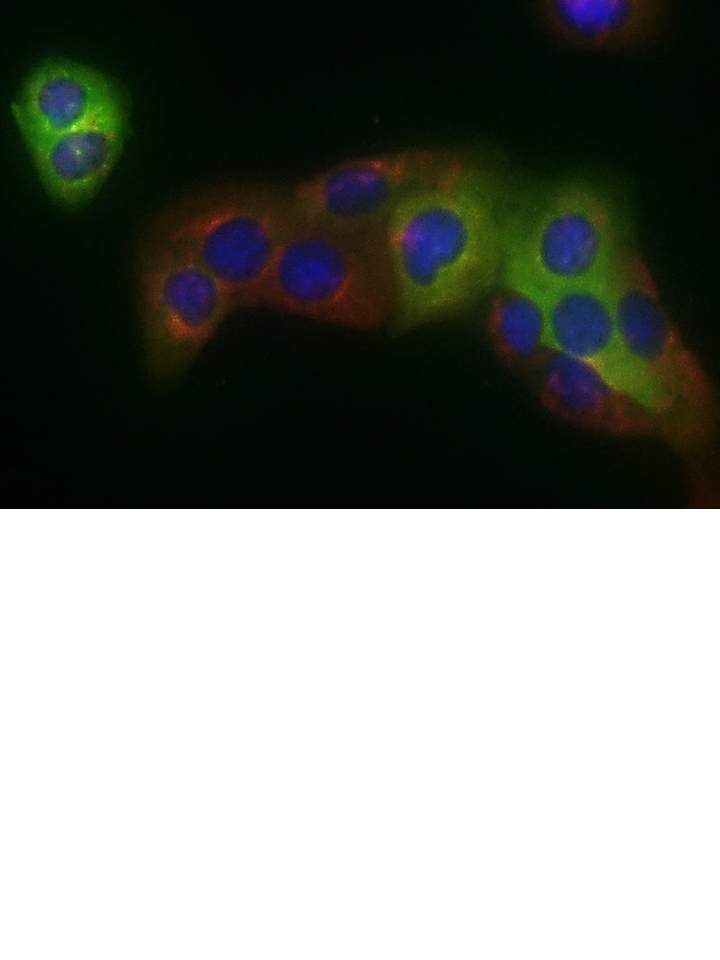

Supplement: Supplementary file 7 — Source data Fig. 4 [file 44321_2025_196_MOESM7_ESM.zip › MM-2024-19448_SourceDataForFig 4/MM-2024-19448_SourceDataForFig 4B/Merge Control.TIF]

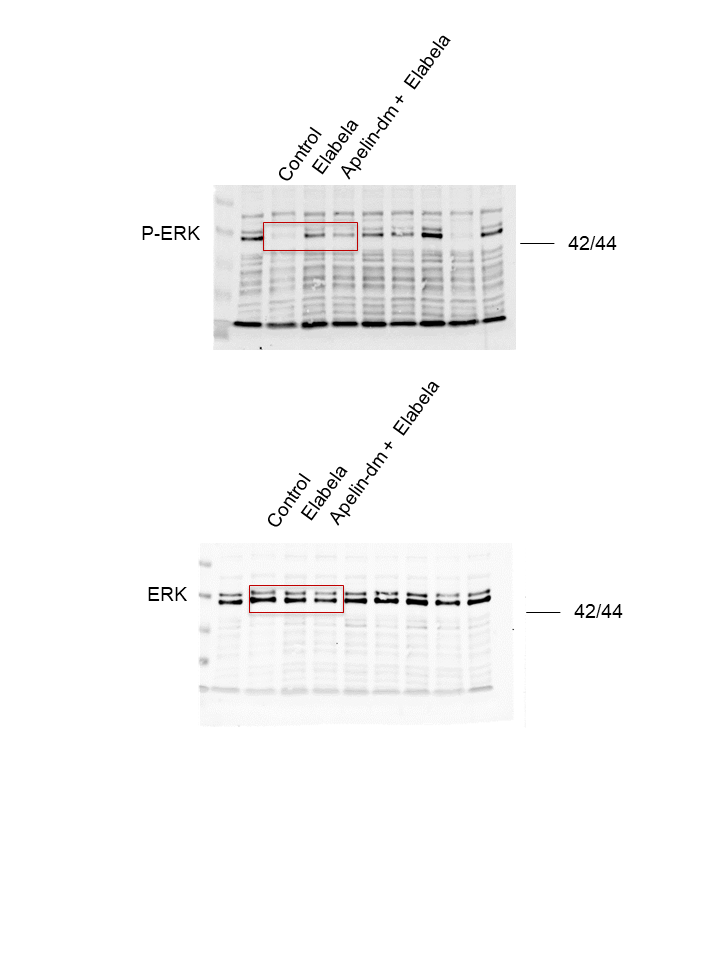

Supplement: Supplementary file 7 — Source data Fig. 4 [file 44321_2025_196_MOESM7_ESM.zip › MM-2024-19448_SourceDataForFig 4/MM-2024-19448_SourceDataForFig 4E.TIF]

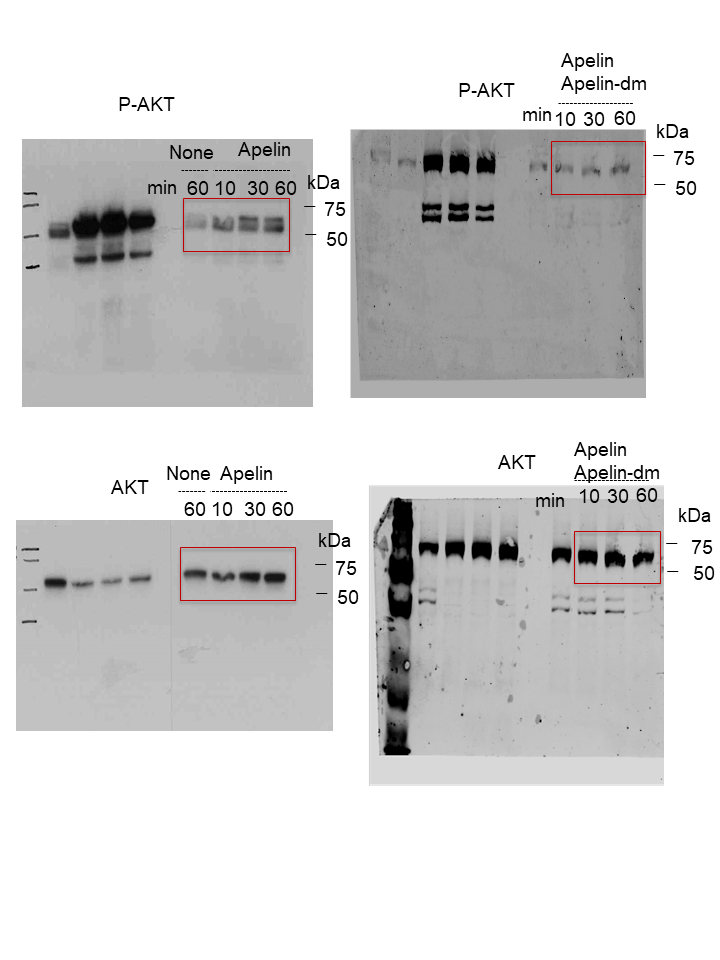

Supplement: Supplementary file 7 — Source data Fig. 4 [file 44321_2025_196_MOESM7_ESM.zip › MM-2024-19448_SourceDataForFig 4/MM-2024-19448_SourceDataForFig 4F.tif]

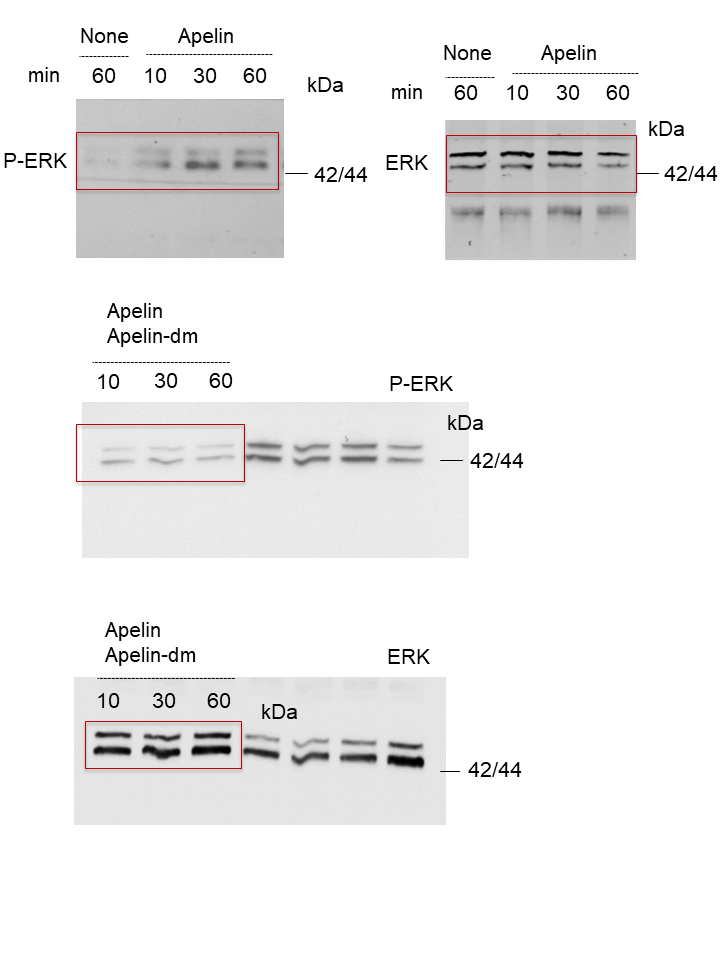

Supplement: Supplementary file 7 — Source data Fig. 4 [file 44321_2025_196_MOESM7_ESM.zip › MM-2024-19448_SourceDataForFig 4/MM-2024-19448_SourceDataForFig 4Gnew.tif]

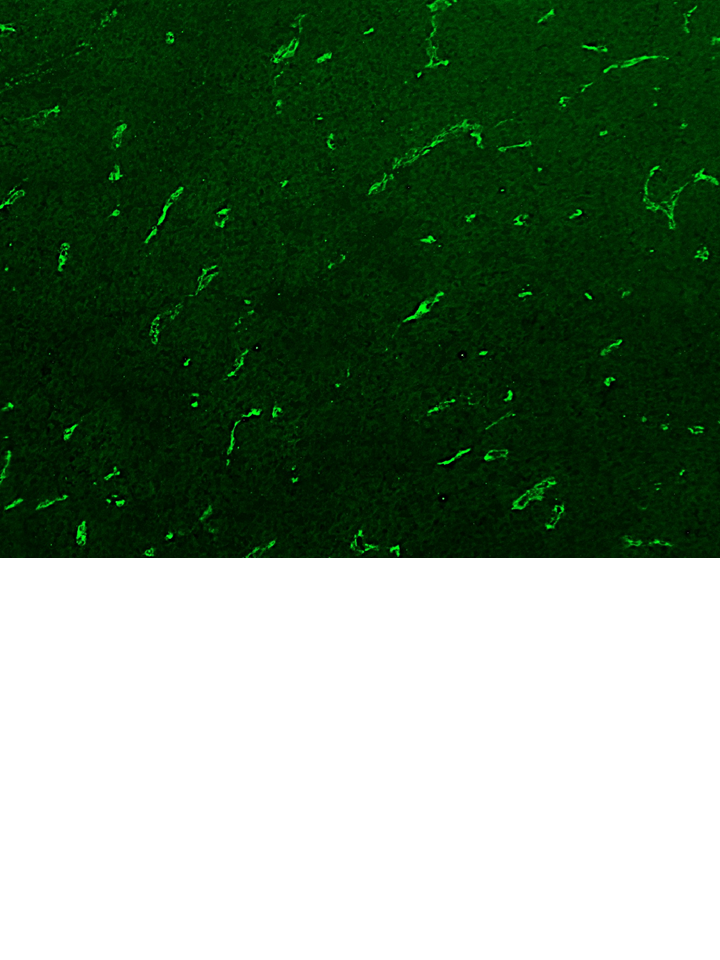

Supplement: Supplementary file 8 — Source data Fig. 5 [file 44321_2025_196_MOESM8_ESM.zip › MM-2024-19448_SourceDataForFig 5/MM-2024-19448_SourceDataForFig 5/MM-2024-19448_SourceDataForFig 5E/CD31 Apln-dm CT-26.TIF]

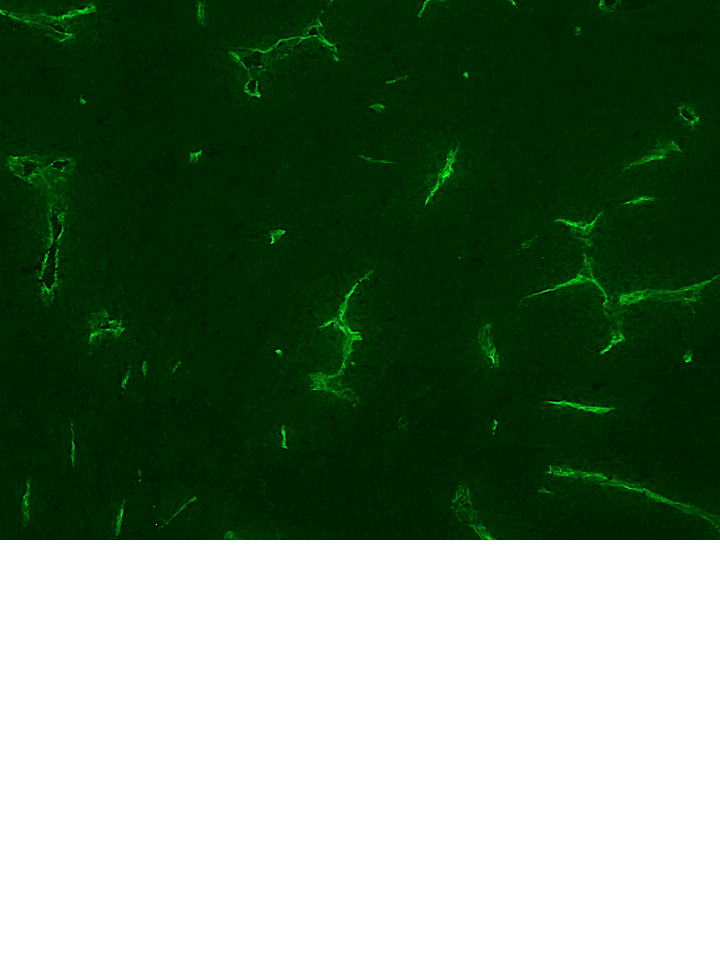

Supplement: Supplementary file 8 — Source data Fig. 5 [file 44321_2025_196_MOESM8_ESM.zip › MM-2024-19448_SourceDataForFig 5/MM-2024-19448_SourceDataForFig 5/MM-2024-19448_SourceDataForFig 5E/CD31 Apln-dm MC-38.TIF]

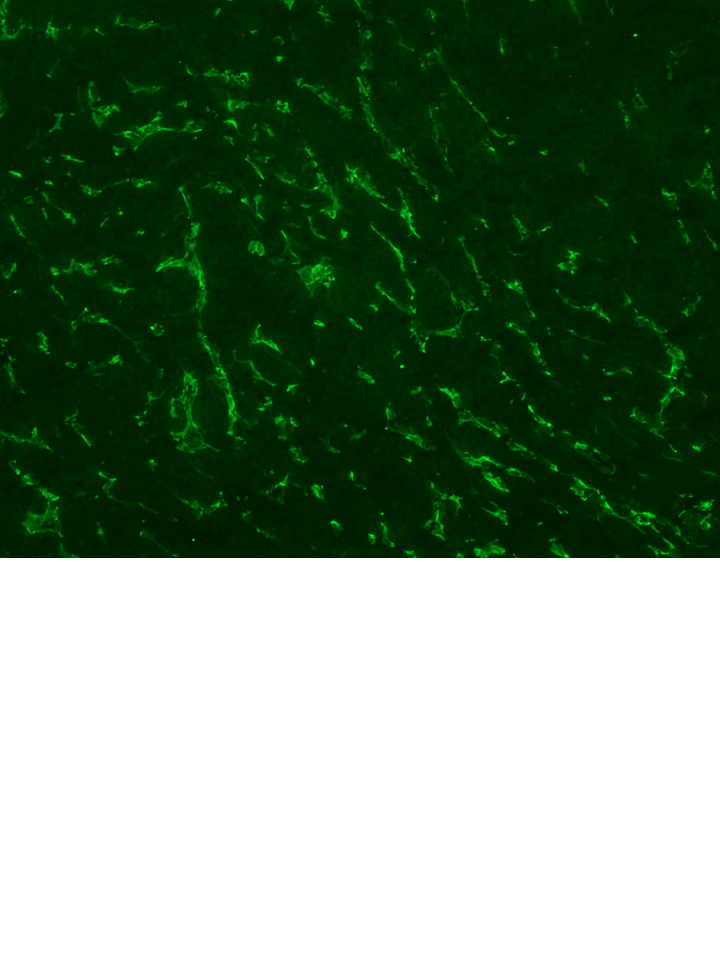

Supplement: Supplementary file 8 — Source data Fig. 5 [file 44321_2025_196_MOESM8_ESM.zip › MM-2024-19448_SourceDataForFig 5/MM-2024-19448_SourceDataForFig 5/MM-2024-19448_SourceDataForFig 5E/CD31 Control CT-26.TIF]

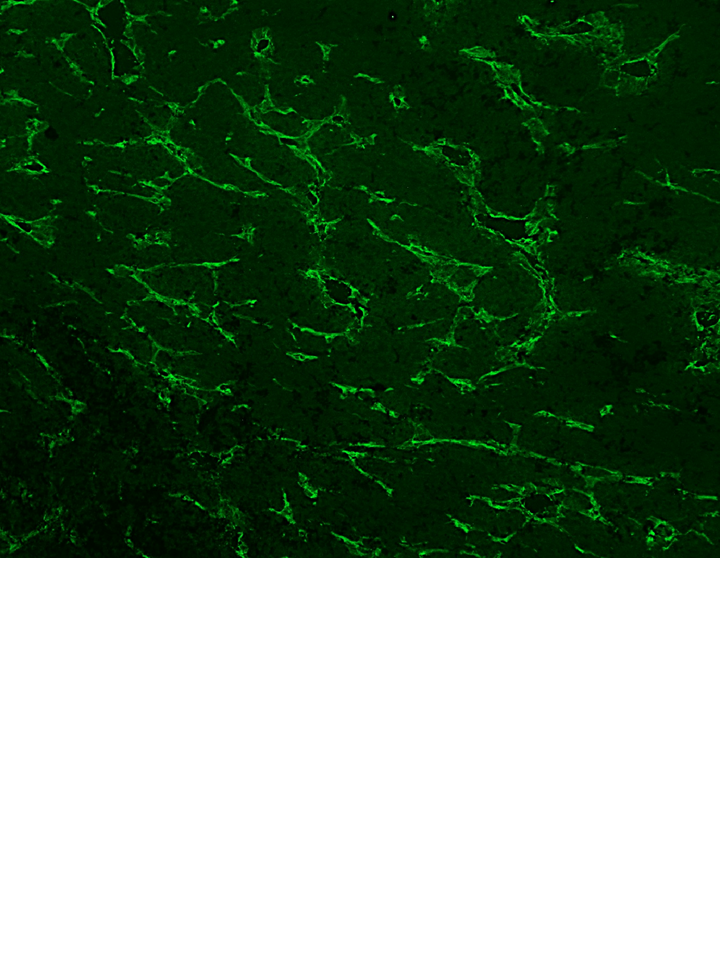

Supplement: Supplementary file 8 — Source data Fig. 5 [file 44321_2025_196_MOESM8_ESM.zip › MM-2024-19448_SourceDataForFig 5/MM-2024-19448_SourceDataForFig 5/MM-2024-19448_SourceDataForFig 5E/CD31 Control MC-38.TIF]

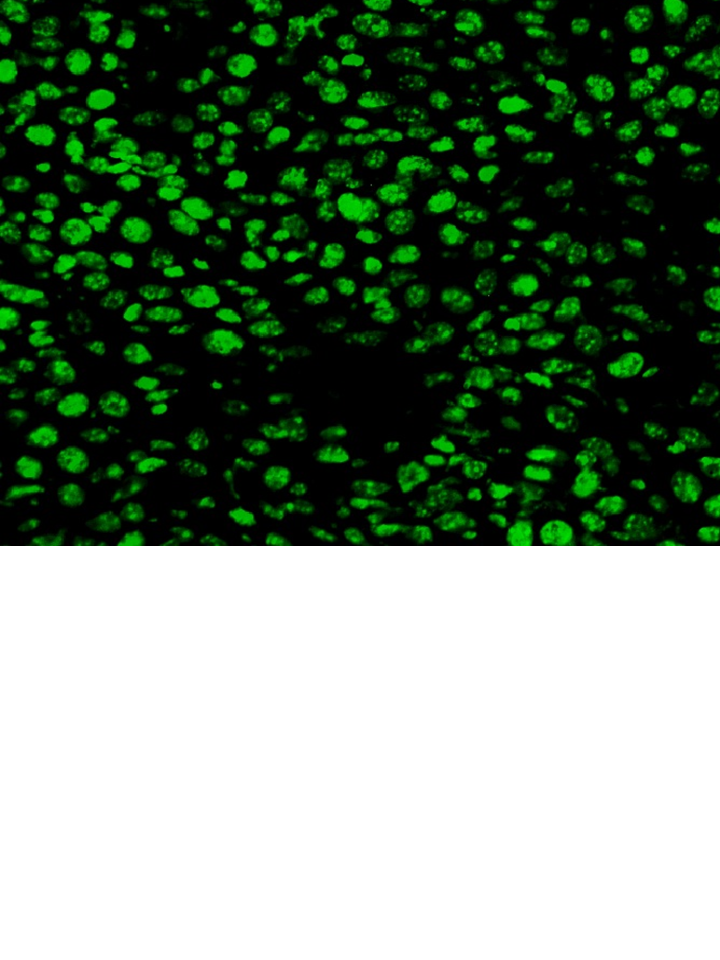

Supplement: Supplementary file 8 — Source data Fig. 5 [file 44321_2025_196_MOESM8_ESM.zip › MM-2024-19448_SourceDataForFig 5/MM-2024-19448_SourceDataForFig 5/MM-2024-19448_SourceDataForFig 5F/KI67 Apln-dm CT-26.TIF]

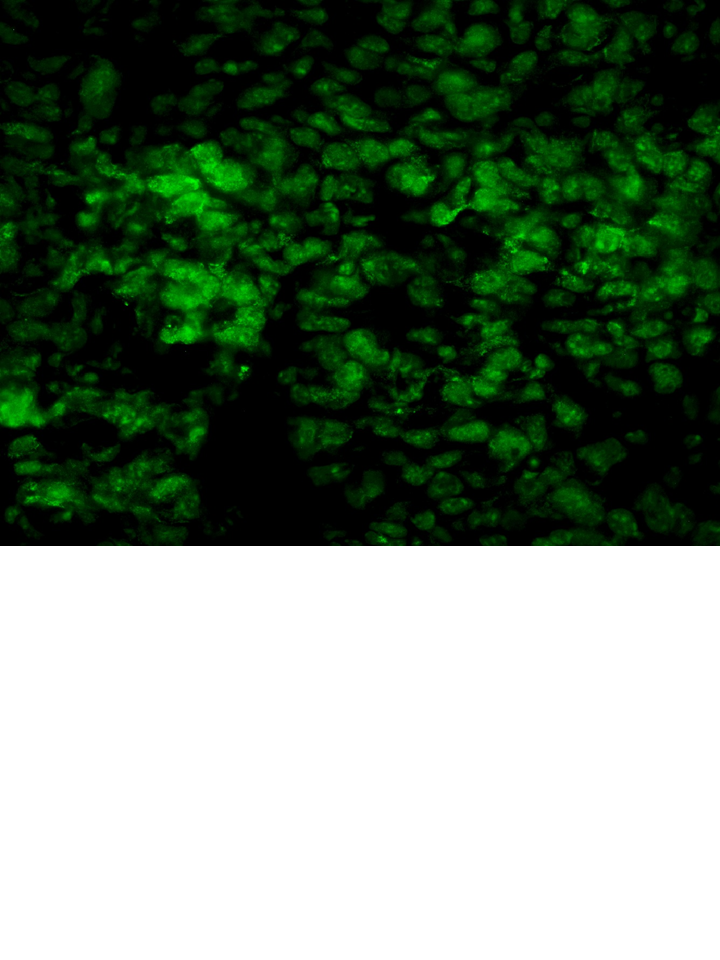

Supplement: Supplementary file 8 — Source data Fig. 5 [file 44321_2025_196_MOESM8_ESM.zip › MM-2024-19448_SourceDataForFig 5/MM-2024-19448_SourceDataForFig 5/MM-2024-19448_SourceDataForFig 5F/KI67 Apln-dm MC-38.TIF]

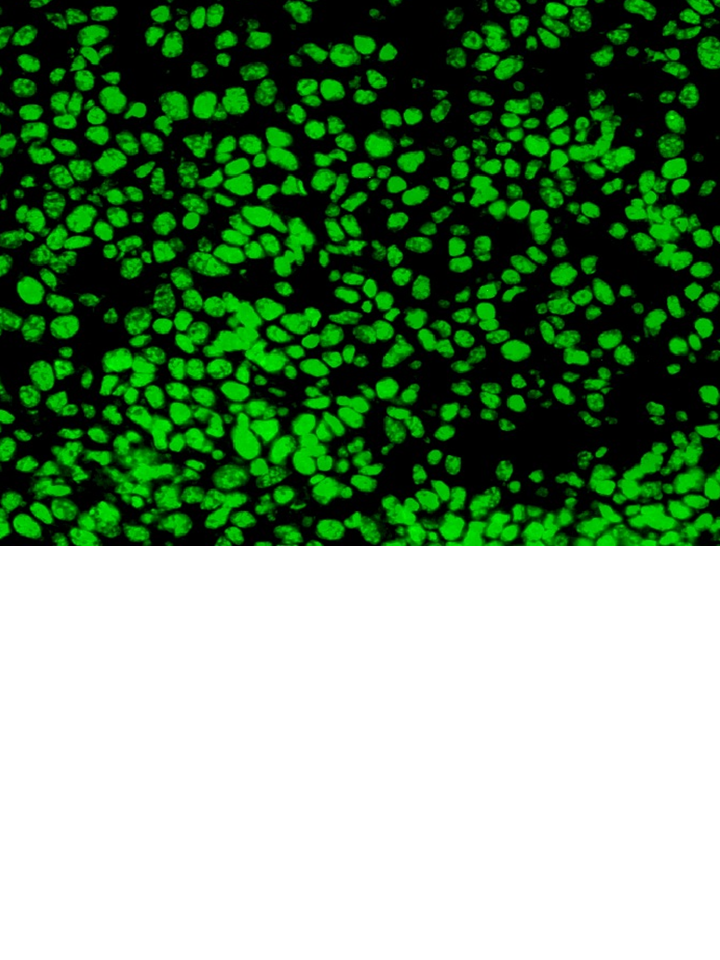

Supplement: Supplementary file 8 — Source data Fig. 5 [file 44321_2025_196_MOESM8_ESM.zip › MM-2024-19448_SourceDataForFig 5/MM-2024-19448_SourceDataForFig 5/MM-2024-19448_SourceDataForFig 5F/KI67 Control CT-26.TIF]

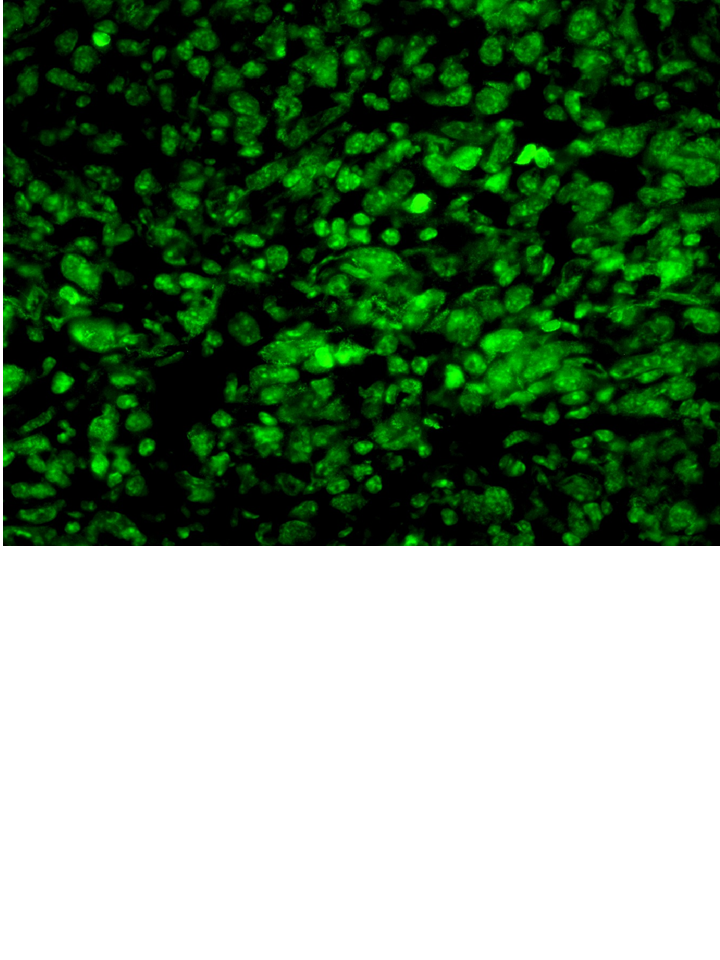

Supplement: Supplementary file 8 — Source data Fig. 5 [file 44321_2025_196_MOESM8_ESM.zip › MM-2024-19448_SourceDataForFig 5/MM-2024-19448_SourceDataForFig 5/MM-2024-19448_SourceDataForFig 5F/KI67 Control MC-38.TIF]

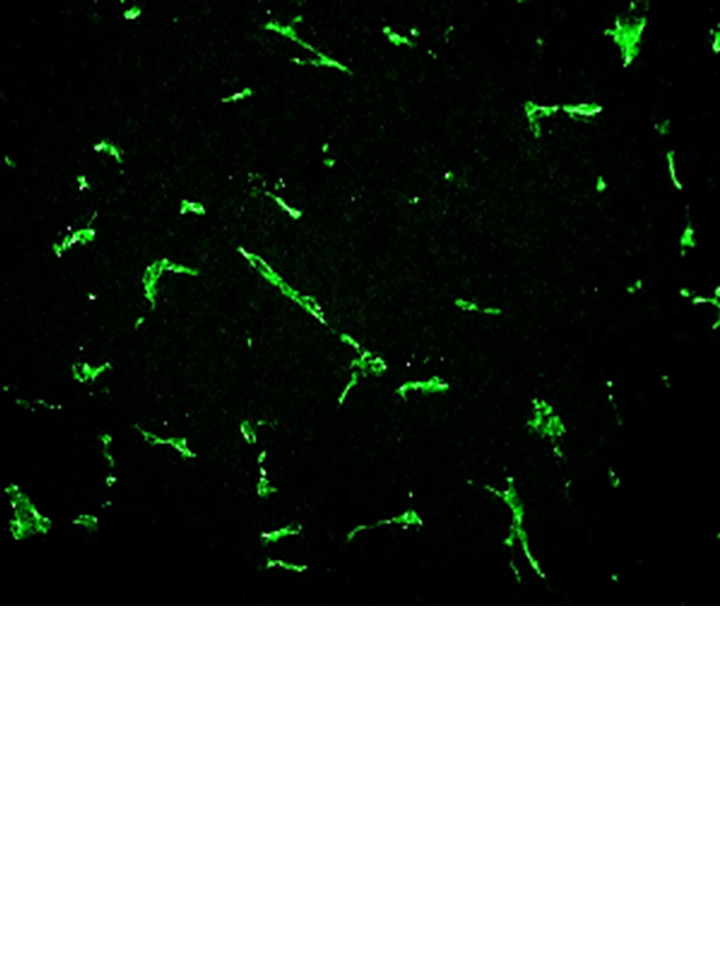

Supplement: Supplementary file 8 — Source data Fig. 5 [file 44321_2025_196_MOESM8_ESM.zip › MM-2024-19448_SourceDataForFig 5/MM-2024-19448_SourceDataForFig 5/MM-2024-19448_SourceDataForFig 5H/CT-26 CD31 Apelin-dm.TIF]

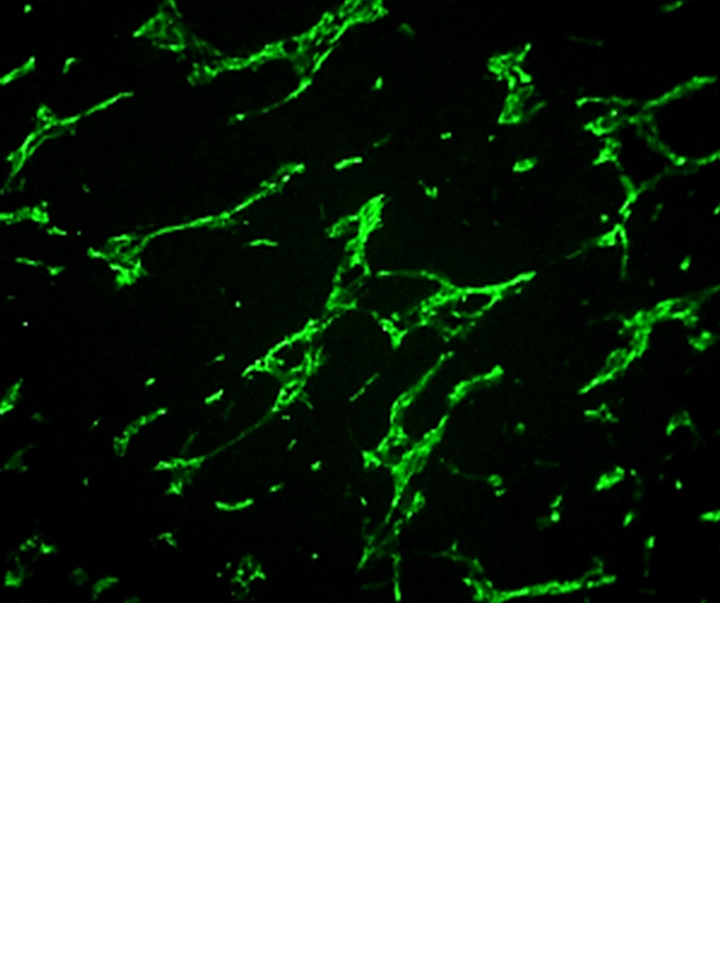

Supplement: Supplementary file 8 — Source data Fig. 5 [file 44321_2025_196_MOESM8_ESM.zip › MM-2024-19448_SourceDataForFig 5/MM-2024-19448_SourceDataForFig 5/MM-2024-19448_SourceDataForFig 5H/CT-26 CD31 Control.TIF]

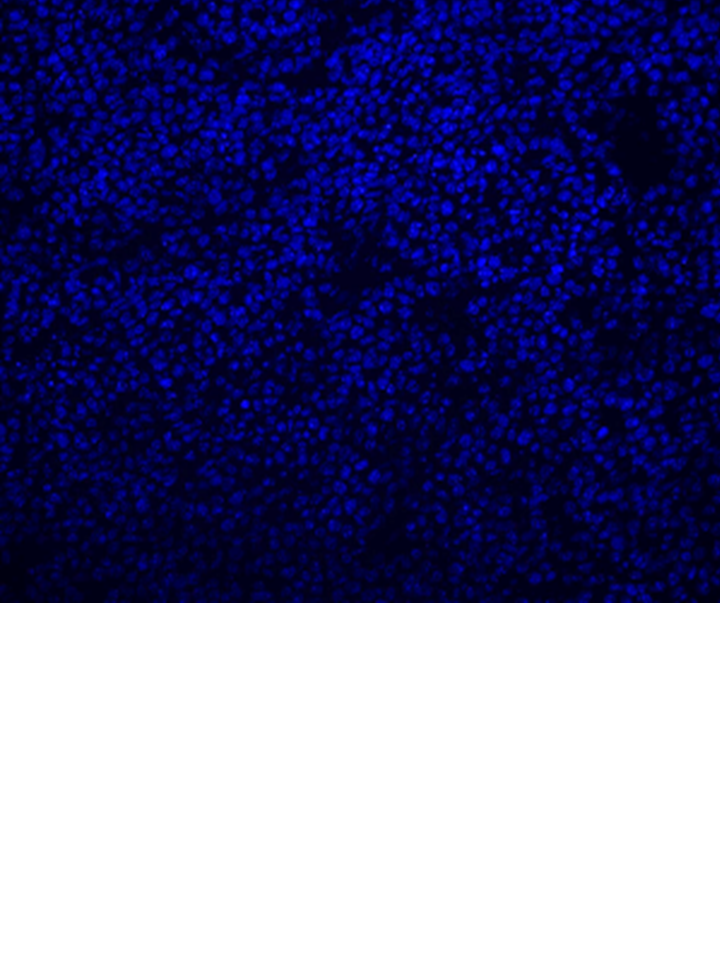

Supplement: Supplementary file 8 — Source data Fig. 5 [file 44321_2025_196_MOESM8_ESM.zip › MM-2024-19448_SourceDataForFig 5/MM-2024-19448_SourceDataForFig 5/MM-2024-19448_SourceDataForFig 5H/CT-26 Dapi Apelin-dm.TIF]

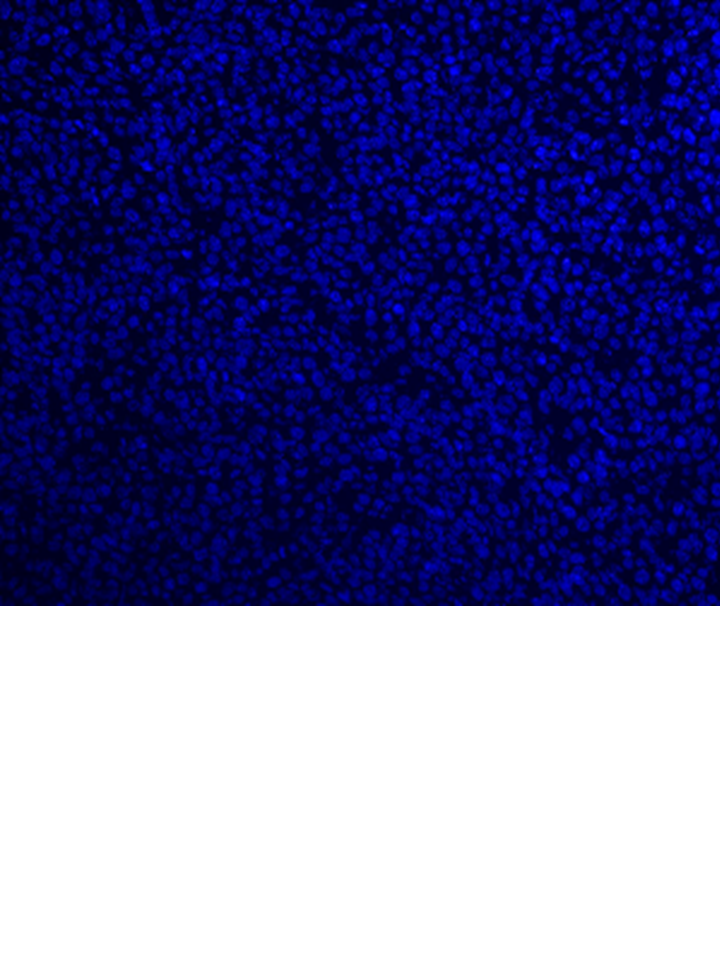

Supplement: Supplementary file 8 — Source data Fig. 5 [file 44321_2025_196_MOESM8_ESM.zip › MM-2024-19448_SourceDataForFig 5/MM-2024-19448_SourceDataForFig 5/MM-2024-19448_SourceDataForFig 5H/CT-26 Dapi Control.TIF]

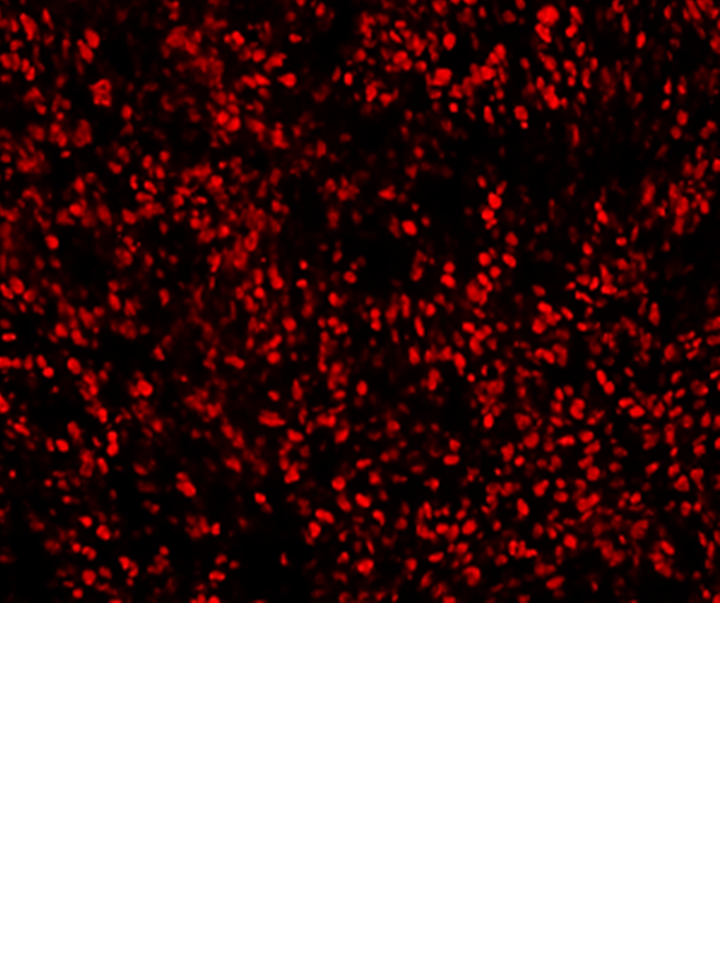

Supplement: Supplementary file 8 — Source data Fig. 5 [file 44321_2025_196_MOESM8_ESM.zip › MM-2024-19448_SourceDataForFig 5/MM-2024-19448_SourceDataForFig 5/MM-2024-19448_SourceDataForFig 5H/CT-26 KI67 Apelin-dm.TIF]

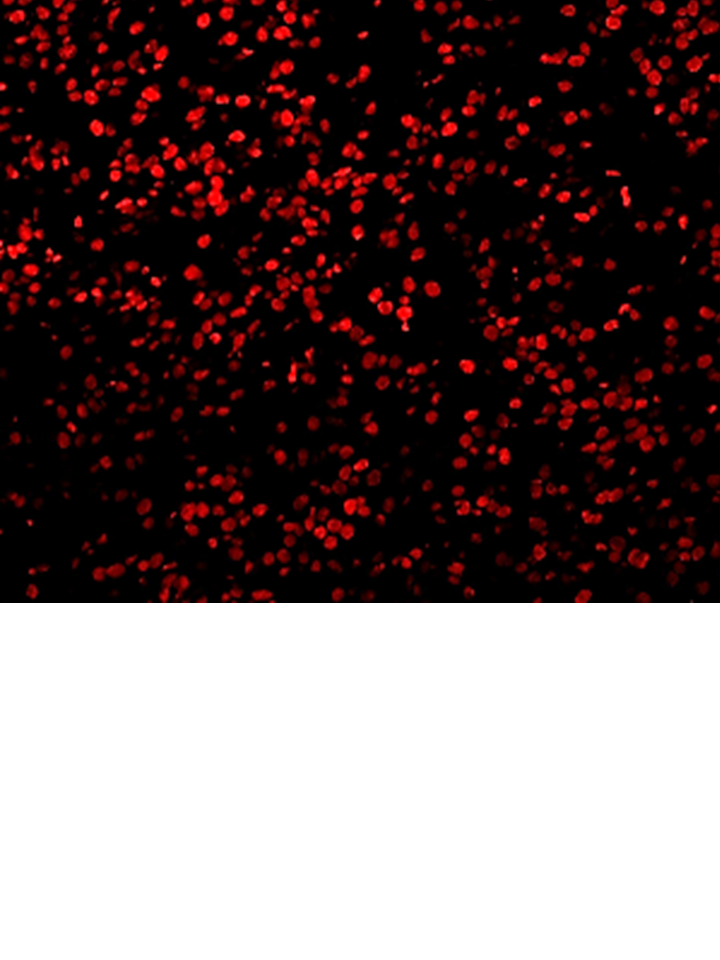

Supplement: Supplementary file 8 — Source data Fig. 5 [file 44321_2025_196_MOESM8_ESM.zip › MM-2024-19448_SourceDataForFig 5/MM-2024-19448_SourceDataForFig 5/MM-2024-19448_SourceDataForFig 5H/CT-26 KI67 CD31 Control.TIF]

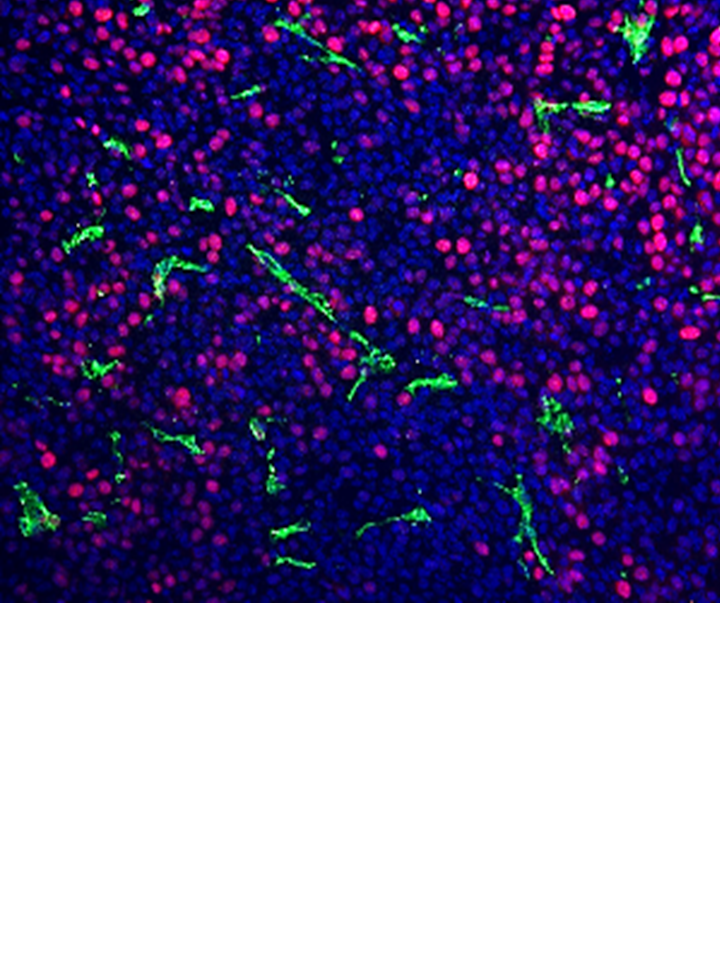

Supplement: Supplementary file 8 — Source data Fig. 5 [file 44321_2025_196_MOESM8_ESM.zip › MM-2024-19448_SourceDataForFig 5/MM-2024-19448_SourceDataForFig 5/MM-2024-19448_SourceDataForFig 5H/CT-26 Merge Apelin-dm.TIF]

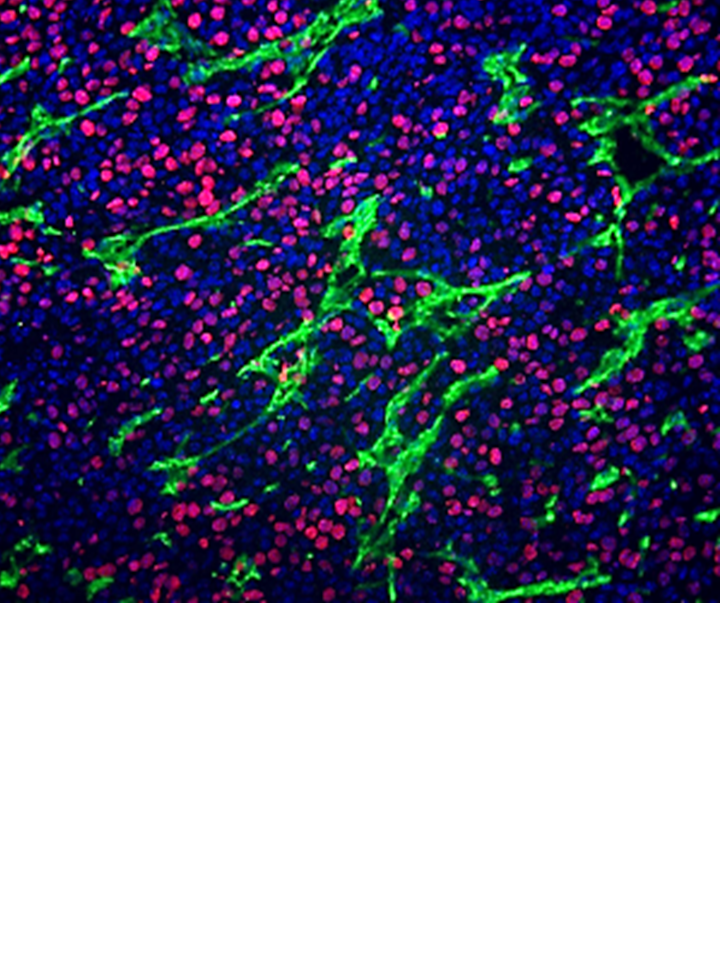

Supplement: Supplementary file 8 — Source data Fig. 5 [file 44321_2025_196_MOESM8_ESM.zip › MM-2024-19448_SourceDataForFig 5/MM-2024-19448_SourceDataForFig 5/MM-2024-19448_SourceDataForFig 5H/CT-26 Merge Control.TIF]

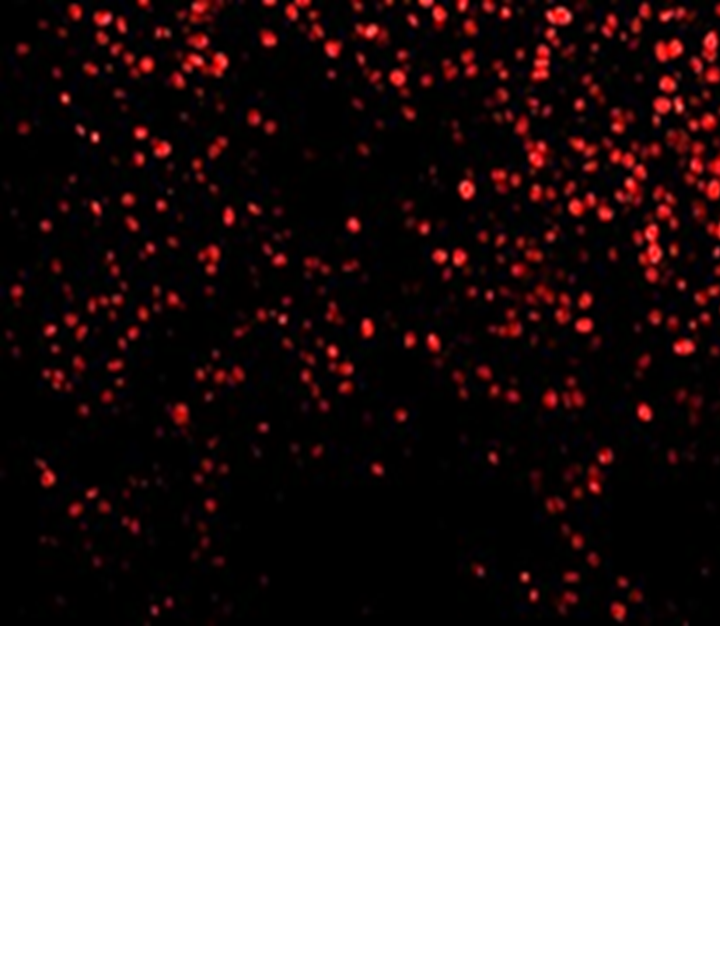

Supplement: Supplementary file 8 — Source data Fig. 5 [file 44321_2025_196_MOESM8_ESM.zip › MM-2024-19448_SourceDataForFig 5/MM-2024-19448_SourceDataForFig 5/MM-2024-19448_SourceDataForFig 5H/MC-38 KI67 Apelin-dm.TIF]

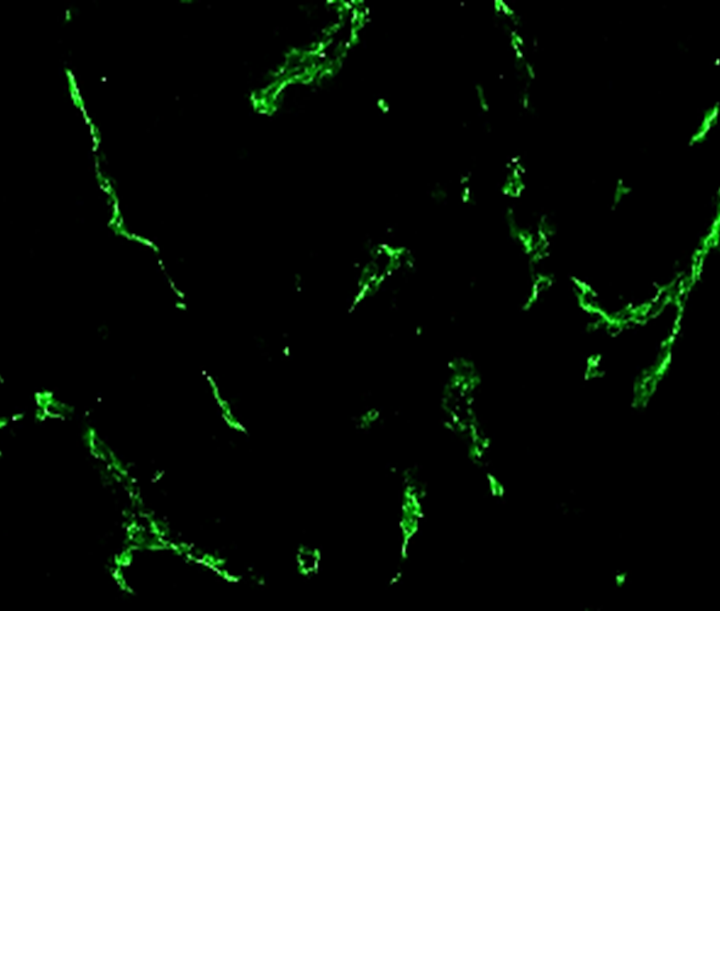

Supplement: Supplementary file 8 — Source data Fig. 5 [file 44321_2025_196_MOESM8_ESM.zip › MM-2024-19448_SourceDataForFig 5/MM-2024-19448_SourceDataForFig 5/MM-2024-19448_SourceDataForFig 5H/MC-38 CD31 Apelin-dm.TIF]

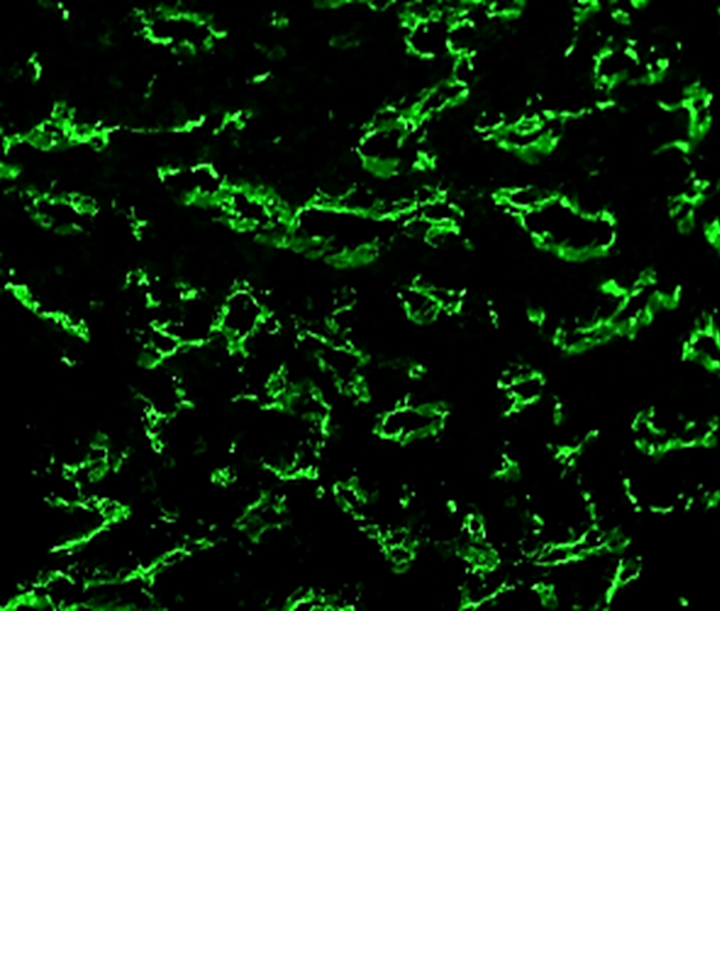

Supplement: Supplementary file 8 — Source data Fig. 5 [file 44321_2025_196_MOESM8_ESM.zip › MM-2024-19448_SourceDataForFig 5/MM-2024-19448_SourceDataForFig 5/MM-2024-19448_SourceDataForFig 5H/MC-38 CD31 Control.TIF]

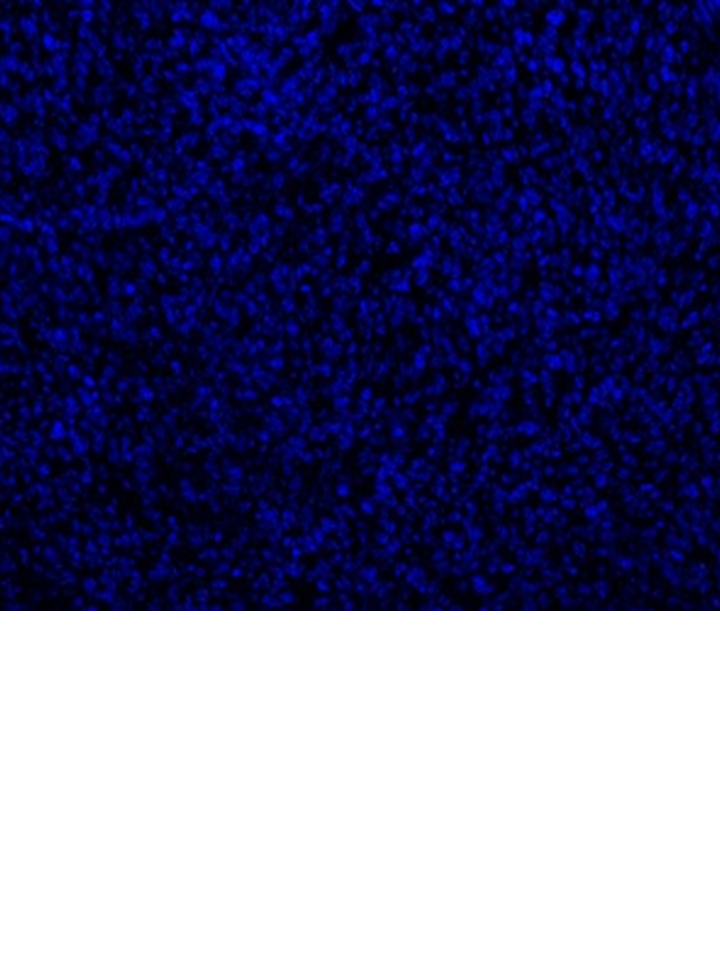

Supplement: Supplementary file 8 — Source data Fig. 5 [file 44321_2025_196_MOESM8_ESM.zip › MM-2024-19448_SourceDataForFig 5/MM-2024-19448_SourceDataForFig 5/MM-2024-19448_SourceDataForFig 5H/MC-38 Dapi Apelin-dm.TIF]

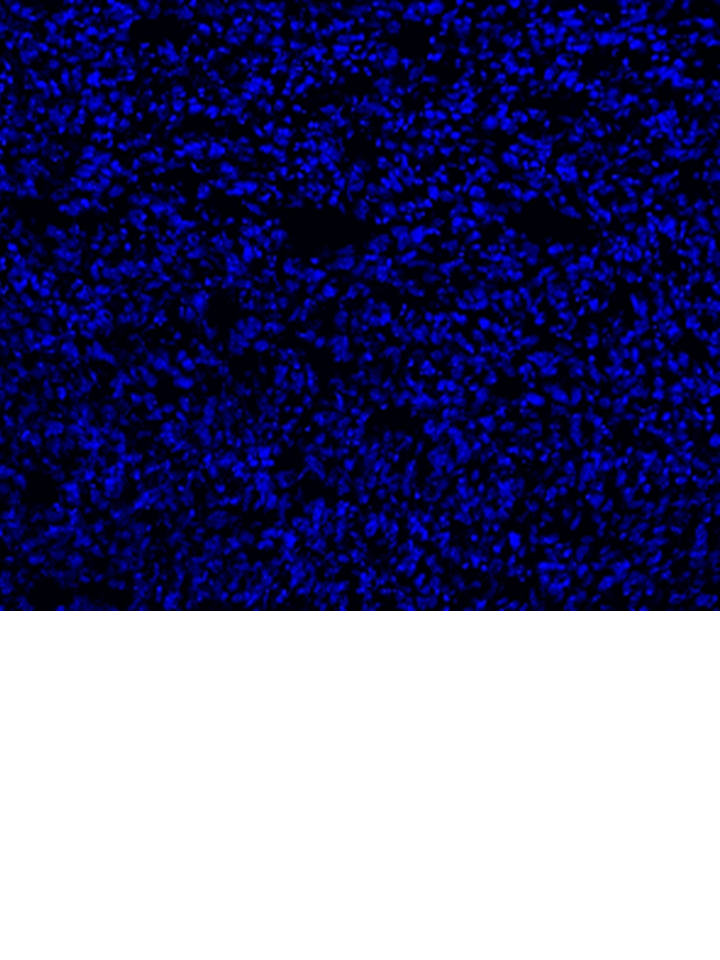

Supplement: Supplementary file 8 — Source data Fig. 5 [file 44321_2025_196_MOESM8_ESM.zip › MM-2024-19448_SourceDataForFig 5/MM-2024-19448_SourceDataForFig 5/MM-2024-19448_SourceDataForFig 5H/MC-38 Dapi Control.TIF]

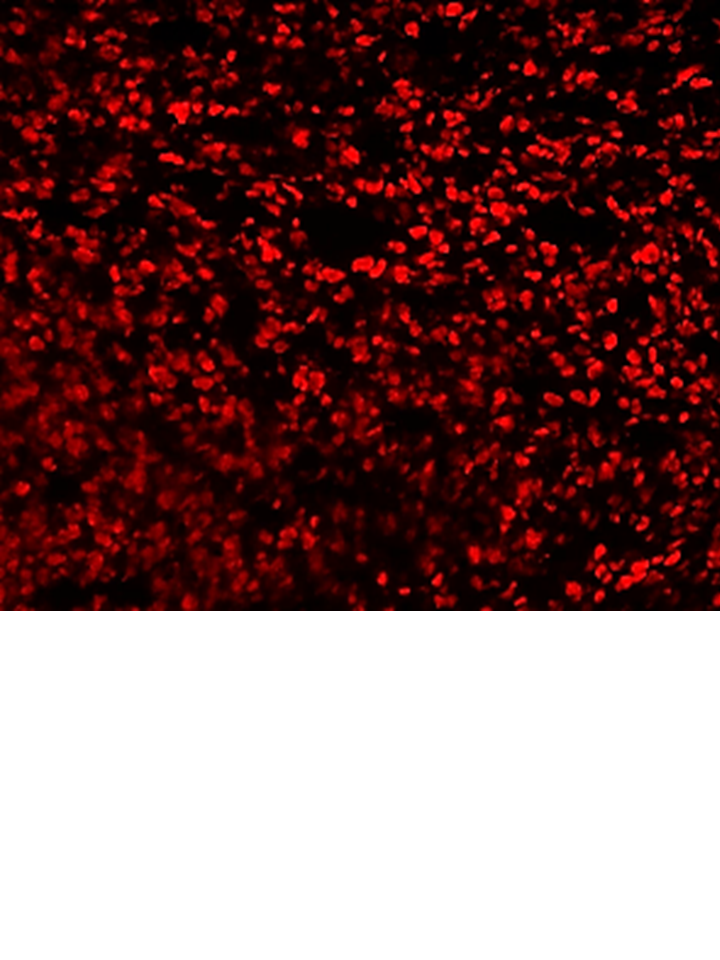

Supplement: Supplementary file 8 — Source data Fig. 5 [file 44321_2025_196_MOESM8_ESM.zip › MM-2024-19448_SourceDataForFig 5/MM-2024-19448_SourceDataForFig 5/MM-2024-19448_SourceDataForFig 5H/MC-38 KI67 Control.TIF]

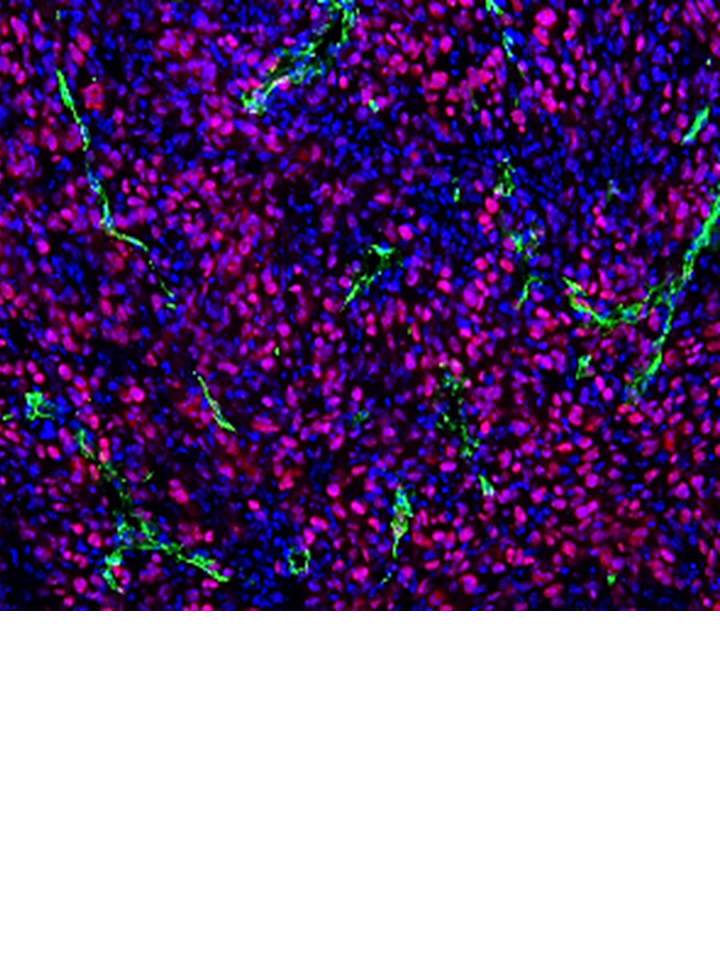

Supplement: Supplementary file 8 — Source data Fig. 5 [file 44321_2025_196_MOESM8_ESM.zip › MM-2024-19448_SourceDataForFig 5/MM-2024-19448_SourceDataForFig 5/MM-2024-19448_SourceDataForFig 5H/MC-38 Merge Apelin-dm.TIF]

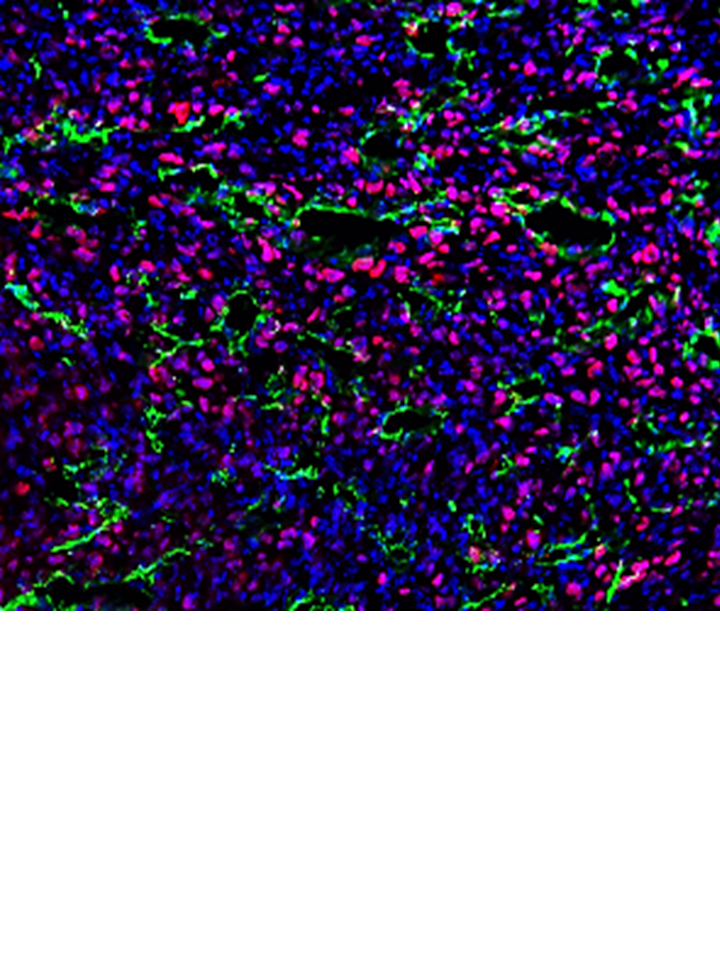

Supplement: Supplementary file 8 — Source data Fig. 5 [file 44321_2025_196_MOESM8_ESM.zip › MM-2024-19448_SourceDataForFig 5/MM-2024-19448_SourceDataForFig 5/MM-2024-19448_SourceDataForFig 5H/MC-38 Merge Control.TIF]

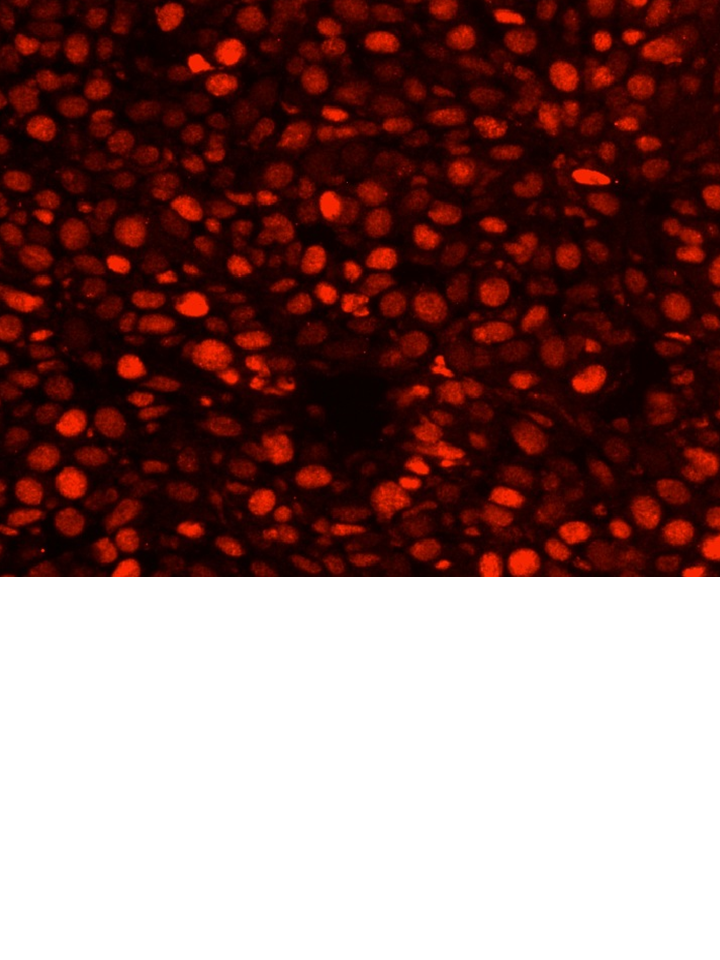

Supplement: Supplementary file 8 — Source data Fig. 5 [file 44321_2025_196_MOESM8_ESM.zip › MM-2024-19448_SourceDataForFig 5/MM-2024-19448_SourceDataForFig 5/MM-2024-19448_SourceDataForFig5G/BIM Apln-dm CT-26.TIF]

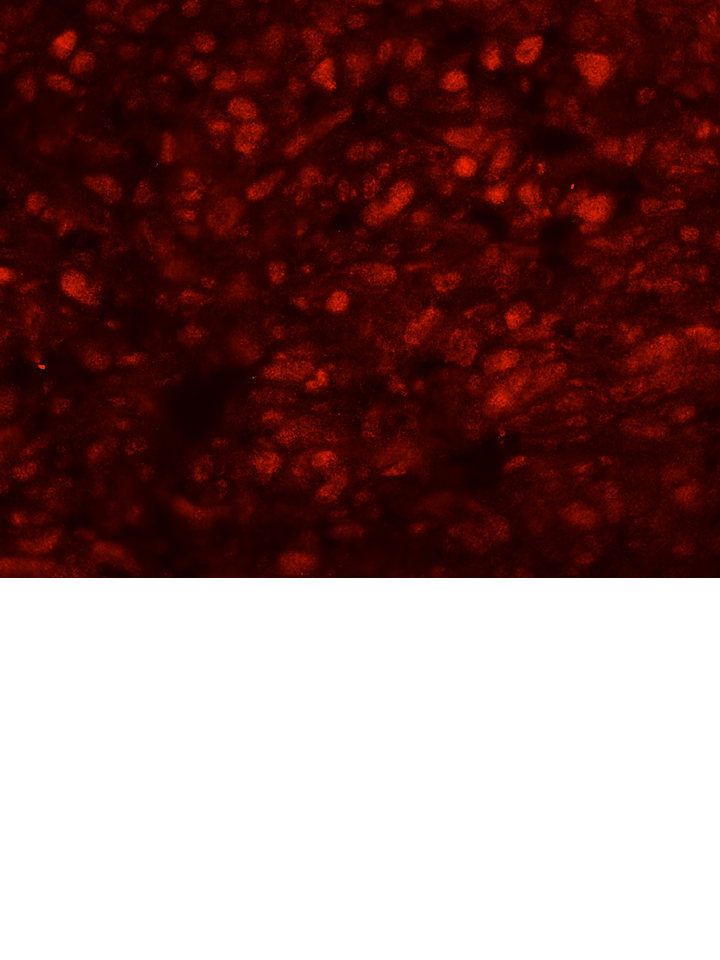

Supplement: Supplementary file 8 — Source data Fig. 5 [file 44321_2025_196_MOESM8_ESM.zip › MM-2024-19448_SourceDataForFig 5/MM-2024-19448_SourceDataForFig 5/MM-2024-19448_SourceDataForFig5G/BIM Apln-dm MC-38.TIF]

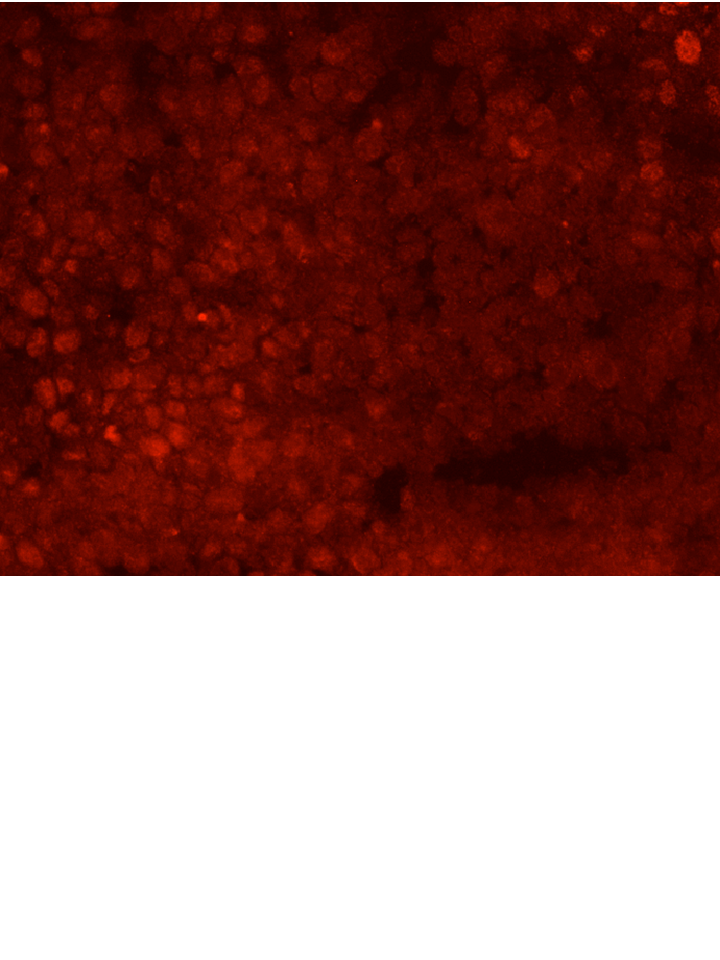

Supplement: Supplementary file 8 — Source data Fig. 5 [file 44321_2025_196_MOESM8_ESM.zip › MM-2024-19448_SourceDataForFig 5/MM-2024-19448_SourceDataForFig 5/MM-2024-19448_SourceDataForFig5G/BIM Control CT-26.TIF]

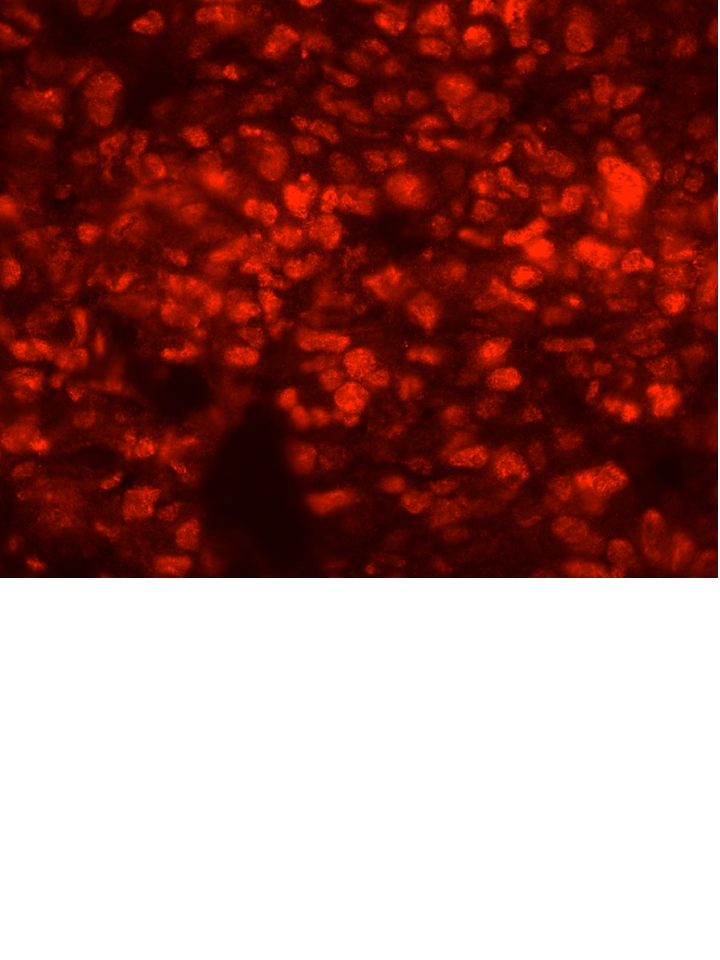

Supplement: Supplementary file 8 — Source data Fig. 5 [file 44321_2025_196_MOESM8_ESM.zip › MM-2024-19448_SourceDataForFig 5/MM-2024-19448_SourceDataForFig 5/MM-2024-19448_SourceDataForFig5G/BIM Control MC-38.TIF]

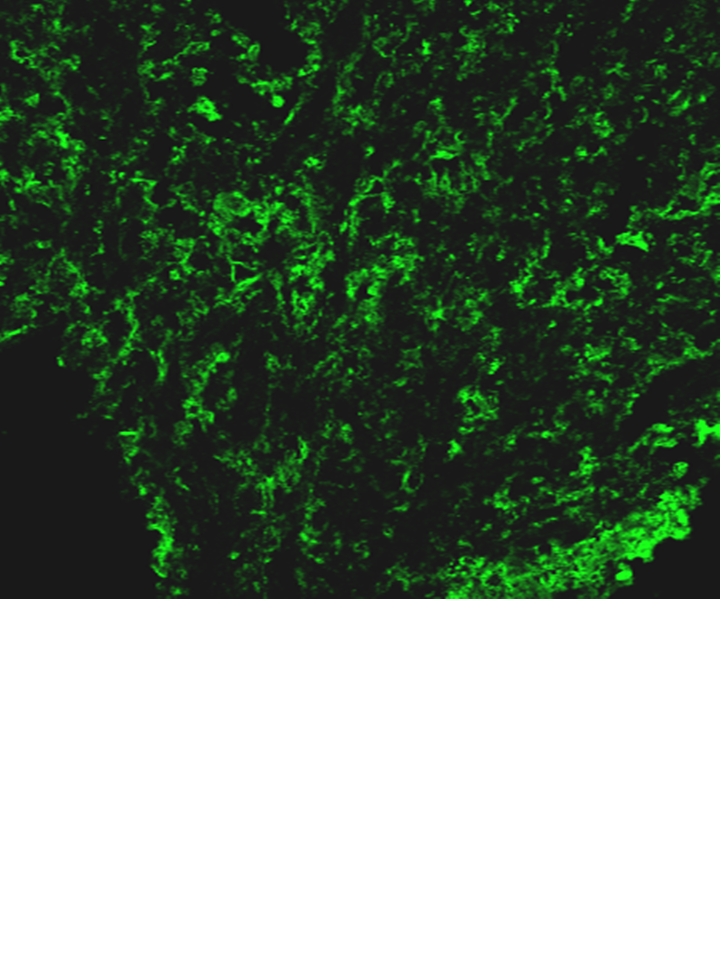

Supplement: Supplementary file 8 — Source data Fig. 5 [file 44321_2025_196_MOESM8_ESM.zip › MM-2024-19448_SourceDataForFig 5/MM-2024-19448_SourceDataForFig 5/MM-2024-19448_SourceDataForFig5O/Apelin cd31.TIF]

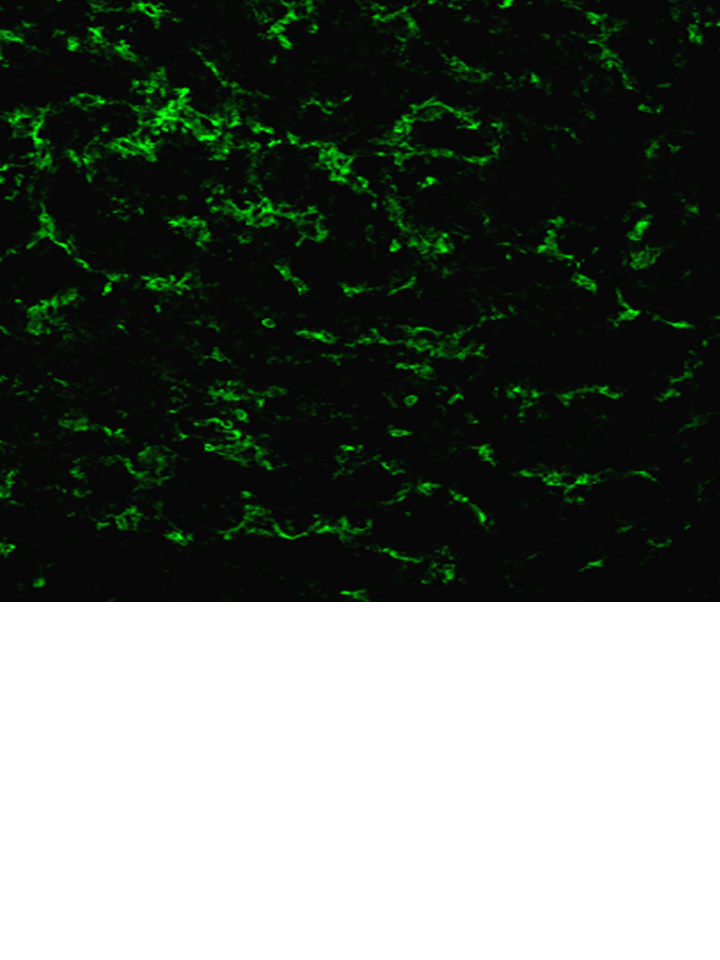

Supplement: Supplementary file 8 — Source data Fig. 5 [file 44321_2025_196_MOESM8_ESM.zip › MM-2024-19448_SourceDataForFig 5/MM-2024-19448_SourceDataForFig 5/MM-2024-19448_SourceDataForFig5O/Apelin-dm cd31.TIF]

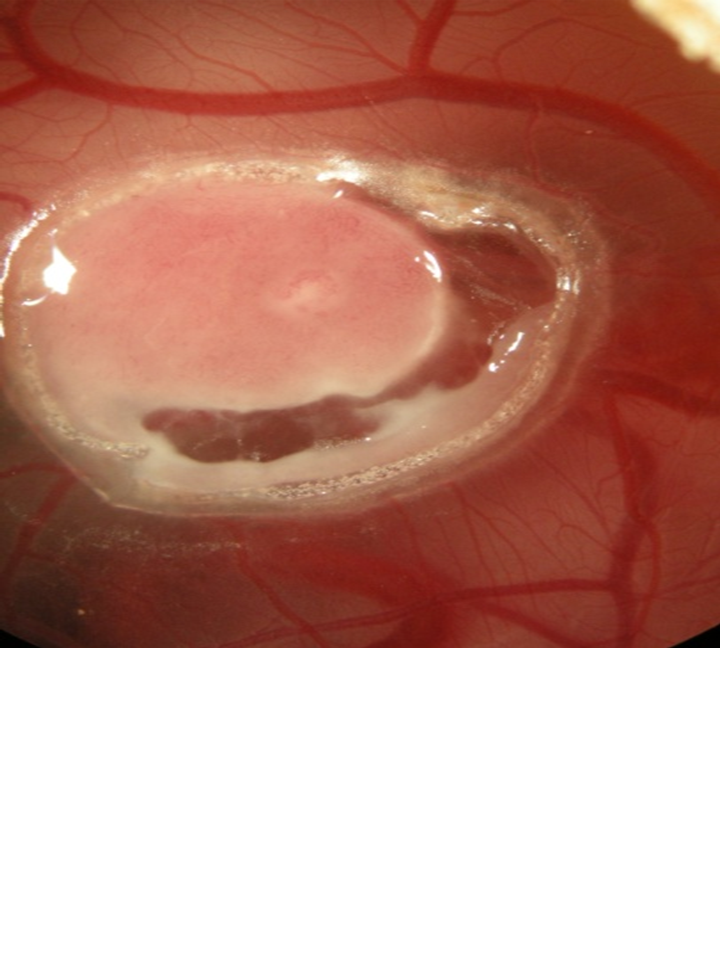

Supplement: Supplementary file 8 — Source data Fig. 5 [file 44321_2025_196_MOESM8_ESM.zip › MM-2024-19448_SourceDataForFig 5/MM-2024-19448_SourceDataForFig 5/MM-2024-19448_SourceDataForFig5O/CAM Apelin.TIF]

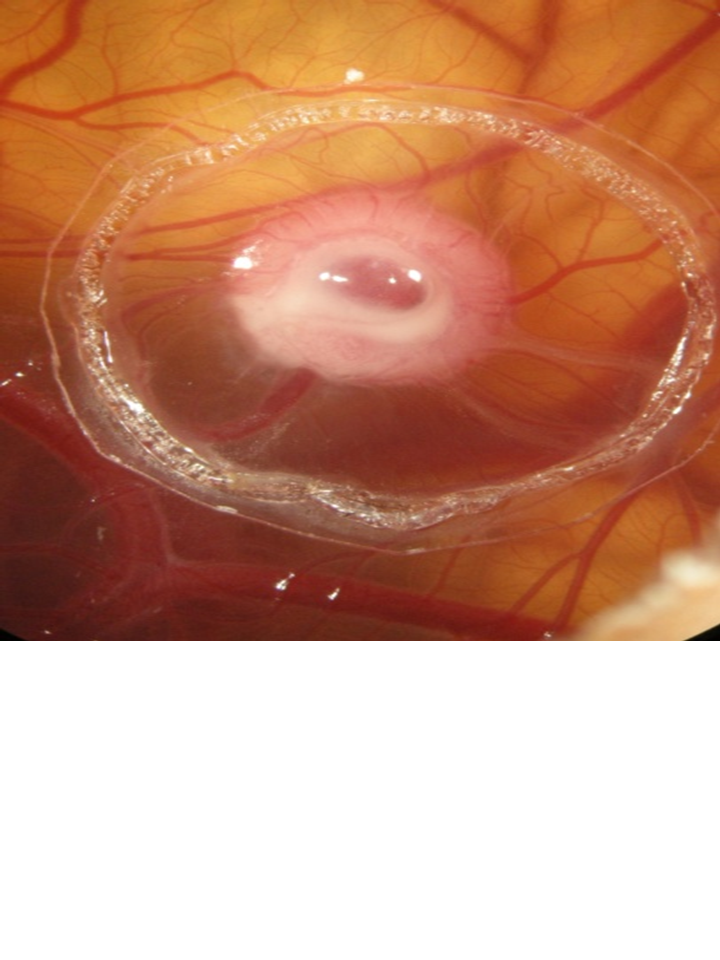

Supplement: Supplementary file 8 — Source data Fig. 5 [file 44321_2025_196_MOESM8_ESM.zip › MM-2024-19448_SourceDataForFig 5/MM-2024-19448_SourceDataForFig 5/MM-2024-19448_SourceDataForFig5O/CAM Apelin-dm.TIF]

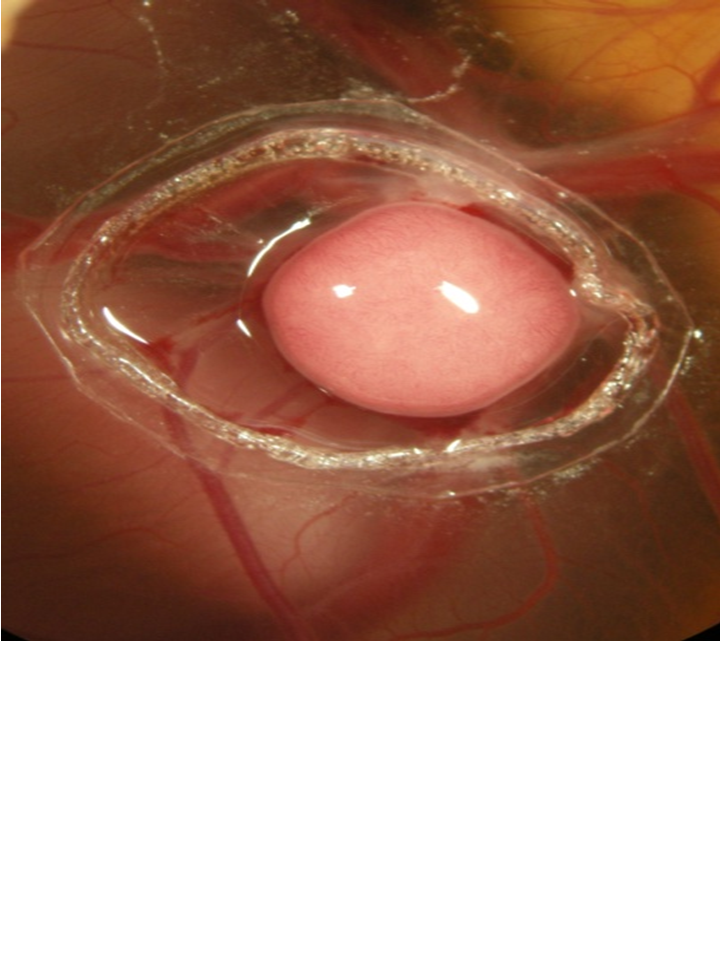

Supplement: Supplementary file 8 — Source data Fig. 5 [file 44321_2025_196_MOESM8_ESM.zip › MM-2024-19448_SourceDataForFig 5/MM-2024-19448_SourceDataForFig 5/MM-2024-19448_SourceDataForFig5O/CAM Control.TIF]

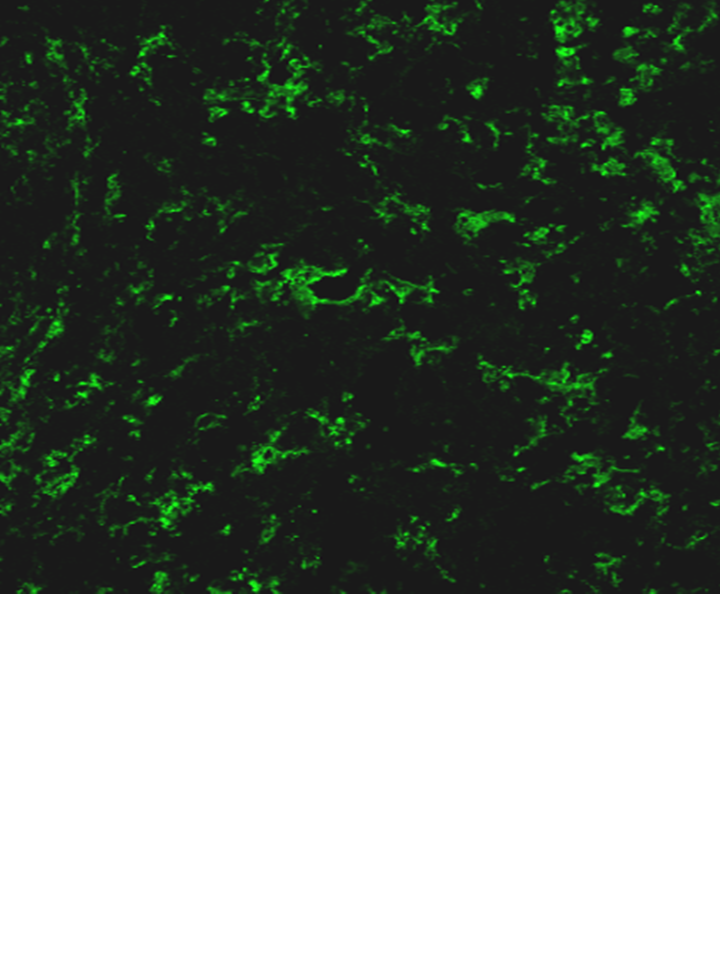

Supplement: Supplementary file 8 — Source data Fig. 5 [file 44321_2025_196_MOESM8_ESM.zip › MM-2024-19448_SourceDataForFig 5/MM-2024-19448_SourceDataForFig 5/MM-2024-19448_SourceDataForFig5O/Control cd31.TIF]

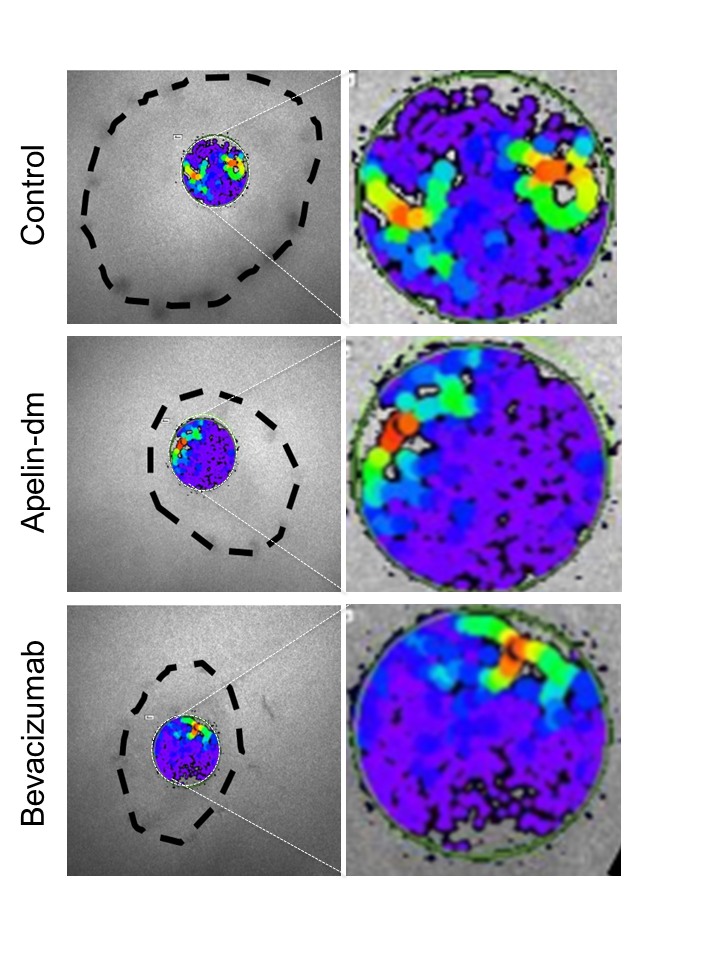

Supplement: Supplementary file 8 — Source data Fig. 5 [file 44321_2025_196_MOESM8_ESM.zip › MM-2024-19448_SourceDataForFig 5/MM-2024-19448_SourceDataForFig 5/MM-2024-19448_SourceDataForFig5R/Fig 5R.tif]

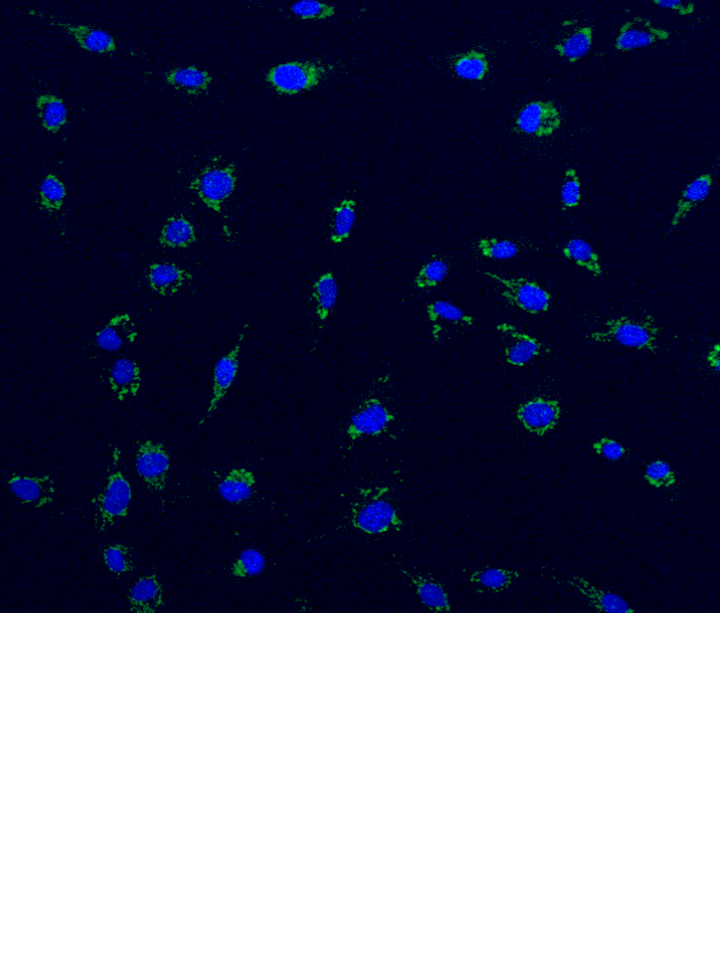

Supplement: Supplementary file 9 — Source data Fig. 6 [file 44321_2025_196_MOESM9_ESM.zip › MM-2024-19448_SourceDataForFig 6/MM-2024-19448_SourceDataForFig 6B/Slide1.TIF]

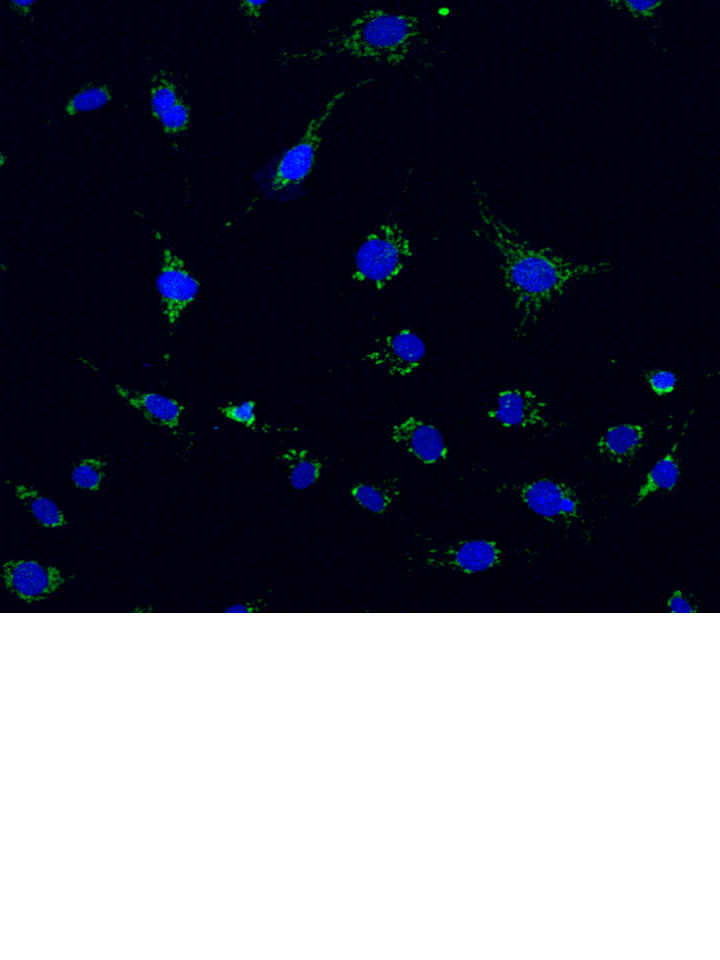

Supplement: Supplementary file 9 — Source data Fig. 6 [file 44321_2025_196_MOESM9_ESM.zip › MM-2024-19448_SourceDataForFig 6/MM-2024-19448_SourceDataForFig 6B/Slide2.TIF]

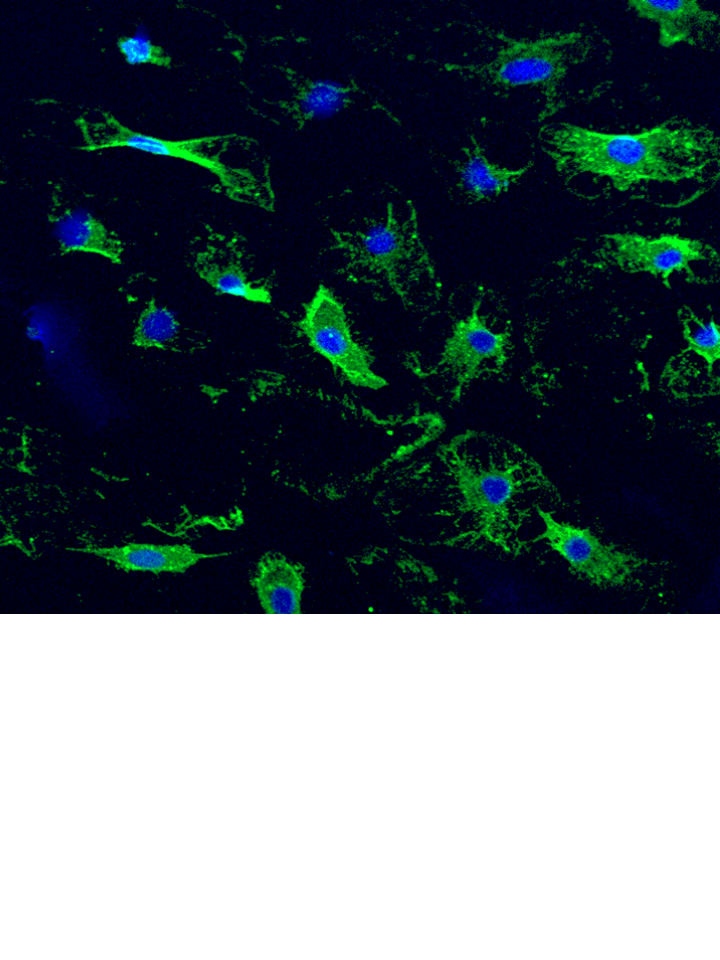

Supplement: Supplementary file 9 — Source data Fig. 6 [file 44321_2025_196_MOESM9_ESM.zip › MM-2024-19448_SourceDataForFig 6/MM-2024-19448_SourceDataForFig 6B/Slide3.TIF]

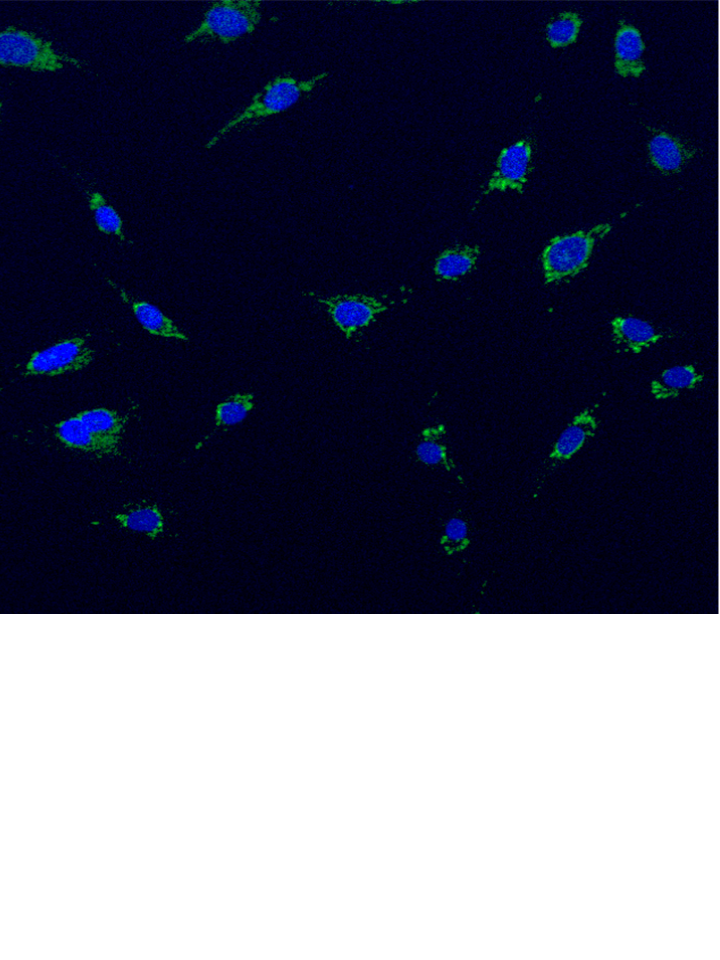

Supplement: Supplementary file 9 — Source data Fig. 6 [file 44321_2025_196_MOESM9_ESM.zip › MM-2024-19448_SourceDataForFig 6/MM-2024-19448_SourceDataForFig 6B/Slide4.TIF]

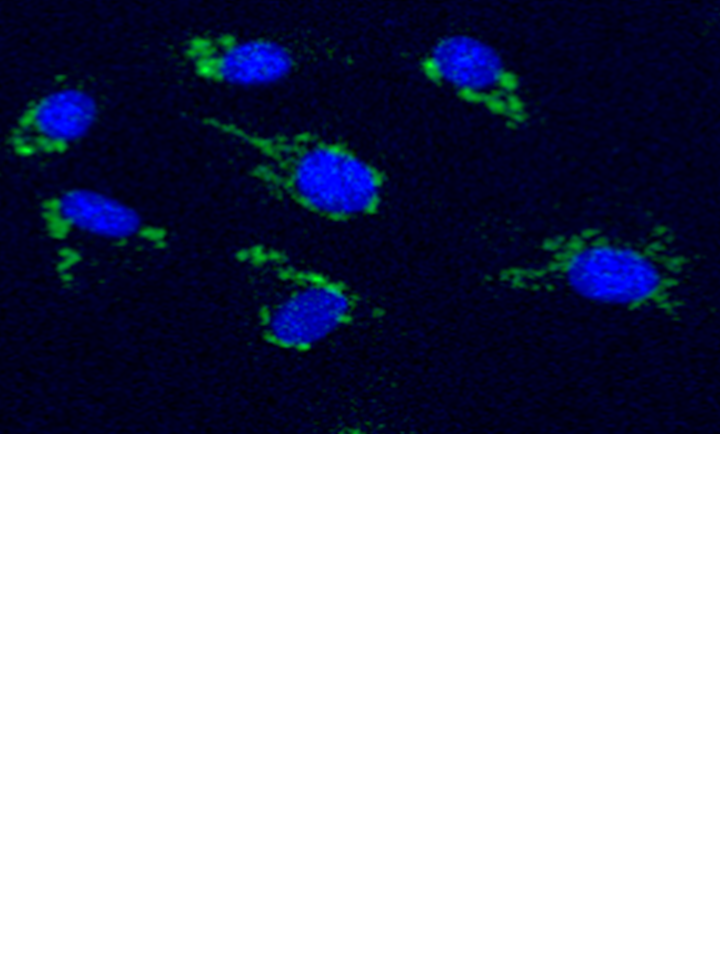

Supplement: Supplementary file 9 — Source data Fig. 6 [file 44321_2025_196_MOESM9_ESM.zip › MM-2024-19448_SourceDataForFig 6/MM-2024-19448_SourceDataForFig 6B/Slide5.TIF]

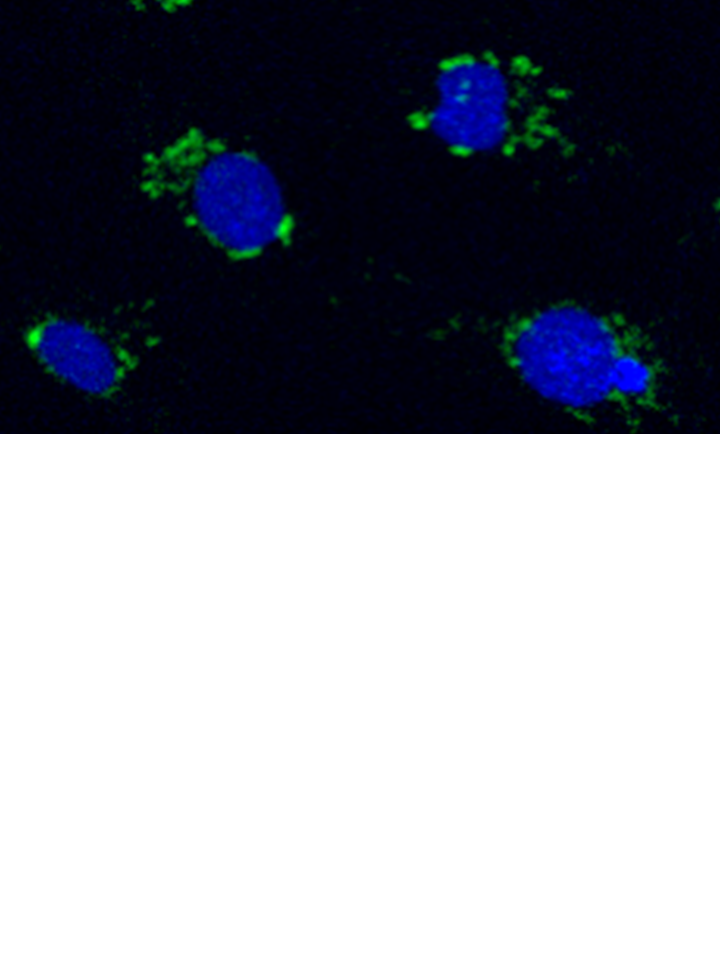

Supplement: Supplementary file 9 — Source data Fig. 6 [file 44321_2025_196_MOESM9_ESM.zip › MM-2024-19448_SourceDataForFig 6/MM-2024-19448_SourceDataForFig 6B/Slide6.TIF]

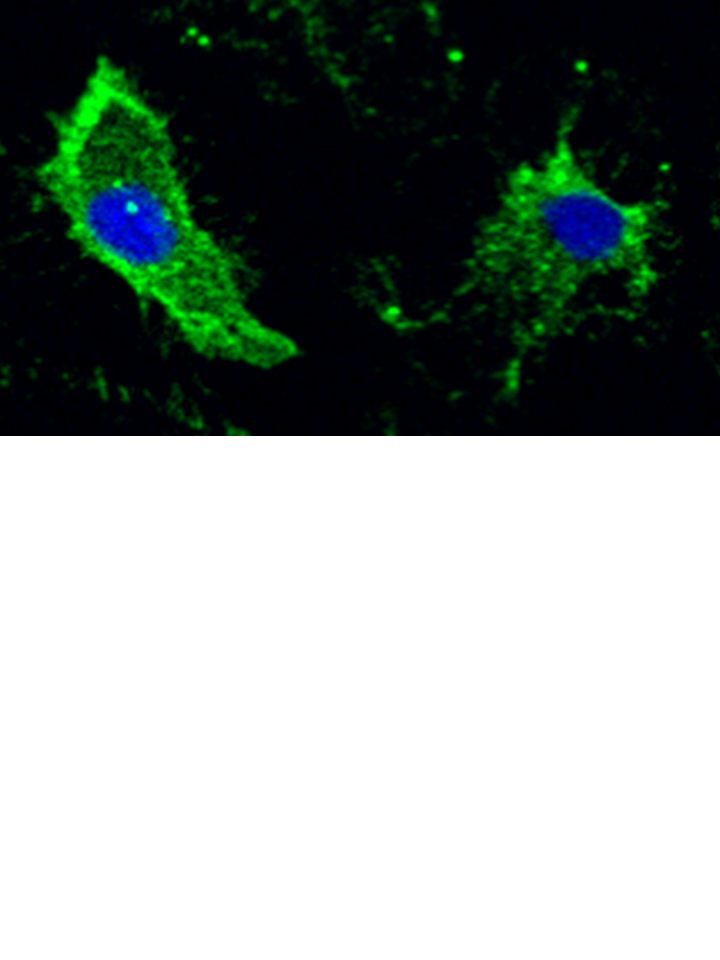

Supplement: Supplementary file 9 — Source data Fig. 6 [file 44321_2025_196_MOESM9_ESM.zip › MM-2024-19448_SourceDataForFig 6/MM-2024-19448_SourceDataForFig 6B/Slide7.TIF]

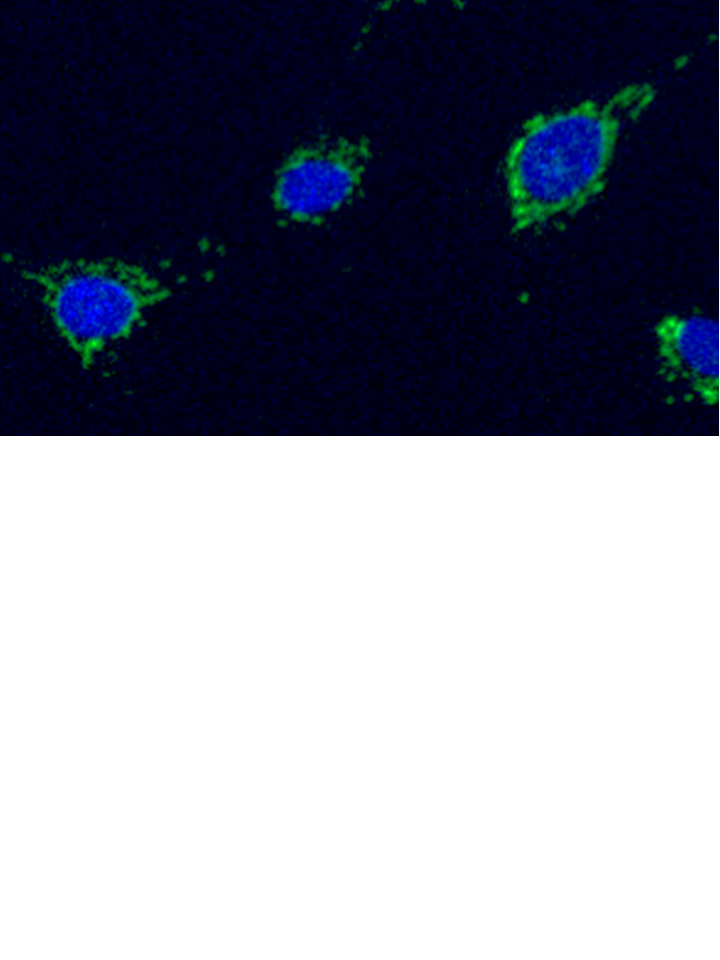

Supplement: Supplementary file 9 — Source data Fig. 6 [file 44321_2025_196_MOESM9_ESM.zip › MM-2024-19448_SourceDataForFig 6/MM-2024-19448_SourceDataForFig 6B/Slide8.TIF]

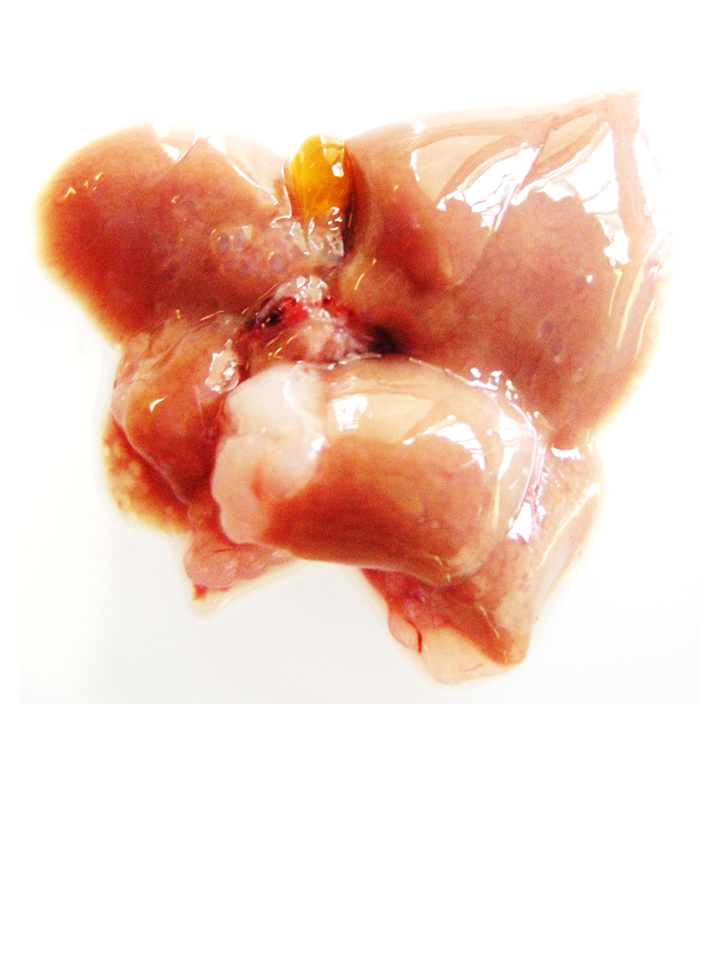

Supplement: Supplementary file 9 — Source data Fig. 6 [file 44321_2025_196_MOESM9_ESM.zip › MM-2024-19448_SourceDataForFig 6/MM-2024-19448_SourceDataForFig6J/Apln-dm.TIF]

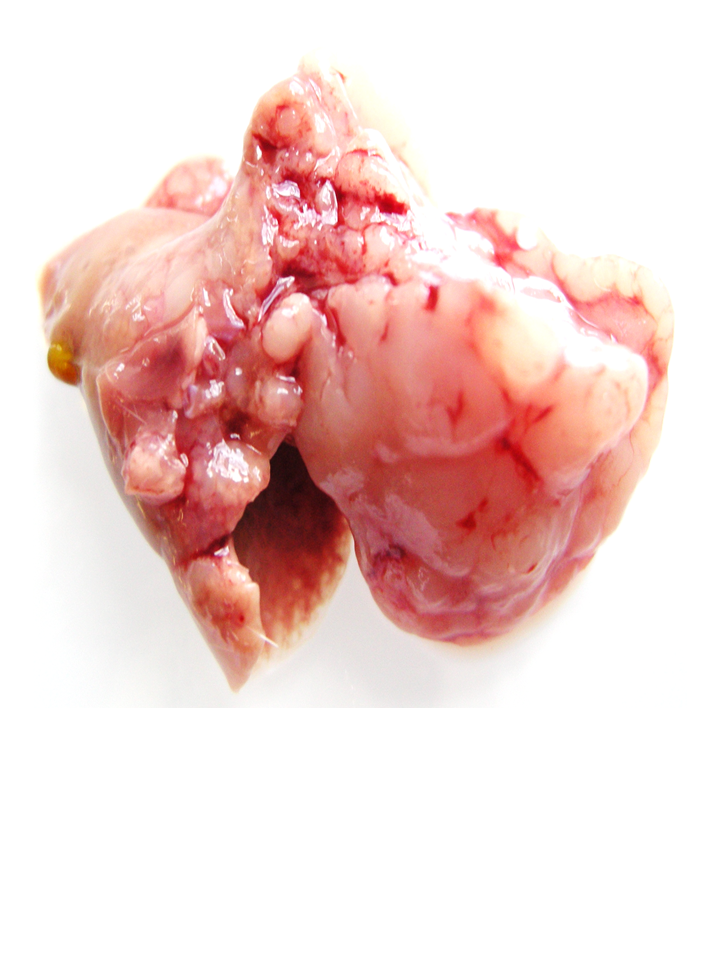

Supplement: Supplementary file 9 — Source data Fig. 6 [file 44321_2025_196_MOESM9_ESM.zip › MM-2024-19448_SourceDataForFig 6/MM-2024-19448_SourceDataForFig6J/Control Apln-dm.TIF]

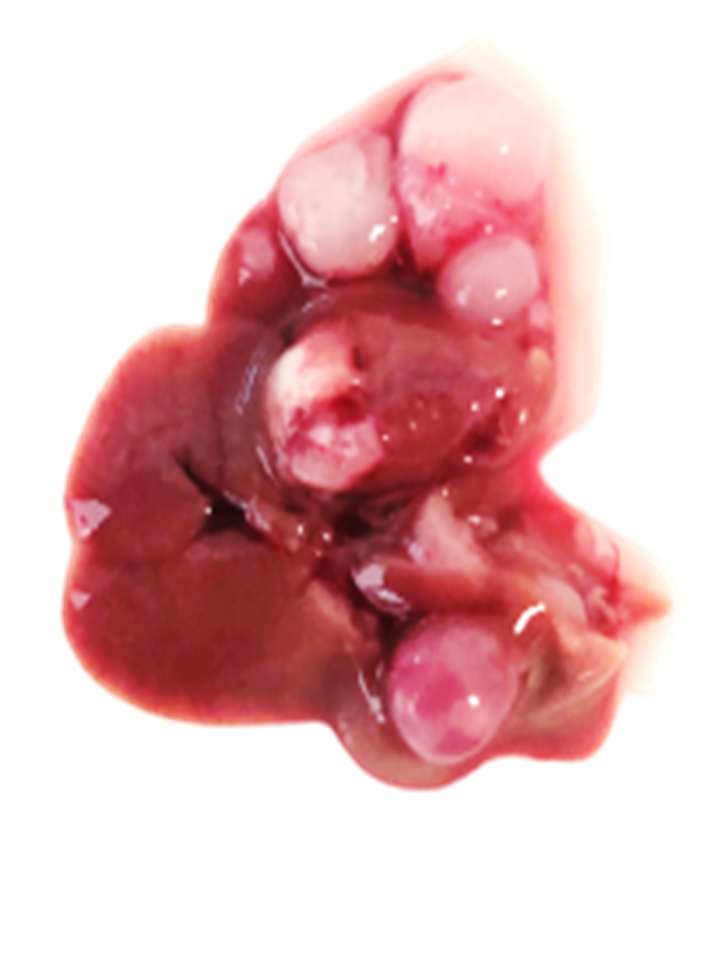

Supplement: Supplementary file 9 — Source data Fig. 6 [file 44321_2025_196_MOESM9_ESM.zip › MM-2024-19448_SourceDataForFig 6/MM-2024-19448_SourceDataForFig6J/Control MM54.TIF]

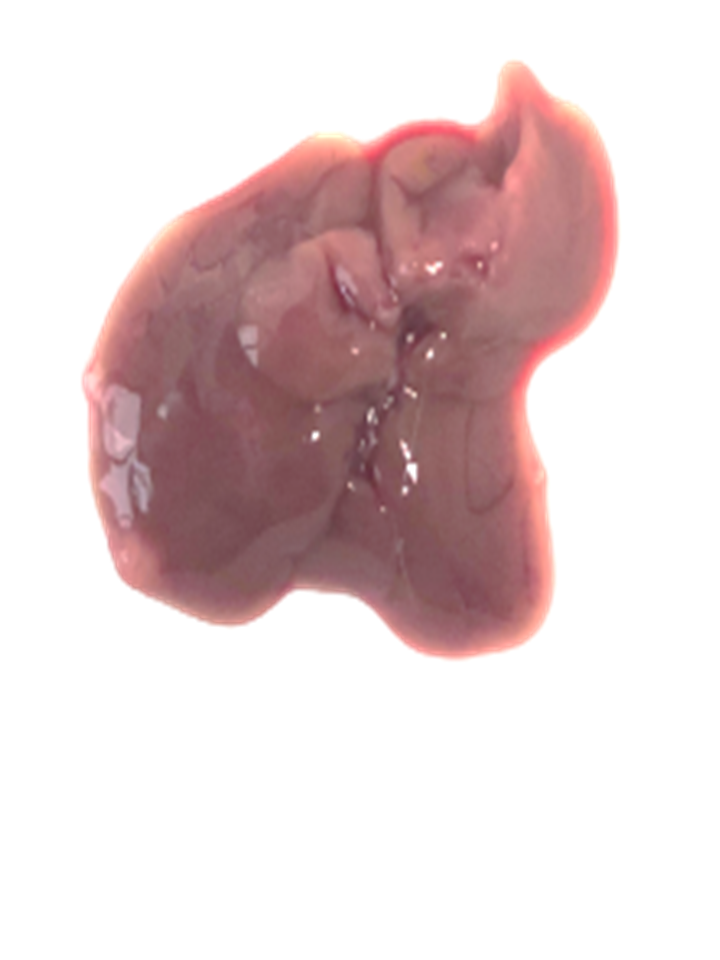

Supplement: Supplementary file 9 — Source data Fig. 6 [file 44321_2025_196_MOESM9_ESM.zip › MM-2024-19448_SourceDataForFig 6/MM-2024-19448_SourceDataForFig6J/MM54.TIF]
