# Supplementary material for: Repression of apelin Furin cleavage sites provides antimetastatic strategy in colorectal cancer
Source: EMBO Mol Med. 2025 Feb 17;17(3):504–34. doi: 10.1038/s44321-025-00196-5 (PMC11904221; doi:10.1038/s44321-025-00196-5)
Supplement: Supplementary file 10 — Expanded View Figures [file 44321_2025_196_MOESM10_ESM.pdf]

Expanded View Figures

Human.AAF25815.1
MNLRLCVQALLLLWLSLTAVCGGSLMPLPDGNGLEDGNVRHLVQPRGSRNGPGPWQGG*RR*KF*RR*QRPRLS HKGPMPF

Gorilla.XP_018874376.1
MNLRLCVQALLLLWLSLTAVCGGSLMPLPDGNGLEEGNVRHLVQPRGSRNGPGPWQGG*RR*KF*RR*QRPRLS HKGPMPF

Rat. AAF25814.1
MNLSFCVQALLLLWLSLTAVCGVPLMLPPDGKGLEEGNMRYLVKPRTSRTGPGAWQGG*RR*KF*RR*QRPRLS HKGPMPF

Mouse. NP_038940.1
MNLRLCVQALLLLWLSLTAVCGVPLMLPPDGTGLEEGSMRYLVKPRTSRTGPGAWQGG*RR*KF*RR*QRPRLS HKGPMPF

Zebrafish. NP_001159596.1
MNVKILTLVIVLVVSLLCSASAGPMASTEHSKEIEEVGSMRTPLRQNPARAGRSQRPAGW*RRRR*PRPRLSHKGPMPF

Figure EV1. Comparison of apelin precursor sequences from various species.

Represented are the putative peptide sequences of human, Gorilla, rat, mouse and zebrafish. The positions of the convertase general motif (K/R)-(X)n-(K/R)↓, where $n = 0, 2, 4$ or 6 and X (green and in italics) are conserved. The National Center for Biotechnology Information (NCBI) accession number for each sequence is given.

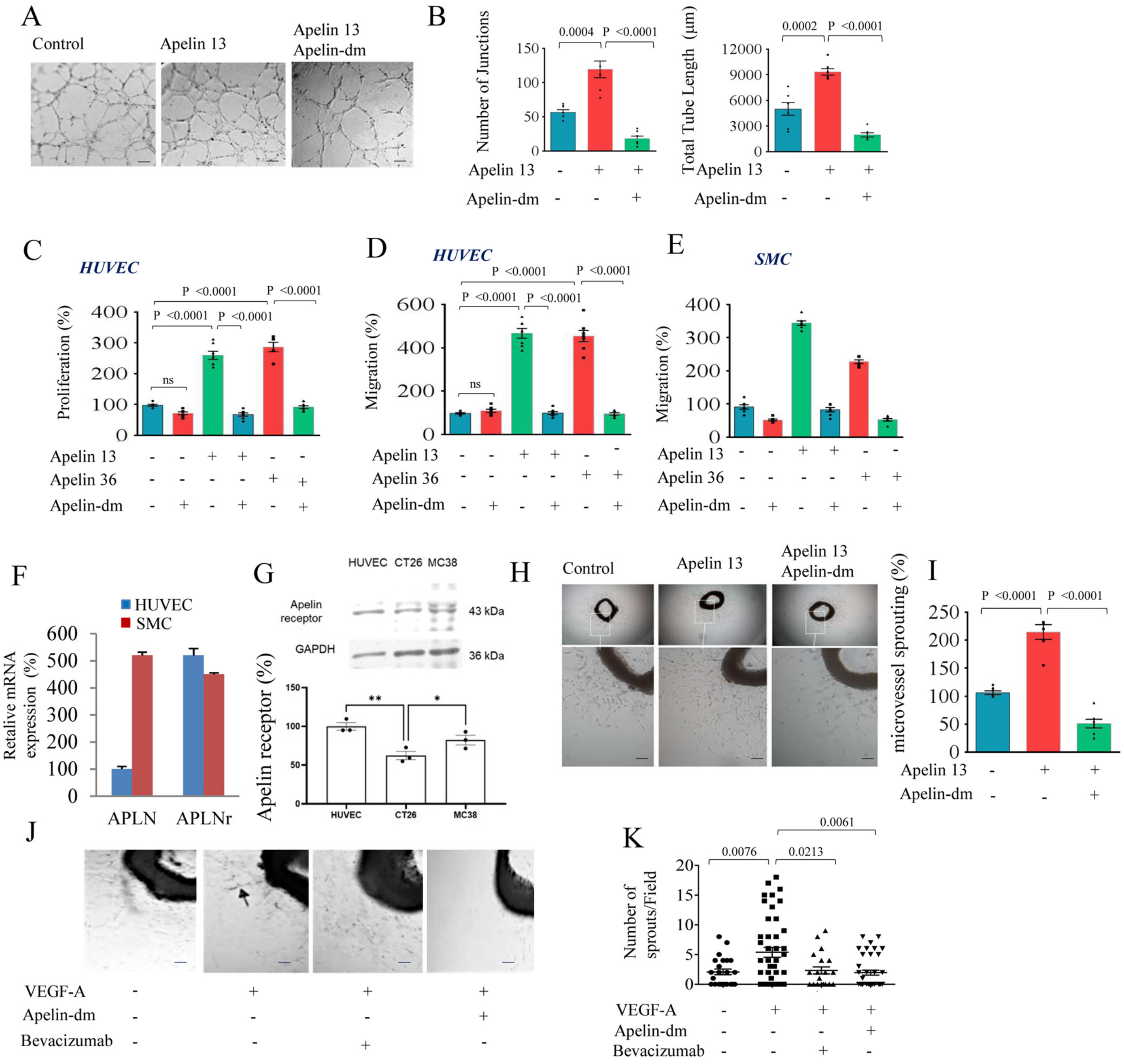

Figure EV2. Effects of apelin-dm on endothelial tube formation, cell migration, proliferation, and aortic ring microvessel sprouting.

(**A**) Representative images of apelin-dm and/or apelin peptides effect on tube-like structure formation by HUVEC. Scale bar indicates 250 µm. (**B**) Effect of Apelin-dm on apelin-induced tube-like structure formation measured by the number of junctions and tubule length. (**C**, **D**) Apelin-dm inhibits HUVEC proliferation (**C**) and migration (**D**) and smooth muscle cells (SMC) migration (**E**) induced by apelin-13 and 36 peptides. (**F**) Relative expression of APLN and APLNR mRNA in Huvec and SMC expressed relative to APLN mRNA abundance in HUVEC assigned 100%. (**G**) Western blotting analysis of apelin receptor expression in HUVEC, CT-26 and MC-38 cells, expressed relative to apelin receptor protein abundance in HUVEC assigned 100%. (**H**) Representative images of apelin-dm peptide effect alone or on apelin-mediated aortic ring microvessel sprouting. (**I**) Quantification of aortic ring vascular sprout surface per aortic ring relative to control untreated aorta (100%). (**J**) Representative images of apelin-dm peptide or Bevacizumab effect on VEGF-mediated aortic ring microvessel sprouting. Scale bar indicates 250 µm. (**K**) Quantification of number of sprouts per field. The data are representative of three independent experiments. n.s. not significant. All values represent the mean ± s.e.m. Significant differences *P* were determined by two-way ANOVA.

 © The Author(s)

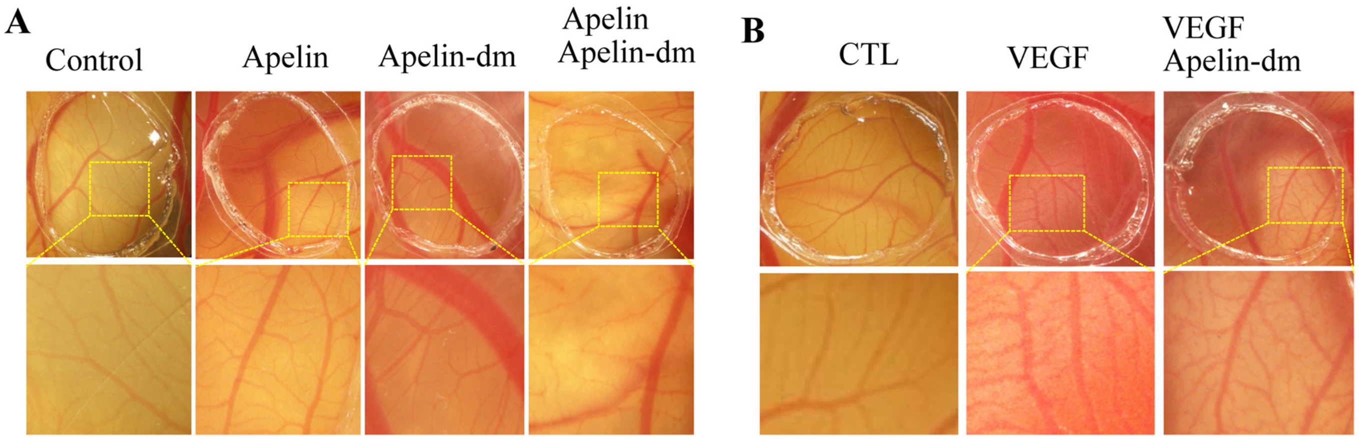

Figure EV3. Effects of apelin-dm on apelin- and VEGF-induced vessel formation in the CAM Assay.

(**A**, **B**) Representative images of apelin-dm effect alone, on apelin (**A**) or on VEGF-mediated (**B**) vessel formation in the CAM assay (corresponding to Fig. 3D, H). On day 9, the CAM received either vehicle (Control), 100 nM of apelin, 100 nM of apelin-dm or both. For A, the photographs shown were taken after treatments (24 h) and are representative of the results obtained in an additional 5 eggs per group. For (**B**), on day 9, the CAM received either vehicle (Control), 20 ng VEGF, or VEGF and apelin-dm. The photographs shown were taken after treatments (24 h) and are representative of the results obtained in an additional 5 eggs per group. high-magnification pictures of CAMs are also indicated.

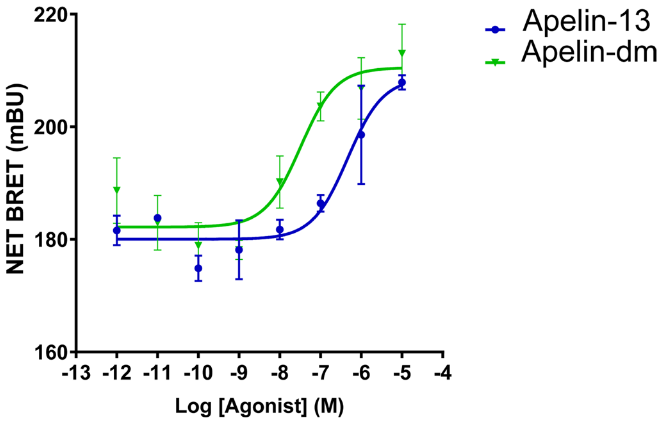

	Ape 13	Ape 36 DM
LogEC50	-6.335	-7.493
EC50	4.626e-007	3.213e-008

Figure EV4. Dose-dependent increase in BRET Signal by apelin and apelin-dm, Indicating apelin receptor internalization.

HEK293T cells transiently transfected with apelin receptor-Rluc and Rab5-YFP were used to measure the Bioluminescence Resonance Energy Transfer (BRET) signal, indicative of apelin receptor internalization in the presence of various concentration of apelin and apelin-dm. Net BRET signals were determined by subtracting the BRET signal from cells expressing only Rluc-tagged apelin receptor from those co-expressing Rluc-tagged apelin receptor and YFP-tagged Rab5.

 © The Author(s)

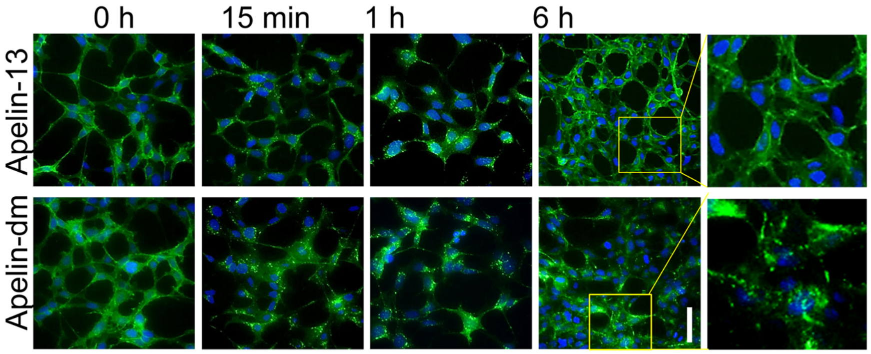

Figure EV5. Representative images of apelin-13 and apelin-dm peptides effect on apelin receptor internalization in HEK293A cells stably expressing apelin receptor-EGFP fusion protein.

The data are representative of three independent experiments. Six hours after peptides washout, the apelin receptor seemed to be mainly returned to the cell surface in apelin-13-treated cells. In contrast, in cells treated with apelin-dm, the intracellular vesicles of the apelin receptor remained internalized. Scale bar, 100 µm.

© The Author(s)
